# Prevalence and Levels of Thyroid Autoantibodies in Polycystic Ovary Syndrome—Impact of TSH- and BMI-Matched Comparisons: A Systematic Review and Meta-Analysis

**DOI:** 10.3390/ijms26157525

**Published:** 2025-08-04

**Authors:** Jakub Kwiatkowski, Nicole Akpang, Lucja Zaborowska, Artur Ludwin

**Affiliations:** 1 1st Department of Obstetrics and Gynecology, Medical University of Warsaw, 02-015 Warsaw, Poland; s085160@student.wum.edu.pl (J.K.); s084954@student.wum.edu.pl (N.A.); zaborowska.lucja@doctoral.uj.edu.pl (L.Z.); 2 Doctoral School of Medical and Health Sciences, Jagiellonian University Collegium Medicum, 31-530 Cracow, Poland

**Keywords:** polycystic ovary syndrome, PCOS, anti-thyroid peroxidase antibodies, anti-thyroglobulin antibodies, anti-TPO, anti-TG, autoimmune thyroid disease, Hashimoto’s thyroiditis, autoimmunity, antibodies

## Abstract

Polycystic ovary syndrome (PCOS) is a complex condition affecting women of reproductive age, characterized by menstrual irregularities, hyperandrogenism, polycystic ovarian morphology, and low-grade inflammation accompanied by oxidative stress and increased autoimmune risk, particularly Hashimoto’s thyroiditis. Many studies have examined thyroid autoantibodies—anti-thyroid peroxidase antibodies (anti-TPO) and anti-thyroglobulin antibodies (anti-TG)—in PCOS; however, observed differences in baseline thyroid-stimulating hormone (TSH) levels and body mass indices (BMIs) impede a direct interpretation of the results. This systematic review and meta-analysis aimed to summarize the available evidence on the prevalence and levels of anti-TPO and anti-TG in women with PCOS. We conducted a systematic search of PubMed, Scopus, and Embase, which yielded 40 eligible, observational studies including 6045 women with PCOS and 4527 controls. Subgroup analyses were conducted separately for TSH- and BMI-matched populations. Anti-TPO prevalence (odds ratio [OR] = 2.03; 95% confidence interval [CI]: 1.35–3.04; *p* = 0.0006) and levels (standardized mean difference [SMD] = 0.63; 95% CI: 0.37–0.88; *p* < 0.00001) were significantly higher in PCOS patients. Anti-TG prevalence (OR = 1.92; 95% CI: 1.23–3.01; *p* = 0.004) and levels (SMD = 0.41; 95% CI: 0.18–0.64; *p* = 0.0004) were also significantly elevated. In matched subgroups, prevalence differences were no longer significant, though anti-TPO levels remained significantly elevated and anti-TG levels were borderline significant in the TSH-matched subgroup of PCOS women. Although differences in thyroid autoantibody prevalence in women with PCOS appear to be driven by elevated TSH levels and BMIs, the persistently increased antibody levels in the majority of matched subgroups suggest that PCOS itself may contribute independently to heightened autoimmune activation.

## 1. Introduction

Polycystic ovary syndrome (PCOS) is a common endocrine disorder affecting not only women of reproductive age but also adolescents and postmenopausal women [1]. It is estimated that PCOS affects approximately 5–15% of women in the general population worldwide [2]. The clinical manifestations are highly heterogeneous, including hyperandrogenism (hirsutism, acne, androgenic alopecia), polycystic ovarian morphology (PCOM), and ovulatory dysfunction (anovulation, oligo-ovulation) [3]. These clinical abnormalities can ultimately lead to infertility [3]. Patients with PCOS exhibit an increased risk of metabolic diseases as well as significant psychiatric disturbances, including anxiety and depression [4,5,6]. The diagnosis of polycystic ovary syndrome (PCOS) remains controversial due to the use of various diagnostic criteria across different regions, which complicates the interpretation and synthesis of available studies [7]. The most widely used are the Rotterdam criteria, which stipulate that a diagnosis of PCOS requires the presence of at least two of the following three features: (1) polycystic ovarian morphology (PCOM), (2) ovulatory dysfunction (anovulation or oligo-ovulation), and (3) clinical or biochemical signs of hyperandrogenism [8]. Even these commonly accepted criteria are subject to debate, particularly regarding the risk of overdiagnosis [1]. Although PCOS was first described by Stein and Leventhal in 1935, its exact pathogenesis remains unclear, resulting in a lack of targeted pharmacological interventions [9,10]. Consequently, treatment is predominantly symptomatic and frequently applied off-label [11]. Beyond the well-known issue of infertility, PCOS is also a significant contributor to cardiovascular and metabolic morbidity. It has been linked to type 2 diabetes, obesity, dyslipidemia, hypertension, and autoimmune diseases [3,12]. Additionally, PCOS is associated with enhanced oxidative stress, chronic low-grade inflammation, and a range of hormonal disturbances [13,14,15]. These include altered pulsatile secretion of gonadotropin-releasing hormone (GnRH), elevated testosterone levels, reduced sex hormone-binding globulin (SHBG), and an increased luteinizing hormone (LH)-to-follicle-stimulating hormone (FSH) ratio [13,14]. Other abnormalities include low progesterone levels, relatively elevated estrogen levels, insulin resistance, and hyperinsulinemia [13,14]. Collectively, these factors contribute to the heightened risk of comorbid conditions, notably those with an autoimmune basis [16]. The link between the frequent co-occurrence of polycystic ovary syndrome (PCOS) and autoimmune thyroid diseases has been extensively studied in recent years.

Autoimmune thyroid disease (AITD) is the leading cause of hypothyroidism in young women, affecting approximately 5% to 20% of the general reproductive-age female population [17]. It is a T cell-mediated, organ-specific autoimmune disorder in which the immune response is directed against the thyroid gland [18]. Diagnostic markers of AITD include anti-thyroid peroxidase antibodies (anti-TPO), anti-thyroglobulin antibodies (anti-TG), as well as a characteristic ultrasound image of a hypoechoic thyroid [19]. To date, systematic reviews and meta-analyses have confirmed the higher prevalence of AITD among patients with PCOS [17,20,21]. Among women with PCOS, AITD is considered the most common autoimmune comorbidity, with an estimated prevalence of around 25% [21]. According to the Rotterdam criteria, diagnosing PCOS requires the exclusion of several disorders that may mimic its clinical presentation, including thyroid diseases [8]. However, these conditions often coexist and their concurrence is unfortunately associated with a higher risk of metabolic complications [22,23,24]. The exact cause of this frequent co-occurrence and the underlying pathophysiological relationship remain unknown [17]. It is still unclear whether this association reflects a shared autoimmune pathogenesis or the presence of common etiological factors contributing to both conditions [17]. Notably, detectable thyroid antibodies can be present years before the clinical manifestation of thyroid dysfunction, which may delay AITD diagnosis until hypothyroidism becomes evident [21]. Many studies have reported a significantly higher prevalence of thyroid autoantibodies in women with PCOS [17]. However, to our knowledge, limited research has focused on assessing thyroid-stimulating hormone (TSH) levels and ensuring that case and control groups are appropriately matched. This systematic review and meta-analysis aimed to determine the prevalence and titers of thyroid autoantibodies in women with PCOS compared to control groups, with special attention to results stratified by matched TSH levels and body mass index (BMI).

## 2. Materials and Methods

### 2.1. Registration and Search Strategy

This review was conducted in accordance with the Preferred Reporting Items for Systematic Reviews and Meta-Analyses (PRISMA) guidelines and has been prospectively registered in the PROSPERO database (registration number: CRD42024622202). The protocol is available online https://www.crd.york.ac.uk/PROSPERO/view/CRD42024622202 (accessed on 3 August 2025). This systematic review is part of a broader investigation into the role of autoantibodies in women with PCOS. The current report focuses specifically on studies assessing thyroid autoantibodies, including anti-TPO and anti-TG antibodies.

A systematic search was conducted in PubMed, Scopus, and Embase on 1 April 2025, without restrictions on language or publication date. Two investigators (J.K. and N.A.) independently performed the literature search. The search strategy was built around two core search terms: “autoantibodies” and “polycystic ovary syndrome”, combined using the Boolean operator AND. Each term was expanded using relevant synonyms and MeSH headings where appropriate. Studies evaluating thyroid autoantibodies—either exclusively or alongside other autoantibodies—were identified during the screening and selection process. EndNote, version 21, Clarivate Analytics, Philadelphia, PA, USA was used for reference management and organization during the literature search and data extraction process. The full search strategy for all databases is provided in the Supplementary Materials (Supplementary Material S1).

### 2.2. Eligibility Criteria and Study Selection

We included observational (case-control, cross-sectional, and cohort) studies in the English language investigating either the prevalence or serum levels of anti-TPO and/or anti-TG antibodies in reproductive-age women with PCOS and without PCOS. Our research included circulating autoantibody levels in plasma measured with well-established methods, such as enzyme-linked immunosorbent assay (ELISA), chemiluminescent immunoassay (CLIA), or electrochemiluminescence immunoassay (ECLIA). Studies on pediatric patients (<18 years), pregnant women, or women undergoing menopause were excluded. Pregnant and menopausal women were excluded due to the profound hormonal and immunological changes occurring during these life stages, which could act as major confounding factors affecting thyroid autoantibody levels and their interpretation in the context of PCOS-related autoimmunity. Book chapters, case reports, commentaries, conference abstracts, editorials, errata, guidelines (also, statements, consensuses, position papers), letters to editors, notes, protocols, and reviews (systematic reviews, narrative reviews, meta-analyses) were excluded.

Titles and abstracts were independently screened by two reviewers (J.K. and N.A.) Subsequently, the full text of every potentially relevant article was retrieved for evaluation. The inclusion and exclusion criteria were applied to the full texts to determine final eligibility. Discrepancies were resolved by a third reviewer (L.Z.).

### 2.3. Data Extraction

Data extraction was performed independently by two reviewers (J.K. and N.A.) using a data extraction form. The following information was collected:

- Study characteristics (first author, publication year, type of work, country of origin);
- Participants’ characteristics (total number of participants, number of PCOS and control women, diagnostic criteria used for PCOS diagnosis, age, BMI, and whether matching for TSH, age, and/or BMI was performed);
- Autoantibody details (types of autoantibodies analyzed, measurement method);
- Outcome details (prevalence of autoantibody positivity and/or serum levels of anti-TPO and anti-TG in PCOS women versus controls, along with reported associations with clinical or biochemical parameters).

### 2.4. Risk of Bias Assessment and Statistical Analysis

- Study quality was assessed independently by two investigators (J.K. and N.A.) using a modified version of the Newcastle–Ottawa Scale (NOS) adapted for observational studies (Supplementary Material S2). Stars (points) were assigned in three main categories: Selection, Comparability, and Outcome. In the Selection domain, points were awarded for the use of an internationally recognized PCOS definition, clearly defined and appropriate inclusion/exclusion criteria, proper selection and description of a control group, and the use and reporting of a validated method for autoantibody measurement (maximum 4 points).
- In the Comparability domain, one point was given for age matching between groups, and an additional point was awarded if either BMI or TSH levels were matched between the PCOS and control groups (maximum 2 points).
- In the Outcome domain, studies were evaluated based on the clarity of outcome presentation, the consideration of potential confounders influencing autoantibody levels or prevalence (particularly history of thyroid disease or euthyroid status), and the application and reporting of appropriate statistical methods (maximum 3 points).

The tool was modified to better reflect methodological aspects relevant to studies on autoantibodies in PCOS. The use of standardized diagnostic criteria, along with clearly defined inclusion and exclusion criteria, ensures that study populations include women with well-characterized and consistently defined PCOS. In turn, validated laboratory methods and controlling for clinical confounding factors—such as TSH levels, BMI, and thyroid status—are essential for ensuring the reliability of autoantibody measurements and the validity of their interpretation. These elements are not specifically addressed in the original NOS but represent key quality indicators in this field of research.

Studies that received 7–9 points were considered low risk of bias, 4–6 as intermediate, and 0–3 as high risk, in accordance with previous NOS-based reviews [25,26].

Meta-analyses were conducted using Review Manager (RevMan), version 5.4.1, The Cochrane Collaboration, London, UK. Forest plots were used to present individual study data and pooled effect estimates. Studies were ordered within the plots according to the effect size. For dichotomous outcomes (prevalence of antibody positivity), odds ratios (ORs) with 95% confidence intervals (CIs) were calculated. For continuous outcomes (serum antibody levels), standardized mean differences (SMDs) with 95% CIs were used. Although all studies reported results in the same unit (IU/mL), different measurement methods were used across the studies; therefore, SMD was chosen to account for potential variability in the measurement scales. The inverse variance method was applied under a random effects model. Between-study heterogeneity was assessed using the I^2^ statistic, with between-study variance (τ^2^) estimated using the DerSimonian and Laird method. A subgroup analysis was performed to assess the potential impact of matching for BMI and TSH level between the PCOS and control groups. All statistical tests were two-sided, with *p*-values < 0.05 considered statistically significant.

In cases where the authors provided only percentages of the total without the number of participants, these were calculated manually based on the reported percentages and the sample size. For studies that did not report continuous variables as means with standard deviations (SDs), these were calculated in RevMan based on the reported means and standard errors of the mean (SEM). When studies reported medians with interquartile ranges (IQRs), it was assumed that the median approximated the mean, and the standard deviation was estimated by dividing the interquartile range (IQR) by 1.35. Similarly, when only the median along with minimum and maximum values were reported, it was assumed that the median approximated the mean, and the standard deviation was estimated by dividing the range (max–min) by 4.

Sensitivity analyses were performed for all main outcomes as well as for subgroup analyses. A leave-one-out approach was applied, in which each study was sequentially excluded from the meta-analysis. Additional sensitivity analyses were performed by restricting the dataset to studies assessed as having a low risk of bias, determined by the modified Newcastle–Ottawa Scale (NOS) scores. Moreover, a further sensitivity analysis excluded the studies in which mean and standard deviation values were imputed from medians, interquartile ranges, or ranges using standard conversion formulas. Potential publication bias was assessed using funnel plots, which were visually inspected for each outcome. In a separate sensitivity analysis, the studies that substantially deviated from the overall pattern in the funnel plot were excluded. Egger’s regression test was additionally applied as a formal statistical method to detect small-study effects and assess the likelihood of publication bias. This analysis was performed using R statistical software, version 4.5.1, R Foundation for Statistical Computing, Vienna, Austria.

## 3. Results

### 3.1. Study Selection Process

**Figure 1:**
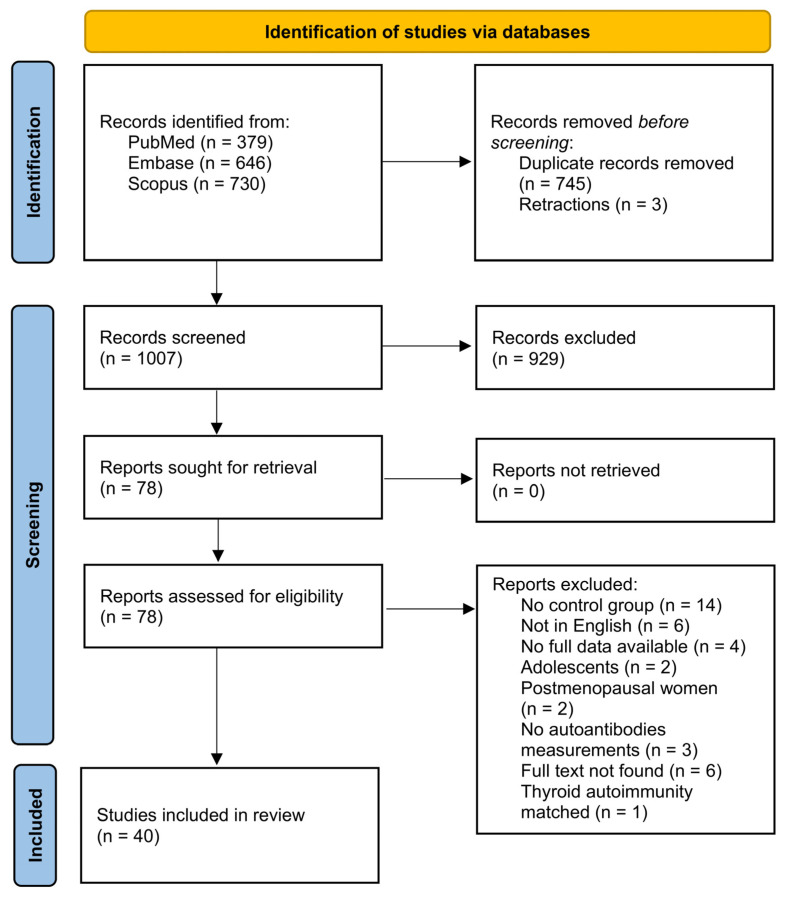
Preferred Reporting Items for Systematic Reviews and Meta-Analyses (PRISMA) flow diagram of the study selection process.

The initial literature search yielded 1755 results. After the removal of 745 duplicates and 3 retracted articles, 1007 records remained. Of these, 929 were excluded based on article type as well as title and abstract screening. As a result, 78 studies were selected for full-text review. Of these, 38 were excluded due to the following reasons: 14 due to the absence of a control group; 6 due to the lack of an English-language version; 4 due to incomplete data; 2 due to the inclusion of adolescents in the study population; 2 due to the inclusion of postmenopausal women in the study group; 3 due to the absence of autoantibody measurements; 6 due to the unavailability of the full text; and 1 due to the case and control groups having been pre-matched for thyroid autoimmunity at the study design stage. Ultimately, 40 studies were included in the systematic review and meta-analysis (Figure 1).

### 3.2. Summary of Included Studies Characteristics

A total of 40 studies were ultimately included in the systematic review and meta-analysis. A summary of the included studies is presented in Table 1. The studies originated from various countries around the world. The analysis comprised 9 studies from Europe (1 from Poland, 1 from Romania, 1 from the Netherlands, 1 from Italy, 1 from Germany, 1 from Bulgaria, 1 from Austria, 1 from Slovakia, and 1 from Greece), 27 from Asia (7 from India, 2 from Iran, 6 from Iraq, 1 from South Korea, 2 from China, 1 from Vietnam, 1 from Syria, and 7 from Turkey), 3 from Africa (2 from Sudan and 1 from Egypt), and 1 from South America (Brazil).The types of studies included 11 cross-sectional studies, 24 case-control studies, 2 studies combining cross-sectional and case-control designs, and 3 retrospective cohort studies. Study types were primarily reported by the authors. However, for those studies where the design was not explicitly stated, we independently assigned the study type based on the available methodological details.

The most commonly used diagnostic criteria across the studies were the Rotterdam 2003 criteria [8], applied in 33 studies. In addition, the National Institutes of Health (NIH) criteria [27] were used in two studies, one study followed the Chinese Ministry of Health standard [28], and three studies did not specify the diagnosis details [29,30,31]. One study applied the authors’ own criteria, which are not widely recognized or standardized [32].

Among the 40 included studies, control groups most frequently included women matched for age (27 studies) and TSH levels (25 studies), with BMI-matching applied in 9 studies. A total of 10 studies explicitly excluded participants with any history of thyroid disease and 6 studies confirmed euthyroid status. The terminology used to describe control participants varied across studies—they were most commonly referred to as “healthy women” or “women without PCOS”. In addition, different sets of chronic conditions were excluded depending on the study.

The size of the study populations ranged from 25 to 1429 participants in the PCOS groups and from 18 to 904 participants in the control groups. In total, data from 6045 individuals with PCOS and 4527 controls were included. The mean age of participants with PCOS ranged from 22 to 33 years, compared to 20.5 to 33.4 years in the control groups. The mean BMI ranged from 20.4 to 34.8 kg/m^2^ in the PCOS groups and from 20.1 to 32.3 kg/m^2^ in the comparison groups.

Across all the included studies, the antibody detection methods reported by the authors were grouped into major methodological categories. Among the 40 included publications, the most frequently used method for antibody detection was chemiluminescent immunoassay (CLIA), applied in 11 studies. This was followed by ELISA (nine studies) and electrochemiluminescence immunoassay (ECLIA) (seven studies). The remaining methods used included competitive chemiluminescent immunoassay (three studies), immunometric assay (two studies), enzyme immunoassay (EIA) (one study), fluorescence immunoassay (FIA) (one study), radioimmunoassay (RIA) (one study), immunoradiometric assay (IRMA) (one study), and luminescence immunoassay (LIA) (one study). In four cases, the detection method was not specified or was reported in insufficient detail to allow categorization.

**Table 1:** Characteristics of the studies included in the systematic review and meta-analysis investigating the prevalence and levels of thyroid autoantibodies in women with polycystic ovary syndrome.

Author, Year	Country	Type of Study	Control Group	PCOS Criteria	PCOS			Control			Antibody Measurement Method
					*N*	Age	BMI	*N*	Age	BMI	
Studies assessing only anti-TG antibodies (*n* = 1)											
Kamil et al., 2020 [31]	Sudan	cross-sectional	Age-matched healthy women (no history of thyroid disease)	not specified	50	30.18 ± 3.78	ND	50	28.88 ± 3.51	ND	ELISA
Studies assessing only anti-TPO antibodies (*n* = 16)											
Adamska et al., 2020 [33]	Poland	cross-sectional	Age-, TSH-matched women without PCOS	2003 Rotterdam criteria	141	ND	ND	88	25 (22.5; 27)	22 (20.7; 24.1)	RIA
Arora et al., 2016 [34]	India	case-control	Age-, TSH-matched healthy women	2003 Rotterdam criteria	55	23.27 ± 5.83	ND	51	22.8 ± 4.4	ND	ELISA
Dhoriyani et al., 2024 [35]	India	cross-sectional	Age-, TSH-matched healthy women (no history of thyroid disease)	2003 Rotterdam criteria	60	25.6 ± 3.7	28.95 ± 6.15	20	24 ± 4.2	22.5 ± 2.1	ECLIA
Gudovan et al., 2008 [36]	Romania	case-control	Age-matched women without PCOS	2003 Rotterdam criteria	25	25.1 ± 2.3	32.7 ± 2.9	20	25.4 ± 2.1	24.5 ± 2.1	immunometric assay
Heidarpour et al., 2023 [32]	Iran	cross-sectional	Healthy women	other	76	26.62 ± 5.58	ND	66	ND	ND	ELISA
Jameel et al., 2022 [30]	Iraq	cross-sectional	Age-, TSH-matched euthyroid healthy women	not specified	35	25.37 ± 4.5	29.91 ± 5.1	30	27.1 ± 3.9	26.3 ± 4.7	ECLIA
Kim et al., 2022 [37]	South Korea	case-control	Age-, TSH-, BMI-matched women without PCOS (no history of thyroid disease)	2003 Rotterdam criteria	210	33.3 ± 5.3	20.4 ± 2.8	343	33.4 ± 4.9	20.1 ± 2.3	IRMA
Menon et al., 2017 [38]	India	case-control	Age-, TSH-matched women without PCOS	2003 Rotterdam criteria	90	30.02 ± 8.51	24.6 ± 4	90	31.4 ± 8.6	21.3 ± 2.8	CLIA
Naruka et al., 2023 [39]	India	case-control	Age-, TSH-matched healthy women (no history of thyroid disease)	2003 Rotterdam criteria	75	30.13 ± 5.32	ND	75	28.54 ± 6.78	ND	CLIA
Shafi et al., 2024 [40]	India	cross-sectional	Age-, BMI-matched healthy women	2003 Rotterdam criteria	90	25.7 ± 4.16	25.3 ± 2.53	90	26.8 ± 5.13	24.9 ± 1.64	ND
Sinha et al., 2013 [41]	India	cross-sectional, case-control	BMI-matched women without PCOS	2003 Rotterdam criteria	80	22.7 ± 5.3	24.68 ± 3.07	80	24.3 ± 5.69	23.55 ± 3.02	EIA
Tran et al., 2023 [42]	Vietnam	retrospective cohort study	Age-, TSH-matched women without PCOS	2003 Rotterdam criteria	904	29 ± 3.58	22.8 ± 3.84	904	29.1 ± 3.4	19.9 ± 2.23	competitive CLIA
Van der Ham et al., 2023 [43]	Netherlands	cross-sectional	TSH-matched women without PCOS	2003 Rotterdam criteria	1429	28.2 (24.6; 31.7)	25.5 (21.9; 30.7)	299	32.8 (29.7; 35.8)	24.4 (22.3; 27.5)	FIA
Wang et al., 2018 [28]	China	retrospective cohort study	Tubal obstruction infertile patients with no other chronic diseases (no history of thyroid disease, negative for anti-TG and anti-TM)	Chinese Ministry of Health (2011) Industry Standard	124	ND	ND	108	ND	ND	ND
Yasar et al., 2022 [44]	Turkey	cross-sectional, case-control	Age-, TSH-, BMI-matched healthy women (no history of thyroid disease)	2003 Rotterdam criteria	180	25.94 ± 6.18	29.77 ± 6.65	100	28.12 ± 7.27	28.04 ± 6.16	CLIA
Yu et al., 2016 [45]	China	case-control	Age-, TSH-matched euthyroid healthy women	2003 Rotterdam criteria	68	26.37 ± 4.5	30.91 ± 5.1	91	28.1 ± 3.9	27.3 ± 4.7	ECLIA
Studies assessing both anti-TG and anti-TPO antibodies (*n* = 23)											
Al-Karawi et al., 2024 [29]	Iraq	case-control	Healthy women	not specified	150	ND	ND	150	ND	ND	LIA
Al-Saab et al., 2014 [46]	Syria	case-control	BMI-, TSH-matched euthyroid women without PCOS	2003 Rotterdam criteria	56	23.8 ± 5.6	24.9 ± 5.9	30	28.9 ± 5.8	23.9 ± 2.9	ECLIA
Anaforoglu et al., 2011 [47]	Turkey	case-control	Age-, TSH-matched euthyroid healthy women	2003 Rotterdam criteria	84	23.7 ± 6.8	30.1 ± 8.3	81	24.5 ± 7.1	27.2 ± 6.9	CLIA
Anusha et al., 2024 [48]	India	cross-sectional	Age-matched euthyroid infertile women without PCOS	2003 Rotterdam criteria	66	28.73 ± 3.28	26.52 ± 4.6	66	29.27 ± 3.97	24.31 ± 3.1	ELISA
Arduc et al., 2015 [49]	Turkey	cross-sectional	Age-, BMI-matched healthy women (no history of thyroid disease)	2003 Rotterdam criteria	86	24.6 ± 5.7	24.9 ± 3.6	60	26.17 ± 5	23.4 ± 2.9	ECLIA
Auda et al., 2023 [50]	Iraq	case-control	Age-matched women without PCOS	2003 Rotterdam criteria	85	27.2 ± 6.4433	27.4575 ± 1.70508	50	26.7 ± 4.4272	23.0571 ± 1.9972	ELISA
De Souza Mayrink Novais et al., 2015 [51]	Brazil	cross-sectional	Women without PCOS (no history of thyroid disease)	2003 Rotterdam criteria	65	27.8 ± 6.9	34.8 ± 8.9	65	33.5 ± 5.7	28.4 ± 4.8	ECLIA
Duran et al., 2015 [52]	Turkey	case-control	Age-, TSH-matched women without PCOS	2003 Rotterdam criteria	73	22 [18; 37]	27.45 ± 5.73	60	20.5 [19; 35]	22.55 ± 3.78	CLIA
El-Din et al., 2015 [53]	Egypt	case-control	TSH-matched euthyroid women without PCOS	2003 Rotterdam criteria	45	22.24 ± 4.7	25.9 ± 2.5	18	27.89 ± 5.4	24.2 ± 2	ELISA
Garelli et al., 2013 [54]	Italy	case-control	Healthy women (no history of thyroid disease)	2003 Rotterdam criteria	113	24 ± 6.3	ND	100	27.1 ± 1.2	ND	CLIA
Hepşen et al., 2019 [55]	Turkey	case-control	Age-, TSH-matched healthy women (no history of thyroid disease)	2003 Rotterdam criteria	184	23.9 ± 5.6	26.4 [16; 44]	106	24.3 ± 4.3	22.1 [16; 40.4]	competitive CLIA
Janssen et al., 2004 [56]	Germany	case-control	Age-matched women without PCOS	NIH	175	28.4 ± 6.5	30 ± 7.9	168	29.8 ± 7.4	25.5 ± 7.1	immunometric assay
Kachuei et al., 2012 [57]	Iran	case-control	Age-, TSH-matched women without PCOS	2003 Rotterdam criteria	78	23.95 ± 5.2	ND	350	24.38 ± 3.17	ND	ELISA
Karaköse et al., 2013 [58]	Turkey	cross-sectional	Age-, TSH-, BMI-matched healthy women (no history of thyroid disease)	2003 Rotterdam criteria	52	24.4 ± 10.5	26.5 ± 6.1	68	26.5 ± 6.5	24.8 ± 4.9	ELISA
Karaköse et al., 2017 [59]	Turkey	case-control	Age-, TSH-matched healthy women with no chronic disease	2003 Rotterdam criteria	97	24.1 ± 6	27.5 ± 6	71	24.4 ± 4.5	23.4 ± 5	competitive CLIA
Malik Aubead et al., 2020 [60]	Iraq	case-control	Age-matched women without PCOS	2003 Rotterdam criteria	35	27.2 ± 6.7	29.4 ± 3.1	35	28.3 ± 7.2	27.7 ± 3.6	ND
Mitkov et al., 2015 [61]	Bulgaria	case-control	Age-, BMI-, TSH-matched euthyroid healthy women	2003 Rotterdam criteria	70	25.06 {0.69}	26.5 {0.83}	22	26.78 {1.7}	23.36 {1.4}	CLIA
Mohammed et al., 2017 [62]	Sudan	case-control	Age-, TSH-matched infertile women without PCOS	2003 Rotterdam criteria	55	27.3 ± 5.2	28.2 ± 5.3	55	27.1 ± 4.8	25.3 ± 5.8	ELISA
Ott et al., 2010 [63]	Austria	retrospective cohort study	Women without PCOS	2003 Rotterdam criteria	196	27.1 ± 5.1	25.5 ± 3.3	100	27.8 ± 6.7	27.3 ± 5.7	ND
Petrikova et al., 2015 [64]	Slovak Republic	case-control	Age-, TSH-matched healthy women (no history of autoimmune disease)	2003 Rotterdam criteria	64	31 ± 4.5	28.08 ± 6.91	68	29 ± 3.9	21.31 ± 3.05	CLIA
Saleh et al., 2021 [65]	Iraq	case-control	Healthy women	2003 Rotterdam criteria	50	[18; 39]	ND	25	ND	ND	CLIA
Vryonidou et al., 2025 [66]	Greece	case-control	Age-, BMI-, TSH-matched women without PCOS (no history of thyroid disease)	NIH	257	24.56 ± 5.98	27.96 ± 7.15	143	25.17 ± 5.97	26.58 ± 7.34	ECLIA
Yasar et al., 2016 [67]	Iraq	case-control	Age-, BMI-, TSH-matched overweight or obese healthy women (no history of thyroid disease)	2003 Rotterdam criteria	122	25.65 ± 5.89	33.51 ± 5.16	65	27.1 ± 7.53	32.31 ± 5.31	CLIA
			Age-, BMI-, TSH-matched healthy women with normal BMI (no history of thyroid disease)	2003 Rotterdam criteria	95	23.98 ± 6.11	21.96 ± 1.7	66	25.15 ± 7.03	21.47 ± 1.97	CLIA

Numerical values are given as mean ± standard deviation, median (IQR), mean [min; max], mean {standard error of the mean}, unless otherwise specified. Anti-TG—anti-thyroglobulin antibodies; anti-TPO—anti-thyroid peroxidase antibodies; BMI—body mass index; CLIA—chemiluminescent immunoassay; ECLIA—electrochemiluminescence immunoassay; EIA—enzyme immunoassay; ELISA—enzyme-linked immunosorbent assay; FIA—fluorescence immunoassay; IRMA—immunoradiometric assay; LIA—luminescence immunoassay; ND—no data; *N*—number of participants; *n*—number of studies; NIH—National Institutes of Health; PCOS—polycystic ovary syndrome; RIA—radioimmunoassay; TSH—thyroid-stimulating hormone.

Among the 40 studies included in this review, one study assessed only anti-TG antibodies, 16 studies focused exclusively on anti-TPO antibodies, while the remaining 23 studies evaluated both autoantibodies.

Across all studies, anti-TPO antibody prevalence was assessed in 23 papers (Table 2), while serum levels were evaluated in 27 (Table 3). For anti-TG antibodies, prevalence was measured in 14 studies (Table 2) and levels were reported in 19 (Table 3).

Regarding the comparisons between women with PCOS and the controls:

· Anti-TPO antibody prevalence was found to be significantly higher in the women with PCOS in 13 studies, while 10 studies reported no significant difference.
· Anti-TPO levels were significantly elevated in the women with PCOS in 16 studies, with 11 studies observing no significant difference.
· Anti-TG antibody prevalence was significantly higher in the women with PCOS in five studies, whereas nine found no significant difference.
· Anti-TG levels were significantly increased in 10 studies, while 9 reported comparable levels between the PCOS and control groups.

One study reporting significantly elevated levels of anti-TG antibodies did not provide full results [34]; therefore, this outcome was not included in the meta-analysis. Additionally, two studies reported only the combined prevalence of thyroid antibodies without providing separate results for anti-TPO and anti-TG [56,61]. As a result, these findings were also not included in the meta-analysis. One of them reported a higher prevalence in women with PCOS [56], while the other found no significant difference between groups [61].

Only 8 out of the 40 studies reported significant correlations between thyroid autoantibody levels or prevalence and various clinical, hormonal, or metabolic parameters (Table 2 and Table 3). Among the associated factors identified for anti-TG antibodies, positive correlations were found with BMI, metabolic syndrome, postprandial glucose, low-density lipoprotein (LDL) cholesterol, triglycerides, and TSH, while age was negatively correlated. For anti-TPO antibodies, positive correlations were reported with estradiol, a BMI ≥ 25, LH, FSH, presence of PCOM, TSH, metabolic syndrome, age, body weight, waist circumference, diastolic blood pressure, and postprandial glucose. Negative correlations were observed with prolactin (PRL), thyroxine (T4), high-density lipoprotein (HDL) cholesterol, testosterone, and dehydroepiandrosterone sulfate (DHEAS).

The methodological quality assessment using the modified Newcastle–Ottawa Scale (NOS) is presented in Table 4. A total of 30 studies were categorized as having a low risk of bias (7–9 points), 9 as having an intermediate risk (4–6 points), and 1 study as having a high risk of bias (0–3 points).

### 3.3. Main Findings

#### 3.3.1. Anti-TPO Prevalence

**Figure 2:**
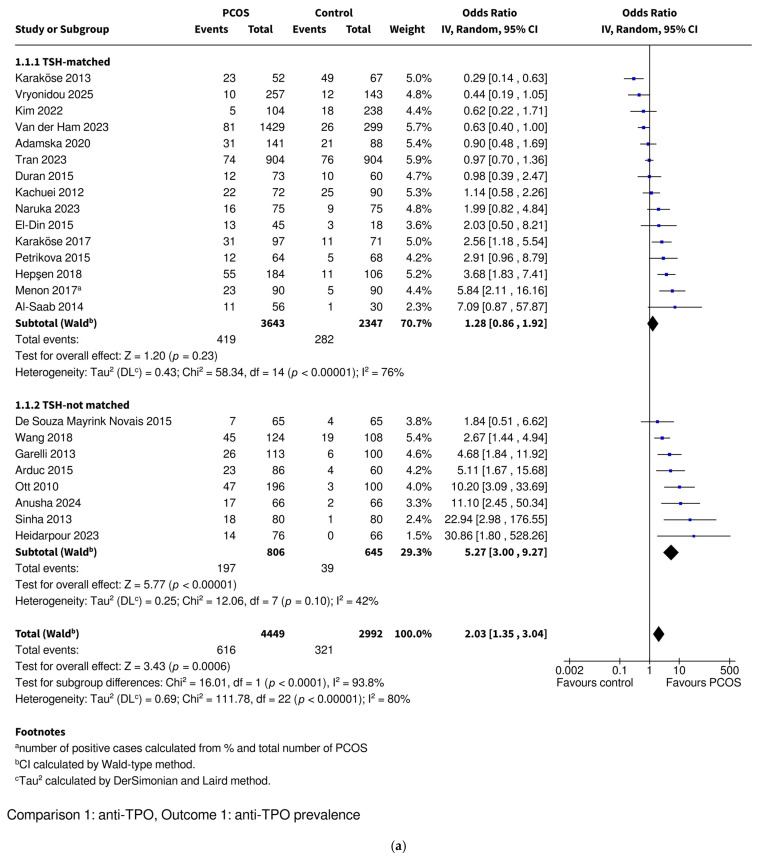

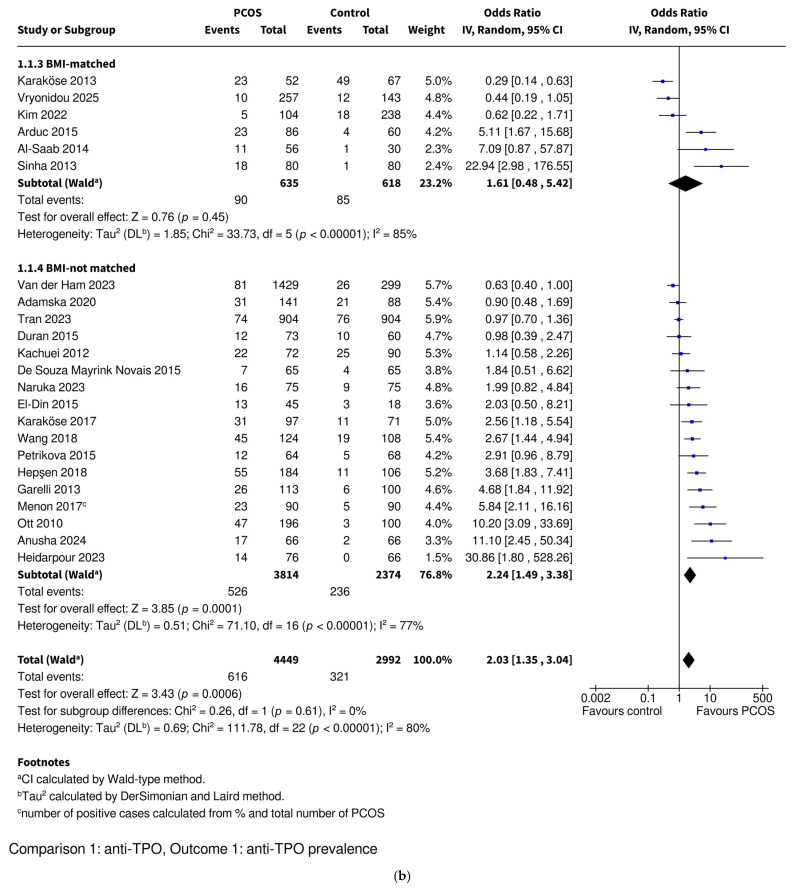
Forest plot of observational studies investigating the association between the prevalence of anti-TPO autoantibodies and polycystic ovary syndrome. The studies were subcategorized into (**a**) TSH-matched and TSH-not matched groups and (**b**) BMI-matched and BMI-not matched groups. The TSH-not matched subgroup includes studies where TSH levels were significantly higher in the PCOS group or where no data on TSH levels was available. The BMI-not matched subgroup includes studies where BMIs were significantly higher in the PCOS group or where no data on BMI was available. Anti-TPO—anti-thyroid peroxidase antibodies; BMI—body mass index; CI—confidence interval; IV—inverse variance; OR—odds ratio; PCOS—polycystic ovary syndrome. References: [28,32,33,37,38,39,41,42,43,46,48,49,51,52,53,54,55,57,58,59,63,64,66].

Women with PCOS were found to be at an increased risk for anti-TPO positivity based on 23 study reports including 4449 PCOS and 2992 control women (OR 2.03, 95% CI: [1.35–3.04], *p* = 0.0006) (Figure 2). Studies were divided into TSH-matched and TSH-not matched groups (Figure 2a) and then into BMI-matched and BMI-not matched groups (Figure 2b). The TSH-not matched and BMI-not matched subgroups included studies where no data on these parameters was available or their values were statistically significantly higher in the PCOS groups. None of the included studies reported statistically significantly lower TSH or BMI values in the PCOS group compared to the controls. The difference was no longer statistically significant in the matched subgroups, while it remained significant in the non-matched subgroups. The women with PCOS who were TSH-matched with the controls had a similar prevalence of anti-TPO positivity (OR 1.28, 95% CI: [0.86–1.92], *p* = 0.23) in contrast to the TSH-not matched women (OR 5.27, 95% CI: [3.00–9.27], *p* < 0.00001). The women with PCOS who were BMI-matched with the controls also had similarly prevalent anti-TPO positivity (OR 1.61, 95% CI: [0.48–5.42], *p* = 0.45), while the BMI-not matched PCOS women had higher anti-TPO positivity prevalence (OR 2.24, 95% CI: [1.49–3.38], *p* = 0.0001). The test for subgroup differences revealed that TSH-matching had a significant impact on decreases in the OR (*p* < 0.0001), while BMI-matching did not reach statistical significance (*p* = 0.61).

Overall heterogeneity was high (I^2^ = 80%), while subgrouping the studies into TSH-matched and TSH-not matched categories decreased it (TSH-matched subgroup I^2^ = 76%; TSH-not matched subgroup I^2^ = 42%). BMI-matching did not decrease heterogeneity (BMI-matched subgroup I^2^ = 85%; BMI-not matched subgroup I^2^ = 77%). Sensitivity analyses were conducted by iteratively excluding each study (the leave-one-out method) and restricting the analysis to studies with a low risk of bias. Removal of any study did not change the statistical significance of the results, both in the overall analysis and the TSH-matching and BMI-matching subgroups (Supplementary Material S3A–W for TSH, Supplementary Material S3X–AT for BMI). Similarly, excluding the studies with intermediate and high risks of bias [28,32,54,63] did not change the significance of the results in the overall analysis or in either subgroup comparison (Supplementary Material S3AU for TSH, S3AV for BMI). A funnel plot was used to assess potential publication bias (Supplementary Material S3AW). It showed noticeable asymmetry, with a greater number of studies from the TSH-not matched subgroup positioned to the right of the pooled effect estimate. Egger’s regression test indicated significant funnel plot asymmetry (*p* = 0.0033), suggesting potential publication bias (Supplementary Material S7). A visual inspection of the funnel plot revealed two studies [32,41] with the greatest deviation from the expected distribution (one TSH-not matched and BMI-matched with a low risk of bias and one TSH-not matched and BMI-not matched with high risk of bias), possibly contributing to the observed asymmetry. However, the sensitivity analyses excluding these outlying studies did not affect the statistical significance of the pooled effect estimates in the overall analysis or in either subgroup comparison (Supplementary Material S3AX for TSH, S3AY for BMI).

#### 3.3.2. Anti-TPO Level

**Figure 3:**
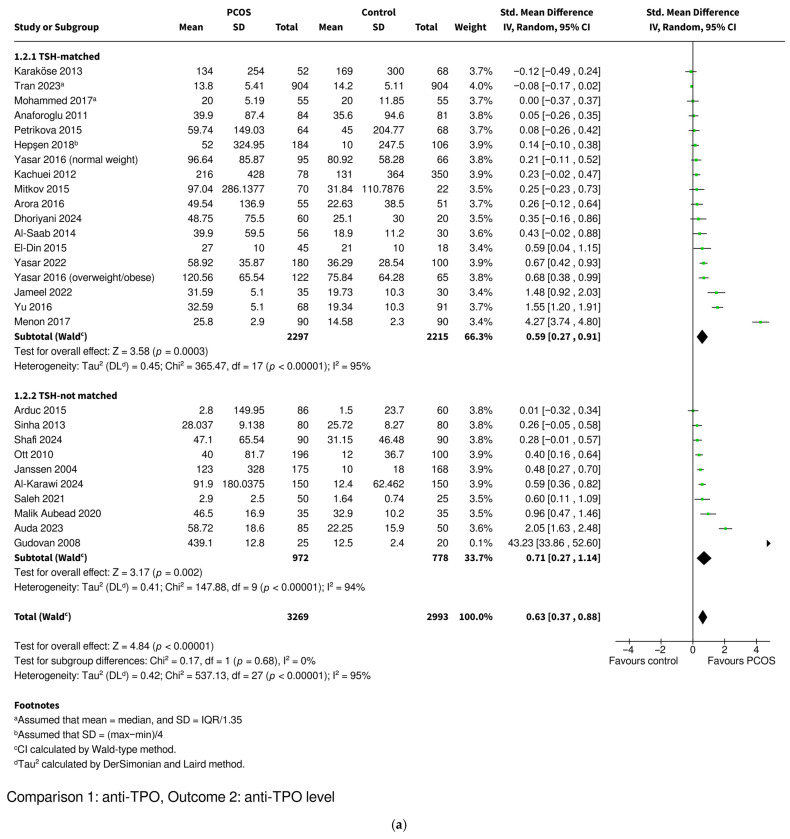

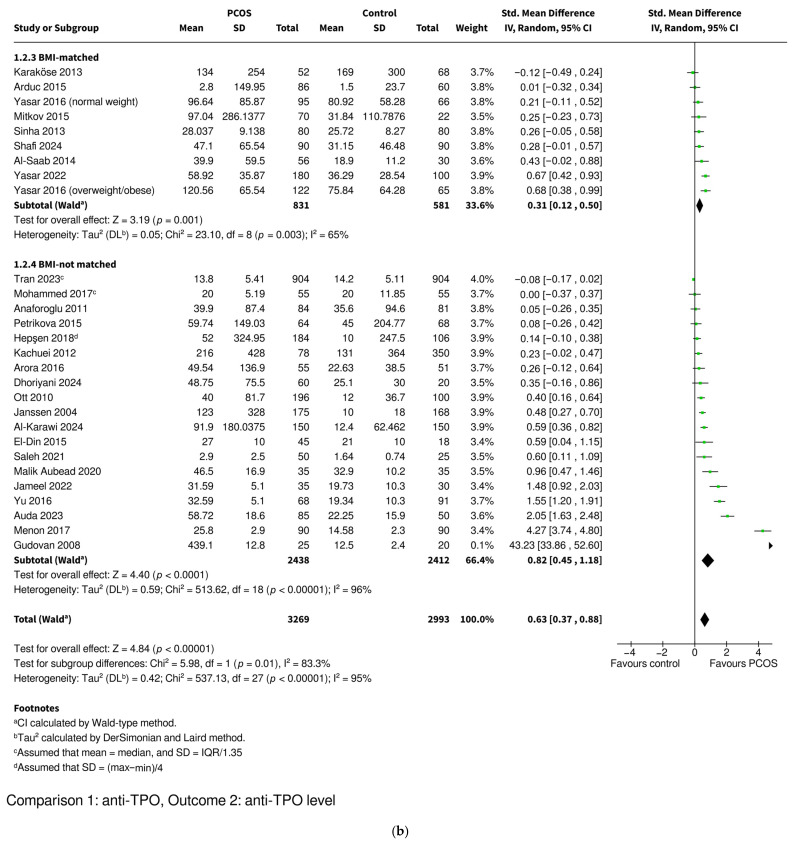
Forest plot of observational studies investigating the association between the level of anti-TPO autoantibodies and polycystic ovary syndrome. The studies were subcategorized into (**a**) TSH-matched and TSH-not matched groups and (**b**) BMI-matched and BMI-not matched groups. The TSH-not matched subgroup includes studies where TSH levels were significantly higher in the PCOS group or where no data on TSH levels was available. The BMI-not matched subgroup includes studies where BMIs were significantly higher in the PCOS group or where no data on BMI was available. Anti-TPO—anti-thyroid peroxidase antibodies; BMI—body mass index; CI—confidence interval; IV—inverse variance; PCOS—polycystic ovary syndrome; SD—standard deviation; Std. Mean Difference—standardized mean difference; TSH—thyroid-stimulating hormone. References: [29,30,34,35,36,38,40,41,42,44,45,46,47,49,50,53,55,56,57,58,60,61,62,63,64,65,67].

Women with PCOS were found to have significantly higher levels of anti-TPO compared to controls, based on 27 studies including 3269 women with PCOS and 2993 controls (SMD 0.63, 95% CI: [0.37–0.88], *p* < 0.00001) (Figure 3). The studies were divided into TSH-matched and TSH-not matched groups (Figure 3a) and then into BMI-matched and BMI-not matched groups (Figure 3b). The TSH-not matched and BMI-not matched subgroups included studies where no data on these parameters was available or their values were statistically significantly higher in the PCOS groups. None of the included studies reported statistically significantly lower TSH or BMI values in the PCOS groups compared to the controls. The difference remained statistically significant in the matched subgroups as well as in the unmatched ones. The women with PCOS who were TSH-matched with the controls had higher levels of anti-TPO (SMD 0.59, 95% CI: [0.27–0.91], *p* = 0.0003) as did the TSH-not matched women (SMD 0.71, 95% CI: [0.27–1.14], *p* = 0.002). The women with PCOS who were BMI-matched with the controls also had higher anti-TPO levels (SMD 0.31, 95% CI: [0.12–0.50], *p* = 0.001), although the difference was even more pronounced in the BMI-not matched women (SMD 0.82, 95% CI: [0.45–1.18], *p* < 0.0001). The test for subgroup differences showed that TSH-matching had no significant effect on the difference in anti-TPO levels between the PCOS and control groups (*p* = 0.68), while BMI-matching was associated with a significantly smaller difference (*p* = 0.01).

Overall heterogeneity was very high (I^2^ = 95%), while subgrouping studies into BMI-matched and BMI-not matched categories decreased it (BMI-matched subgroup I^2^ = 65%; BMI-not matched subgroup I^2^ = 96%). TSH-matching did not decrease heterogeneity (TSH-matched subgroup I^2^ = 95%; TSH-not matched subgroup I^2^ = 94%). Sensitivity analyses were conducted by iteratively excluding each study (the leave-one-out method), restricting the analysis to studies with a low risk of bias and excluding studies in which mean and standard deviation values were imputed from medians, interquartile ranges, or ranges. The removal of any individual study did not affect the statistical significance of the results in the overall analysis or TSH-matching and BMI-matching subgroups; however, the exclusion of one study eliminated the statistically significant difference between the BMI-matched and BMI-not matched subgroups [38] (Supplementary material S4A–AB for TSH, Supplementary material S4AC–BD for BMI). Similarly, the exclusion of studies with an intermediate or high risk of bias [29,36,50,60,63,65] did not change the significance of the results in the overall analysis or in either subgroup comparison; however, it eliminated the statistically significant difference between the BMI-matched and BMI-not matched subgroups (Supplementary Material S4BE for TSH, S4BF for BMI). The exclusion of studies with imputed mean and standard deviation values [42,55,62] did not change the significance of results in the overall meta-analysis or in either subgroup comparison (Supplementary Material S4BG for TSH, S4BH for BMI). A funnel plot was used to assess potential publication bias (Supplementary Material S4BI). The distribution of studies showed asymmetry with one distinct outlier with substantial deviation from the expected pattern (TSH-not matched, BMI-not matched, intermediate risk of bias), characterized by an exceptionally large effect size compared to the remaining studies [36]. Egger’s regression test confirmed significant funnel plot asymmetry for anti-TPO level (*p* = 0.0003), indicating potential publication bias (Supplementary Material S7). After exclusion of this outlier, the funnel plot appeared less asymmetrical (Supplementary Material S4BJ). The exclusion of this outlier did not significantly alter the results (Supplementary Material S4AB for TSH, S4BD for BMI).

#### 3.3.3. Anti-TG Prevalence

**Figure 4:**
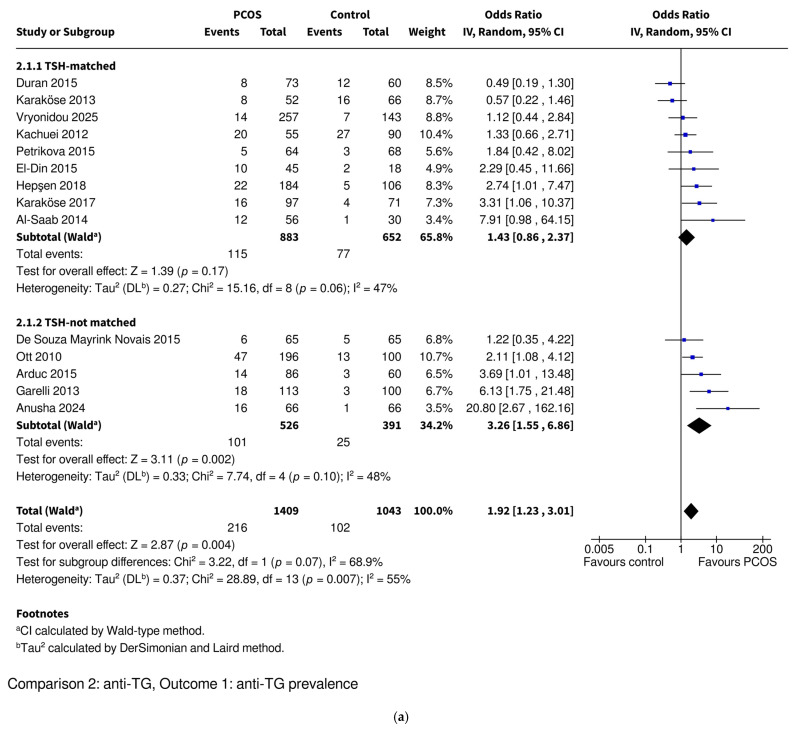

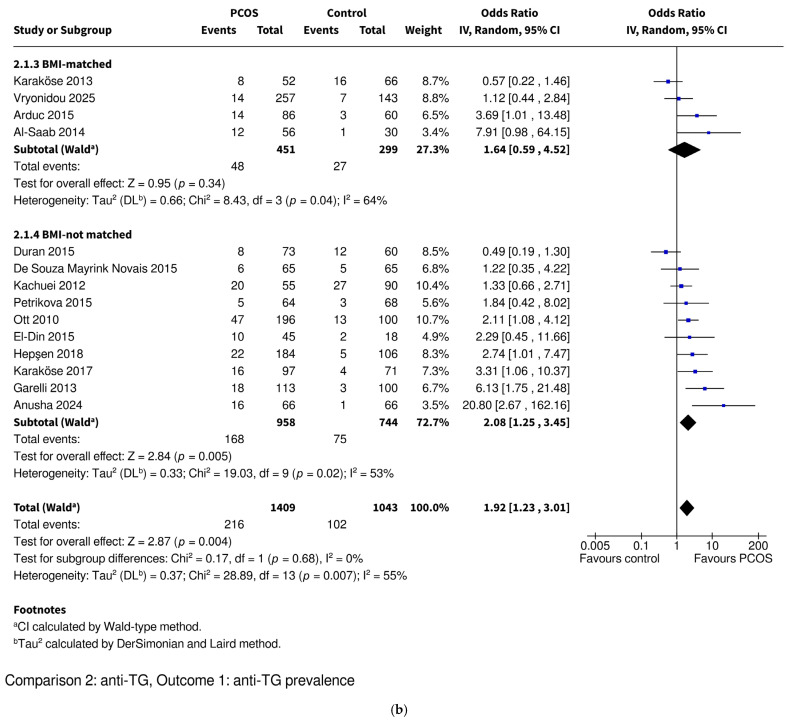
Forest plot of observational studies investigating the association between the prevalence of anti-TG autoantibodies and polycystic ovary syndrome. The studies were subcategorized into (**a**) TSH-matched and TSH-not matched groups and (**b**) BMI-matched and BMI-not matched groups. The TSH-not matched subgroup includes studies where TSH levels were significantly higher in the PCOS group or where no data on TSH levels was available. The BMI-not matched subgroup includes studies where BMIs were significantly higher in the PCOS group or where no data on BMI was available. Anti-TG—anti-thyroglobulin antibodies; BMI—body mass index; CI—confidence interval; IV—inverse variance; OR—odds ratio; PCOS—polycystic ovary syndrome; TSH—thyroid-stimulating hormone. References: [46,48,49,51,52,53,54,55,57,58,59,63,64,66].

Women with PCOS were found to be at an increased risk for anti-TG positivity based on 14 studies including 1409 PCOS and 1043 control women (OR 1.92, 95% CI: [1.23–3.01], *p* = 0.004) (Figure 4). Studies were divided into TSH-matched and TSH-not matched groups (Figure 4a) and then into BMI-matched and BMI-not matched groups (Figure 4b). The TSH-not matched and BMI-not matched subgroups included studies where no data on these parameters was available or their values were statistically significantly higher in the PCOS groups. None of the included studies reported statistically significantly lower TSH or BMI values in the PCOS groups compared to controls. The difference was no longer statistically significant in the matched subgroups, while it remained significant in the not matched subgroups. The women with PCOS who were TSH-matched with the controls had a similar prevalence of anti-TG positivity (OR 1.43, 95% CI: [0.86–2.37], *p* = 0.17) in contrast to the TSH-not matched women (OR 3.26, 95% CI: [1.55–6.86], *p* = 0.002). The women with PCOS who were BMI-matched with the controls also had similar anti-TG positivity prevalence (OR 1.64, 95% CI: [0.59–4.52], *p* = 0.34), while the BMI-not matched PCOS women had higher prevalences of anti-TG positivity (OR 2.08, 95% CI: [1.25–3.45], *p* = 0.005). The test for subgroup differences revealed that TSH-matching or BMI-matching had no significant effect on the difference in anti-TG positivity prevalence between the PCOS and control groups (TSH-matching *p* = 0.07; BMI-matching *p* = 0.68).

Overall heterogeneity was moderate (I^2^ = 55%), while subgrouping studies into TSH-matched and TSH-not matched categories decreased it (TSH-matched subgroup I^2^ = 47%; TSH-not matched subgroup I^2^ = 48%). BMI-matching did not decrease heterogeneity (BMI-matched subgroup I^2^ = 64%; BMI-not matched subgroup I^2^ = 53%). Sensitivity analyses were conducted by iteratively excluding each study (leave-one-out method) and by restricting the analysis to studies with a low risk of bias. These analyses had no impact on the statistical significance of the overall pooled effect. However, the removal of one study [52] caused the effect in the TSH-matched subgroup to shift just above the threshold of statistical significance, resulting in a significantly higher prevalence of anti-TG antibodies in the TSH-matched women with PCOS, similar to the TSH-not matched group. Additionally, the removal of any of three studies [46,51,59] resulted in a statistically significant difference in anti-TG prevalence between the TSH-matched and TSH-not matched subgroups, with higher prevalence observed in the TSH-not matched group (Supplementary Material S5A–N for TSH). In contrast, the removal of any individual study did not affect the statistical significance of the results in the BMI-matching subgroups (Supplementary Material S5O–AB for BMI). Excluding the studies with intermediate and high risks of bias [54,63] resulted in a loss of statistical significance in the TSH-not matched subgroup and in the BMI-not matched subgroup; the result became borderline significant (*p* = 0.05). Importantly, these changes did not influence the overall pooled effect (Supplementary Material S5AC for TSH, S5AD for BMI). A funnel plot was used to assess potential publication bias (Supplementary Material S5AE). Visual inspection revealed a relatively symmetrical distribution of studies, with only minimal asymmetry observed. Egger’s regression test did not reveal significant funnel plot asymmetry (*p* = 0.0705), suggesting no strong evidence of publication bias (Supplementary Material S7). Two studies [46,48], identified as the most divergent in terms of effect size (one TSH- and BMI-matched and the other TSH-not matched and BMI-not matched, both with a low risk of bias), were excluded from the sensitivity analyses. Their exclusion did not alter the statistical significance of the results within or between subgroups and importantly, the removal of these studies also had no effect on the overall pooled estimate (Supplementary Material S5AF for TSH, S5AG for BMI).

#### 3.3.4. Anti-TG Level

**Figure 5:**
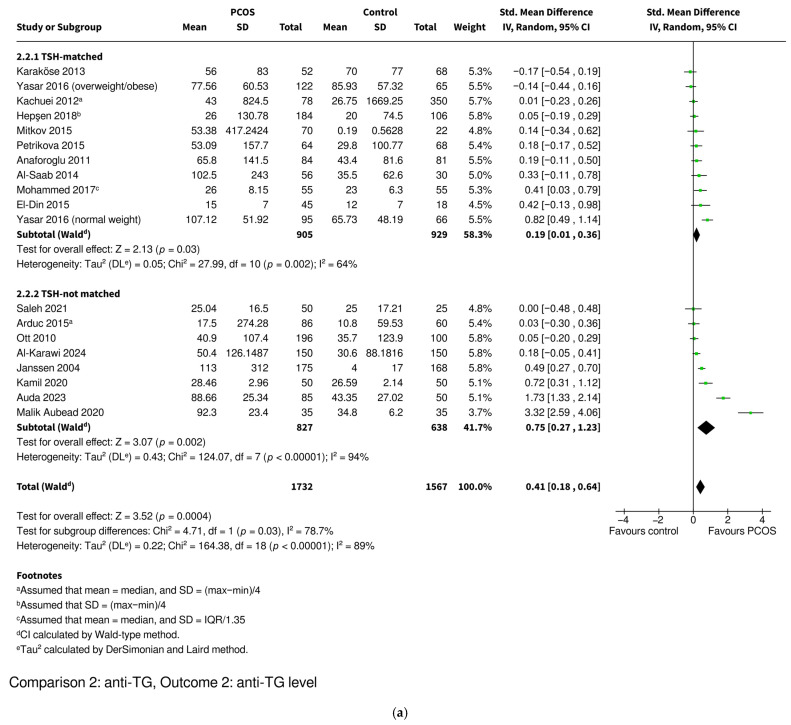

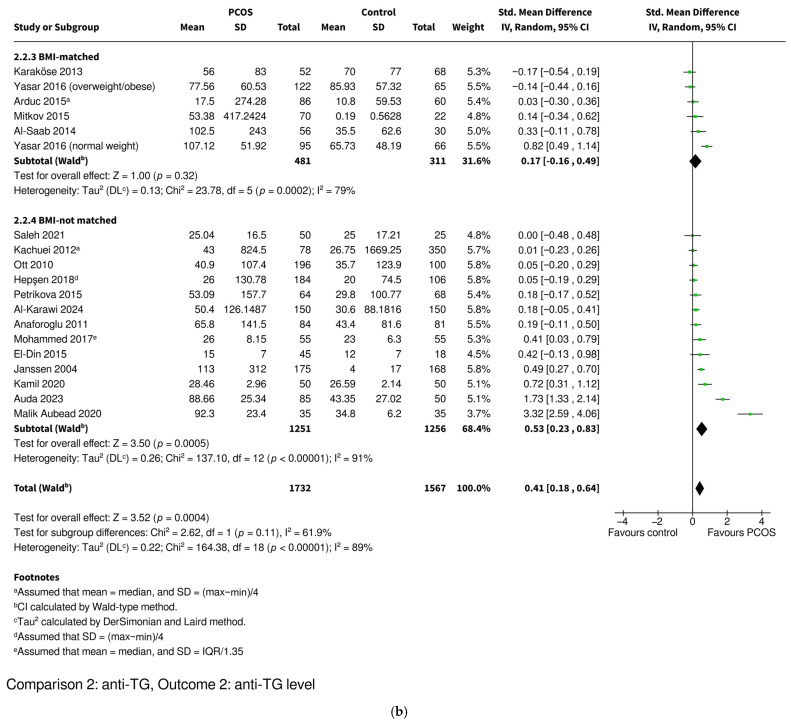
Forest plot of observational studies investigating the association between the level of anti-TG autoantibodies and polycystic ovary syndrome. The studies were subcategorized into (**a**) TSH-matched and TSH-not matched groups and (**b**) BMI-matched and BMI-not matched groups. The TSH-not matched subgroup includes studies where TSH levels were significantly higher in the PCOS group or where no data on TSH levels was available. The BMI-not matched subgroup includes studies where BMIs were significantly higher in the PCOS group or where no data on BMI was available. Anti-TG—anti-thyroglobulin antibodies; BMI—body mass index; CI—confidence interval; IV—inverse variance; PCOS—polycystic ovary syndrome; SD—standard deviation; Std. Mean Difference—standardized mean difference; TSH—thyroid-stimulating hormone. References: [29,31,46,47,49,50,53,55,56,57,58,60,61,62,63,64,65,67].

Women with PCOS were found to have higher anti-TG levels based on 18 studies including 1732 PCOS and 1567 control women (SMD 0.41, 95% CI: [0.18–0.64], *p* = 0.0004) (Figure 5). Studies were divided into TSH-matched and TSH-not matched groups (Figure 5a) and then into BMI-matched and BMI-not matched groups (Figure 5b). TSH-not matched and BMI-not matched subgroups included studies where no data on these parameters was available, or their values were statistically significantly higher in the PCOS groups. None of the included studies reported statistically significantly lower TSH or BMI values in the PCOS groups compared to the controls. The difference was no longer statistically significant in the BMI-matched subgroup, while it remained significant in the TSH-matched subgroup and in the not matched subgroups. The women with PCOS who were TSH-matched with the controls showed slightly higher anti-TG levels (SMD 0.19, 95% CI: [0.01–0.36], *p* = 0.03), whereas the TSH-not matched women exhibited markedly higher levels (SMD 0.75, 95% CI: [0.27–1.23], *p* = 0.002). In contrast, the women with PCOS who were BMI-matched with the controls had similar anti-TG levels (SMD 0.17, 95% CI: [−0.16–0.49], *p* = 0.32), while the BMI-not matched PCOS women had higher anti-TG levels (SMD 0.53, 95% CI: [0.23–0.83], *p* = 0.0005). The test for subgroup differences revealed that TSH-matching had a significant impact on reducing the SMD (*p* = 0.03), while BMI-matching did not reach statistical significance (*p* = 0.11).

Overall heterogeneity was very high (I^2^ = 89%), while subgrouping studies into TSH-matched and TSH-not matched categories decreased it (TSH-matched subgroup I^2^ = 64%; TSH-not matched subgroup I^2^ = 94%). BMI-matching also led to a modest reduction in heterogeneity (BMI-matched subgroup I^2^ = 79%; BMI-not matched subgroup I^2^ = 91%). Sensitivity analyses were conducted by iteratively excluding each study (the leave-one-out method), restricting the analysis to studies with a low risk of bias and excluding studies in which mean and standard deviation values were imputed from medians, interquartile ranges, or ranges. These analyses did not affect the statistical significance of the overall pooled effect. However, in the TSH-matched subgroup, the exclusion of any one of five studies led to the loss of statistical significance [46,47,53,62,64,67]. In one additional case [64], the *p*-value increased to 0.05, indicating borderline significance (Supplementary Material S6A–K for TSH-matched). However, these changes were minimal, as the original confidence interval already approached the threshold of significance (95% CI: [0.01–0.36]), suggesting that the effect was borderline from the outset rather than substantially altered by these studies. In contrast, the results for the TSH-not matched and BMI subgroups remained stable. However, the exclusion of a single study [67] caused the BMI-matched and unmatched subgroups to differ significantly (Supplementary Material S6T–AL for BMI). For the TSH subgroups, the exclusion of any one of two studies led to a loss of statistical significance between subgroups [50,60], while the exclusion of two others resulted in borderline significance (*p* = 0.05) [31,56] (Supplementary Material S6L–S for TSH-not matched). Excluding the studies with intermediate and high risks of bias [29,31,50,60,63,65] resulted in the loss of the significant effect in the TSH-not matched subgroup and the difference between the TSH-matched and TSH-not matched subgroups was no longer statistically significant (Supplementary Material S6AM for TSH, S6AN for BMI). Importantly, the overall pooled effect remained statistically significant. The exclusion of studies with imputed mean and standard deviation values [49,55,57,62] led to the loss of borderline statistical significance in the TSH-matched subgroup. However, the effect estimate remained similar (SMD 0.21, 95% CI: –0.03 to 0.46, *p* = 0.09). The overall effect, as well as the results for the TSH-not matched and BMI subgroups, remained stable (Supplementary Material S6AO for TSH, S6AP for BMI). A funnel plot was used to assess potential publication bias (Supplementary Material S6AR). Visual inspection revealed slight asymmetry in the distribution of studies around the pooled effect estimate, with no individual study showing substantial deviation from the expected pattern. Egger’s regression test indicated statistically significant funnel plot asymmetry for anti-TG level (*p* = 0.0452), suggesting potential publication bias (Supplementary Material S7).

A summary of all the pooled results from the meta-analysis, including the subgroup analyses based on TSH- and BMI-matching, is presented in Table 5.

## 4. Discussion

This is the first study to comprehensively assess both the prevalence and serum levels of anti-TPO and anti-TG antibodies in women with and without PCOS. In this systematic review and meta-analysis, women with PCOS had significantly higher prevalences and levels of antithyroid autoantibodies. Anti-TPO prevalence was significantly increased in PCOS women (OR 2.03, 95% CI: [1.35–3.04], *p* = 0.0006), similarly to anti-TPO level (SMD 0.63, 95% CI: [0.37–0.88], *p* < 0.00001). Observed anti-TG prevalence was higher in PCOS patients (OR 1.92, 95% CI: [1.23–3.01], *p* = 0.004), as was anti-TG level (SMD 0.41, 95% CI: [0.18–0.64], *p* = 0.0004) (Table 5).

The vast majority of available systematic reviews focus on the risk of developing Hashimoto’s thyroiditis (HT) in women with PCOS, while only a limited number of studies include data on the prevalence or levels of thyroid autoantibodies in this population. Nevertheless, the findings across these studies are consistent—HT occurs significantly more frequently in women with PCOS than in the general population [17,20,21,68]. A systematic review published in 2022 confirmed the correlation between PCOS and HT. Interestingly, the meta-analysis demonstrated not only that patients with PCOS are more likely to develop HT than those without PCOS, but also that the risk of PCOS in patients with HT is higher than in those without HT [68]. Unfortunately, most studies do not explore the presence of specific thyroid autoantibodies in women with PCOS. One of the few systematic reviews and meta-analyses addressing this topic, which included eighteen studies with a total of 3657 participants, showed elevated levels of anti-TPO (SMD = 0.36; 95% CI: 0.19–0.53; *p* < 0.001) and anti-TG (SMD = 1.24; 95% CI: 0.37–2.10; *p* < 0.001) in the women with PCOS compared to the control groups [17]. Another study addressing this topic, published in 2013, included a total of six studies involving 726 PCOS patients and 879 controls. The results demonstrated that the likelihood of testing positive for anti-TPO (*p* = 0.02) and anti-TG (*p* = 0.04) antibodies was significantly higher in patients with PCOS compared to control subjects [20]. These findings are consistent with our results. This raises the question of what mechanisms might underlie the association between the presence of thyroid autoantibodies and PCOS. It is possible that the increased prevalence and elevated levels of autoantibodies in women with PCOS are driven by complex molecular, immunological, or hormonal pathways [69]. However, genetic factors may also contribute to this relationship, given that HT itself is more common among women with PCOS. Notably, three genetic polymorphisms have been proposed as potential shared risk factors for both conditions: FBN3, CYP1B1, and GNRHR [70]. Genetic polymorphisms in the fibrillin-3 (FBN3) gene in women with PCOS offer an interesting explanation for the coexistence of these two diseases. TGF-beta is a cytokine that stimulates regulatory T cells (Tregs), thereby inhibiting excessive and undesired immune responses [71]. Its levels are reduced both in HT and PCOS patients carrying the D19S884 allele 8 in the FBN3 gene. This may explain the exaggerated immune response, resulting in a higher incidence of autoimmune diseases such as HT in women with PCOS [68]. Another factor that may contribute to the increased prevalence and levels of thyroid autoantibodies in PCOS is the hormonal dysregulation typical of this condition: estrogen stimulates immune activity, while androgens and progesterone exert an inhibitory effect [16,72]. For example, testosterone enhances T suppressor cell activity, whereas progesterone inhibits macrophage proliferation, interleukin-6 (IL-6) synthesis, and peripheral antibody production [21]. Nonetheless, due to chronic anovulatory cycles and an elevated estrogen-to-progesterone ratio, a state of relative hyperestrogenism develops in PCOS patients and the elevated androgen levels commonly observed in PCOS patients do not appear to exert a sufficient protective effect against autoimmunity [21]. This may increase susceptibility to autoimmune disorders compared to the general population, helping to explain both the higher frequency and elevated levels of anti-TPO and anti-TG antibodies observed in these patients. Additionally, PCOS is recognized as a low-grade inflammatory condition. Women with PCOS exhibit elevated levels of proinflammatory cytokines, including C-reactive protein (CRP), tumor necrosis factor alpha (TNF-α), and IL-6 [14]. This chronic inflammatory state is likely associated with a dysfunction of the immune system in women with PCOS, which may in turn contribute to the higher prevalence of autoantibodies and autoimmune disorders observed in this population [73].

A subgroup analysis based on TSH-matching revealed that the higher prevalence of anti-TPO and anti-TG antibodies in women with PCOS was no longer statistically significant in the TSH-matched subgroup (anti-TPO: OR 1.28, 95% CI: [0.86–1.92], *p* = 0.23; anti-TG: OR 1.43, 95% CI: [0.86–2.37], *p* = 0.17), in contrast to the TSH-not matched subgroup (anti-TPO: OR 5.27, 95% CI: [3.00–9.27], *p* < 0.00001; anti-TG: OR 3.26, 95% CI: [1.55–6.86], *p* = 0.002). For both anti-TPO and anti-TG, elevated antibody levels remained statistically significant in the TSH-matched subgroup, although the association for anti-TG was weaker and closer to the threshold of significance (anti-TPO: SMD 0.59, 95% CI: [0.27–0.91], *p* = 0.0003; anti-TG: SMD 0.19, 95% CI: [0.01–0.36], *p* = 0.03) (Table 5).

The diagnosis of PCOS itself remains controversial, as thyroid disorders should be excluded during its assessment due to their ability to mimic PCOS symptoms [8]—a particularly relevant issue in the context of the present analysis focusing on thyroid autoantibodies in women with PCOS. To address this, some researchers often include only euthyroid women (based on normal TSH levels), others exclude women reporting any history of thyroid disease, while some studies do not apply such criteria at all and include women with previously diagnosed autoimmune thyroiditis. These differences in inclusion criteria are among the main reasons for high heterogeneity and the inconsistent findings across studies on this topic. To address this challenge, we stratified the studies based on whether thyroid function, as reflected by TSH levels, was comparable between the women with PCOS and the controls. This approach not only reduced heterogeneity, but also helped to clarify whether increased antibody levels were associated with PCOS per se, rather than secondary to unrecognized or subclinical hypothyroidism. These results support the view that PCOS may independently contribute to elevated anti-TPO and anti-TG antibody levels, as this association persisted even after TSH-matching. This may be due to several factors, including the hypothesis that PCOS predisposes individuals to various autoimmune reactions, as supported by the increased prevalence of autoimmune conditions observed in women with PCOS [74]. Moreover, PCOS has been recognized as a chronic low-grade inflammatory condition, which may play a role in immune system dysregulation and the development of autoimmunity [14,73]. There are also hypotheses suggesting that, at least in a subset of cases, autoantibodies may play a direct role in the pathogenesis of PCOS itself, which further highlights their potential significance [75].

Another subgroup analysis based on BMI-matching revealed that the higher prevalence of anti-TPO and anti-TG antibodies in women with PCOS was no longer statistically significant in the BMI-matched subgroup (anti-TPO: OR 1.61, 95% CI: [0.48–5.42], *p* = 0.45; anti-TG: OR 1.64, 95% CI: [0.59–4.52], *p* = 0.34), in contrast to the BMI-not matched subgroup (anti-TPO: OR 2.24, 95% CI: [1.49–3.38], *p* = 0.0001; anti-TG: OR 2.08, 95% CI: [1.25–3.45], *p* = 0.005). For anti-TPO levels, the elevation persisted in the BMI-matched group (SMD 0.31, 95% CI: [0.12–0.50], *p* = 0.001), whereas for anti-TG levels, statistical significance was lost in the BMI-matched subgroup (SMD 0.17, 95% CI: [−0.16–0.49], *p* = 0.32) (Table 5).

BMI is a key variable that may confound the observed relationship between PCOS and thyroid autoantibodies. Women with PCOS typically present with higher BMIs [76]; however, an increased BMI is not a diagnostic criterion for PCOS [8]. Some women exhibit a so-called lean phenotype of PCOS, which, despite being characterized by insulin resistance, does not involve an elevated BMI [77,78]. Moreover, the design of many studies tends to preferentially recruit women with the full-blown phenotype A, who show more pronounced clinical features and are often more obese than the average PCOS population [79]. As a result, the findings from such studies may be biased, as they reflect not only the effect of PCOS itself, but also the influence of obesity. Obesity, as indicated by an increased BMI, has been associated with a higher risk of AITD [80]. To address this confounding factor, we stratified the studies based on whether BMI significantly differed between the PCOS and control groups. In the BMI-matched subgroups, the only outcome that remained significantly elevated was anti-TPO levels; however, even these were significantly lower compared to the BMI-not matched subgroup. This suggests that BMI may influence thyroid autoantibody levels, particularly anti-TG, for which the previously significant elevation was no longer observed. The association between obesity and anti-TPO or anti-TG positivity remains inconsistent across studies. According to a cross-sectional survey of 2505 individuals, obesity was significantly associated with anti-TG positivity after adjusting for sex, age, and smoking, but not with a higher anti-TPO positivity rate [81], which is consistent with our findings. Other studies, however, have reported significant associations between both anti-TPO and anti-TG positivity and obesity [82], whereas some failed to confirm this relationship for anti-TPO [83]. It is possible that obesity contributes to the generation of both anti-TPO and anti-TG autoantibodies, although experimental models have led to the hypothesis that the production of anti-TG may occur earlier, followed by the development of anti-TPO [81,84,85].

It is also important to note that TSH level and BMI may influence each other [86], primarily through mechanisms involving insulin resistance [87]. These interconnected pathways not only affect the clinical presentation of PCOS but also complicate the interpretation of thyroid-related findings in this population.

Only a limited number of studies examined the clinical or hormonal factors potentially associated with thyroid autoantibodies in PCOS [28,31,33,35,39,47,59,61]. The most frequently assessed variables included BMI, TSH levels, PCOM, metabolic syndrome, age, and sex hormone levels. However, the results were often heterogeneous and inconclusive, making it difficult to determine whether these factors independently influence thyroid autoimmunity. There is a clear need for future studies with consistent methodologies, well-defined subgroups, and multivariable analyses to better identify the contributors to thyroid autoantibody elevation in PCOS patients.

This systematic review and meta-analysis have several limitations. Heterogeneity across outcomes was high, as reflected by the I^2^ values, particularly for anti-TPO and anti-TG levels. This can largely be attributed to substantial variability in the study design. Although most studies used the Rotterdam criteria, some applied alternative diagnostic definitions for PCOS, such as the NIH criteria. The inclusion criteria for the control groups also varied widely—some studies included women without PCOS, others specified ‘healthy controls’ and only some clearly defined euthyroid status based on normal TSH values. In some cases, women with a history of thyroid disease were excluded, while in others this was not mentioned at all. The exclusion criteria were even more inconsistent across studies, ranging from autoimmune conditions to smoking or specific medications. While most studies were age-matched, only a subset matched participants by BMI. Another source of variability was the use of different laboratory methods to assess antibody titers and the application of varying cut-off values to define antibody positivity. These methodological inconsistencies highlight the inherent difficulty in achieving full comparability across studies and help explain the high degree of heterogeneity observed in this analysis. To mitigate some of these differences, we stratified the analyses by TSH-matching and BMI-matching—two important variables that differed across the studies and may have influenced thyroid autoimmunity. Notably, heterogeneity (I^2^) decreased in several comparisons following this stratification, supporting this methodological strategy.

Another limitation is the varying methodological quality of the included studies. Although the majority (30 out of 40) were assessed as having a low risk of bias, 9 studies showed an intermediate risk, with 1 study rated as high risk (Table 4). However, excluding these studies from the sensitivity analyses did not significantly affect the overall outcomes or the results within the matched subgroups. The impact was observed only in certain unmatched subgroups, which generally included the majority of studies with lower quality scores on the NOS.

Additionally, the possibility of publication bias cannot be fully excluded. Several studies were identified as outliers and some asymmetry was observed—particularly in the case of anti-TPO prevalence. To further assess this, Egger’s regression tests were conducted for each outcome (Supplementary Material S7). The results indicated statistically significant asymmetry for anti-TPO prevalence and level, as well as for anti-TG level (*p* < 0.05), suggesting the presence of potential publication bias in these comparisons. However, this may be at least partially explained by the high degree of heterogeneity among the included studies. Sensitivity analyses confirmed the stability of the overall findings, although certain subgroup comparisons—especially for anti-TG levels—showed borderline significance that was lost upon the exclusion of specific studies. These findings highlight the need for cautious interpretation, particularly for outcomes close to the threshold of statistical significance.

Finally, some studies provided only percentages of the total without the number of participants or did not report data as continuous variables (means with standard deviations), requiring the use of statistical approximations for inclusion in the meta-analysis. While such methods are widely accepted in meta-analyses, they introduce additional uncertainty and should be interpreted with caution. To address this, a sensitivity analysis excluding studies with imputed data based on conversions was performed and confirmed that the overall results remained stable.

Our meta-analysis also has several strengths. To our knowledge, it is the first to comprehensively assess both the prevalence and serum levels of anti-TPO and anti-TG antibodies in women with and without PCOS, while taking into account important methodological factors such as TSH- and BMI-matching. Previous publications on this topic have mainly focused on the association between AITD and PCOS, while the role of thyroid autoantibodies has not been fully explored or systematically assessed. Subgroup analysis based on these variables allowed for a more refined analysis and helped reduce heterogeneity, but most importantly, it brought us closer to addressing the key question of whether PCOS itself contributes to elevated thyroid autoantibody levels, or whether this association is primarily driven by additional factors or by inconsistencies and bias in studies’ designs. Moreover, the inclusion of a large number of studies (40) and participants likely contributed to the overall stability of the findings, despite the high degree of heterogeneity. The inclusion of studies from diverse geographic regions may enhance the generalizability of the findings across different populations. This study followed PRISMA’s guidelines for systematic reviews, ensuring transparency and methodological quality. Sensitivity analyses were also systematically conducted, including leave-one-out procedures and risk-of-bias restrictions, providing insight into the stability of the findings.

## 5. Conclusions

Anti-TPO and anti-TG antibodies are more frequently detected and reach higher levels in women with PCOS. While factors such as BMI and TSH clearly contribute to this increase and should be carefully considered in the design of future research, PCOS itself appears to independently influence thyroid autoantibody levels. Given that elevated BMIs and TSH levels are common in PCOS patients, these individuals may be at a particularly increased risk of developing thyroid autoimmunity.

## Figures and Tables

**Table 2:** Main findings and antibody-associated factors in studies assessing the prevalence of thyroid autoantibodies included in the systematic review and meta-analysis.

Author, Year	Associated Factors	Main Findings
Studies assessing the prevalence of anti-TPO antibodies only (*n* = 9)		
Adamska et al., 2020 [33]	estradiol (+), PRL (−)	The prevalence of anti-TPO was not significantly different between women with PCOS and controls.
Heidarpour et al., 2023 [32]	ND	The prevalence of anti-TPO was significantly higher in women with PCOS compared to controls.
Kim et al., 2022 [37]	ND	The prevalence of anti-TPO was not significantly different between women with PCOS and controls.
Menon et al., 2017 [38]	ND	The prevalence of anti-TPO was significantly higher in women with PCOS compared to controls.
Naruka et al., 2023 [39]	T4 (−), TSH (+)	The prevalence of anti-TPO was not significantly different between women with PCOS and controls.
Sinha et al., 2013 [41]	ND	The prevalence of anti-TPO was significantly higher in women with PCOS compared to controls.
Tran et al., 2023 [42]	ND	The prevalence of anti-TPO was not significantly different between women with PCOS and controls.
Van der Ham et al., 2023 [43]	ND	The prevalence of anti-TPO was not significantly different between women with PCOS and controls.
Wang et al., 2018 [28]	PCOS women aged 28–35 years (+)	The prevalence of anti-TPO was significantly higher in women with PCOS compared to controls.
Studies assessing the prevalence of both anti-TG and anti-TPO antibodies (*n* = 16)		
Al-Saab et al., 2014 [46]	ND	The prevalence of both anti-TPO and anti-TG antibodies was significantly higher in women with PCOS compared to controls.
Anusha et al., 2024 [48]	ND	The prevalence of both anti-TPO and anti-TG antibodies was significantly higher in women with PCOS compared to controls.
Arduc et al., 2015 [49]	ND	The prevalence of both anti-TPO and anti-TG antibodies was significantly higher in women with PCOS compared to controls.
De Souza Mayrink Novais et al., 2015 [51]	ND	The prevalence of both anti-TPO and anti-TG antibodies was not significantly different between women with PCOS and controls.
Duran et al., 2015 [52]	ND	The prevalence of both anti-TPO and anti-TG antibodies was not significantly different between women with PCOS and controls.
El-Din et al., 2015 [53]	ND	The prevalence of both anti-TPO and anti-TG antibodies was not significantly different between women with PCOS and controls.
Garelli et al., 2013 [54]	ND	The prevalence of both anti-TPO and anti-TG antibodies was significantly higher in women with PCOS compared to controls.
Hepşen et al., 2019 [55]	ND	The prevalence of both anti-TPO and anti-TG antibodies was significantly higher in women with PCOS compared to controls.
Janssen et al., 2004 [56]	ND	The prevalence of thyroid autoantibodies was significantly higher in women with PCOS compared to controls (no separate data available for anti-TPO and anti-TG antibodies).
Kachuei et al., 2012 [57]	ND	The prevalence of both anti-TPO and anti-TG antibodies was not significantly different between women with PCOS and controls.
Karaköse et al., 2013 [58]	ND	The prevalence of anti-TPO antibodies was significantly higher in women with PCOS, while the prevalence of anti-TG antibodies was not significantly different between groups.
Karaköse et al., 2017 [59]	anti-TG: TSH (+)	The prevalence of anti-TPO antibodies was significantly higher in women with PCOS, while the prevalence of anti-TG antibodies was not significantly different between groups.
	anti-TPO: TSH (+)	
Mitkov et al., 2015 [61]	anti-TG: TSH (+), age (−)	The prevalence of thyroid autoantibodies was not significantly different between women with PCOS and controls (no separate data available for anti-TPO and anti-TG anti-bodies).
	anti-TPO: TSH (+), testosterone (−), DHEAS (−), age (+)	
Ott et al., 2010 [63]	ND	The prevalence of anti-TPO antibodies was significantly higher in women with PCOS, while the prevalence of anti-TG antibodies was not significantly different between groups.
Petrikova et al., 2015 [64]	ND	The prevalence of anti-TPO antibodies was significantly higher in women with PCOS, while the prevalence of anti-TG antibodies was not significantly different between groups.
Vryonidou et al., 2025 [66]	ND	The prevalence of both anti-TPO and anti-TG antibodies was not significantly different between women with PCOS and controls.

(+) indicates a positive correlation or association; (−) indicates a negative correlation or association. Anti-TG—anti-thyroglobulin antibodies; anti-TPO—anti-thyroid peroxidase antibodies; DHEAS—dehydroepiandrosterone sulfate; ND—no data; *n*—number of studies; PCOS—polycystic ovary syndrome; PRL—prolactin; T4—thyroxine; TSH—thyroid-stimulating hormone.

**Table 3:** Main findings and antibody-associated factors in studies assessing the levels of thyroid autoantibodies included in the systematic review and meta-analysis.

Author, Year	Associated Factors	Main Findings
Studies assessing the level of anti-TG antibodies only (*n* = 1)		
Kamil et al., 2020 [31]	BMI (+)	The level of anti-TG antibodies was significantly higher in women with PCOS compared to controls.
Studies assessing the level of anti-TPO antibodies only (*n* = 9)		
Dhoriyani et al., 2024 [35]	BMI ≥ 25 (+), LH (+), FSH (+), PCOM (+)	The level of anti-TPO antibodies was not significantly different between women with PCOS and controls. However, significantly higher levels were observed in PCOS women with PCOM or elevated BMI.
Gudovan et al., 2008 [36]	ND	The level of anti-TPO antibodies was significantly higher in women with PCOS compared to controls.
Jameel et al., 2022 [30]	ND	The level of anti-TPO antibodies was significantly higher in women with PCOS compared to controls.
Menon et al., 2017 [38]	ND	The level of anti-TPO antibodies was significantly higher in women with PCOS compared to controls.
Shafi et al., 2024 [40]	ND	The level of anti-TPO antibodies was significantly higher in women with PCOS compared to controls.
Sinha et al., 2013 [41]	ND	The level of anti-TPO antibodies was significantly higher in women with PCOS compared to controls.
Tran et al., 2023 [42]	ND	The level of anti-TPO antibodies was not significantly different between women with PCOS and controls.
Yasar et al., 2022 [44]	ND	The level of anti-TPO antibodies was not significantly different between women with PCOS and controls.
Yu et al., 2016 [45]	ND	The level of anti-TPO antibodies was not significantly different between women with PCOS and controls.
Studies assessing the level of both anti-TG and anti-TPO antibodies (*n* = 18)		
Al-Karawi et al., 2024 [29]	ND	The levels of both anti-TPO and anti-TG antibodies were significantly higher in women with PCOS compared to controls.
Al-Saab et al., 2014 [46]	ND	The level of anti-TPO antibodies was significantly higher in women with PCOS, while the level of anti-TG antibodies was not significantly different between groups.
Anaforoglu et al., 2011 [47]	anti-TG: metabolic syndrome (+), post-prandial glucose (+), LDL (+), triglycerides (+)	The levels of both anti-TPO and anti-TG antibodies were not significantly different between women with PCOS and controls. However, levels were significantly higher in women with metabolic syndrome.
	anti-TPO: metabolic syndrome (+), age (+), weight (+), waist circumference (+), diastolic blood pressure (+), post-prandial glucose (+), HDL (−)	
Arduc et al., 2015 [49]	ND	The levels of both anti-TPO and anti-TG antibodies were significantly higher in women with PCOS compared to controls.
Arora et al., 2016 [34]	ND	The level of anti-TPO antibodies was not significantly different between women with PCOS and controls. The level of anti-TG antibodies was significantly higher in women with PCOS compared to controls, although full results were not available.
Auda et al., 2023 [50]	ND	The levels of both anti-TPO and anti-TG antibodies were significantly higher in women with PCOS compared to controls.
El-Din et al., 2015 [53]	ND	The level of anti-TPO antibodies was significantly higher in women with PCOS, while the level of anti-TG antibodies was not significantly different between groups.
Hepşen et al., 2019 [55]	ND	The levels of both anti-TPO and anti-TG antibodies were significantly higher in women with PCOS compared to controls.
Janssen et al., 2004 [56]	ND	The levels of both anti-TPO and anti-TG antibodies were significantly higher in women with PCOS compared to controls.
Kachuei et al., 2012 [57]	ND	The level of anti-TPO antibodies was significantly higher in women with PCOS, while the level of anti-TG antibodies was not significantly different between groups.
Karaköse et al., 2013 [58]	ND	The levels of both anti-TPO and anti-TG antibodies were not significantly different between women with PCOS and controls.
Malik Aubead et al., 2020 [60]	ND	The levels of both anti-TPO and anti-TG antibodies were significantly higher in women with PCOS compared to controls.
Mitkov et al., 2015 [61]	anti-TG: TSH (+), age (−)	The level of anti-TG antibodies was significantly higher in women with PCOS, while the level of anti-TPO antibodies was not significantly different between groups.
	anti-TPO: TSH (+), testosterone (−), DHEAS (−), age (+)	
Mohammed et al., 2017 [62]	ND	The levels of both anti-TPO and anti-TG antibodies were not significantly different between women with PCOS and controls.
Ott et al., 2010 [63]	ND	The levels of both anti-TPO and anti-TG antibodies were significantly higher in women with PCOS compared to controls. Elevated anti-TPO levels may indicate a risk for clomiphene citrate resistance.
Petrikova et al., 2015 [64]	ND	The levels of both anti-TPO and anti-TG antibodies were not significantly different between women with PCOS and controls.
Saleh et al., 2021 [65]	ND	The level of anti-TPO antibodies was significantly higher in women with PCOS, while the level of anti-TG antibodies was not significantly different between groups.
Yasar et al., 2016 [67]	ND	The levels of both anti-TPO and anti-TG antibodies were not significantly different between women with PCOS and controls.

(+) indicates a positive correlation or association; (−) indicates a negative correlation or association. Anti-TG—anti-thyroglobulin antibodies; anti-TPO—anti-thyroid peroxidase antibodies; BMI—body mass index; DHEAS—dehydroepiandrosterone sulfate; FSH—follicle-stimulating hormone; HDL—high-density lipoprotein; LDL—low-density lipoprotein; LH—luteinizing hormone; ND—no data; *n*—number of studies; PCOM—polycystic ovarian morphology; PCOS—polycystic ovary syndrome; TSH—thyroid-stimulating hormone.

**Table 4:** Quality assessment of the included studies using the modified Newcastle–Ottawa Scale.

Author, Year	Selection				Comparability	Outcome			Sum
	PCOS Definition	Inclusion/Exclusion Criteria	Control Group	Antibody Measurement	Confounders	Clear Results	Antibodies Influencing Factors	Statistical Analysis	
Adamska et al., 2020 [33]	★	★	★	★	★★	★		★	8
Al-Karawi et al., 2024 [29]		★		★		★		★	4
Al-Saab et al., 2014 [46]	★	★	★	★	★	★	★	★	8
Anaforoglu et al., 2011 [47]	★	★	★	★	★★	★	★	★	9
Anusha et al., 2024 [48]	★	★	★	★	★	★	★	★	8
Arduc et al., 2015 [49]	★	★	★	★	★★	★	★	★	9
Arora et al., 2016 [34]	★	★	★	★	★★	★		★	8
Auda et al., 2023 [50]	★		★	★	★	★		★	6
De Souza Mayrink Novais et al., 2015 [51]	★	★	★	★		★	★	★	7
Dhoriyani et al., 2024 [35]	★	★	★		★★	★	★	★	8
Duran et al., 2025 [52]	★	★		★	★★	★		★	7
El-Din et al., 2015 [53]	★	★	★	★	★	★	★	★	8
Garelli et al., 2013 [54]	★	★	★	★		★		★	6
Gudovan et al., 2008 [36]	★			★	★	★			4
Heidarpour et al., 2023 [32]			★	★					2
Hepşen et al., 2018 [55]	★	★	★	★	★★		★	★	8
Jameel et al., 2022 [30]		★		★	★★	★	★	★	7
Janssen et al., 2022 [30]	★	★	★	★	★	★		★	7
Kachuei et al., 2012 [57]	★	★		★	★★	★		★	7
Kamil et al., 2020 [31]		★		★	★	★	★	★	6
Karaköse et al., 2013 [58]	★	★		★	★★	★	★	★	8
Karaköse et al., 2017 [59]	★	★		★	★★	★		★	7
Kim et al., 2022 [37]	★	★	★	★	★★	★	★	★	9
Malik Aubead et al., 2020 [60]	★	★	★		★			★	5
Menon et al., 2017 [38]	★		★	★	★★	★		★	7
Mitkov et al., 2015 [61]	★	★		★	★★	★	★	★	8
Mohammed et al., 2017 [62]	★	★	★	★	★★	★		★	8
Naruka et al., 2023 [39]	★	★	★	★	★★	★	★	★	9
Ott et al., 2010 [63]	★		★			★		★	4
Petrikova et al., 2015 [64]	★	★	★	★	★★	★	★	★	9
Saleh et al., 2021 [65]	★			★		★		★	4
Shafi et al., 2024 [40]	★	★	★		★★	★		★	7
Sinha et al., 2013 [41]	★	★	★	★	★	★		★	7
Tran et al., 2023 [42]	★	★	★	★	★★	★		★	8
Van der Ham et al., 2023 [43]	★	★	★	★	★	★	★	★	8
Vryonidou et al., 2025 [66]	★	★	★	★	★★	★	★	★	9
Wang et al., 2018 [28]		★			★★	★	★	★	6
Yasar et al., 2016 [67]	★	★		★	★★	★	★	★	8
Yasar et al., 2022 [44]	★	★		★	★★	★	★	★	8
Yu et al., 2016 [45]	★	★	★	★	★★	★	★	★	9

Stars indicate points awarded according to the modified Newcastle–Ottawa Scale. The maximum number of stars is 4 for Selection, 2 for Comparability, and 3 for Outcome. The ‘Sum’ column represents the total number of stars assigned (maximum score: 9). PCOS—polycystic ovary syndrome.

**Table 5:** Summary of pooled prevalence and level estimates for anti-TPO and anti-TG antibodies, including TSH- and BMI-based subgroup analyses.

Antibody	Outcome	Overall	TSH-Matched	TSH-Not Matched	BMI-Matched	BMI-Not Matched
Anti-TPO	Prevalence	OR 2.03 [1.35–3.04], *p* = 0.0006 *	OR 1.28 [0.86–1.92], *p* = 0.23	OR 5.27 [3.00–9.27], *p* < 0.00001 *	OR 1.61 [0.48–5.42], *p* = 0.45	OR 2.24 [1.49–3.38], *p* = 0.0001 *
	Level	SMD 0.63 [0.37–0.88], *p* < 0.00001 *	SMD 0.59 [0.27–0.91], *p* = 0.0003 *	SMD 0.71 [0.27–1.14], *p* = 0.002 *	SMD 0.31 [0.12–0.50], *p* = 0.001 *	SMD 0.82 [0.45–1.18], *p* < 0.0001 *
Anti-TG	Prevalence	OR 1.92 [1.23–3.01], *p* = 0.004 *	OR 1.43 [0.86–2.37], *p* = 0.17	OR 3.26 [1.55–6.86], *p* = 0.002 *	OR 1.64 [0.59–4.52], *p* = 0.34	OR 2.08 [1.25–3.45], *p* = 0.005 *
	Level	SMD 0.41 [0.18–0.64], *p* = 0.0004 *	SMD 0.19 [0.01–0.36], *p* = 0.03 *	SMD 0.75 [0.27–1.23], *p* = 0.002 *	SMD 0.17 [−0.16–0.49], *p* = 0.32	SMD 0.53 [0.23–0.83], *p* = 0.0005 *

Statistically significant results (*p* < 0.05) are indicated with an asterisk (*); 95% confidence intervals are presented in square brackets ([]). Anti-TG—anti-thyroglobulin antibodies; anti-TPO—anti-thyroid peroxidase antibodies; BMI—body mass index; CI –confidence interval; OR—odds ratio; PCOS—polycystic ovary syndrome; SMD—standardized mean difference; TSH—thyroid-stimulating hormone.

## Data Availability

The raw data supporting the conclusions of this article will be made available by the authors on request.

## Supplementary Materials

The following supporting information can be downloaded at https://www.mdpi.com/article/10.3390/ijms26157525/s1.
