# Supplementary material for: Prevalence and Levels of Thyroid Autoantibodies in Polycystic Ovary Syndrome—Impact of TSH- and BMI-Matched Comparisons: A Systematic Review and Meta-Analysis
Source: Int J Mol Sci. 2025 Aug 4;26(15):7525. doi: 10.3390/ijms26157525 (PMC12347112; doi:10.3390/ijms26157525)
Supplement: Supplementary file 1 [file ijms-26-07525-s001.zip › Supplementary material S1 – search strategy.pdf]

SUPPLEMENTARY MATERIAL S1 – SEARCH STRATEGY

A comprehensive literature search was performed on April 1, 2025, using three major databases: PubMed, Embase, and Scopus. The aim was to identify observational studies evaluating the presence or levels of autoantibodies in women with polycystic ovary syndrome (PCOS). The search strategy included terms related to autoimmunity and PCOS, applied to the title, abstract, keywords, or MeSH terms (where applicable). No date or language restrictions were applied.

The full electronic search strategies for each database were as follows:

PubMed (n = 379)

((antibodies[Title/Abstract]) OR (autoantibodies[Title/Abstract]) OR (autoimmunity[Title/Abstract]) OR (autoimmune[Title/Abstract]) OR ("autoimmune antibodies"[Title/Abstract]) OR ("self-antibodies"[Title/Abstract]) OR ("autoreactive antibodies"[Title/Abstract]) OR ("autoantigen antibodies"[Title/Abstract]) OR (autoimmunization[Title/Abstract]) OR ("immune dysregulation"[Title/Abstract]) OR ("autoimmune disease"[Title/Abstract]) OR (autoantibodies[MeSH Terms]) OR (autoimmunity[MeSH Terms])) AND ((polycystic ovary syndrome[Title/Abstract]) OR ("polycystic ovarian syndrome"[Title/Abstract]) OR (PCOS[Title/Abstract]) OR ("Stein-Leventhal syndrome"[Title/Abstract]) OR ("polycystic ovary disease"[Title/Abstract]) OR ("polycystic ovarian disease"[Title/Abstract]) OR ("sclerocystic ovary syndrome"[Title/Abstract]) OR (PCOD[Title/Abstract]) OR (polycystic ovarian syndrome[MeSH Terms]) OR (polycystic ovary syndrome[MeSH Terms]) OR (syndrome, polycystic ovary[MeSH Terms]))

Embase (via Elsevier) (n = 646)

(antibodies:ti,ab,kw OR autoantibodies:ti,ab,kw OR autoimmunity:ti,ab,kw OR autoimmune:ti,ab,kw OR 'autoimmune antibodies':ti,ab,kw OR 'self-antibodies':ti,ab,kw OR 'autoreactive antibodies':ti,ab,kw OR 'autoantigen antibodies':ti,ab,kw OR autoimmunization:ti,ab,kw OR 'immune dysregulation':ti,ab,kw OR 'autoimmune disease':ti,ab,kw) AND ('polycystic ovary syndrome':ti,ab,kw OR 'polycystic ovarian syndrome':ti,ab,kw OR pcos:ti,ab,kw OR 'stein-leventhal syndrome':ti,ab,kw OR 'polycystic ovary disease':ti,ab,kw OR 'polycystic ovarian disease':ti,ab,kw OR 'sclerocystic ovary syndrome':ti,ab,kw OR pcod:ti,ab,kw)

Scopus (n = 730)

TITLE-ABS-KEY (antibodies OR autoantibodies OR autoimmunity OR autoimmune OR "autoimmune antibodies" OR "self-antibodies" OR "autoreactive antibodies" OR "autoantigen antibodies" OR autoimmunization OR "immune dysregulation" OR "autoimmune disease") AND ("polycystic ovary syndrome" OR "polycystic ovarian syndrome" OR pcos OR "Stein-Leventhal syndrome" OR "polycystic ovary disease" OR "polycystic ovarian disease" OR "sclerocystic ovary syndrome" OR pcod)
