# Supplementary material for: Prevalence and Levels of Thyroid Autoantibodies in Polycystic Ovary Syndrome—Impact of TSH- and BMI-Matched Comparisons: A Systematic Review and Meta-Analysis
Source: Int J Mol Sci. 2025 Aug 4;26(15):7525. doi: 10.3390/ijms26157525 (PMC12347112; doi:10.3390/ijms26157525)
Supplement: Supplementary file 1 [file ijms-26-07525-s001.zip › Supplementary material S2 – modified Newcastle-Ottawa quality assessment scale.pdf]

SUPPLEMENTARY MATERIAL S2: MODIFIED NEWCASTLE-OTTAWA QUALITY ASSESSMENT SCALE

(Adapted to assess observational studies evaluating autoantibodies in women with PCOS)

Note: A study can be awarded a maximum of one star (★) for each criterion fulfilled within the Selection, Comparability, and Outcome domains, unless otherwise specified.

I. SELECTION DOMAIN ***(Maximum score: 4 stars)***

1. **Use of Internationally Recognized PCOS Definition**
   Yes: Internationally recognized diagnostic criteria for PCOS (e.g., Rotterdam, NIH) were clearly stated and applied. ★
   No: No PCOS definition was provided, or a non-standard definition was used.
2. **Clearly Defined and Appropriate Inclusion/Exclusion Criteria**
   Yes: Inclusion and exclusion criteria for the PCOS group were clearly stated and aligned with the diagnostic criteria used (e.g., Rotterdam, NIH), ensuring appropriate selection of participants. ★
   No: Criteria were not stated, incomplete, or not aligned with established diagnostic standards.
3. **Proper Selection and Description of a Control Group**
   Yes: The control group consisted of women without PCOS and was clearly defined with appropriate criteria (e.g., disease-free, reproductive age, regular menstrual cycles, no signs of hyperandrogenism, normal ovarian morphology, and normal hormone levels), and adequately described. ★
   No: Control group selection or health status description was unclear or insufficient.
4. **Use and Reporting of a Validated Method for Autoantibody Measurement**
   Yes: A validated method for autoantibody measurement was clearly reported (e.g., enzyme-linked immunosorbent assay [ELISA], chemiluminescent immunoassay [CLIA], electrochemiluminescence immunoassay [ECLIA]), including reference ranges or assay details. ★
   No: The method for autoantibody measurement was not reported, insufficiently described, or not validated.

II. COMPARABILITY DOMAIN ***(Maximum score: 2 stars)***

1. **Age Matching or Statistical Control**
   Yes: Age was matched or statistically controlled for between PCOS and control groups. ★
   No: Age was not matched or controlled for.
2. **Matching or Control for BMI or TSH**
   Yes: Either BMI or TSH levels were matched or statistically controlled for between

groups. ★
No: Neither BMI nor TSH were matched or controlled for.

Rationale: This approach aligns with the original Newcastle-Ottawa Scale, which assigns one point for the most important confounding factor (age), and a second point for any additional relevant factor. Since dual matching for both BMI and TSH was uncommon among the included studies, requiring both would have been overly restrictive and excluded the majority of studies from scoring. Therefore, one point was awarded if at least one of these two key variables (BMI or TSH) was matched or statistically controlled for, maintaining consistency with the NOS framework.

III. OUTCOME DOMAIN ***(Maximum score: 3 stars)***

1. **Clarity of Outcome Presentation**
   Yes: Outcomes (e.g., prevalence or levels of autoantibodies) were clearly reported, with consistent and unambiguous presentation (e.g., clear distinction between positive and negative cases, no discrepancies between text and tables). ★
   No: Outcome data were presented unclearly, inconsistently, or lacked key information needed for interpretation.
2. **Consideration of Potential Confounders**
   Yes: The study clearly addressed key confounders influencing autoantibody levels, particularly by including only euthyroid participants and/or excluding individuals with a history of thyroid disease. ★
   No: Potential confounders were not adequately considered or controlled for.
3. **Appropriate Statistical Methods**
   Yes: Statistical methods used to analyze the outcomes (e.g., prevalence or levels of autoantibodies) were appropriate and clearly reported. ★
   No: Statistical methods were inappropriate, unclear, or not adequately reported.

Risk of Bias Interpretation:

Studies were categorized as low risk of bias (7–9 stars), moderate risk (4–6 stars), and high risk (0–3 stars).

Adapted from the Newcastle-Ottawa Scale for assessing the quality of nonrandomized studies in meta-analyses. Available at: https://www.ohri.ca/programs/clinical_epidemiology/oxford.asp
