# Supplementary material for: Prevalence and Levels of Thyroid Autoantibodies in Polycystic Ovary Syndrome—Impact of TSH- and BMI-Matched Comparisons: A Systematic Review and Meta-Analysis
Source: Int J Mol Sci. 2025 Aug 4;26(15):7525. doi: 10.3390/ijms26157525 (PMC12347112; doi:10.3390/ijms26157525)
Supplement: Supplementary file 1 [file ijms-26-07525-s001.zip › Supplementary material S7 – Egger’s regression test.pdf]

SUPPLEMENTARY MATERIAL S7 – EGGER'S REGRESSION TEST FOR FUNNEL PLOT ASYMMETRY

To assess potential publication bias in the meta-analyses of anti-thyroid autoantibodies in PCOS, Egger's regression test was performed separately for each outcome. The test evaluates the relationship between the effect size and its standard error, with a statistically significant intercept suggesting potential small-study effects or publication bias. The analysis was conducted using a linear regression model with inverse variance weighting and the standard error as the predictor variable.

Antibody	Outcome	t-value	df	Bias estimate	SE (bias)	p value
Anti-TPO	Prevalence	3.31	21	2.9057	0.8768	0.0033
	Level	4.12	26	5.5779	1.3522	0.0003
Anti-TG	Prevalence	1.99	12	2.2952	1.1561	0.0705
	Level	2.16	17	4.6847	2.1669	0.0452
