# Supplementary figures and images for: Prevalence and Levels of Thyroid Autoantibodies in Polycystic Ovary Syndrome—Impact of TSH- and BMI-Matched Comparisons: A Systematic Review and Meta-Analysis (part 2 of 2)
Source: Int J Mol Sci. 2025 Aug 4;26(15):7525. doi: 10.3390/ijms26157525 (PMC12347112; doi:10.3390/ijms26157525)

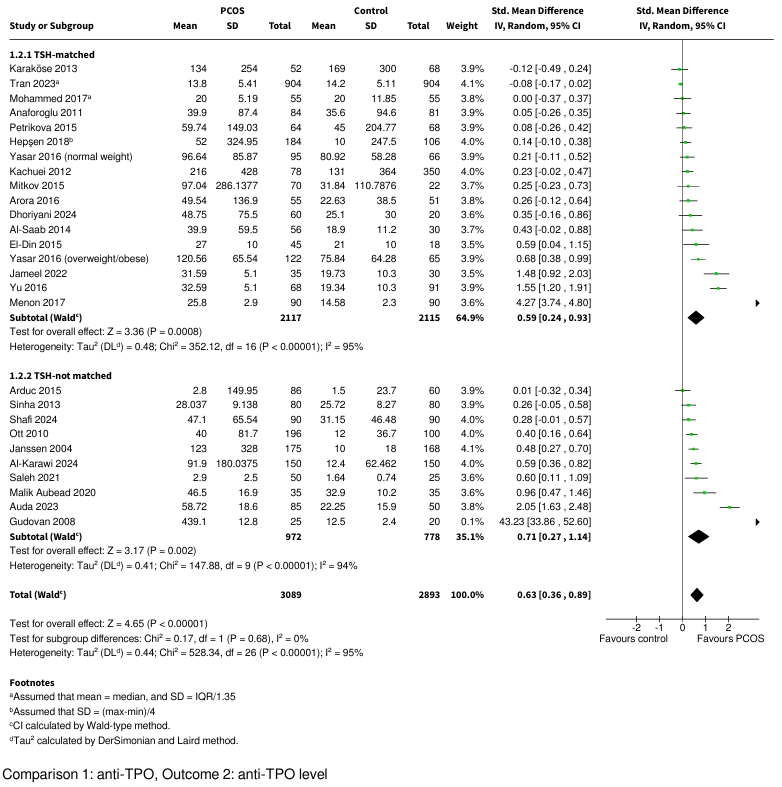

Supplement: Supplementary file 1 [file ijms-26-07525-s001.zip › Supplementary material S4 – sensitivity analyses anti-TPO level/SUPPLEMENTARY MATERIAL S4N.png]

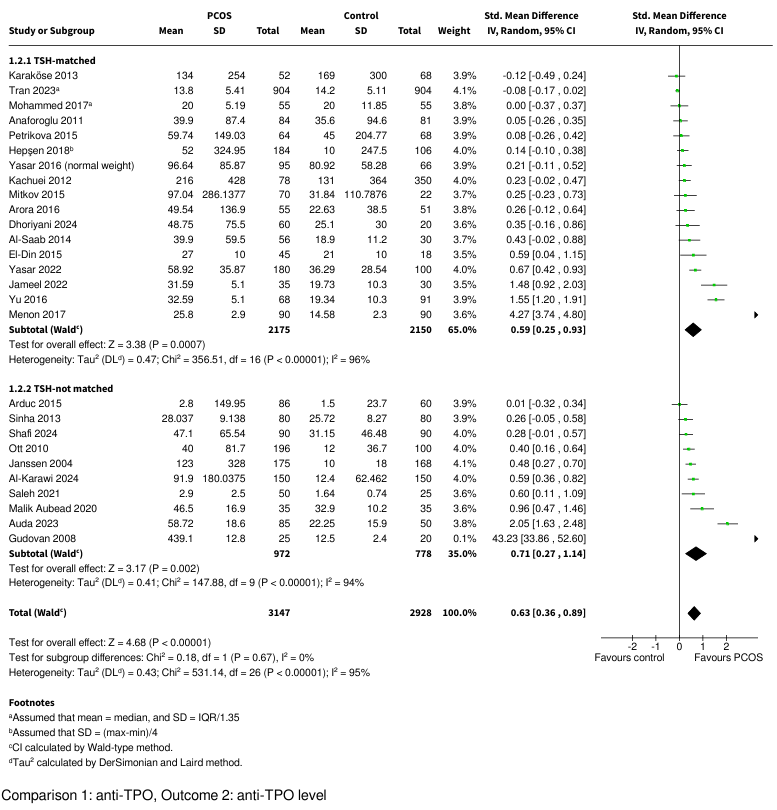

Supplement: Supplementary file 1 [file ijms-26-07525-s001.zip › Supplementary material S4 – sensitivity analyses anti-TPO level/SUPPLEMENTARY MATERIAL S4O.png]

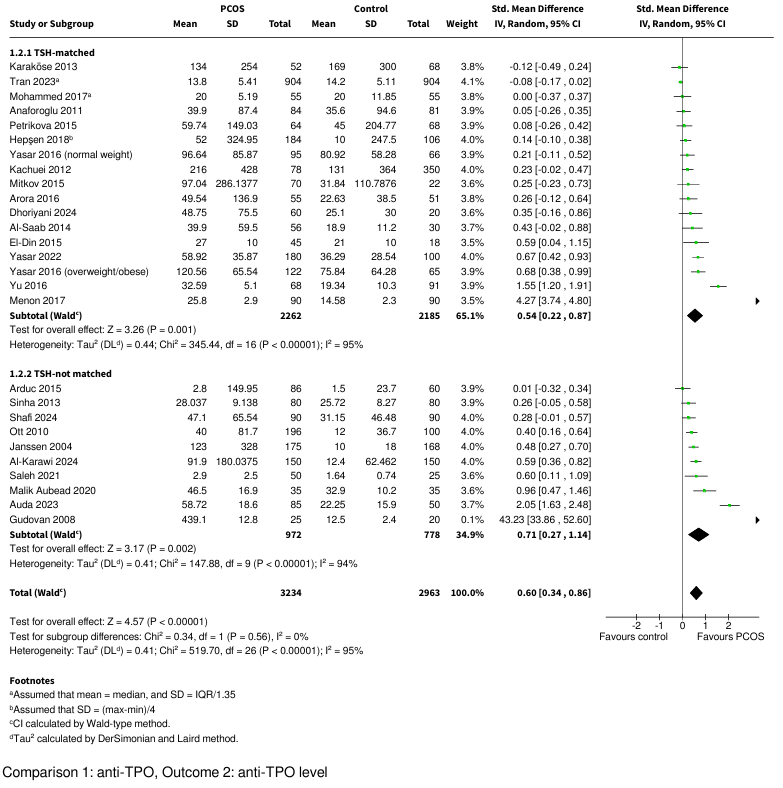

Supplement: Supplementary file 1 [file ijms-26-07525-s001.zip › Supplementary material S4 – sensitivity analyses anti-TPO level/SUPPLEMENTARY MATERIAL S4P.png]

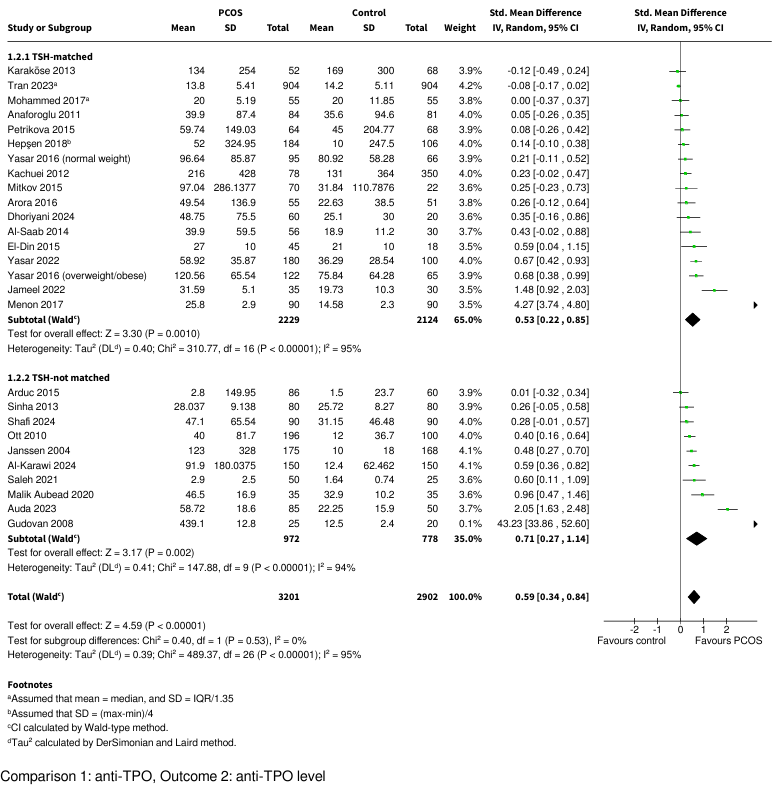

Supplement: Supplementary file 1 [file ijms-26-07525-s001.zip › Supplementary material S4 – sensitivity analyses anti-TPO level/SUPPLEMENTARY MATERIAL S4Q.png]

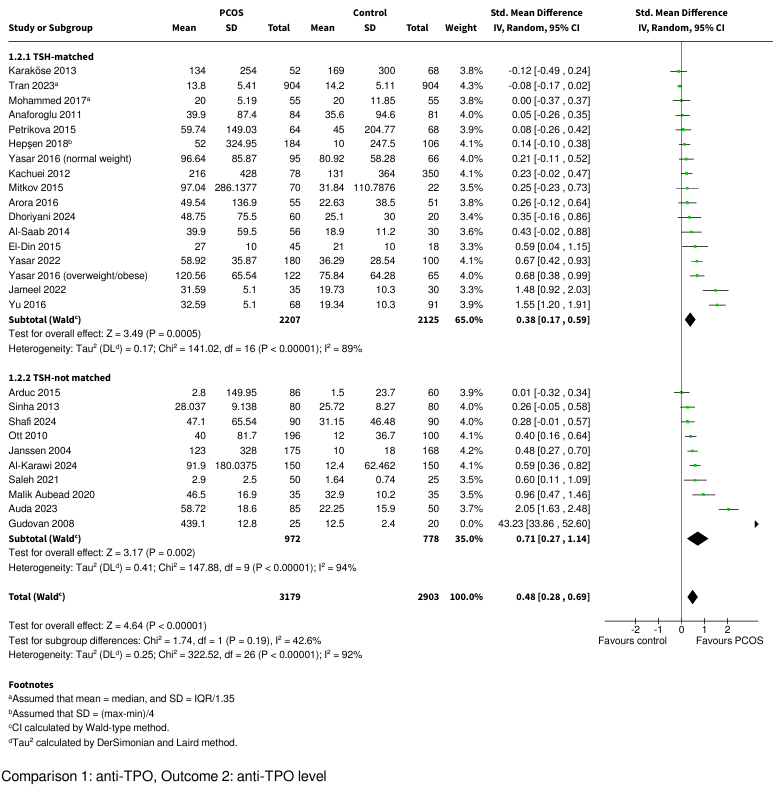

Supplement: Supplementary file 1 [file ijms-26-07525-s001.zip › Supplementary material S4 – sensitivity analyses anti-TPO level/SUPPLEMENTARY MATERIAL S4R.png]

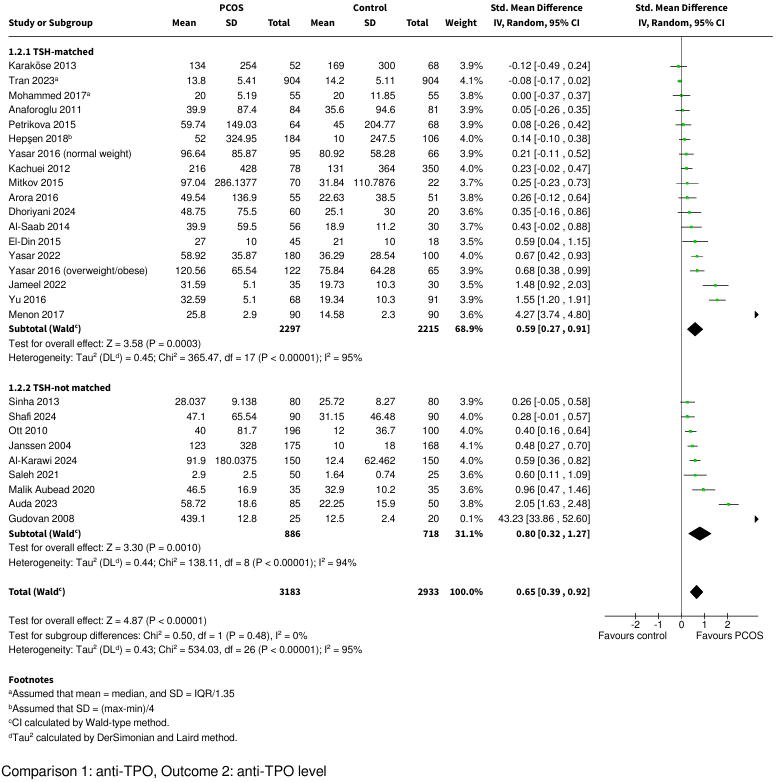

Supplement: Supplementary file 1 [file ijms-26-07525-s001.zip › Supplementary material S4 – sensitivity analyses anti-TPO level/SUPPLEMENTARY MATERIAL S4S.png]

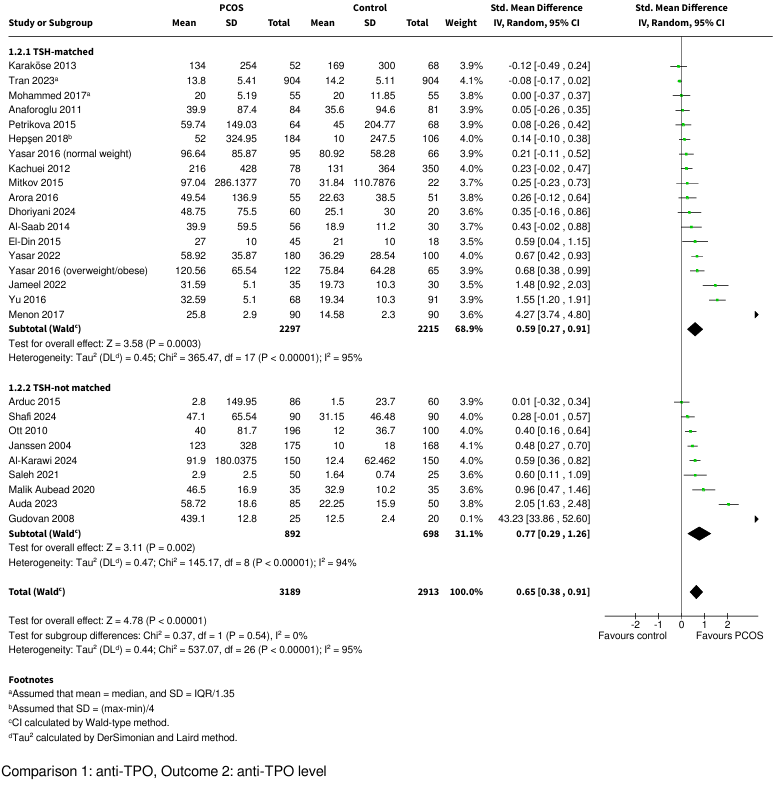

Supplement: Supplementary file 1 [file ijms-26-07525-s001.zip › Supplementary material S4 – sensitivity analyses anti-TPO level/SUPPLEMENTARY MATERIAL S4T.png]

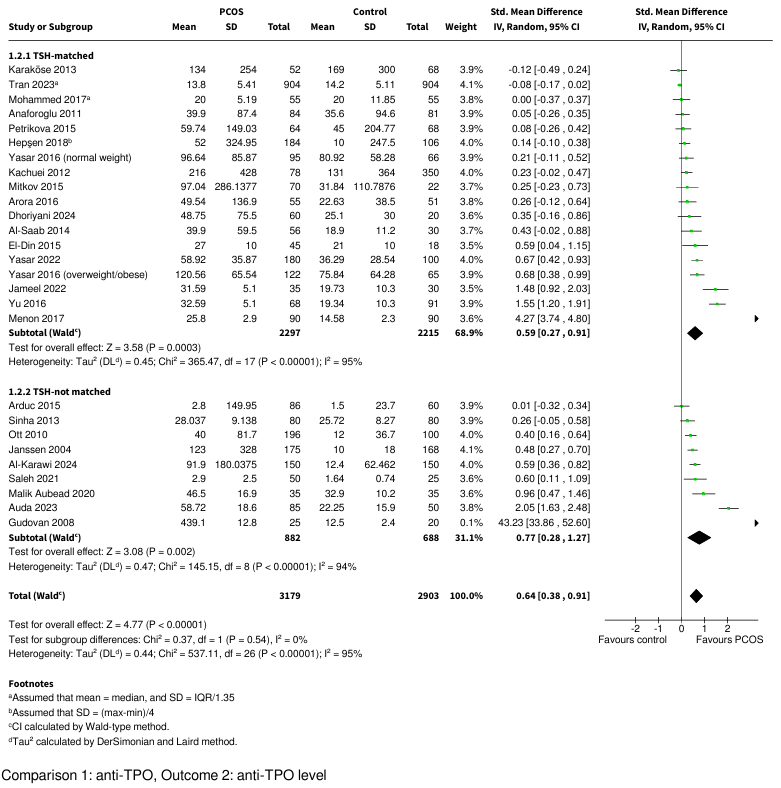

Supplement: Supplementary file 1 [file ijms-26-07525-s001.zip › Supplementary material S4 – sensitivity analyses anti-TPO level/SUPPLEMENTARY MATERIAL S4U.png]

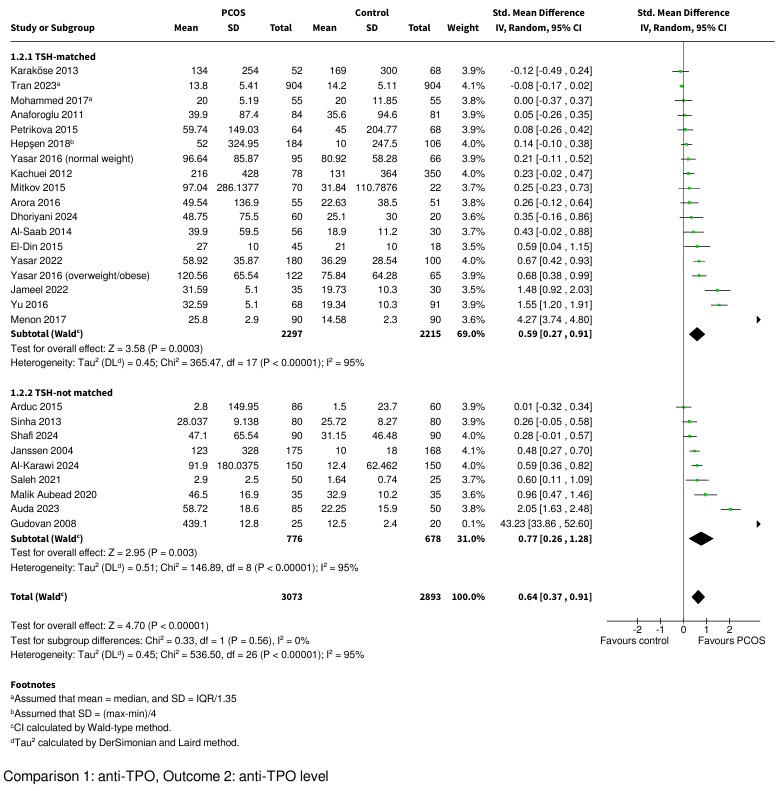

Supplement: Supplementary file 1 [file ijms-26-07525-s001.zip › Supplementary material S4 – sensitivity analyses anti-TPO level/SUPPLEMENTARY MATERIAL S4V.png]

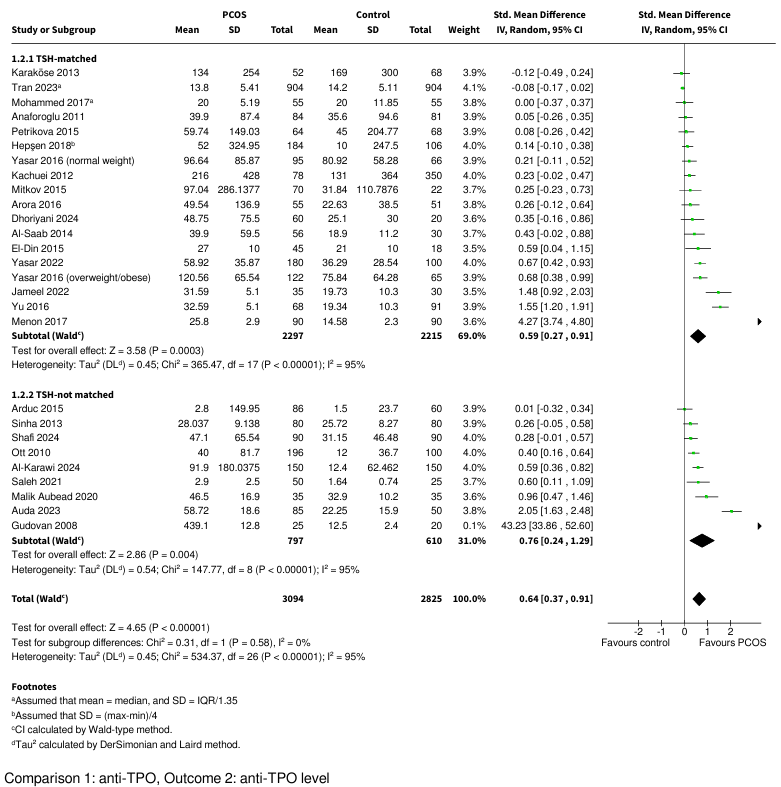

Supplement: Supplementary file 1 [file ijms-26-07525-s001.zip › Supplementary material S4 – sensitivity analyses anti-TPO level/SUPPLEMENTARY MATERIAL S4W.png]

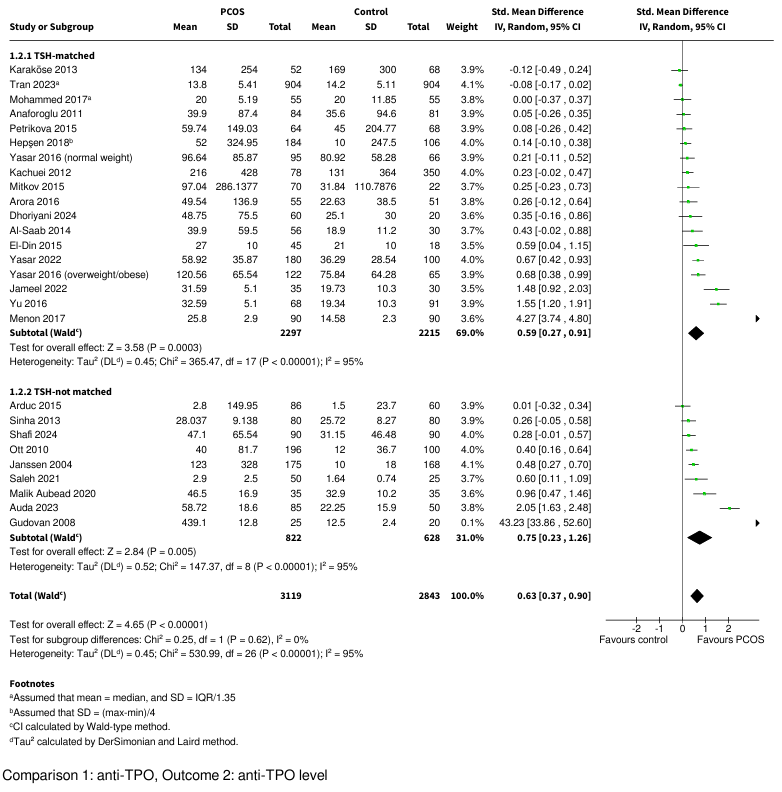

Supplement: Supplementary file 1 [file ijms-26-07525-s001.zip › Supplementary material S4 – sensitivity analyses anti-TPO level/SUPPLEMENTARY MATERIAL S4X.png]

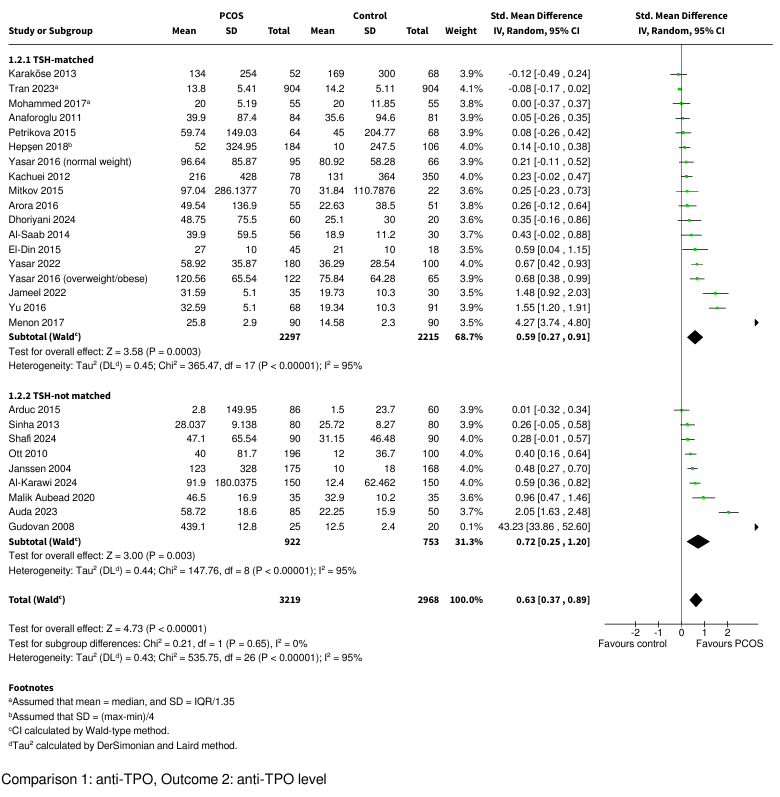

Supplement: Supplementary file 1 [file ijms-26-07525-s001.zip › Supplementary material S4 – sensitivity analyses anti-TPO level/SUPPLEMENTARY MATERIAL S4Y.png]

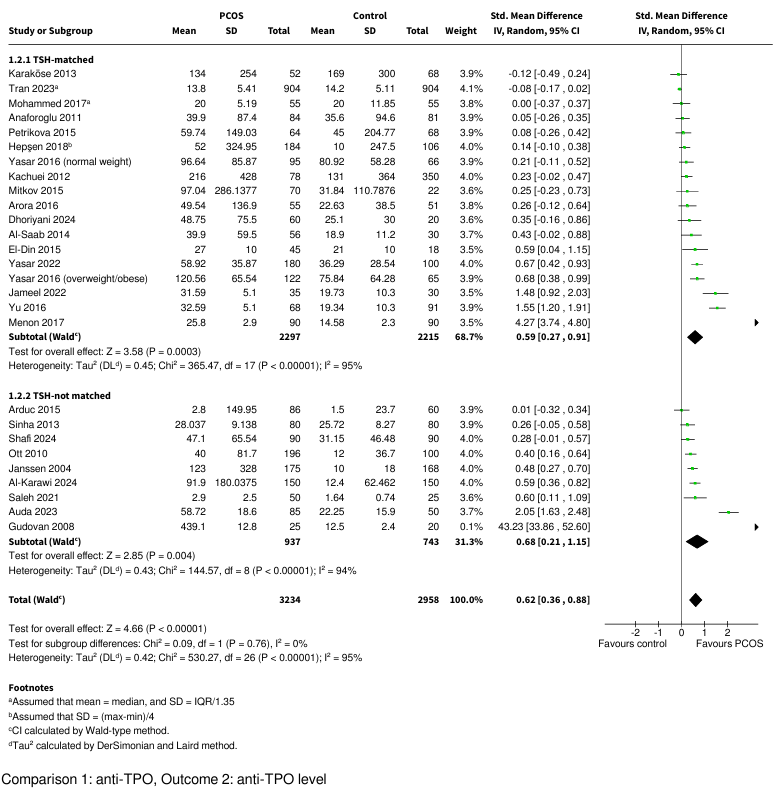

Supplement: Supplementary file 1 [file ijms-26-07525-s001.zip › Supplementary material S4 – sensitivity analyses anti-TPO level/SUPPLEMENTARY MATERIAL S4Z.png]

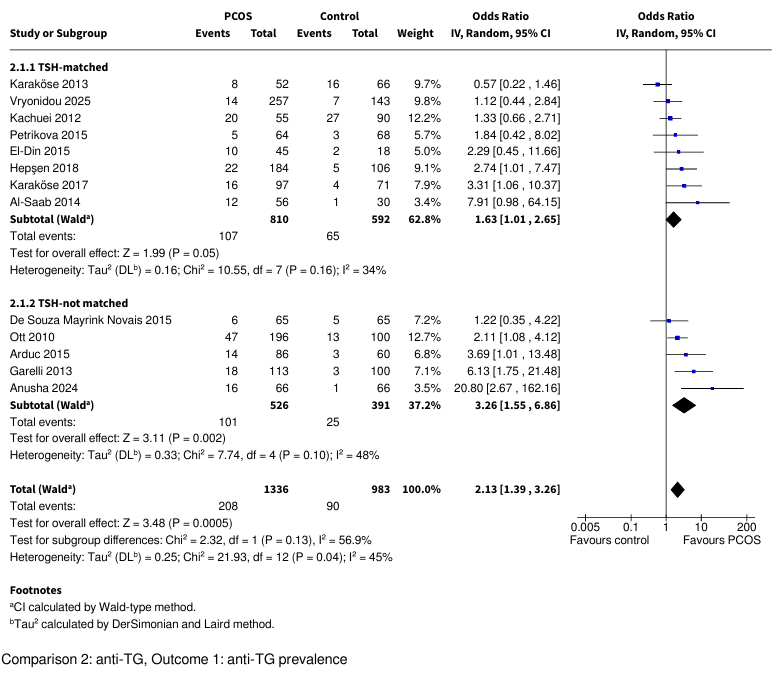

Supplement: Supplementary file 1 [file ijms-26-07525-s001.zip › Supplementary material S5 – sensitivity analyses anti-TG prevalence/SUPPLEMENTARY MATERIAL S5A.png]

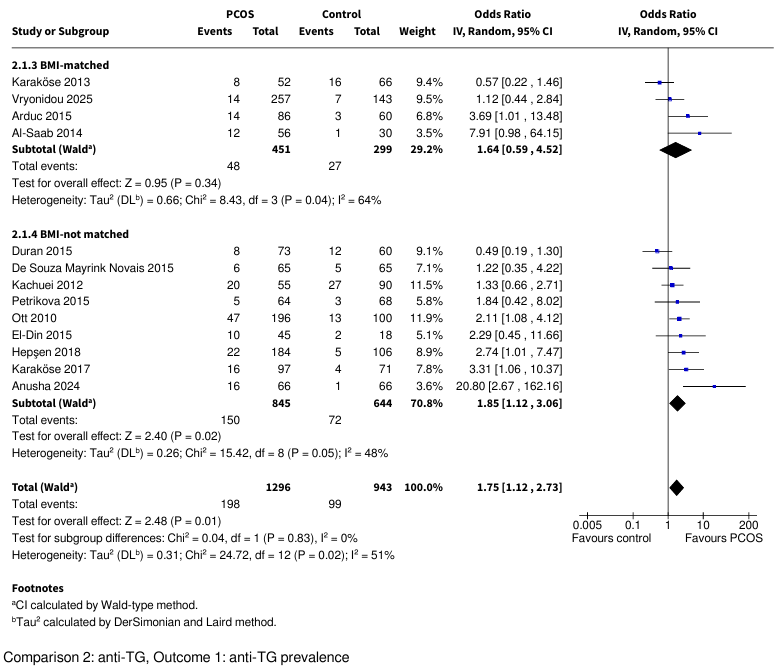

Supplement: Supplementary file 1 [file ijms-26-07525-s001.zip › Supplementary material S5 – sensitivity analyses anti-TG prevalence/SUPPLEMENTARY MATERIAL S5AA.png]

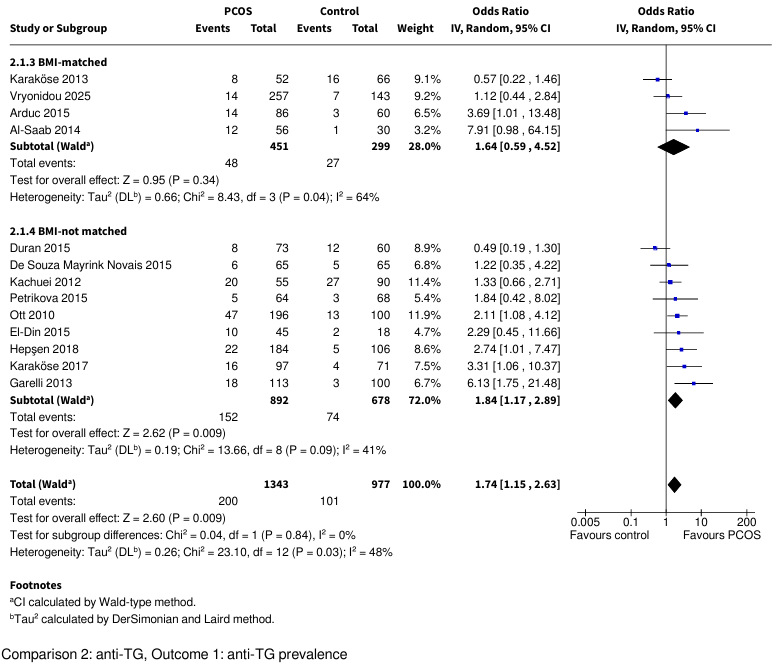

Supplement: Supplementary file 1 [file ijms-26-07525-s001.zip › Supplementary material S5 – sensitivity analyses anti-TG prevalence/SUPPLEMENTARY MATERIAL S5AB.png]

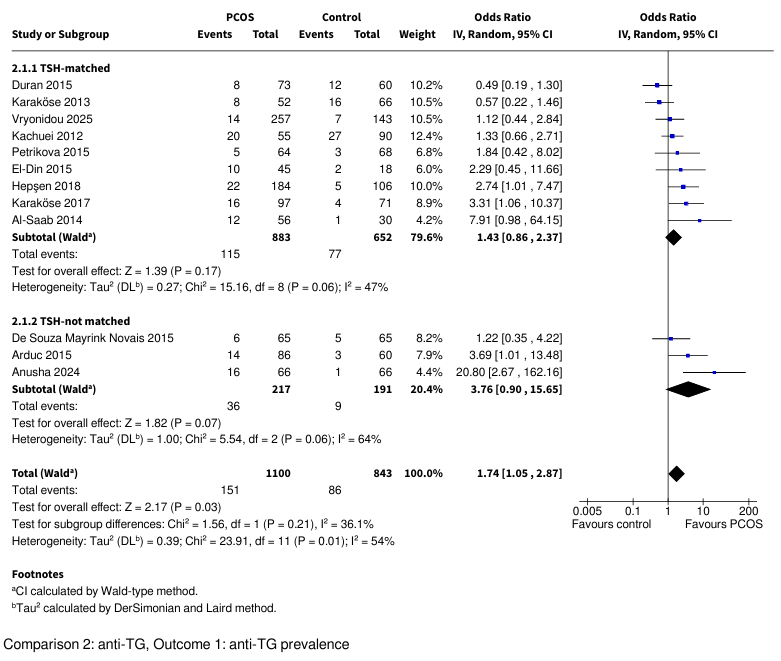

Supplement: Supplementary file 1 [file ijms-26-07525-s001.zip › Supplementary material S5 – sensitivity analyses anti-TG prevalence/SUPPLEMENTARY MATERIAL S5AC.png]

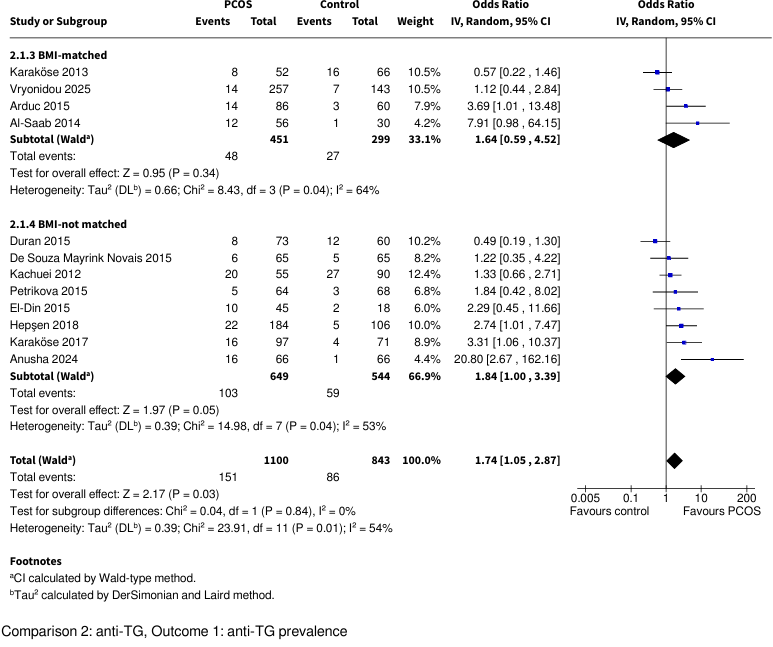

Supplement: Supplementary file 1 [file ijms-26-07525-s001.zip › Supplementary material S5 – sensitivity analyses anti-TG prevalence/SUPPLEMENTARY MATERIAL S5AD.png]

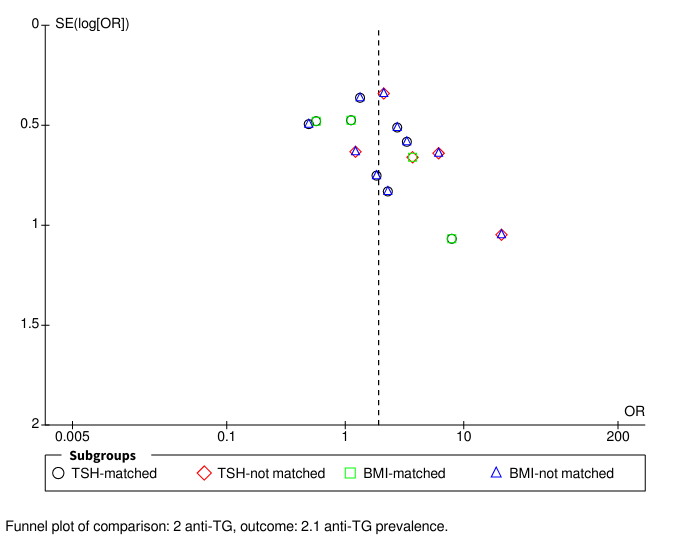

Supplement: Supplementary file 1 [file ijms-26-07525-s001.zip › Supplementary material S5 – sensitivity analyses anti-TG prevalence/SUPPLEMENTARY MATERIAL S5AE.png]

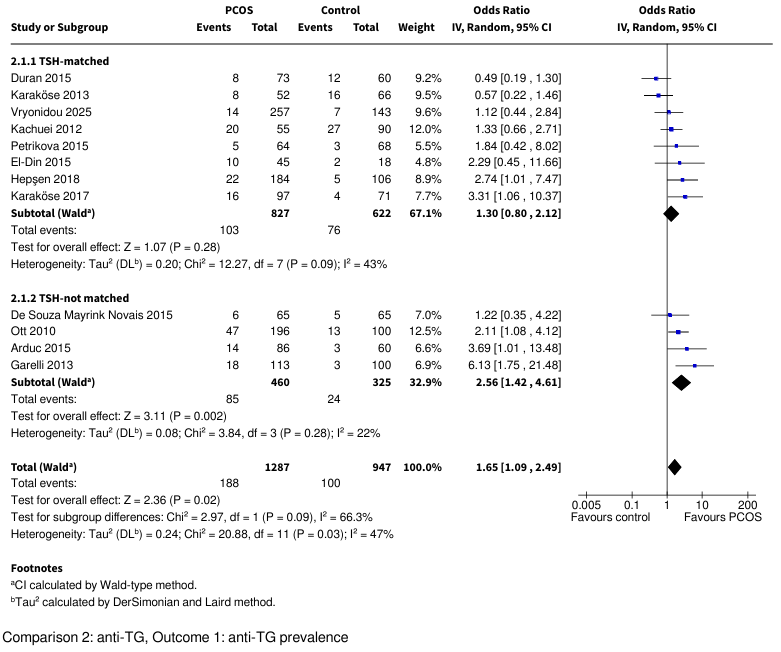

Supplement: Supplementary file 1 [file ijms-26-07525-s001.zip › Supplementary material S5 – sensitivity analyses anti-TG prevalence/SUPPLEMENTARY MATERIAL S5AF.png]

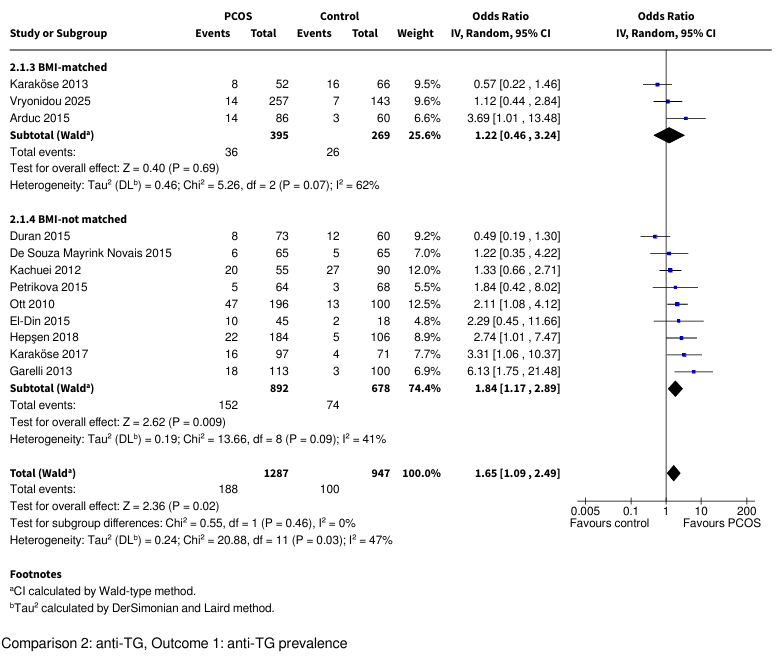

Supplement: Supplementary file 1 [file ijms-26-07525-s001.zip › Supplementary material S5 – sensitivity analyses anti-TG prevalence/SUPPLEMENTARY MATERIAL S5AG.png]

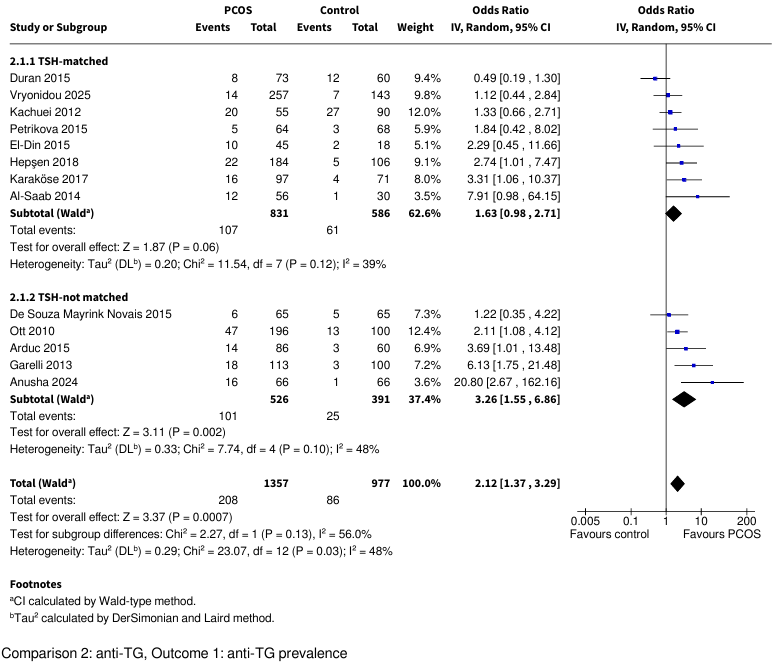

Supplement: Supplementary file 1 [file ijms-26-07525-s001.zip › Supplementary material S5 – sensitivity analyses anti-TG prevalence/SUPPLEMENTARY MATERIAL S5B.png]

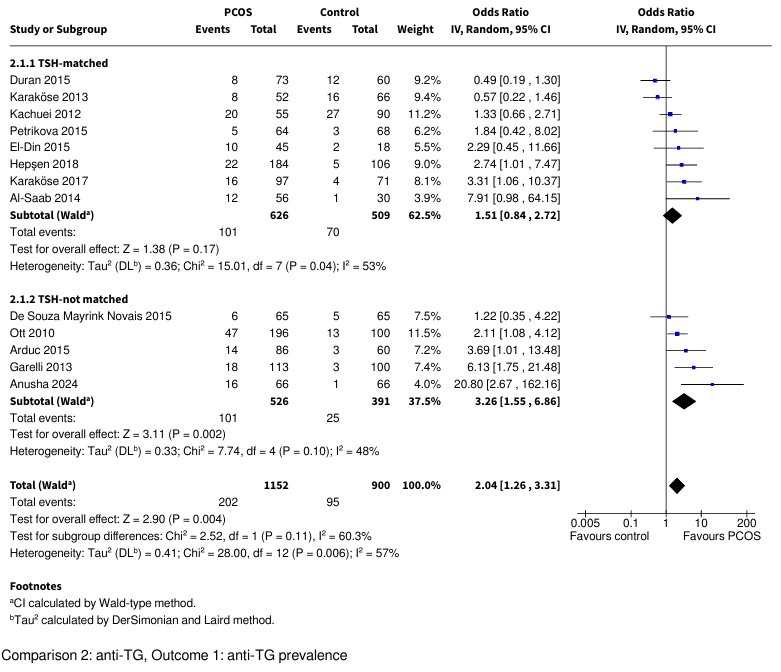

Supplement: Supplementary file 1 [file ijms-26-07525-s001.zip › Supplementary material S5 – sensitivity analyses anti-TG prevalence/SUPPLEMENTARY MATERIAL S5C.png]

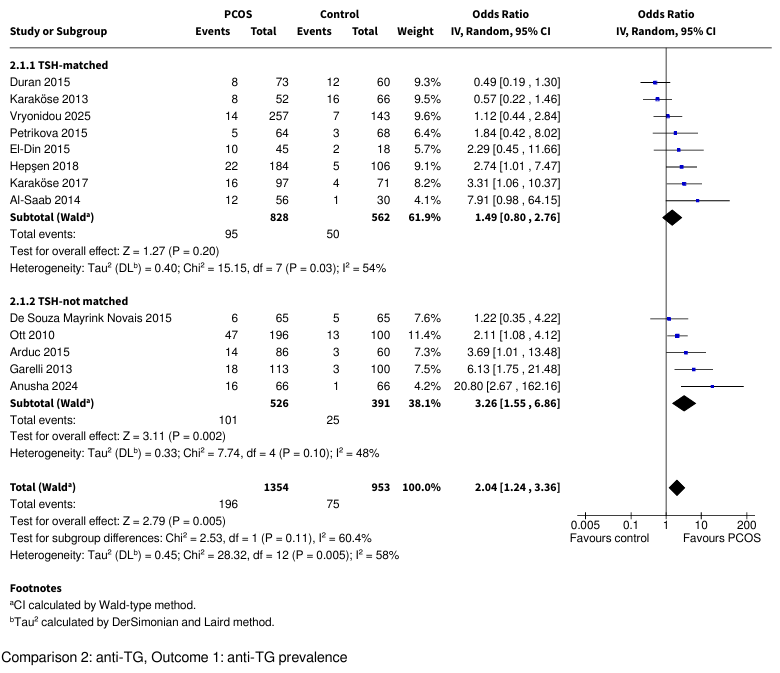

Supplement: Supplementary file 1 [file ijms-26-07525-s001.zip › Supplementary material S5 – sensitivity analyses anti-TG prevalence/SUPPLEMENTARY MATERIAL S5D.png]

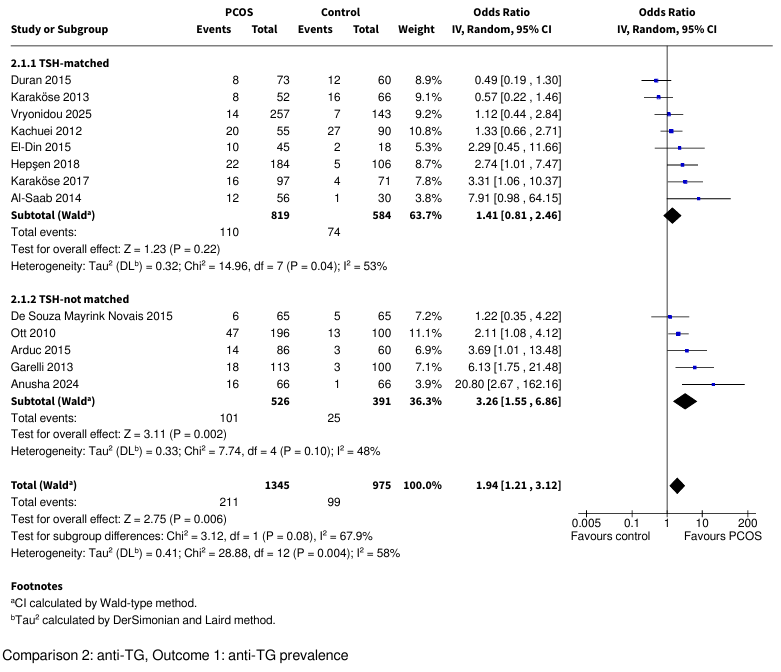

Supplement: Supplementary file 1 [file ijms-26-07525-s001.zip › Supplementary material S5 – sensitivity analyses anti-TG prevalence/SUPPLEMENTARY MATERIAL S5E.png]

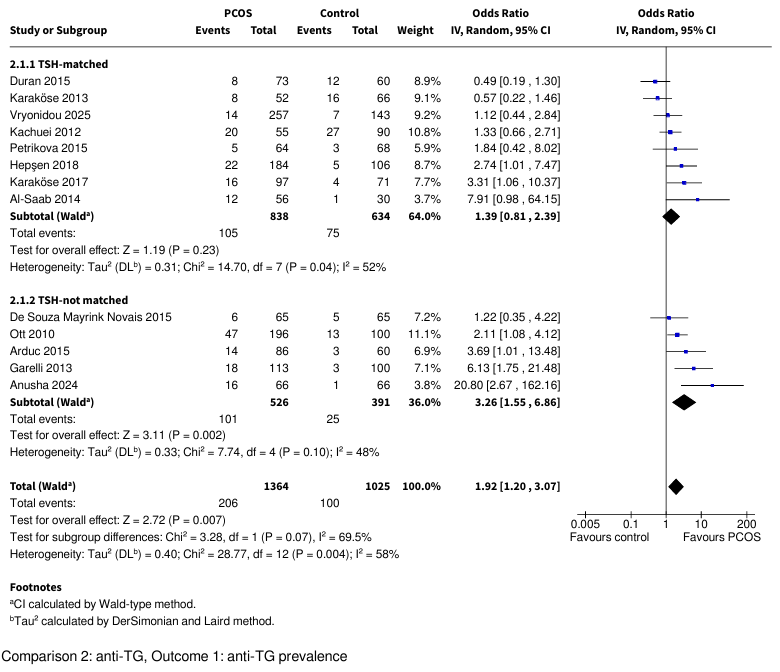

Supplement: Supplementary file 1 [file ijms-26-07525-s001.zip › Supplementary material S5 – sensitivity analyses anti-TG prevalence/SUPPLEMENTARY MATERIAL S5F.png]

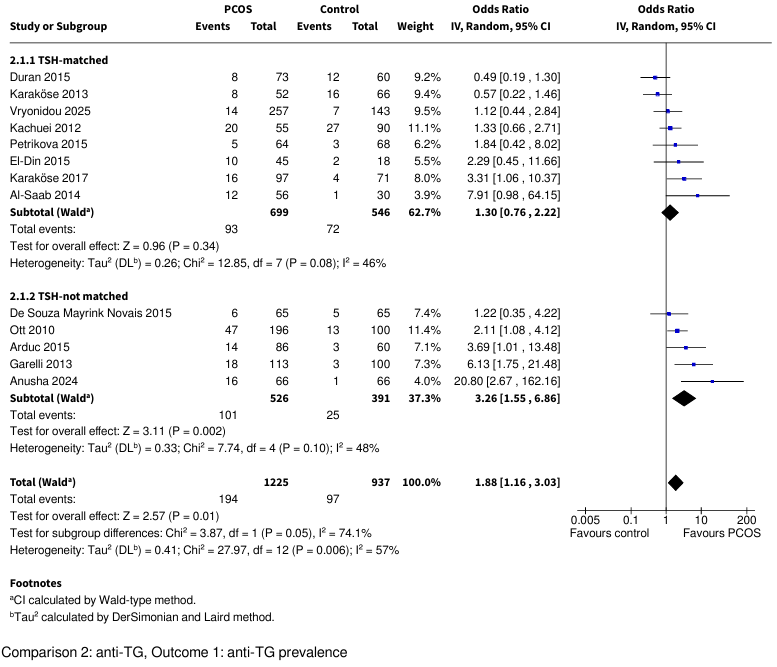

Supplement: Supplementary file 1 [file ijms-26-07525-s001.zip › Supplementary material S5 – sensitivity analyses anti-TG prevalence/SUPPLEMENTARY MATERIAL S5G.png]

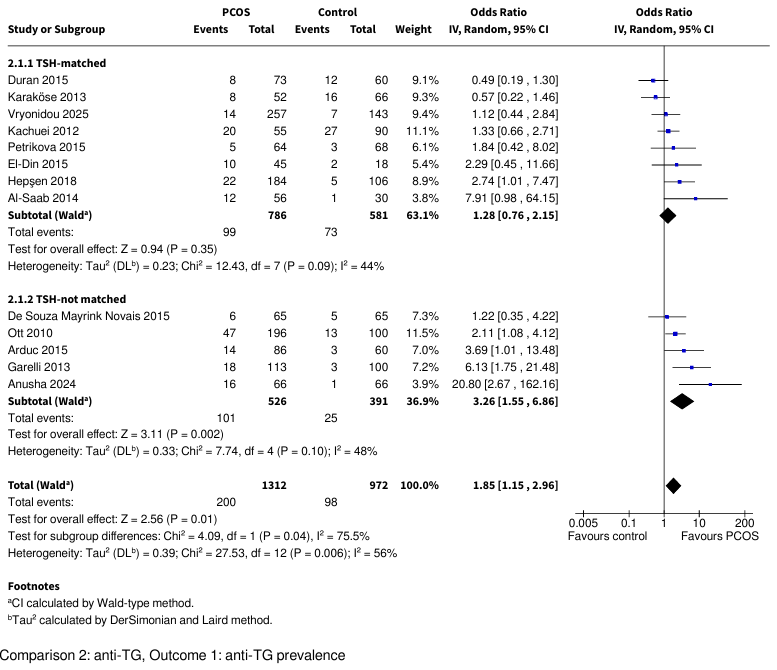

Supplement: Supplementary file 1 [file ijms-26-07525-s001.zip › Supplementary material S5 – sensitivity analyses anti-TG prevalence/SUPPLEMENTARY MATERIAL S5H.png]

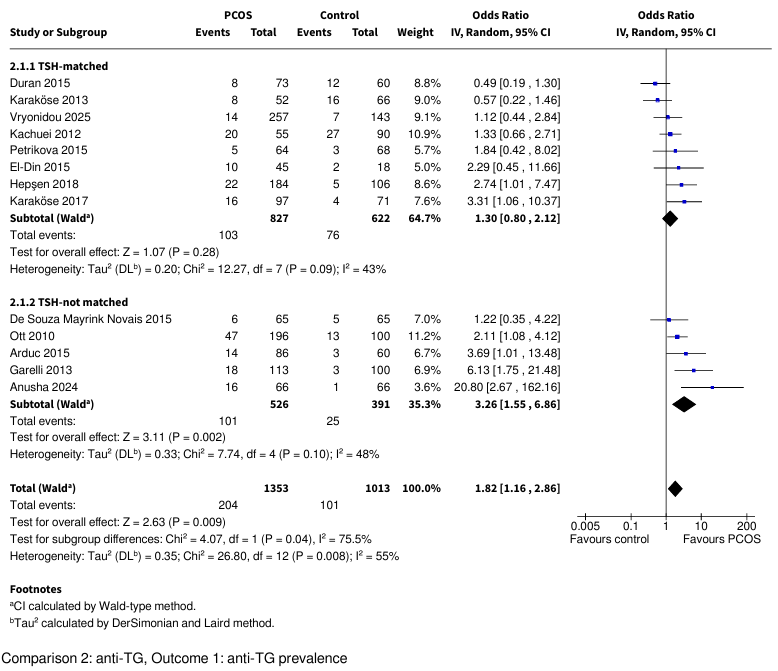

Supplement: Supplementary file 1 [file ijms-26-07525-s001.zip › Supplementary material S5 – sensitivity analyses anti-TG prevalence/SUPPLEMENTARY MATERIAL S5I.png]

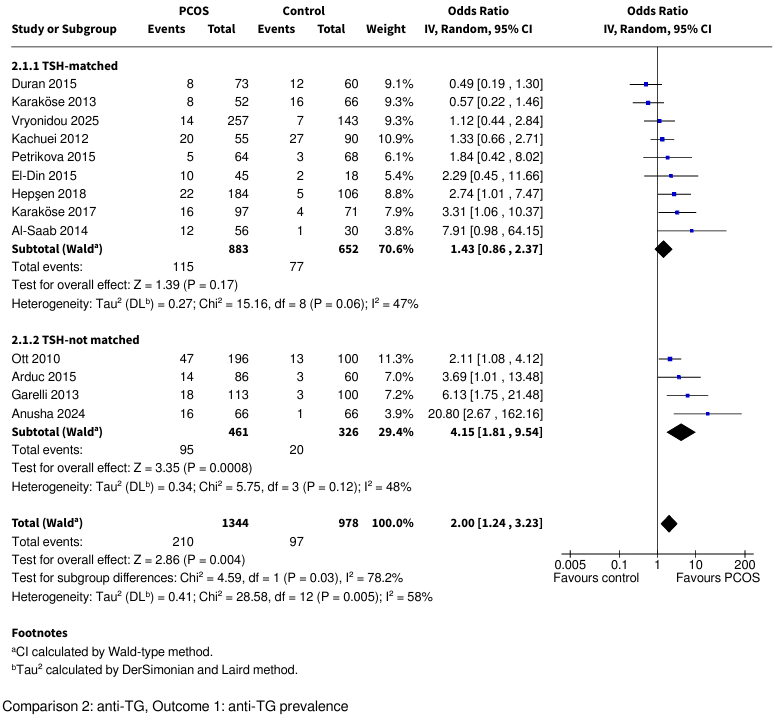

Supplement: Supplementary file 1 [file ijms-26-07525-s001.zip › Supplementary material S5 – sensitivity analyses anti-TG prevalence/SUPPLEMENTARY MATERIAL S5J.png]

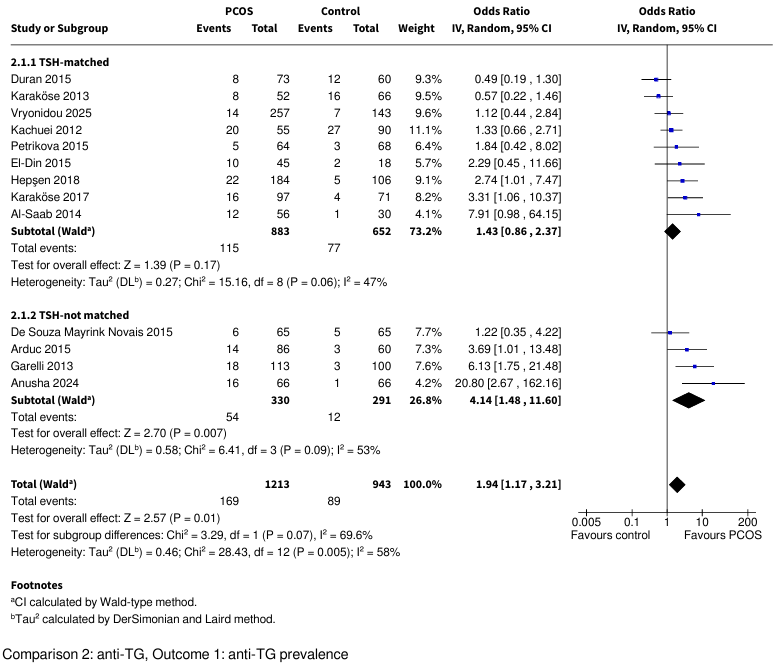

Supplement: Supplementary file 1 [file ijms-26-07525-s001.zip › Supplementary material S5 – sensitivity analyses anti-TG prevalence/SUPPLEMENTARY MATERIAL S5K.png]

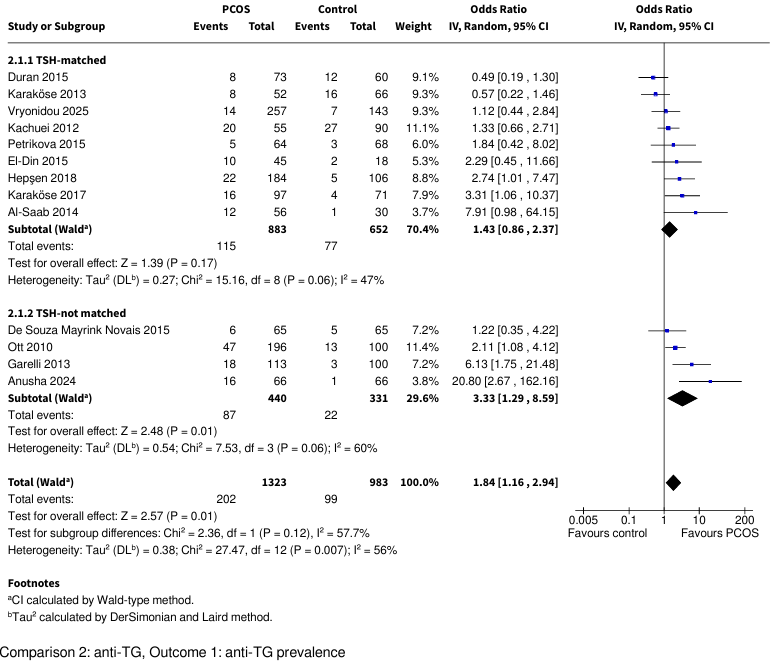

Supplement: Supplementary file 1 [file ijms-26-07525-s001.zip › Supplementary material S5 – sensitivity analyses anti-TG prevalence/SUPPLEMENTARY MATERIAL S5L.png]

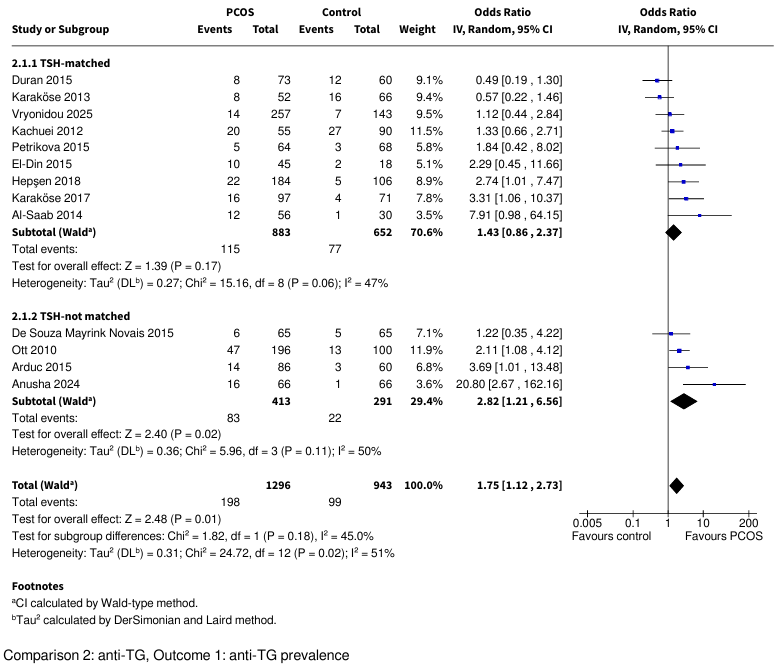

Supplement: Supplementary file 1 [file ijms-26-07525-s001.zip › Supplementary material S5 – sensitivity analyses anti-TG prevalence/SUPPLEMENTARY MATERIAL S5M.png]

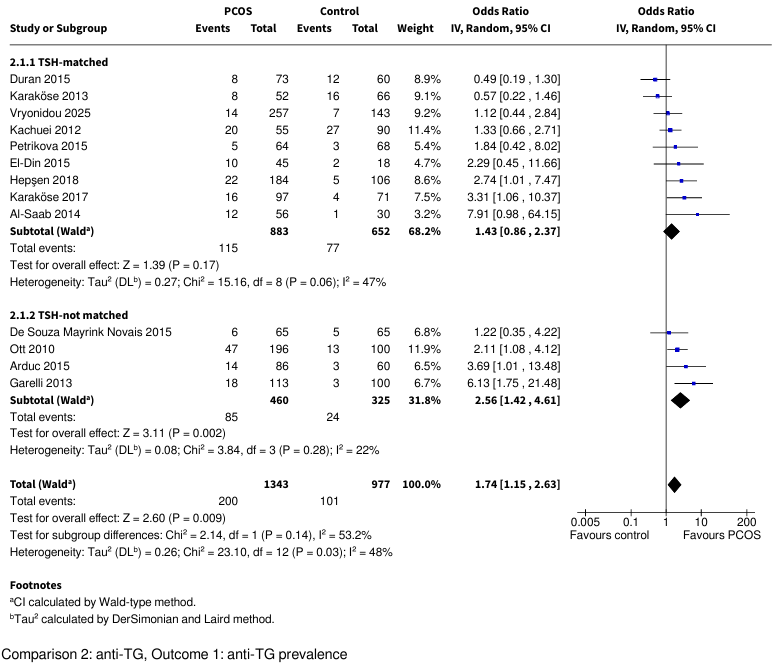

Supplement: Supplementary file 1 [file ijms-26-07525-s001.zip › Supplementary material S5 – sensitivity analyses anti-TG prevalence/SUPPLEMENTARY MATERIAL S5N.png]

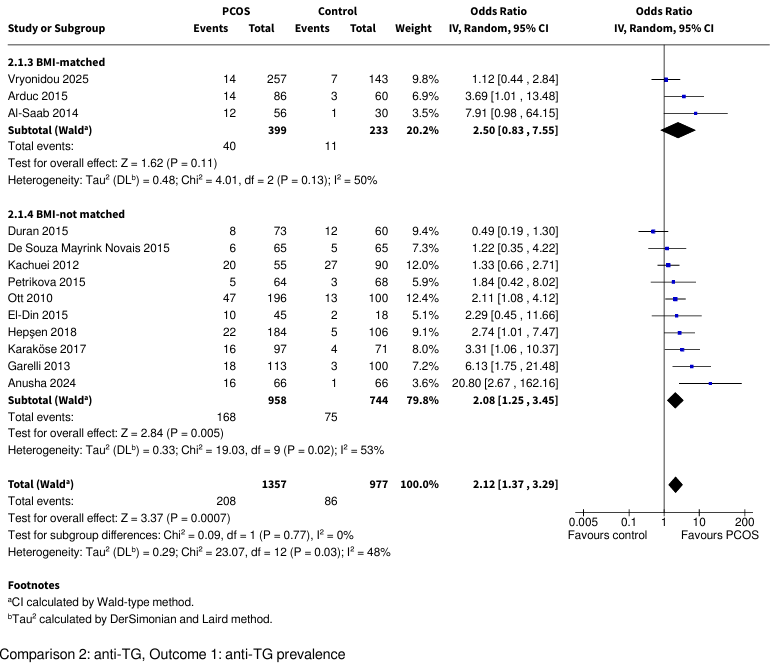

Supplement: Supplementary file 1 [file ijms-26-07525-s001.zip › Supplementary material S5 – sensitivity analyses anti-TG prevalence/SUPPLEMENTARY MATERIAL S5O.png]

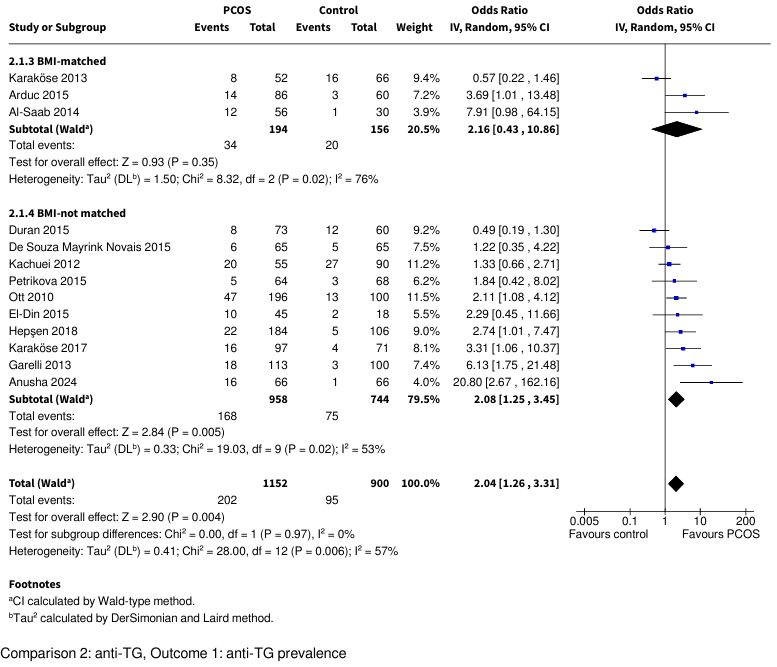

Supplement: Supplementary file 1 [file ijms-26-07525-s001.zip › Supplementary material S5 – sensitivity analyses anti-TG prevalence/SUPPLEMENTARY MATERIAL S5P.png]

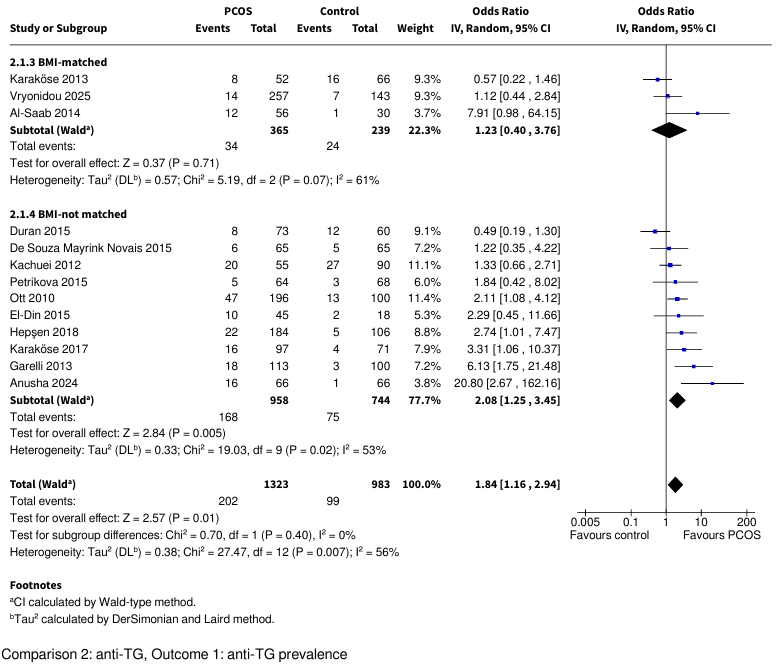

Supplement: Supplementary file 1 [file ijms-26-07525-s001.zip › Supplementary material S5 – sensitivity analyses anti-TG prevalence/SUPPLEMENTARY MATERIAL S5Q.png]

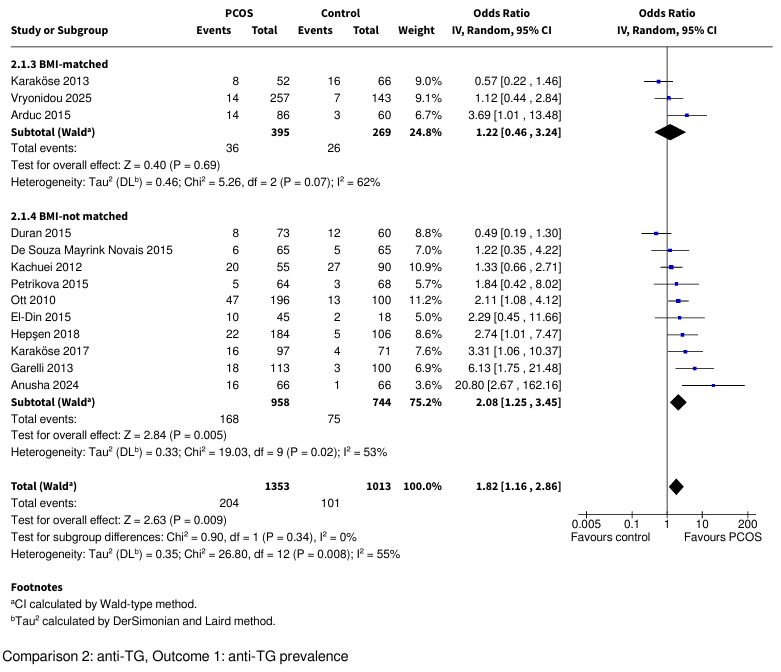

Supplement: Supplementary file 1 [file ijms-26-07525-s001.zip › Supplementary material S5 – sensitivity analyses anti-TG prevalence/SUPPLEMENTARY MATERIAL S5R.png]

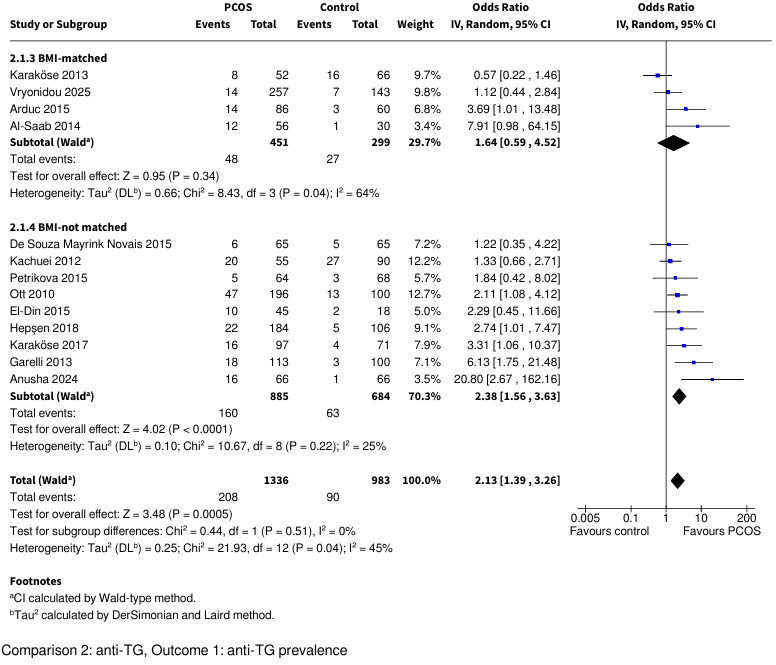

Supplement: Supplementary file 1 [file ijms-26-07525-s001.zip › Supplementary material S5 – sensitivity analyses anti-TG prevalence/SUPPLEMENTARY MATERIAL S5S.png]

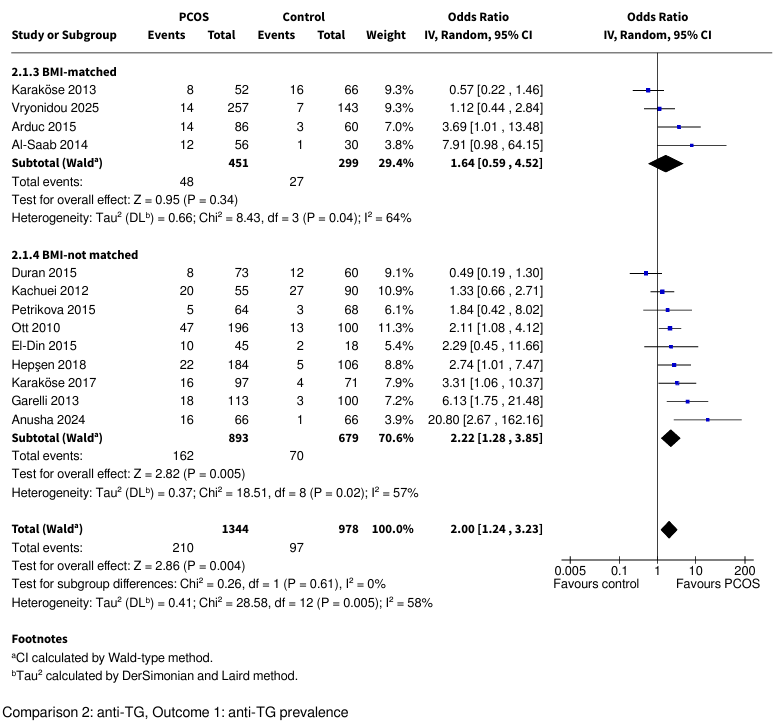

Supplement: Supplementary file 1 [file ijms-26-07525-s001.zip › Supplementary material S5 – sensitivity analyses anti-TG prevalence/SUPPLEMENTARY MATERIAL S5T.png]

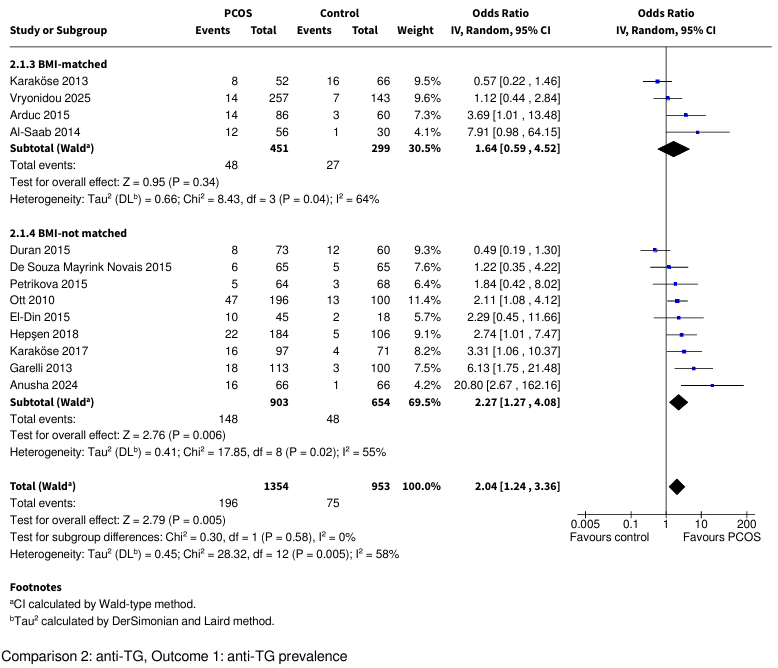

Supplement: Supplementary file 1 [file ijms-26-07525-s001.zip › Supplementary material S5 – sensitivity analyses anti-TG prevalence/SUPPLEMENTARY MATERIAL S5U.png]

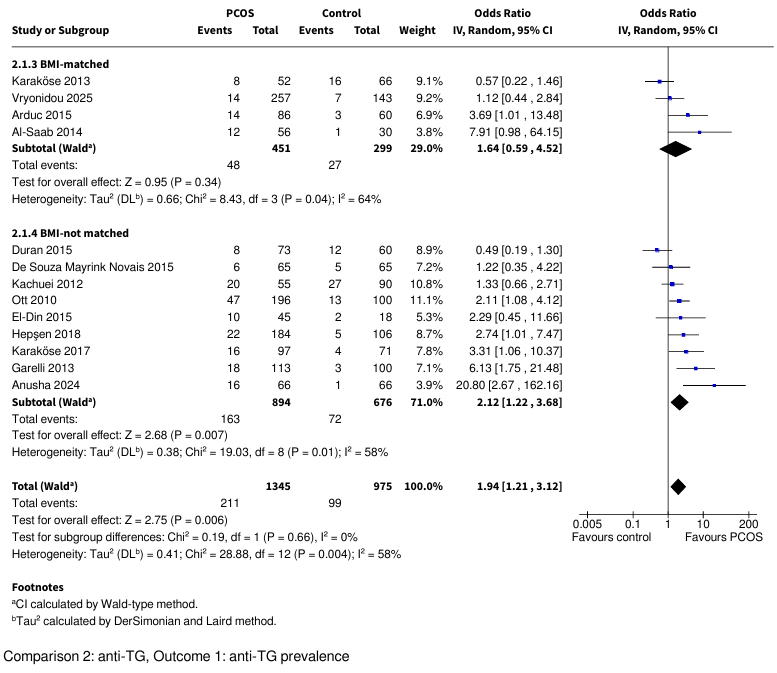

Supplement: Supplementary file 1 [file ijms-26-07525-s001.zip › Supplementary material S5 – sensitivity analyses anti-TG prevalence/SUPPLEMENTARY MATERIAL S5V.png]

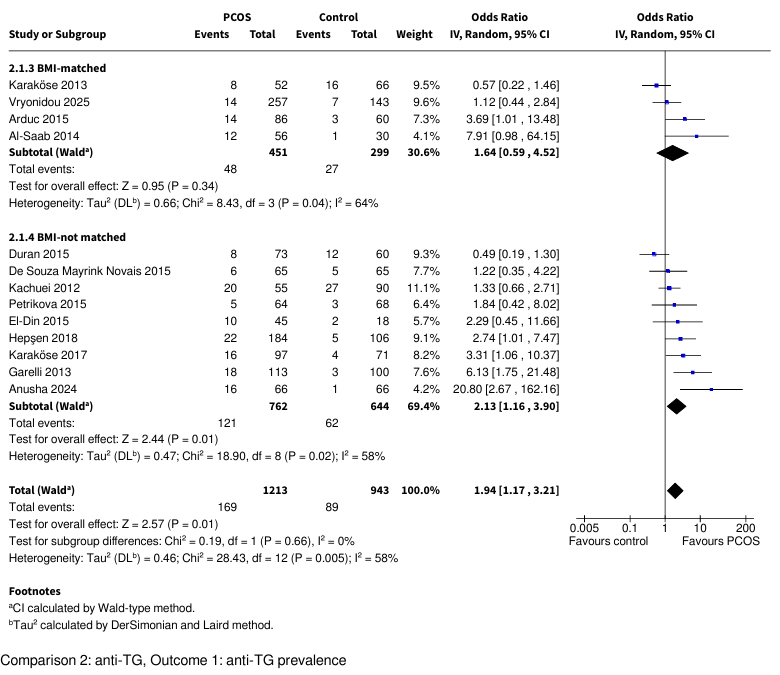

Supplement: Supplementary file 1 [file ijms-26-07525-s001.zip › Supplementary material S5 – sensitivity analyses anti-TG prevalence/SUPPLEMENTARY MATERIAL S5W.png]

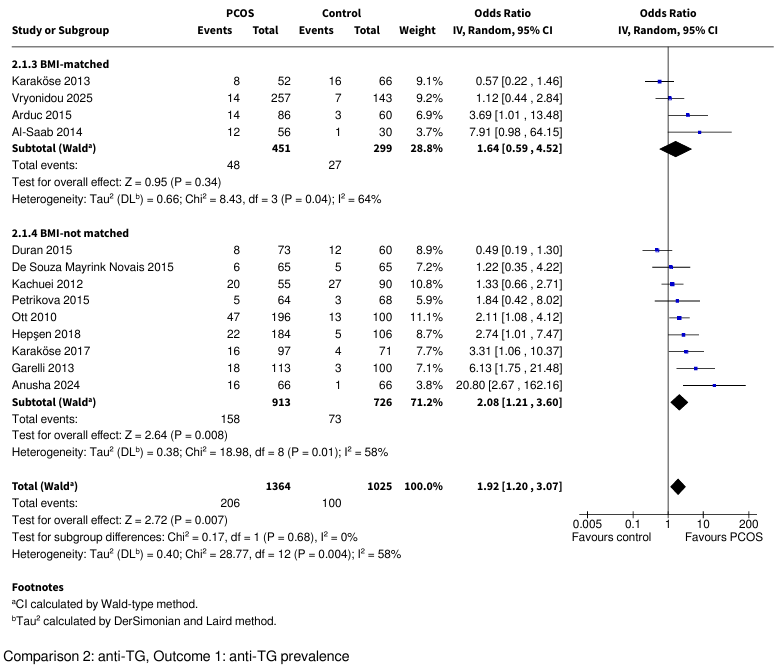

Supplement: Supplementary file 1 [file ijms-26-07525-s001.zip › Supplementary material S5 – sensitivity analyses anti-TG prevalence/SUPPLEMENTARY MATERIAL S5X.png]

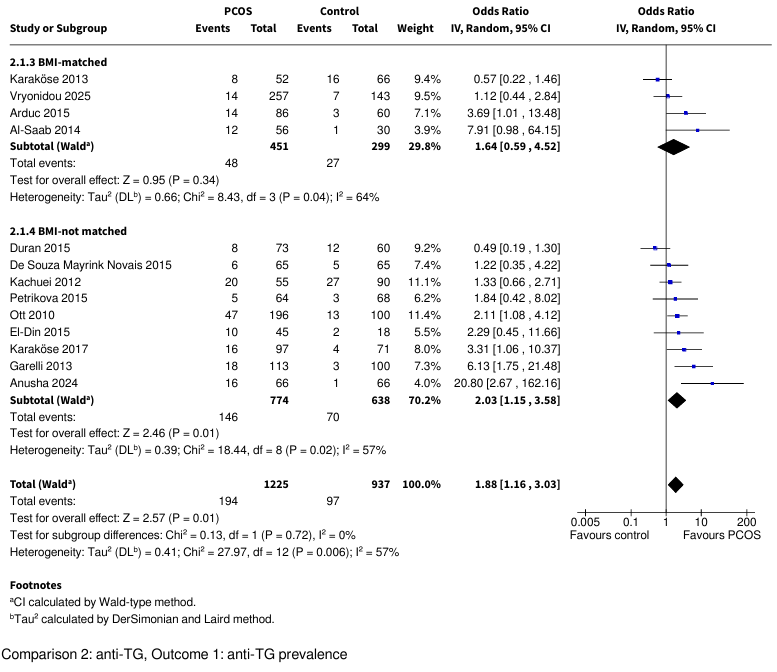

Supplement: Supplementary file 1 [file ijms-26-07525-s001.zip › Supplementary material S5 – sensitivity analyses anti-TG prevalence/SUPPLEMENTARY MATERIAL S5Y.png]

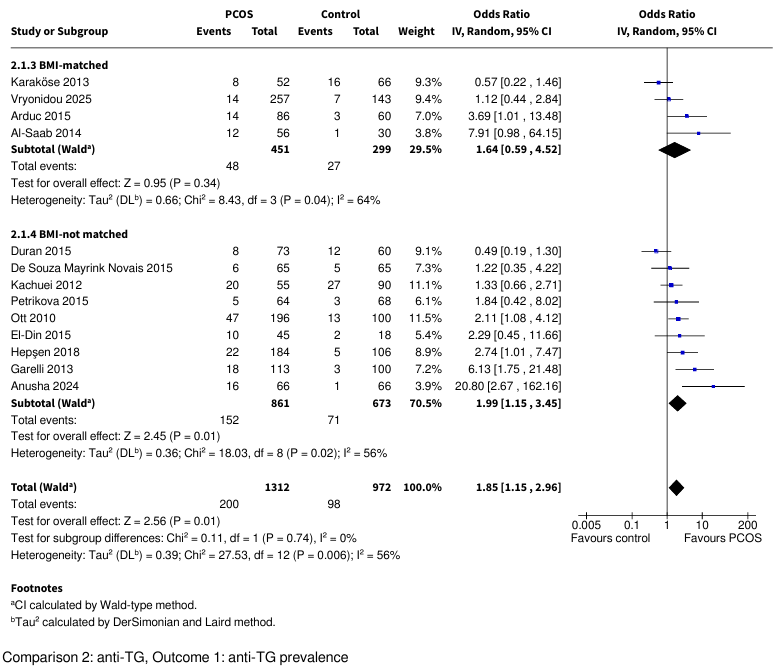

Supplement: Supplementary file 1 [file ijms-26-07525-s001.zip › Supplementary material S5 – sensitivity analyses anti-TG prevalence/SUPPLEMENTARY MATERIAL S5Z.png]

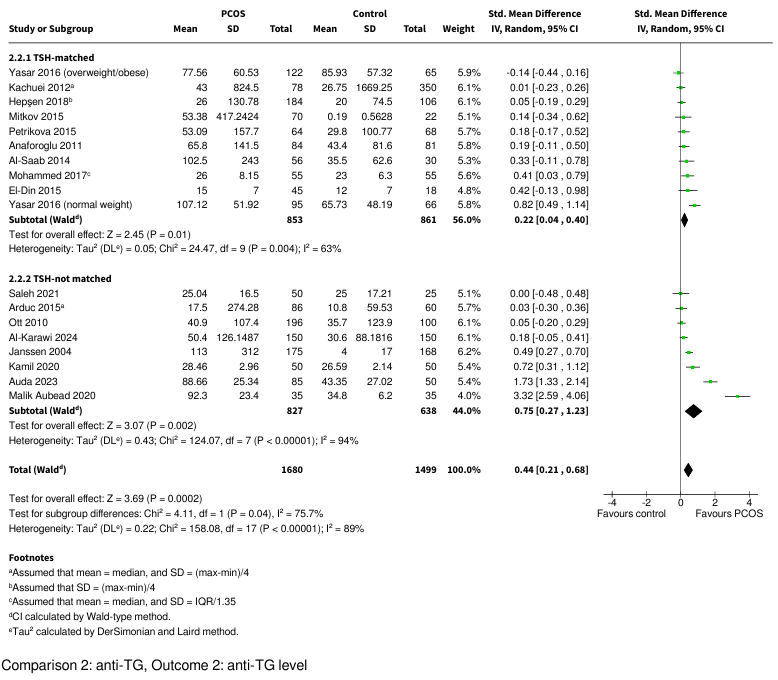

Supplement: Supplementary file 1 [file ijms-26-07525-s001.zip › Supplementary material S6 – sensitivity analyses anti-TG level/SUPPLEMENTARY MATERIAL S6A.png]

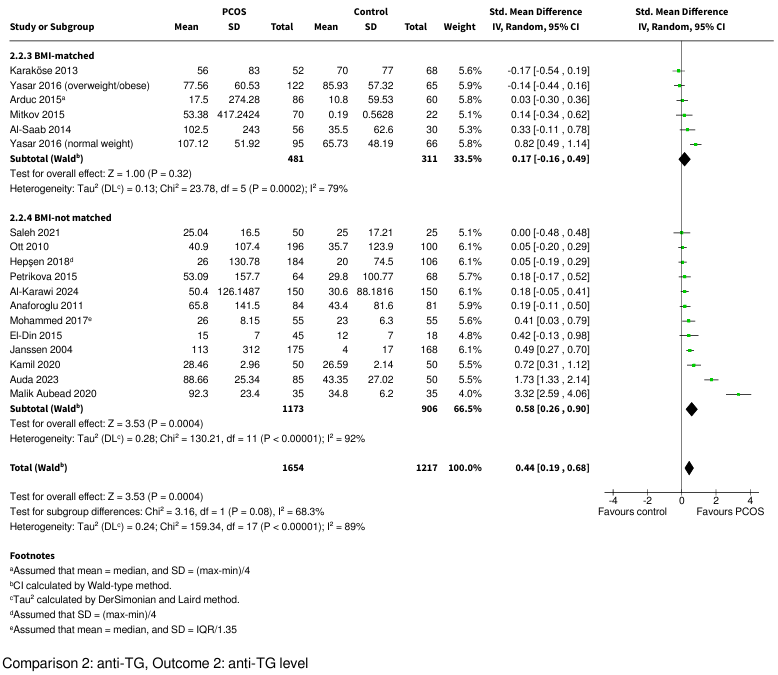

Supplement: Supplementary file 1 [file ijms-26-07525-s001.zip › Supplementary material S6 – sensitivity analyses anti-TG level/SUPPLEMENTARY MATERIAL S6AA.png]

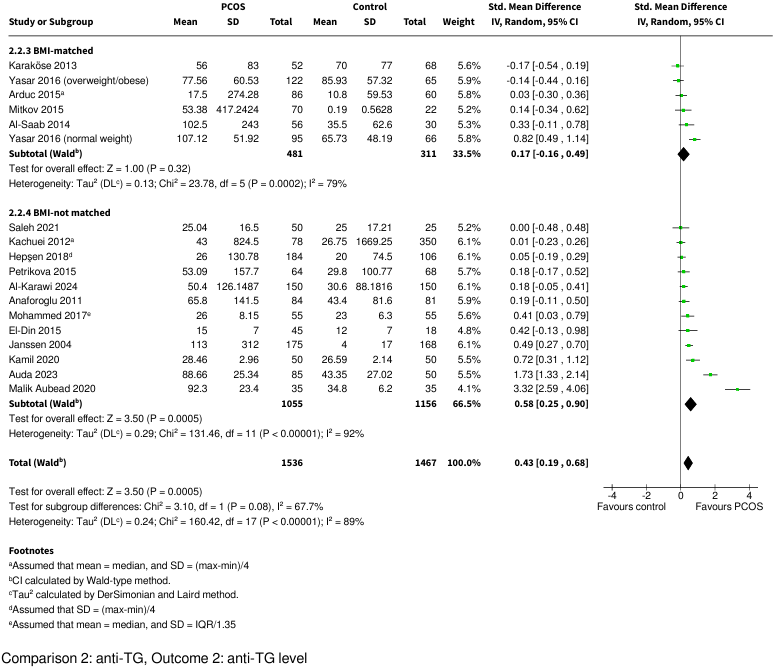

Supplement: Supplementary file 1 [file ijms-26-07525-s001.zip › Supplementary material S6 – sensitivity analyses anti-TG level/SUPPLEMENTARY MATERIAL S6AB.png]

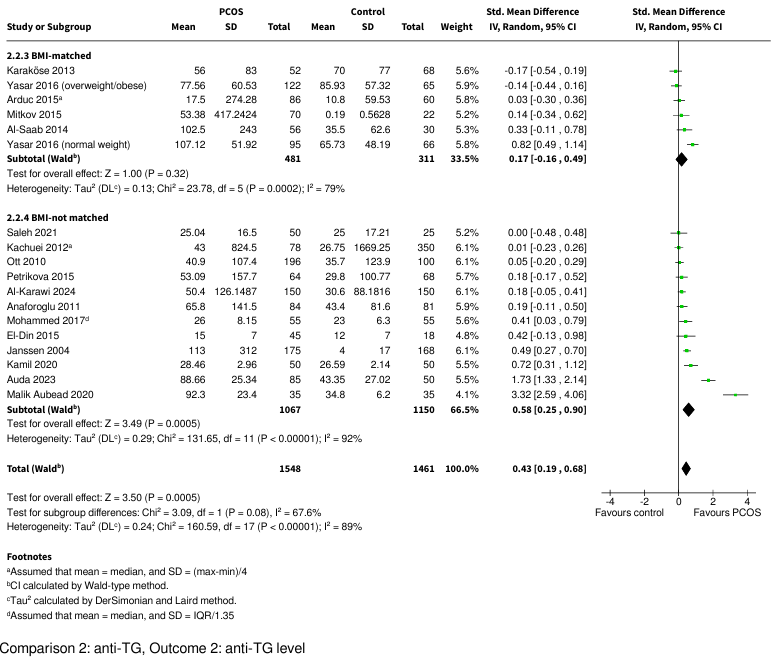

Supplement: Supplementary file 1 [file ijms-26-07525-s001.zip › Supplementary material S6 – sensitivity analyses anti-TG level/SUPPLEMENTARY MATERIAL S6AC.png]

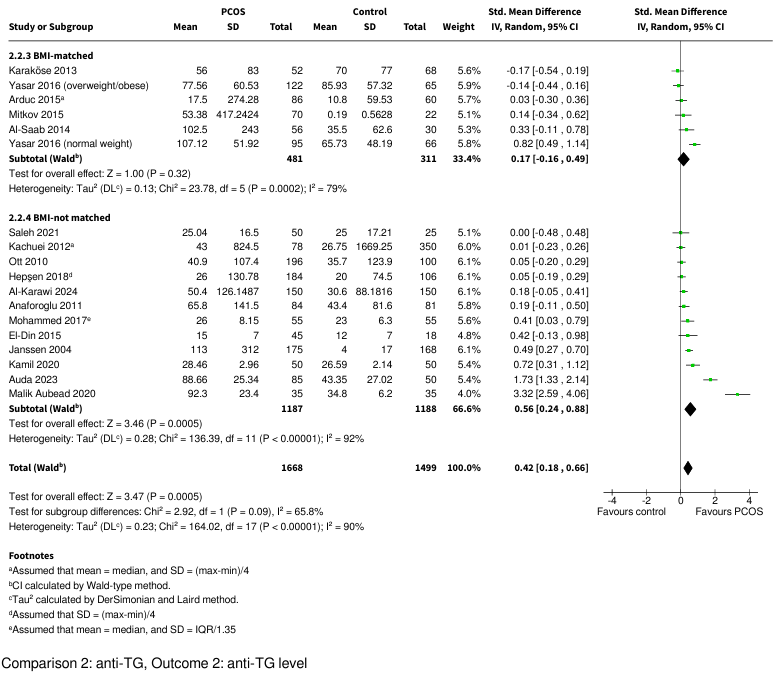

Supplement: Supplementary file 1 [file ijms-26-07525-s001.zip › Supplementary material S6 – sensitivity analyses anti-TG level/SUPPLEMENTARY MATERIAL S6AD.png]

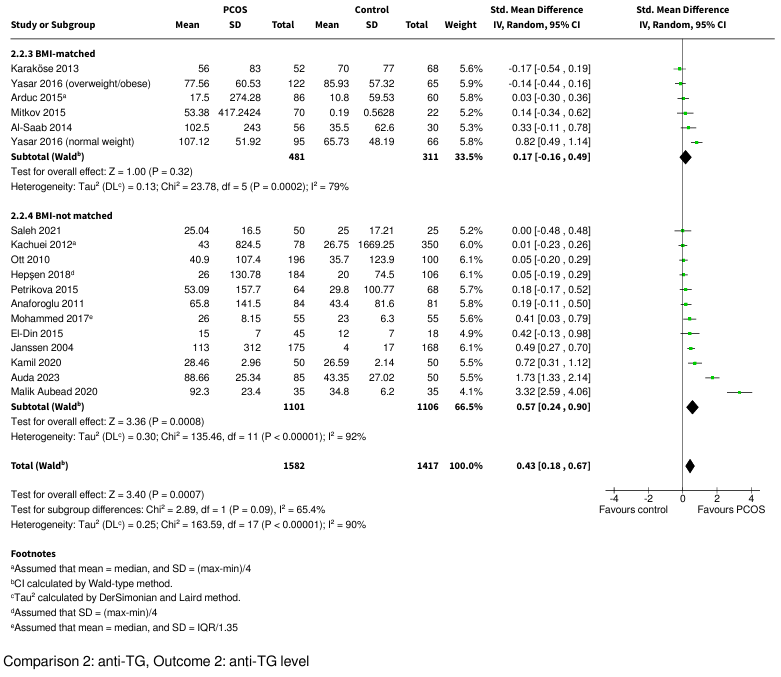

Supplement: Supplementary file 1 [file ijms-26-07525-s001.zip › Supplementary material S6 – sensitivity analyses anti-TG level/SUPPLEMENTARY MATERIAL S6AE.png]

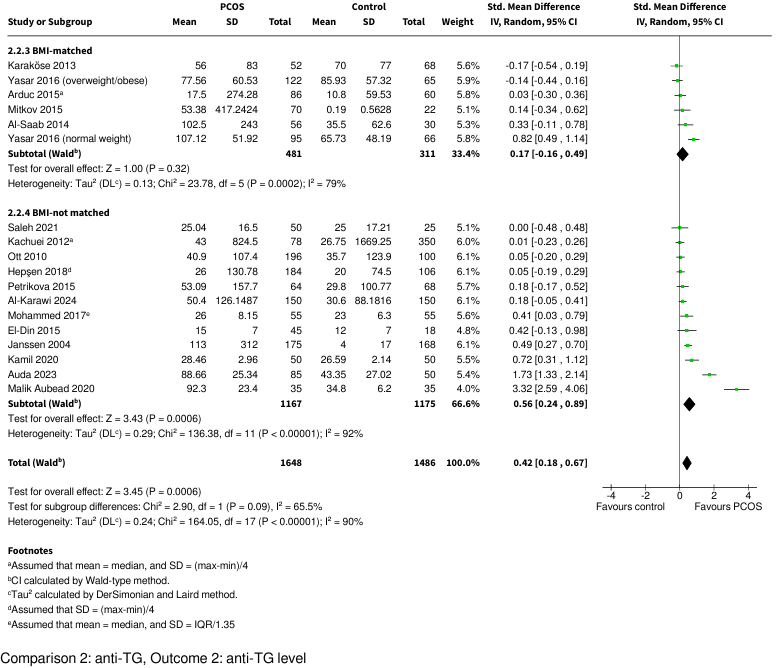

Supplement: Supplementary file 1 [file ijms-26-07525-s001.zip › Supplementary material S6 – sensitivity analyses anti-TG level/SUPPLEMENTARY MATERIAL S6AF.png]

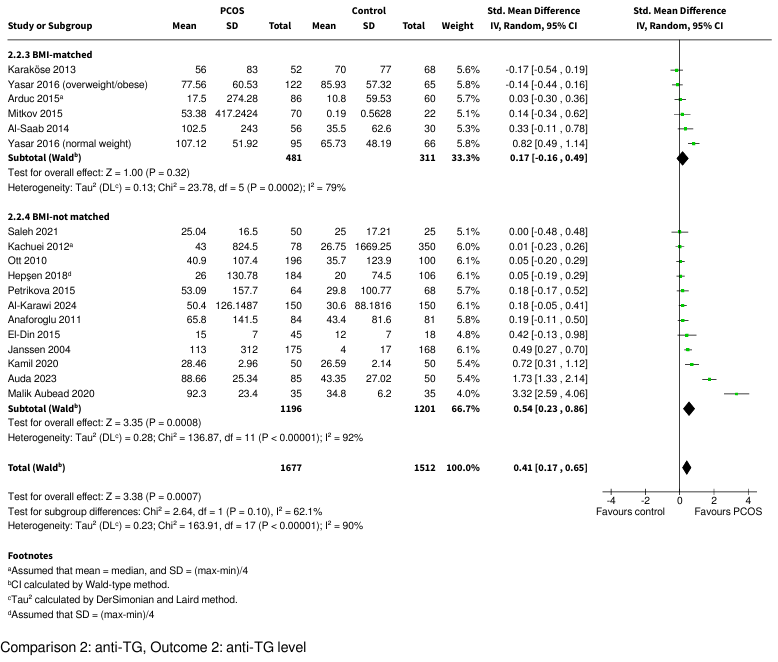

Supplement: Supplementary file 1 [file ijms-26-07525-s001.zip › Supplementary material S6 – sensitivity analyses anti-TG level/SUPPLEMENTARY MATERIAL S6AG.png]

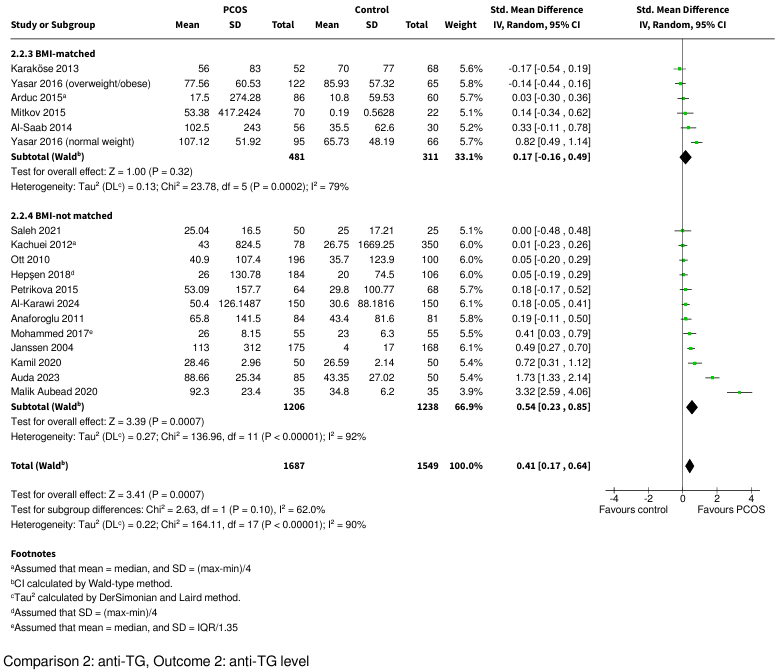

Supplement: Supplementary file 1 [file ijms-26-07525-s001.zip › Supplementary material S6 – sensitivity analyses anti-TG level/SUPPLEMENTARY MATERIAL S6AH.png]

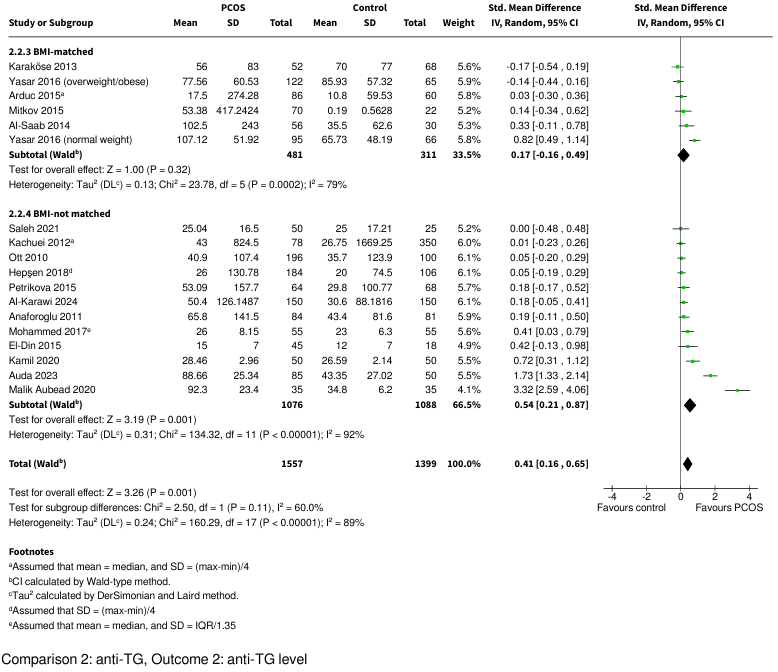

Supplement: Supplementary file 1 [file ijms-26-07525-s001.zip › Supplementary material S6 – sensitivity analyses anti-TG level/SUPPLEMENTARY MATERIAL S6AI.png]

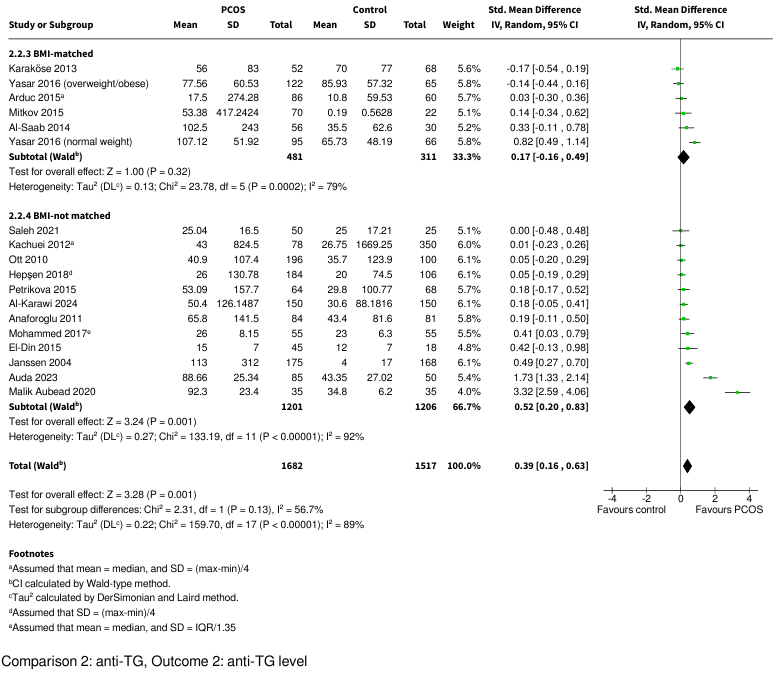

Supplement: Supplementary file 1 [file ijms-26-07525-s001.zip › Supplementary material S6 – sensitivity analyses anti-TG level/SUPPLEMENTARY MATERIAL S6AJ.png]

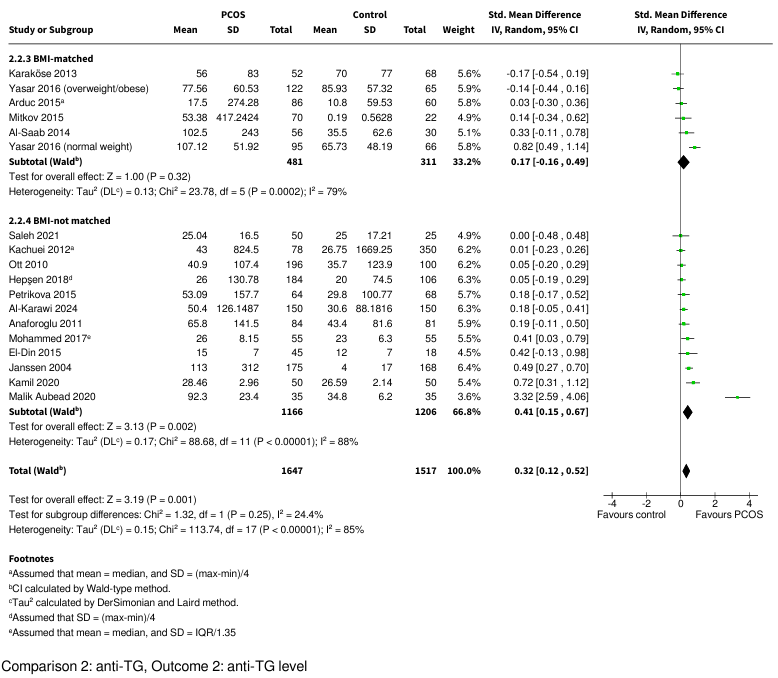

Supplement: Supplementary file 1 [file ijms-26-07525-s001.zip › Supplementary material S6 – sensitivity analyses anti-TG level/SUPPLEMENTARY MATERIAL S6AK.png]

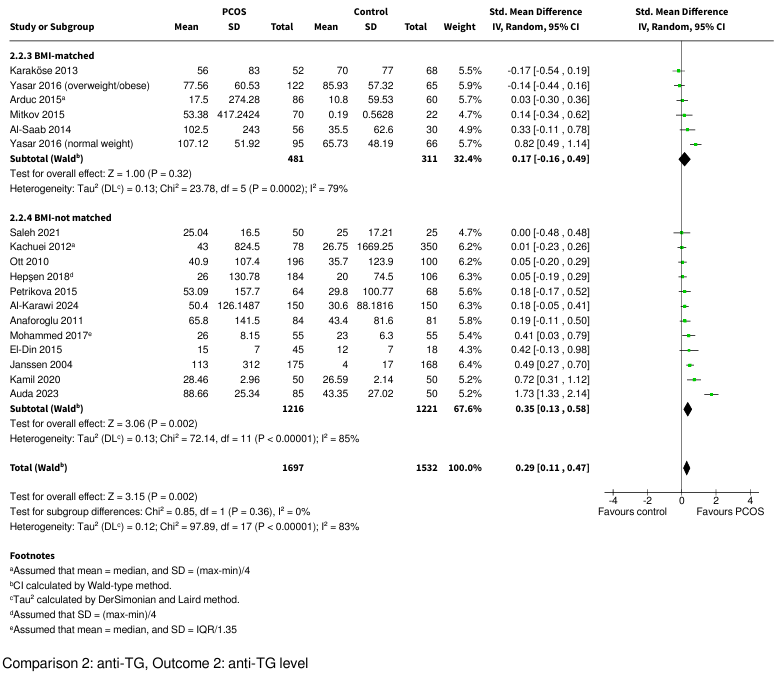

Supplement: Supplementary file 1 [file ijms-26-07525-s001.zip › Supplementary material S6 – sensitivity analyses anti-TG level/SUPPLEMENTARY MATERIAL S6AL.png]

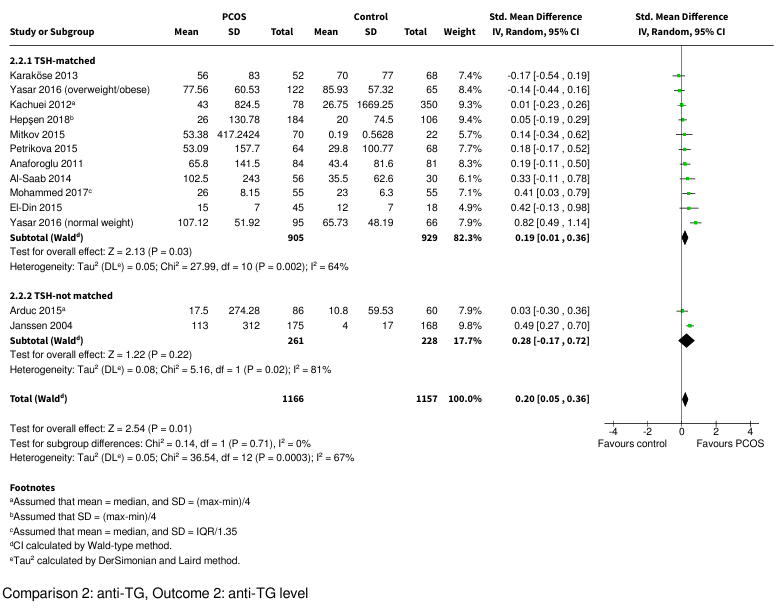

Supplement: Supplementary file 1 [file ijms-26-07525-s001.zip › Supplementary material S6 – sensitivity analyses anti-TG level/SUPPLEMENTARY MATERIAL S6AM.png]

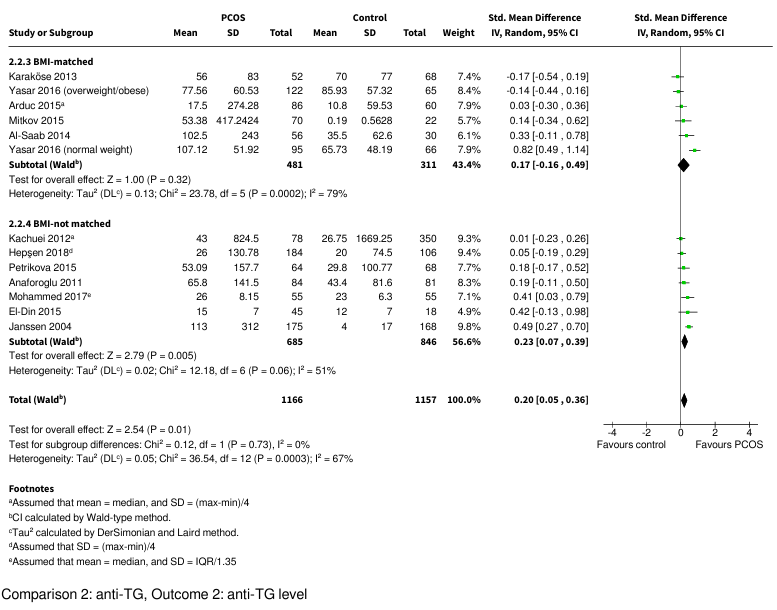

Supplement: Supplementary file 1 [file ijms-26-07525-s001.zip › Supplementary material S6 – sensitivity analyses anti-TG level/SUPPLEMENTARY MATERIAL S6AN.png]

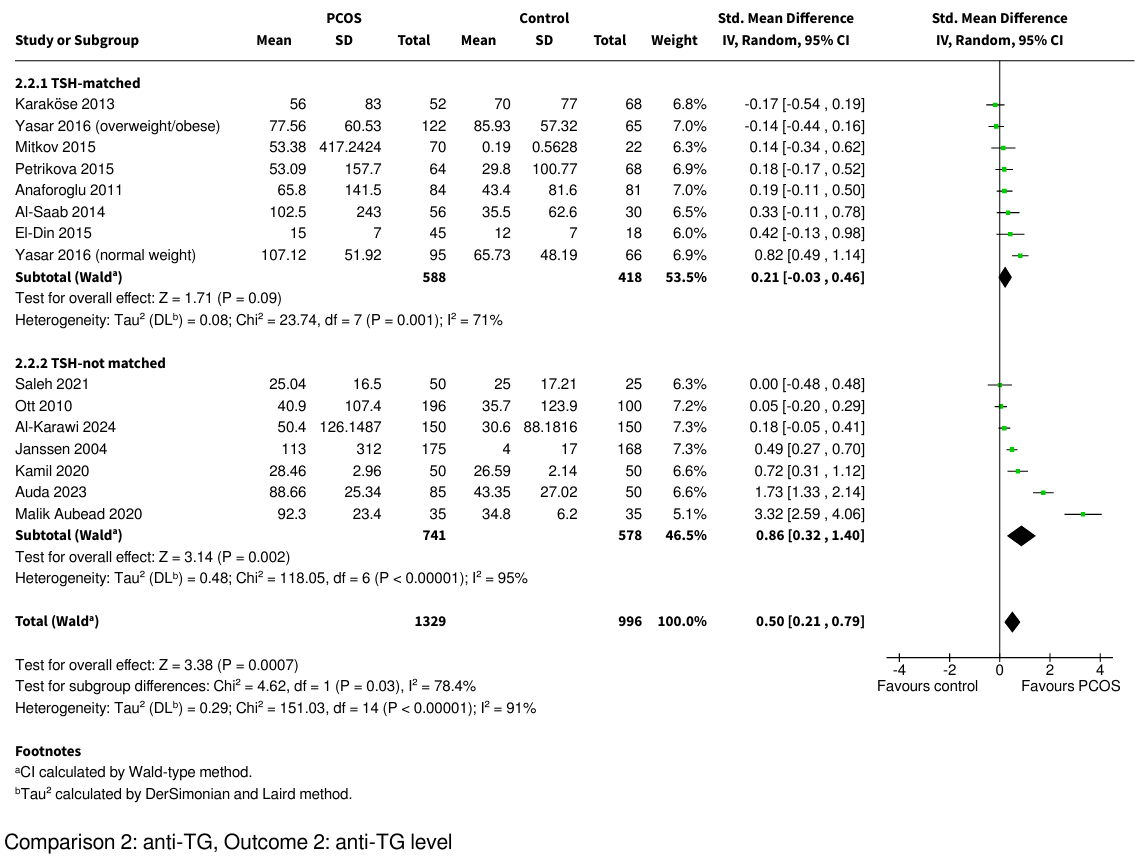

Supplement: Supplementary file 1 [file ijms-26-07525-s001.zip › Supplementary material S6 – sensitivity analyses anti-TG level/SUPPLEMENTARY MATERIAL S6AO.png]

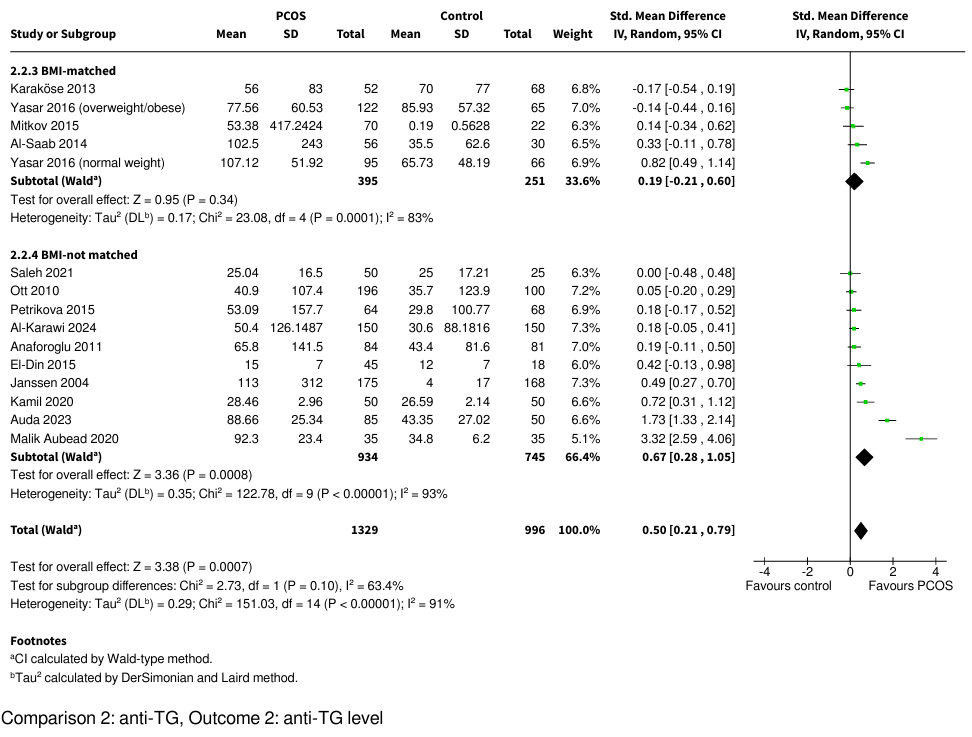

Supplement: Supplementary file 1 [file ijms-26-07525-s001.zip › Supplementary material S6 – sensitivity analyses anti-TG level/SUPPLEMENTARY MATERIAL S6AP.png]

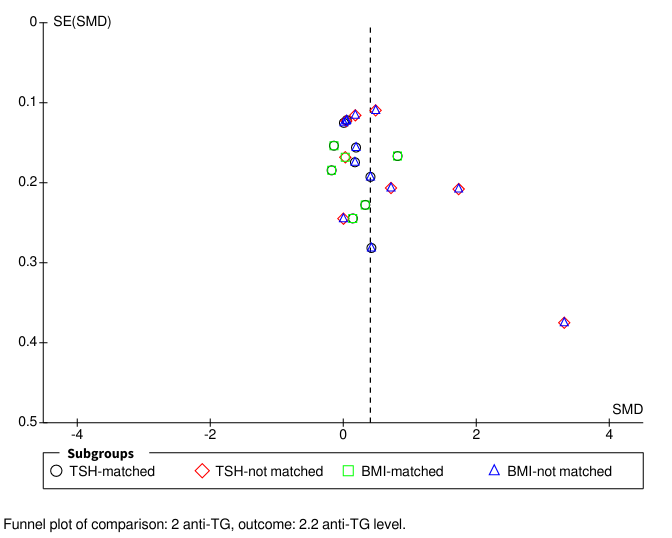

Supplement: Supplementary file 1 [file ijms-26-07525-s001.zip › Supplementary material S6 – sensitivity analyses anti-TG level/SUPPLEMENTARY MATERIAL S6AR.png]

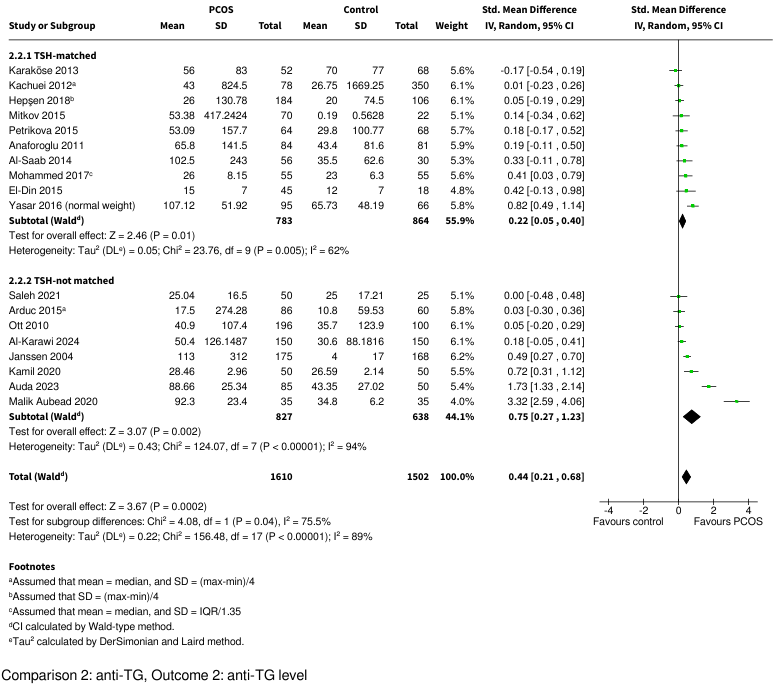

Supplement: Supplementary file 1 [file ijms-26-07525-s001.zip › Supplementary material S6 – sensitivity analyses anti-TG level/SUPPLEMENTARY MATERIAL S6B.png]

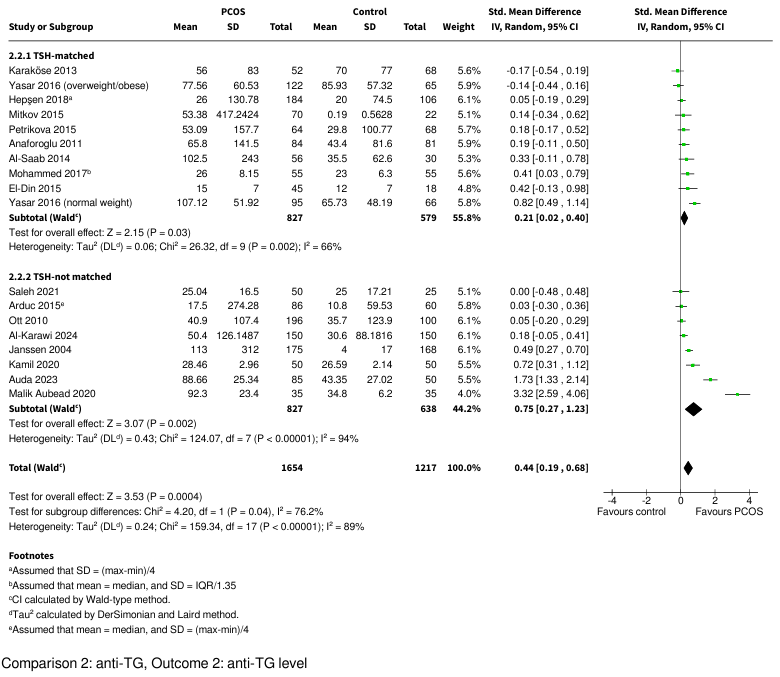

Supplement: Supplementary file 1 [file ijms-26-07525-s001.zip › Supplementary material S6 – sensitivity analyses anti-TG level/SUPPLEMENTARY MATERIAL S6C.png]

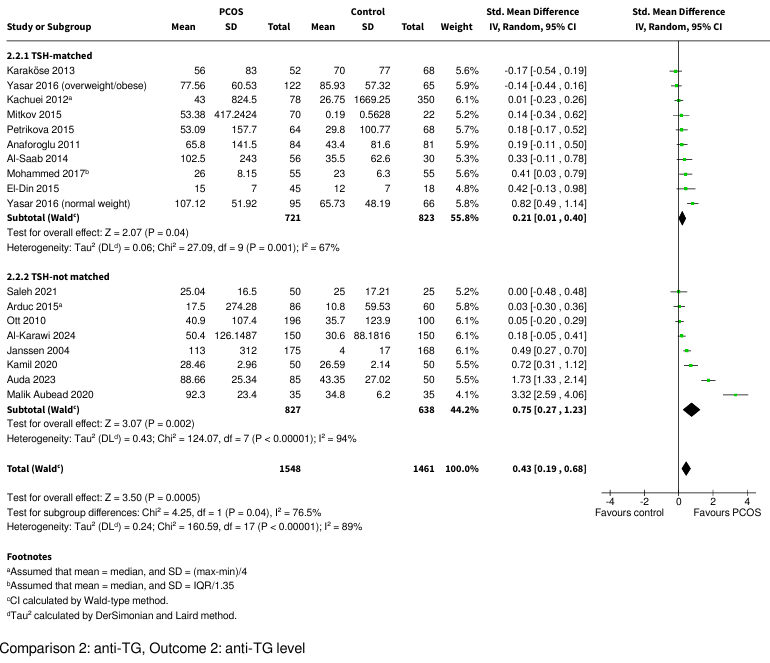

Supplement: Supplementary file 1 [file ijms-26-07525-s001.zip › Supplementary material S6 – sensitivity analyses anti-TG level/SUPPLEMENTARY MATERIAL S6D.png]

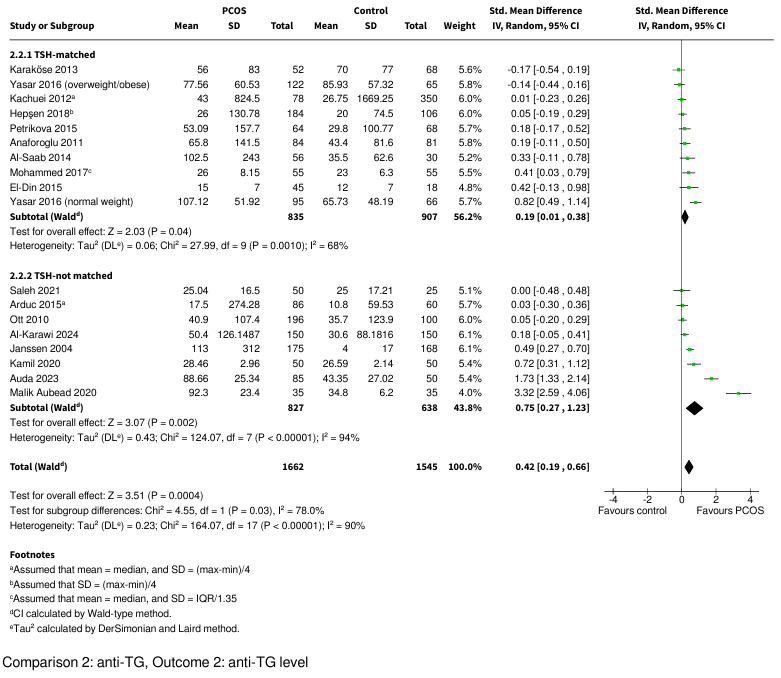

Supplement: Supplementary file 1 [file ijms-26-07525-s001.zip › Supplementary material S6 – sensitivity analyses anti-TG level/SUPPLEMENTARY MATERIAL S6E.png]

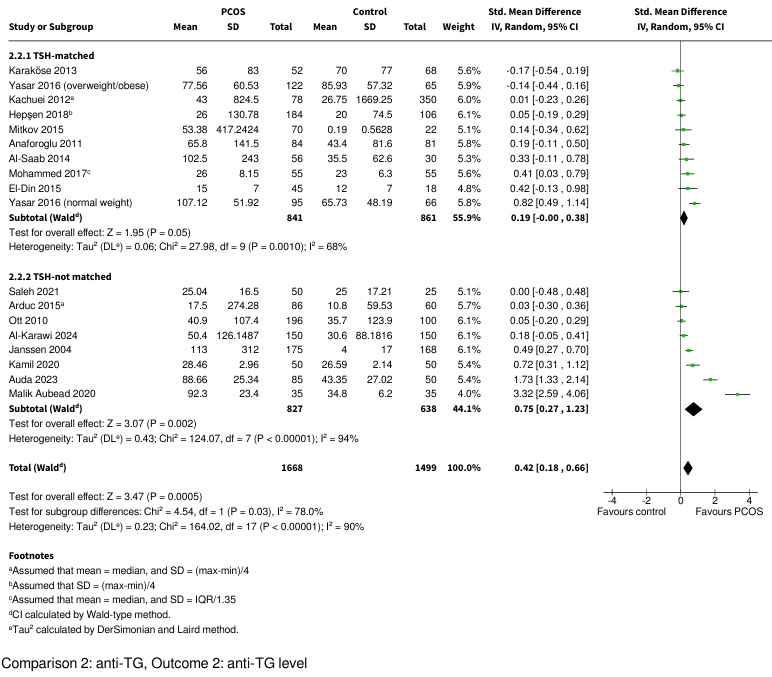

Supplement: Supplementary file 1 [file ijms-26-07525-s001.zip › Supplementary material S6 – sensitivity analyses anti-TG level/SUPPLEMENTARY MATERIAL S6F.png]

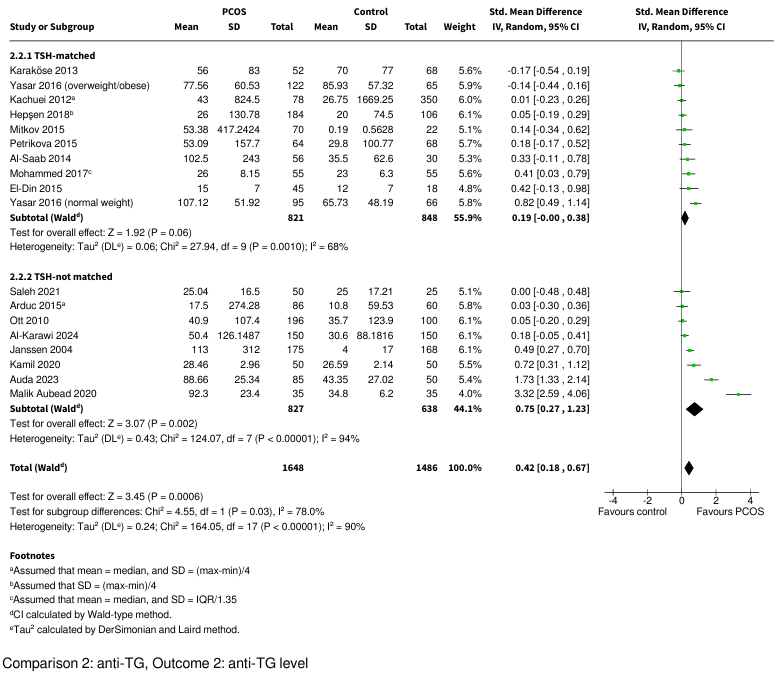

Supplement: Supplementary file 1 [file ijms-26-07525-s001.zip › Supplementary material S6 – sensitivity analyses anti-TG level/SUPPLEMENTARY MATERIAL S6G.png]

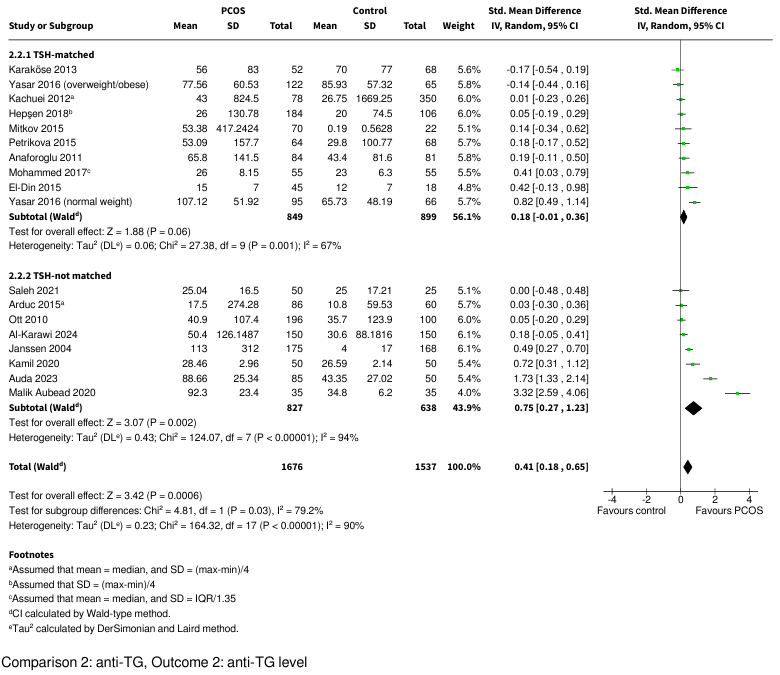

Supplement: Supplementary file 1 [file ijms-26-07525-s001.zip › Supplementary material S6 – sensitivity analyses anti-TG level/SUPPLEMENTARY MATERIAL S6H.png]

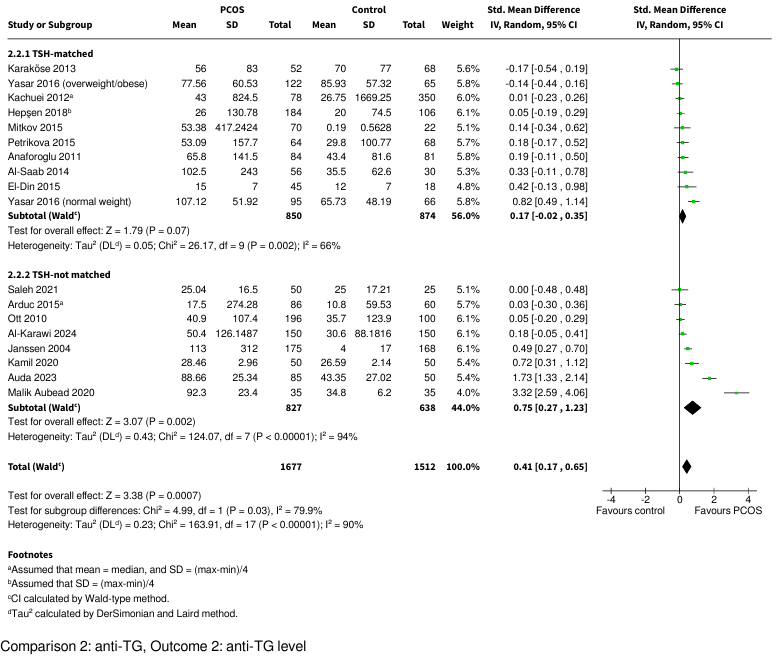

Supplement: Supplementary file 1 [file ijms-26-07525-s001.zip › Supplementary material S6 – sensitivity analyses anti-TG level/SUPPLEMENTARY MATERIAL S6I.png]

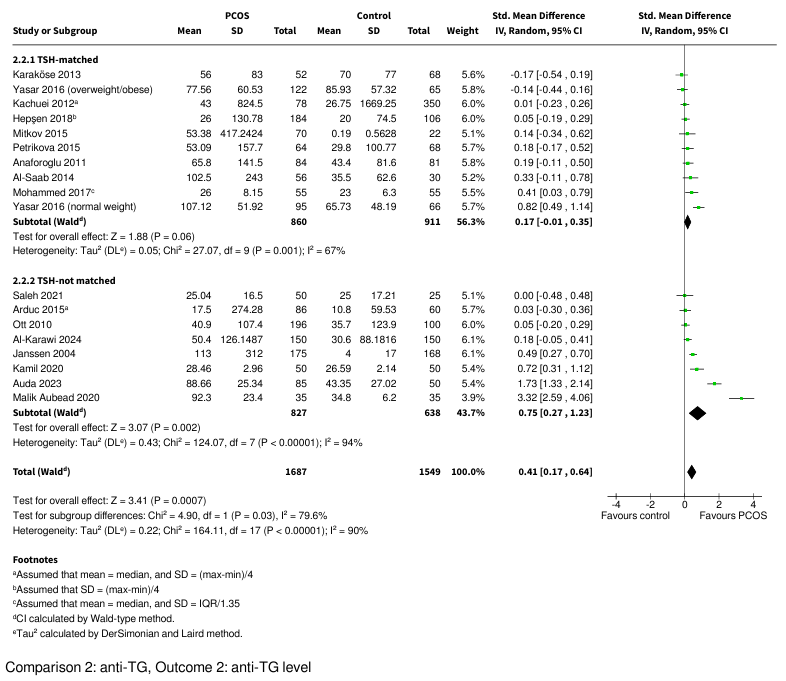

Supplement: Supplementary file 1 [file ijms-26-07525-s001.zip › Supplementary material S6 – sensitivity analyses anti-TG level/SUPPLEMENTARY MATERIAL S6J.png]

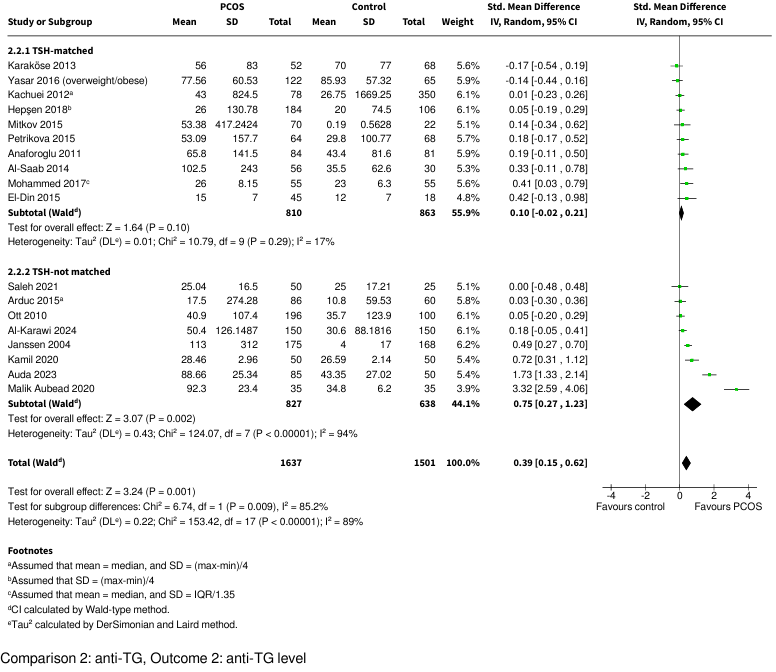

Supplement: Supplementary file 1 [file ijms-26-07525-s001.zip › Supplementary material S6 – sensitivity analyses anti-TG level/SUPPLEMENTARY MATERIAL S6K.png]

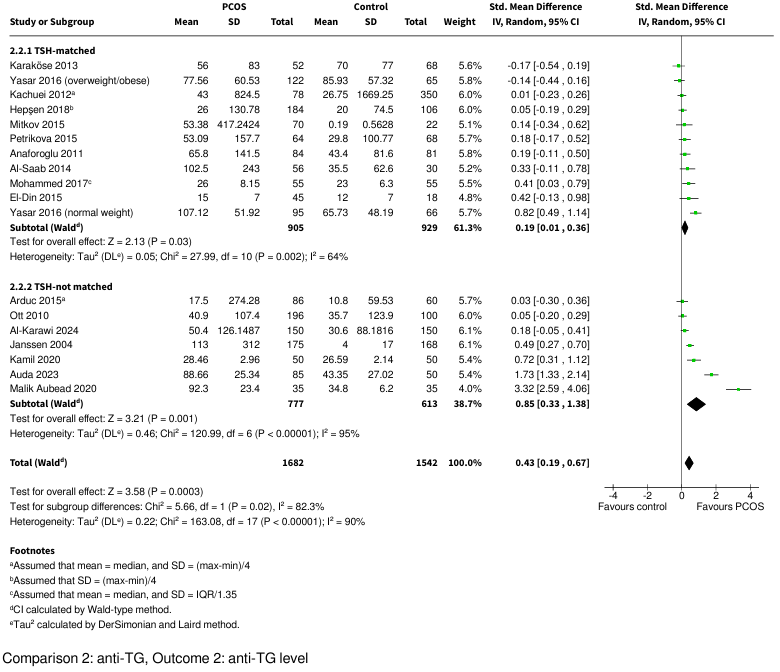

Supplement: Supplementary file 1 [file ijms-26-07525-s001.zip › Supplementary material S6 – sensitivity analyses anti-TG level/SUPPLEMENTARY MATERIAL S6L.png]

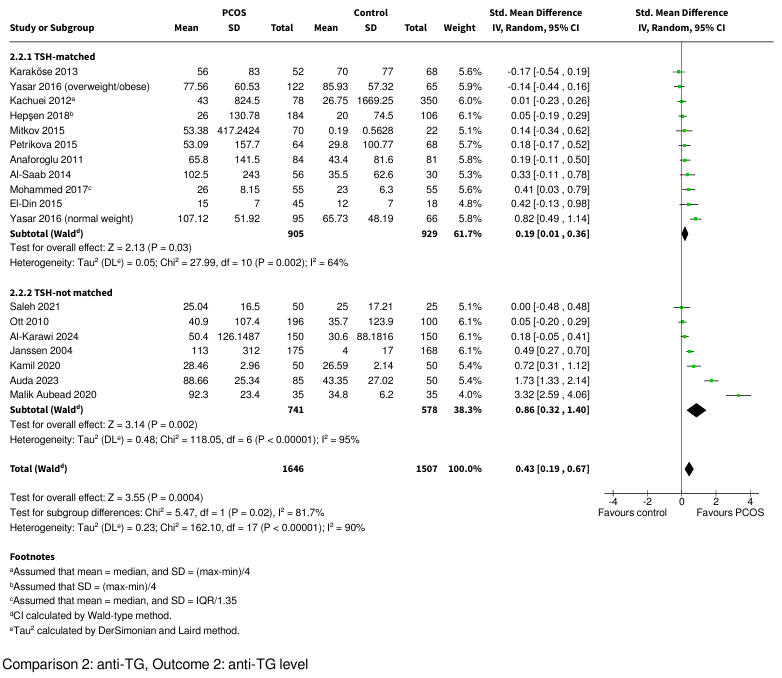

Supplement: Supplementary file 1 [file ijms-26-07525-s001.zip › Supplementary material S6 – sensitivity analyses anti-TG level/SUPPLEMENTARY MATERIAL S6M.png]

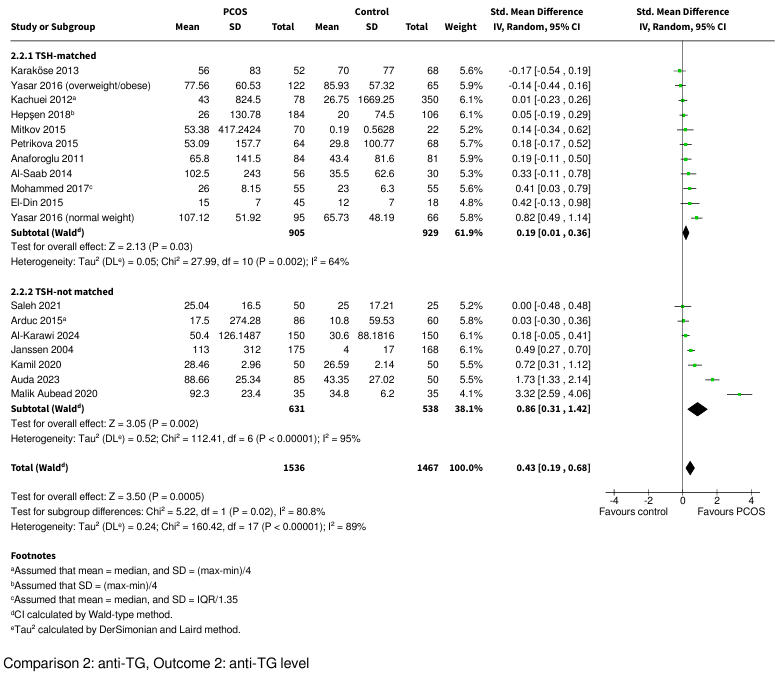

Supplement: Supplementary file 1 [file ijms-26-07525-s001.zip › Supplementary material S6 – sensitivity analyses anti-TG level/SUPPLEMENTARY MATERIAL S6N.png]

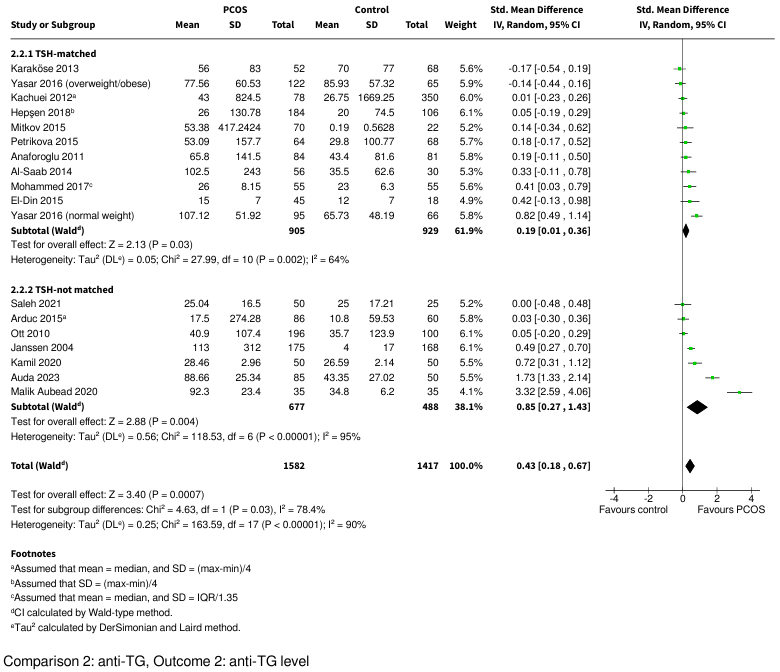

Supplement: Supplementary file 1 [file ijms-26-07525-s001.zip › Supplementary material S6 – sensitivity analyses anti-TG level/SUPPLEMENTARY MATERIAL S6O.png]

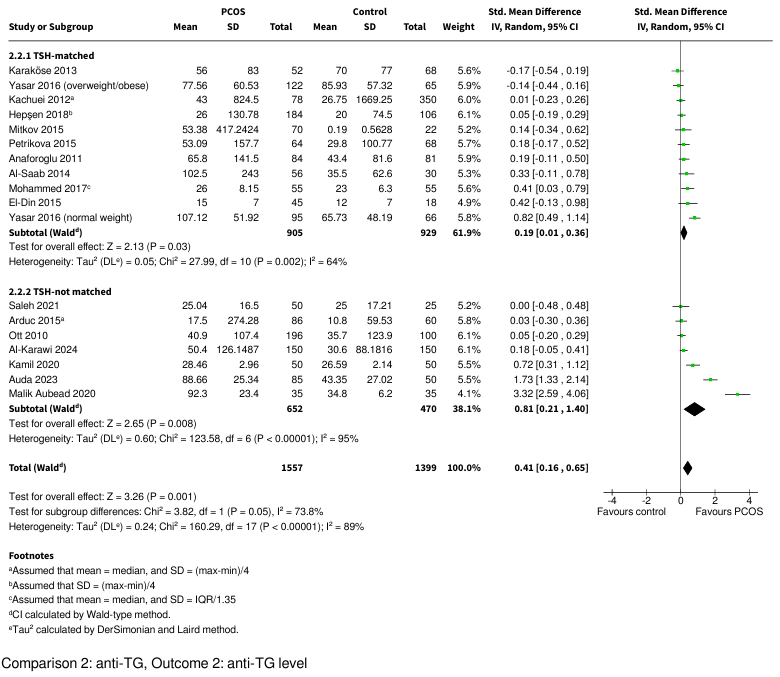

Supplement: Supplementary file 1 [file ijms-26-07525-s001.zip › Supplementary material S6 – sensitivity analyses anti-TG level/SUPPLEMENTARY MATERIAL S6P.png]

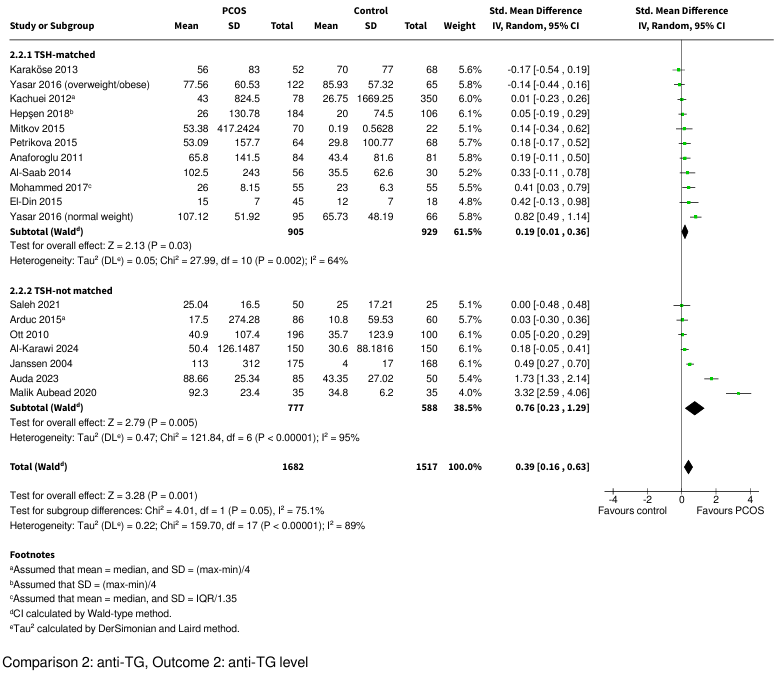

Supplement: Supplementary file 1 [file ijms-26-07525-s001.zip › Supplementary material S6 – sensitivity analyses anti-TG level/SUPPLEMENTARY MATERIAL S6Q.png]

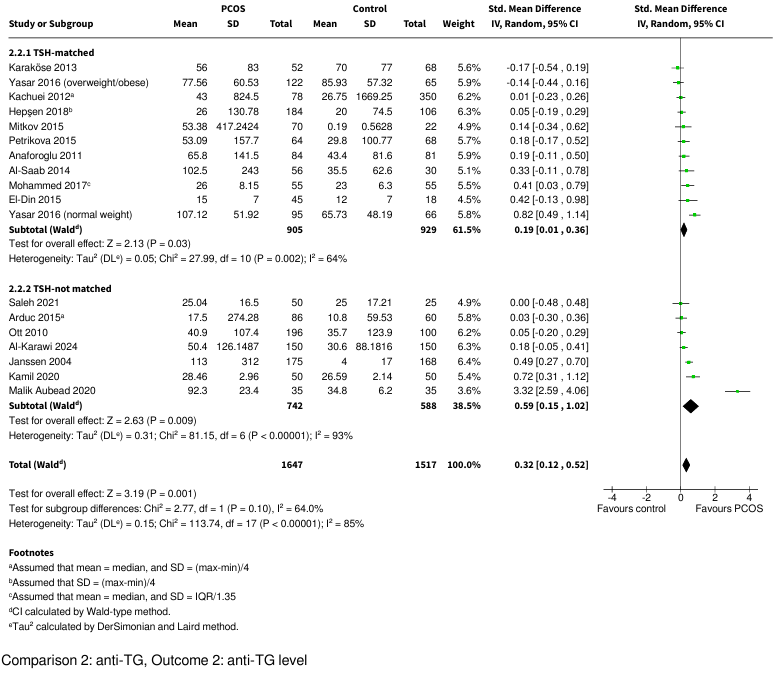

Supplement: Supplementary file 1 [file ijms-26-07525-s001.zip › Supplementary material S6 – sensitivity analyses anti-TG level/SUPPLEMENTARY MATERIAL S6R.png]

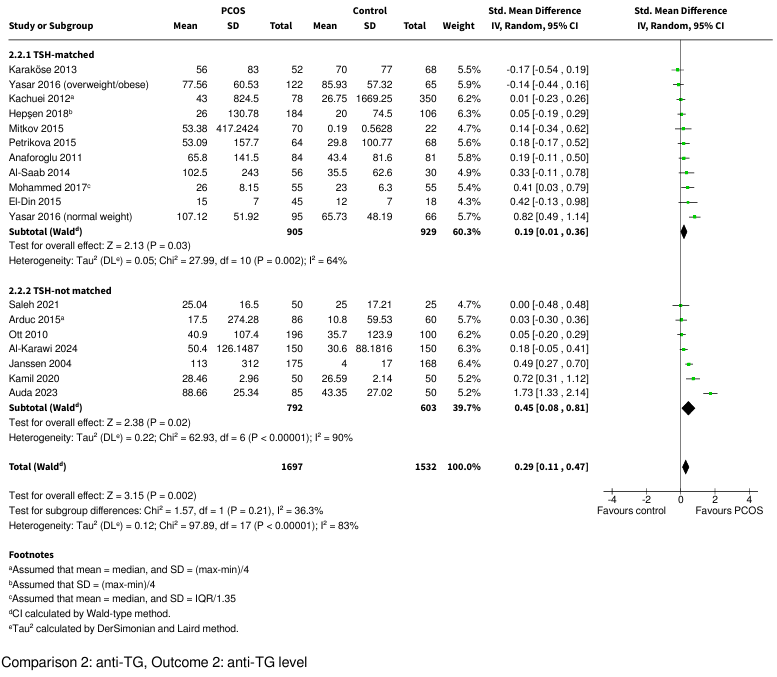

Supplement: Supplementary file 1 [file ijms-26-07525-s001.zip › Supplementary material S6 – sensitivity analyses anti-TG level/SUPPLEMENTARY MATERIAL S6S.png]

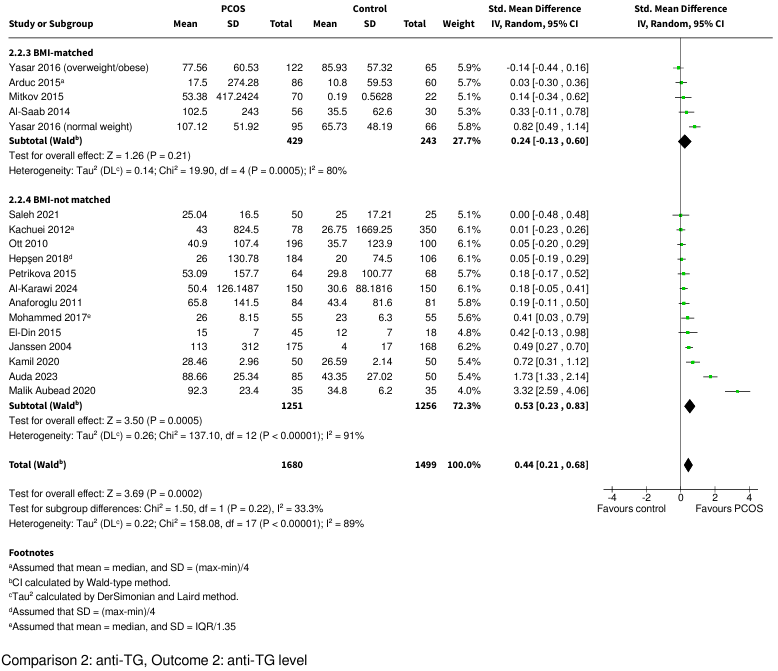

Supplement: Supplementary file 1 [file ijms-26-07525-s001.zip › Supplementary material S6 – sensitivity analyses anti-TG level/SUPPLEMENTARY MATERIAL S6T.png]

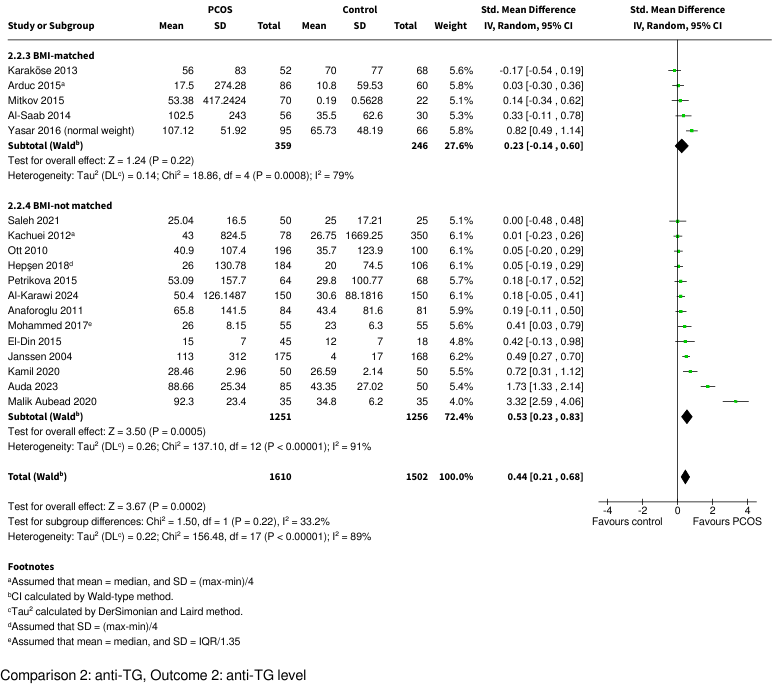

Supplement: Supplementary file 1 [file ijms-26-07525-s001.zip › Supplementary material S6 – sensitivity analyses anti-TG level/SUPPLEMENTARY MATERIAL S6U.png]

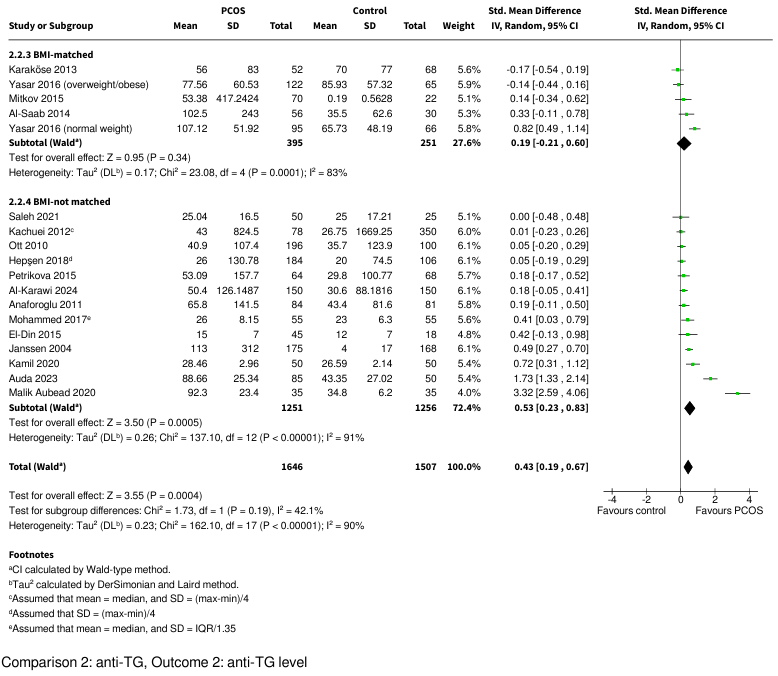

Supplement: Supplementary file 1 [file ijms-26-07525-s001.zip › Supplementary material S6 – sensitivity analyses anti-TG level/SUPPLEMENTARY MATERIAL S6V.png]

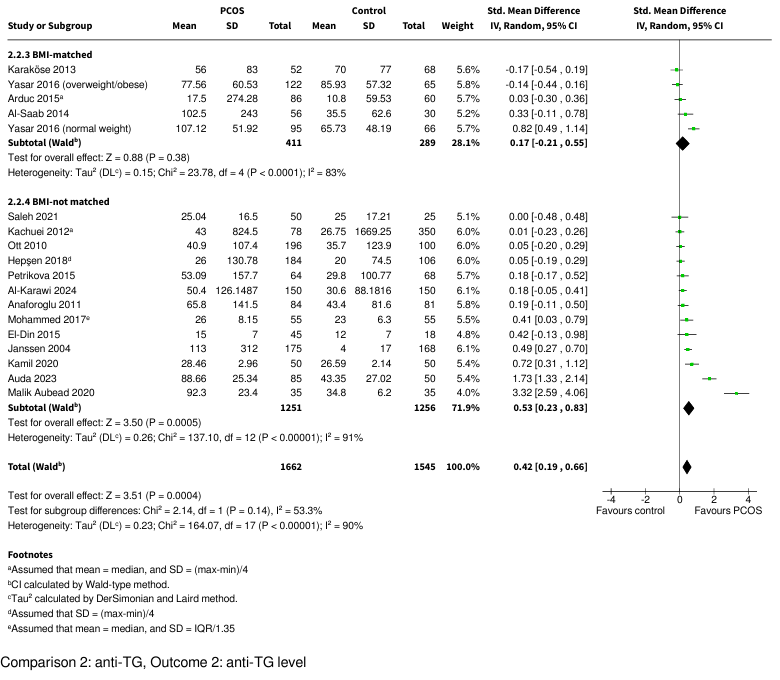

Supplement: Supplementary file 1 [file ijms-26-07525-s001.zip › Supplementary material S6 – sensitivity analyses anti-TG level/SUPPLEMENTARY MATERIAL S6W.png]

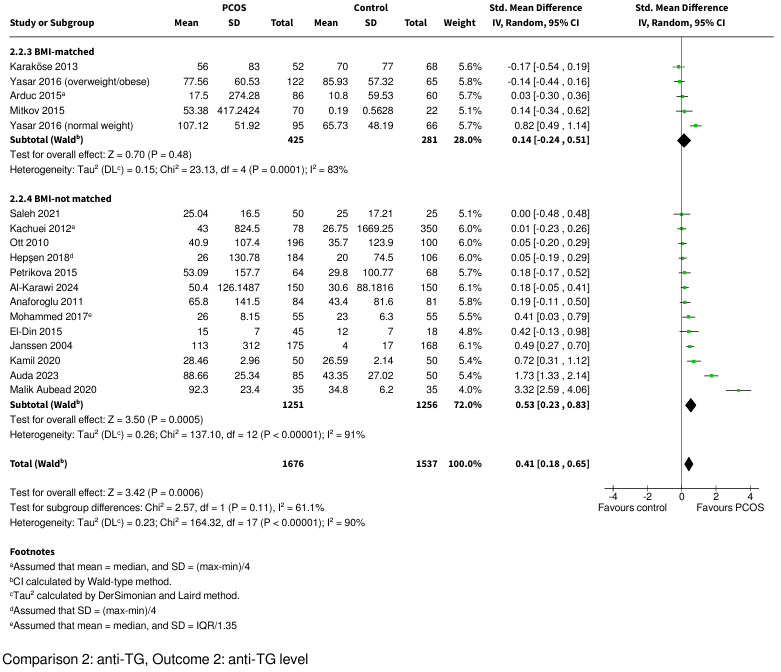

Supplement: Supplementary file 1 [file ijms-26-07525-s001.zip › Supplementary material S6 – sensitivity analyses anti-TG level/SUPPLEMENTARY MATERIAL S6X.png]

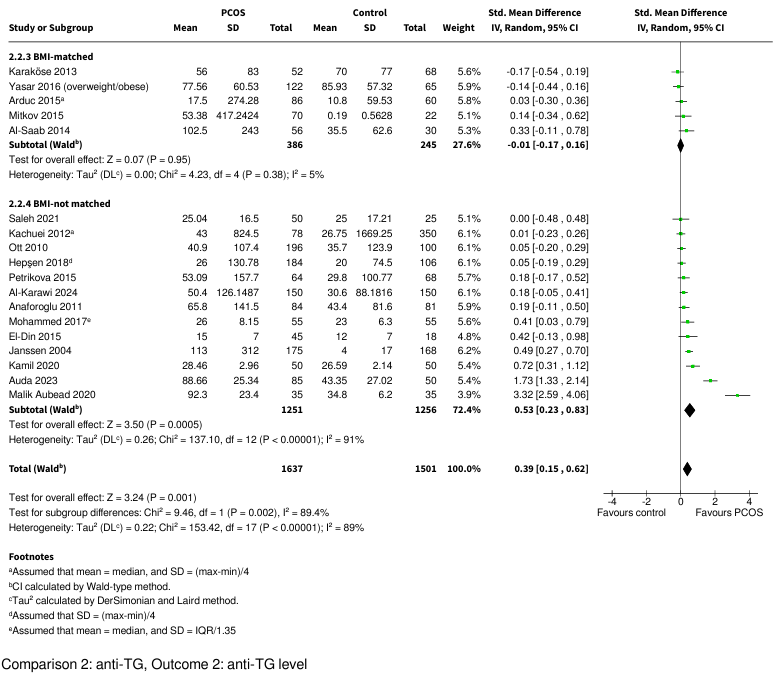

Supplement: Supplementary file 1 [file ijms-26-07525-s001.zip › Supplementary material S6 – sensitivity analyses anti-TG level/SUPPLEMENTARY MATERIAL S6Y.png]

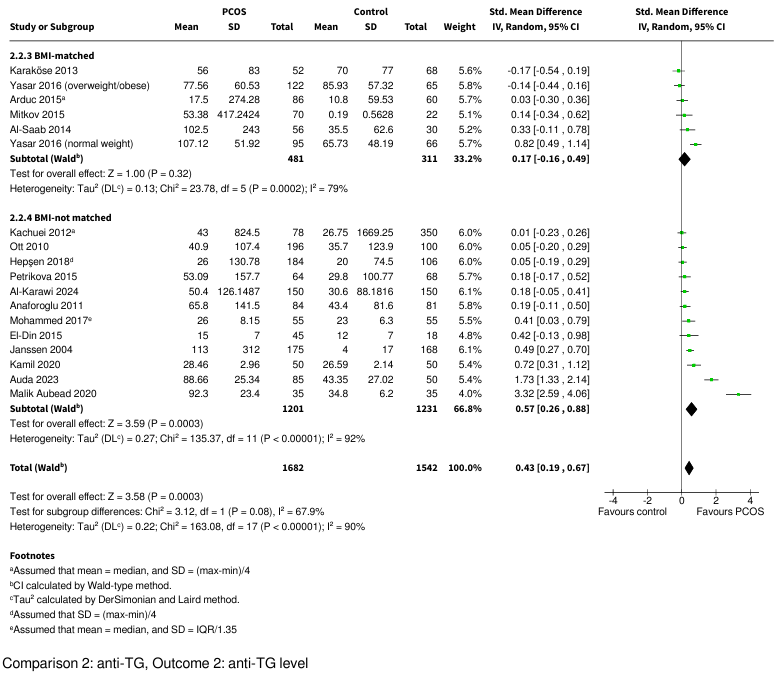

Supplement: Supplementary file 1 [file ijms-26-07525-s001.zip › Supplementary material S6 – sensitivity analyses anti-TG level/SUPPLEMENTARY MATERIAL S6Z.png]
