# Supplementary figures and images for: Prevalence and Levels of Thyroid Autoantibodies in Polycystic Ovary Syndrome—Impact of TSH- and BMI-Matched Comparisons: A Systematic Review and Meta-Analysis (part 1 of 2)
Source: Int J Mol Sci. 2025 Aug 4;26(15):7525. doi: 10.3390/ijms26157525 (PMC12347112; doi:10.3390/ijms26157525)

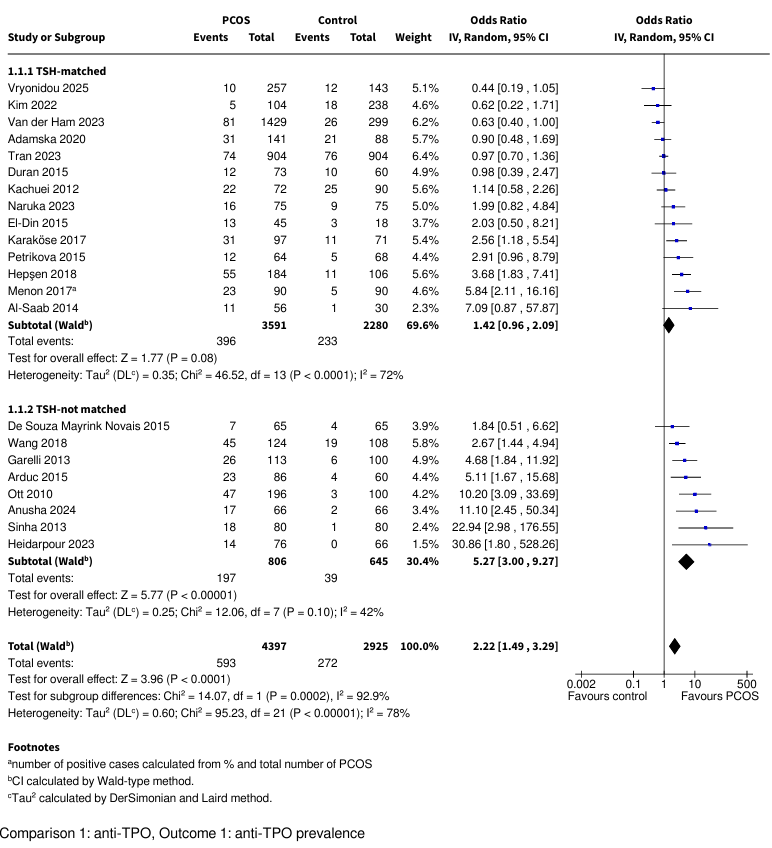

Supplement: Supplementary file 1 [file ijms-26-07525-s001.zip › Supplementary material S3 – sensitivity analyses anti-TPO prevalence/SUPPLEMENTARY MATERIAL S3A.png]

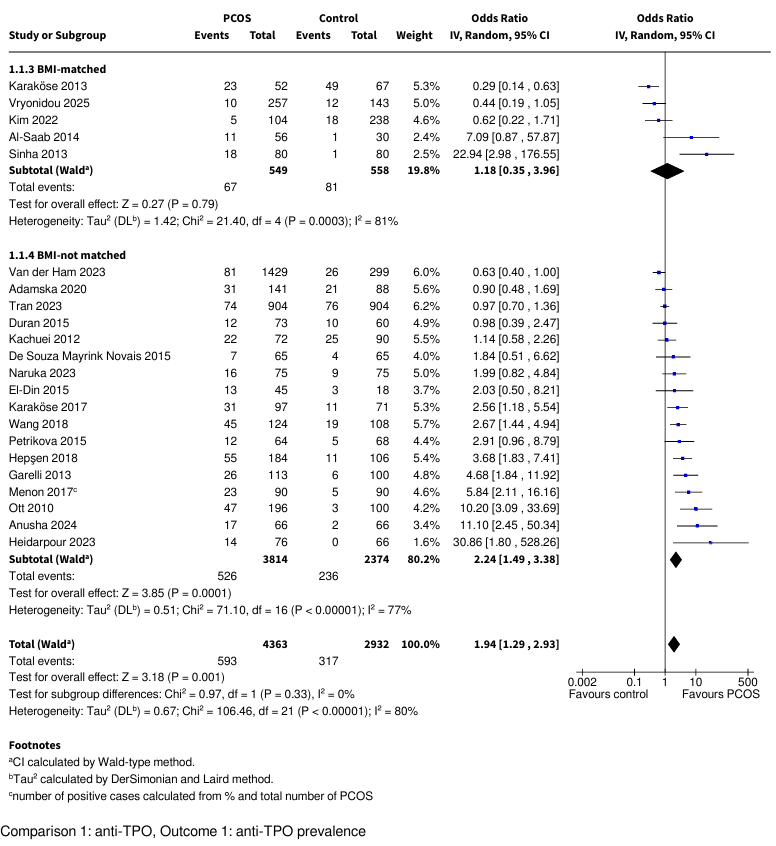

Supplement: Supplementary file 1 [file ijms-26-07525-s001.zip › Supplementary material S3 – sensitivity analyses anti-TPO prevalence/SUPPLEMENTARY MATERIAL S3AA.png]

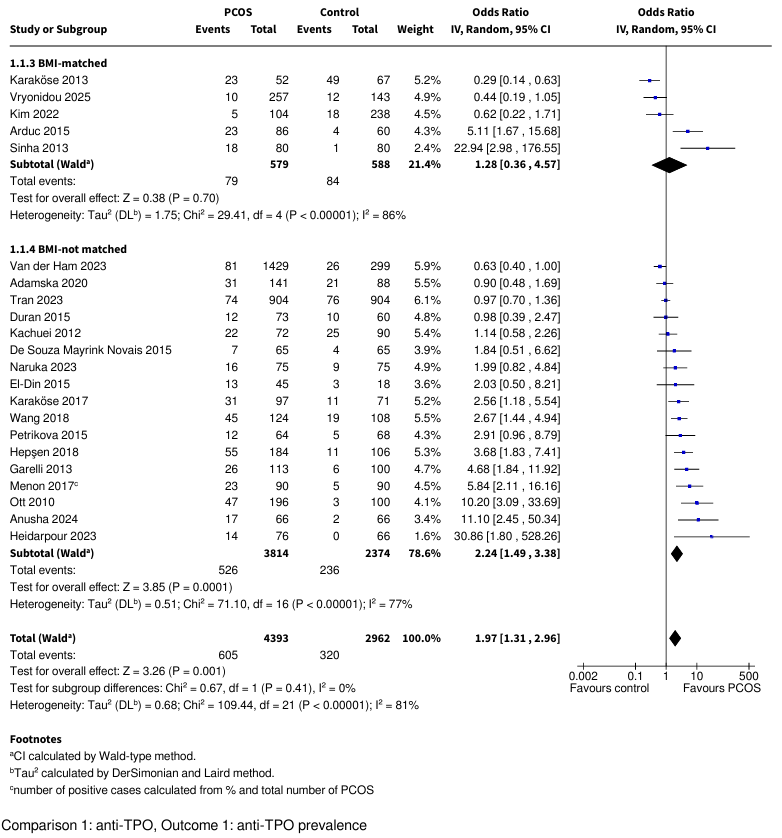

Supplement: Supplementary file 1 [file ijms-26-07525-s001.zip › Supplementary material S3 – sensitivity analyses anti-TPO prevalence/SUPPLEMENTARY MATERIAL S3AB.png]

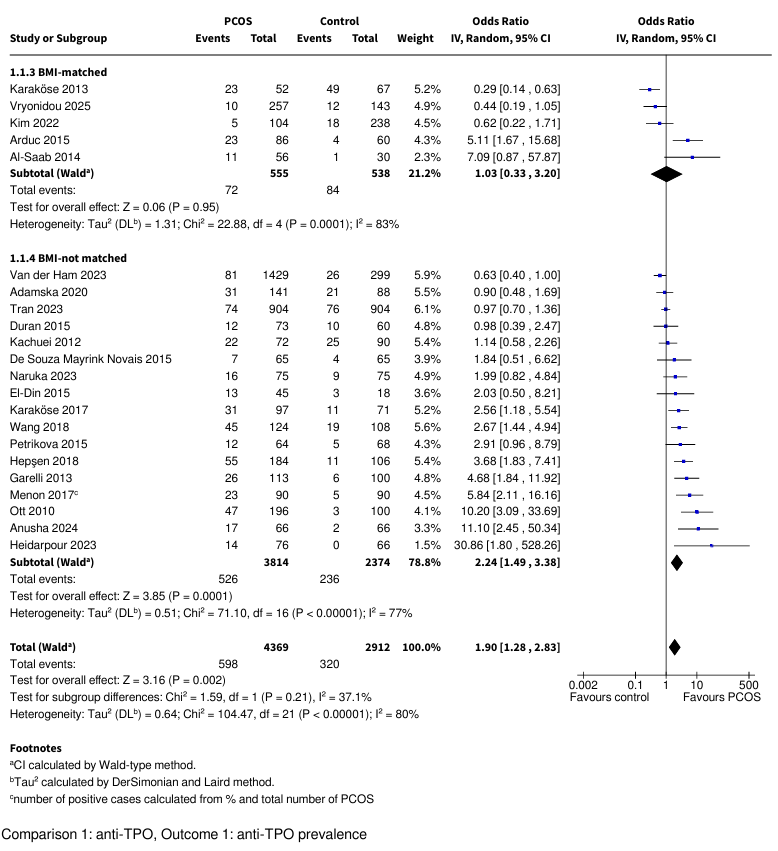

Supplement: Supplementary file 1 [file ijms-26-07525-s001.zip › Supplementary material S3 – sensitivity analyses anti-TPO prevalence/SUPPLEMENTARY MATERIAL S3AC.png]

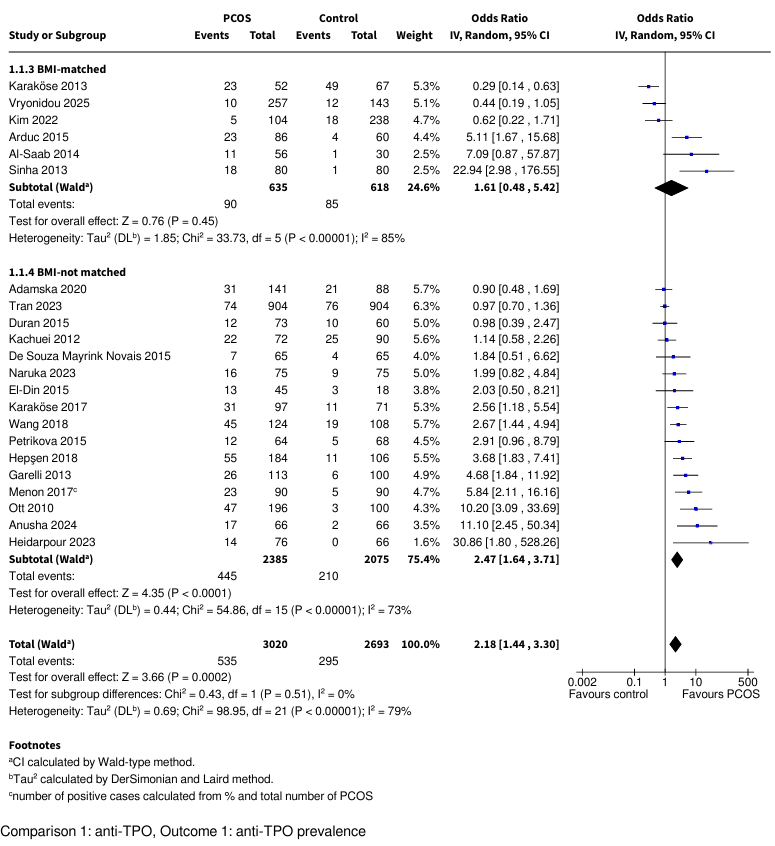

Supplement: Supplementary file 1 [file ijms-26-07525-s001.zip › Supplementary material S3 – sensitivity analyses anti-TPO prevalence/SUPPLEMENTARY MATERIAL S3AD.png]

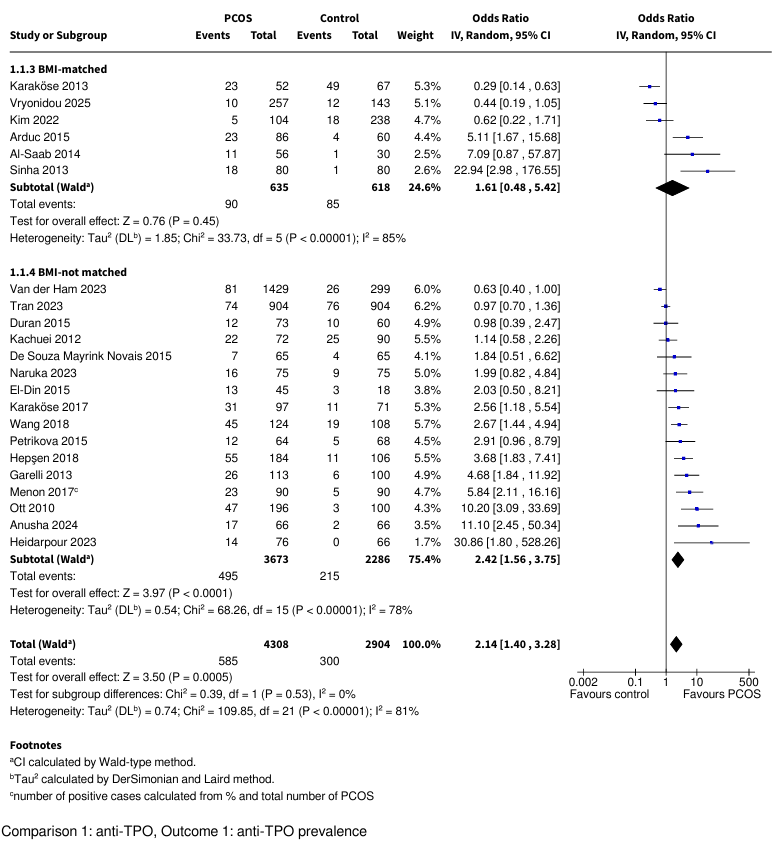

Supplement: Supplementary file 1 [file ijms-26-07525-s001.zip › Supplementary material S3 – sensitivity analyses anti-TPO prevalence/SUPPLEMENTARY MATERIAL S3AE.png]

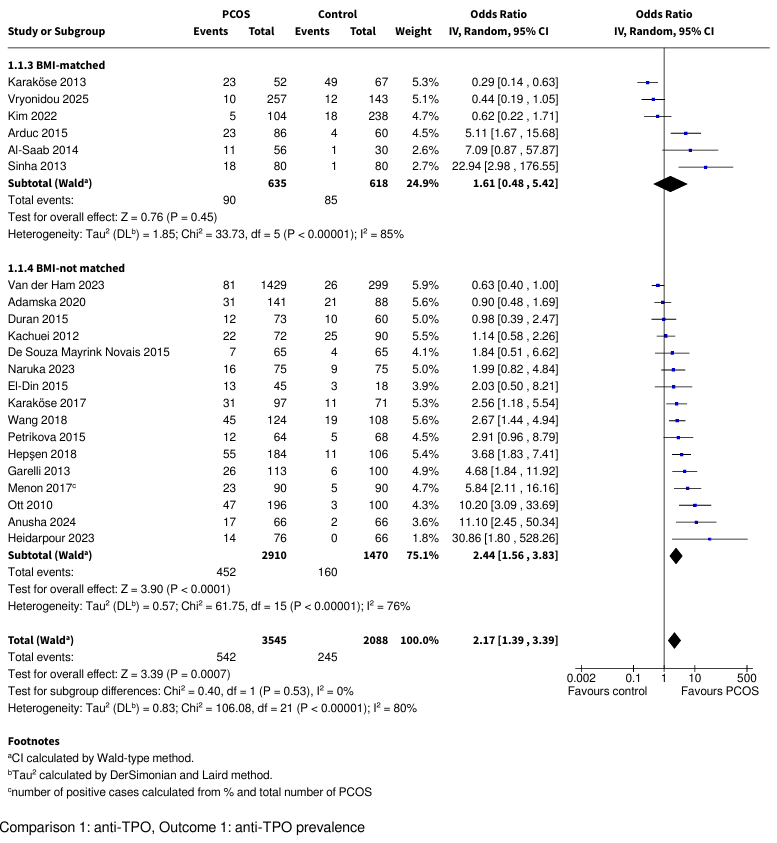

Supplement: Supplementary file 1 [file ijms-26-07525-s001.zip › Supplementary material S3 – sensitivity analyses anti-TPO prevalence/SUPPLEMENTARY MATERIAL S3AF.png]

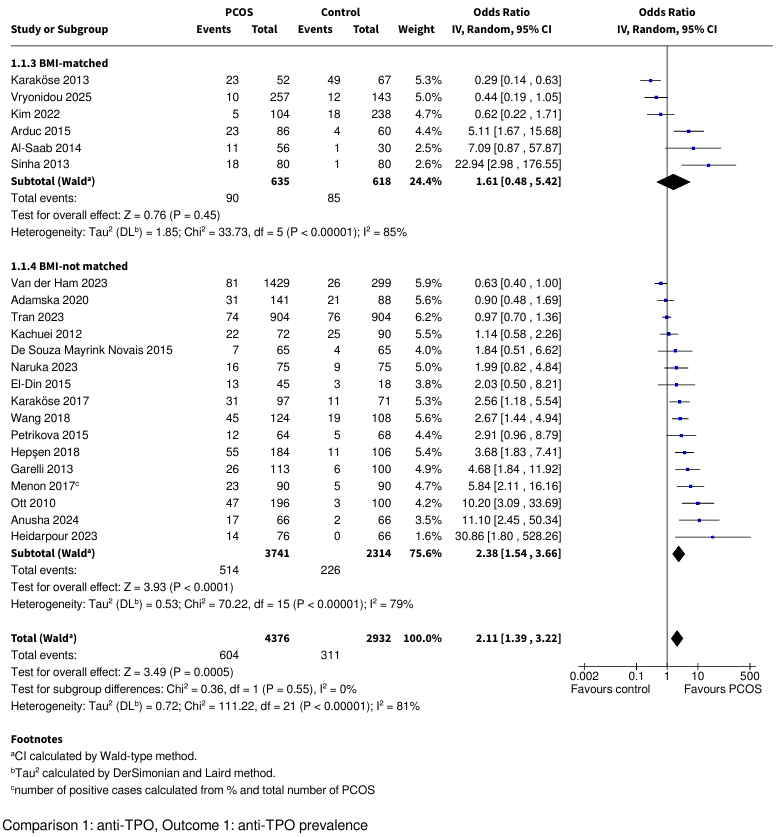

Supplement: Supplementary file 1 [file ijms-26-07525-s001.zip › Supplementary material S3 – sensitivity analyses anti-TPO prevalence/SUPPLEMENTARY MATERIAL S3AG.png]

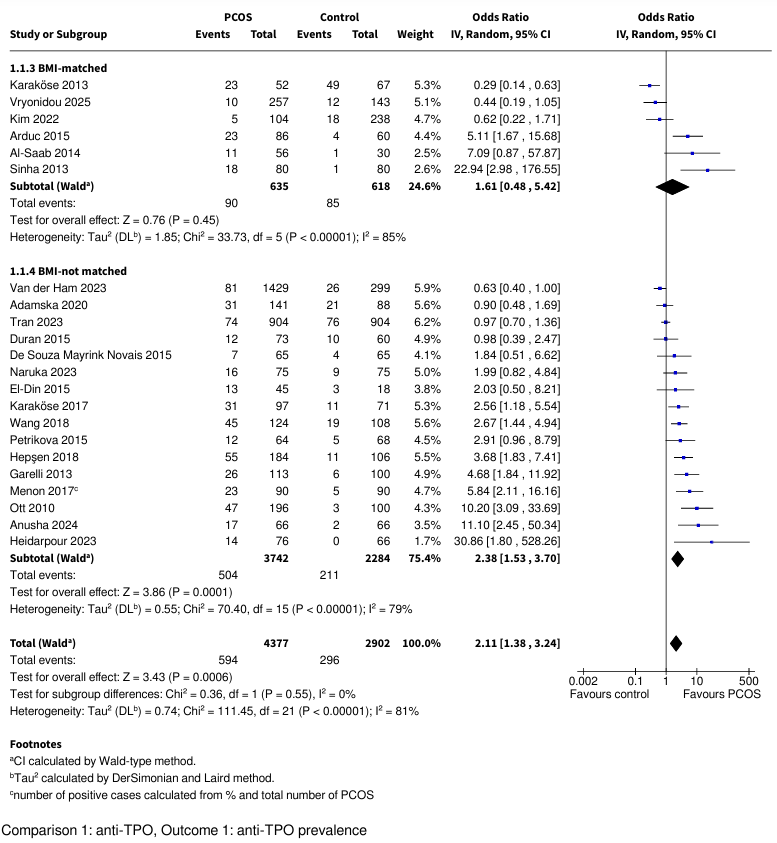

Supplement: Supplementary file 1 [file ijms-26-07525-s001.zip › Supplementary material S3 – sensitivity analyses anti-TPO prevalence/SUPPLEMENTARY MATERIAL S3AH.png]

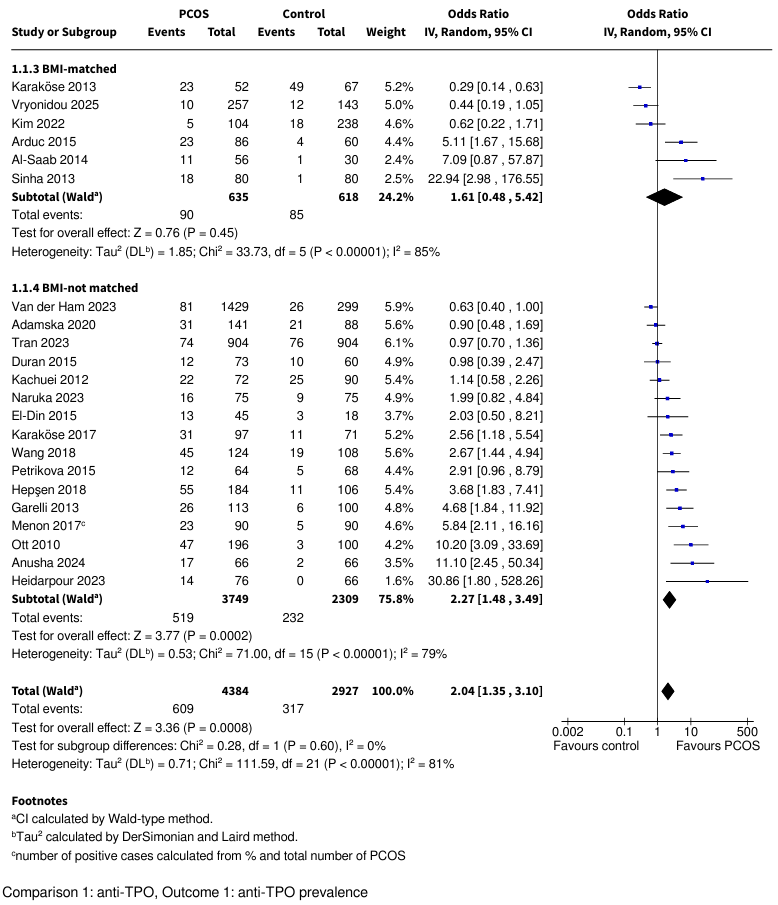

Supplement: Supplementary file 1 [file ijms-26-07525-s001.zip › Supplementary material S3 – sensitivity analyses anti-TPO prevalence/SUPPLEMENTARY MATERIAL S3AI.png]

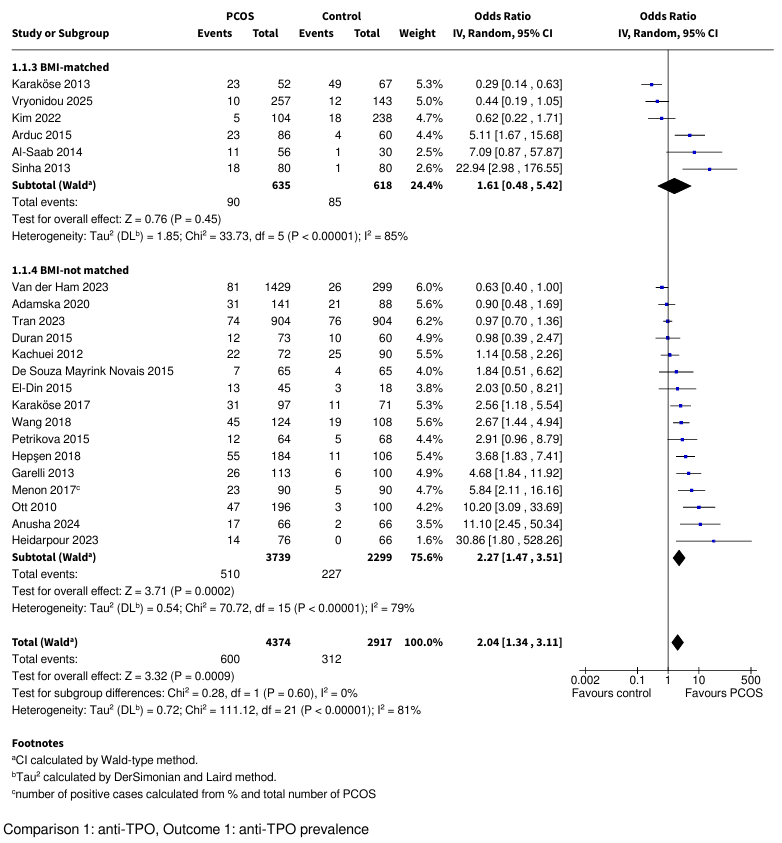

Supplement: Supplementary file 1 [file ijms-26-07525-s001.zip › Supplementary material S3 – sensitivity analyses anti-TPO prevalence/SUPPLEMENTARY MATERIAL S3AJ.png]

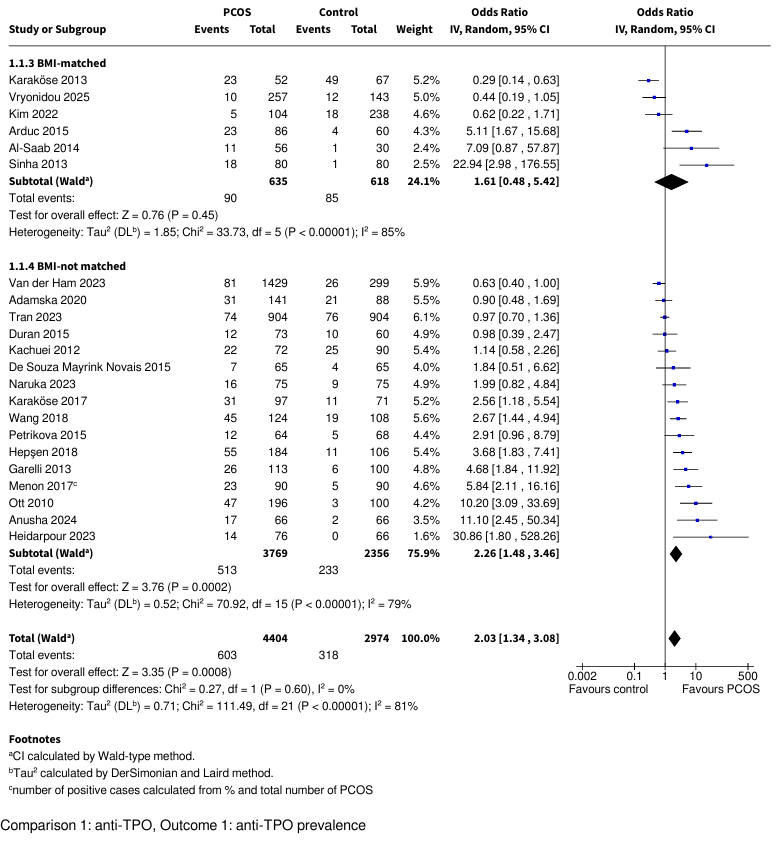

Supplement: Supplementary file 1 [file ijms-26-07525-s001.zip › Supplementary material S3 – sensitivity analyses anti-TPO prevalence/SUPPLEMENTARY MATERIAL S3AK.png]

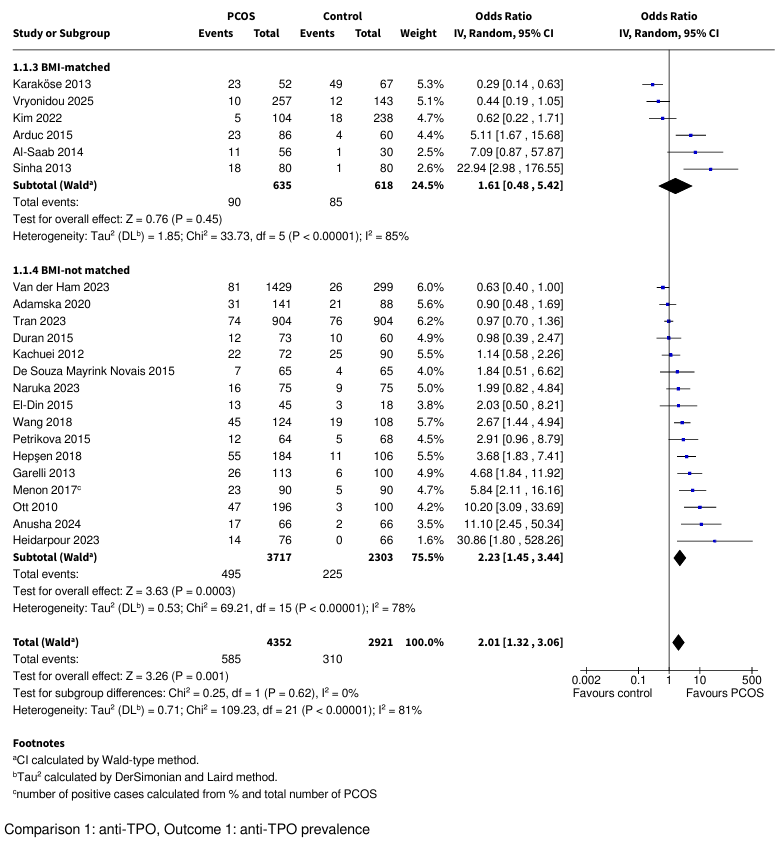

Supplement: Supplementary file 1 [file ijms-26-07525-s001.zip › Supplementary material S3 – sensitivity analyses anti-TPO prevalence/SUPPLEMENTARY MATERIAL S3AL.png]

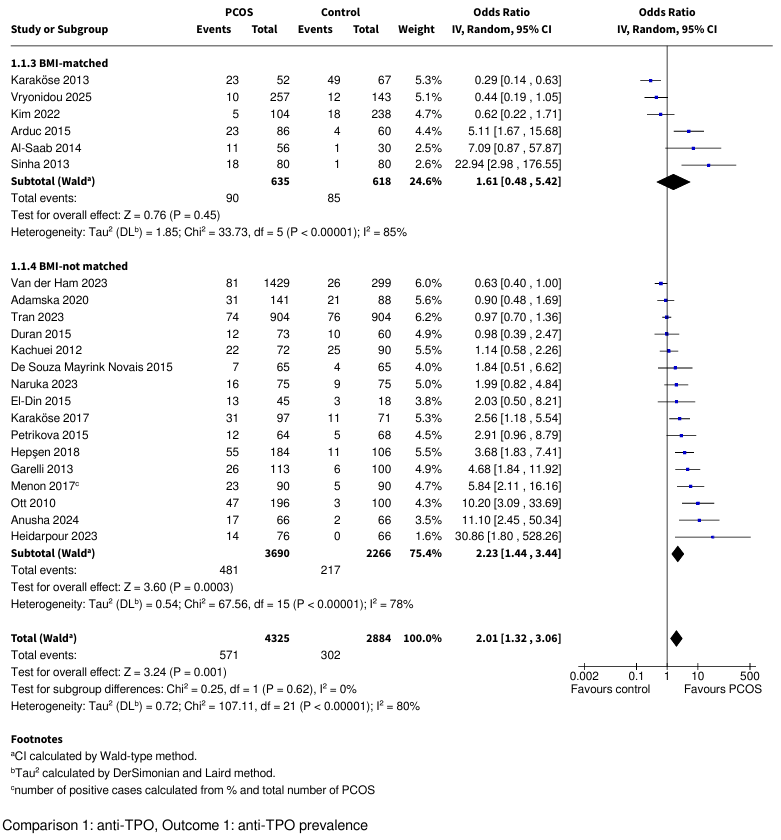

Supplement: Supplementary file 1 [file ijms-26-07525-s001.zip › Supplementary material S3 – sensitivity analyses anti-TPO prevalence/SUPPLEMENTARY MATERIAL S3AM.png]

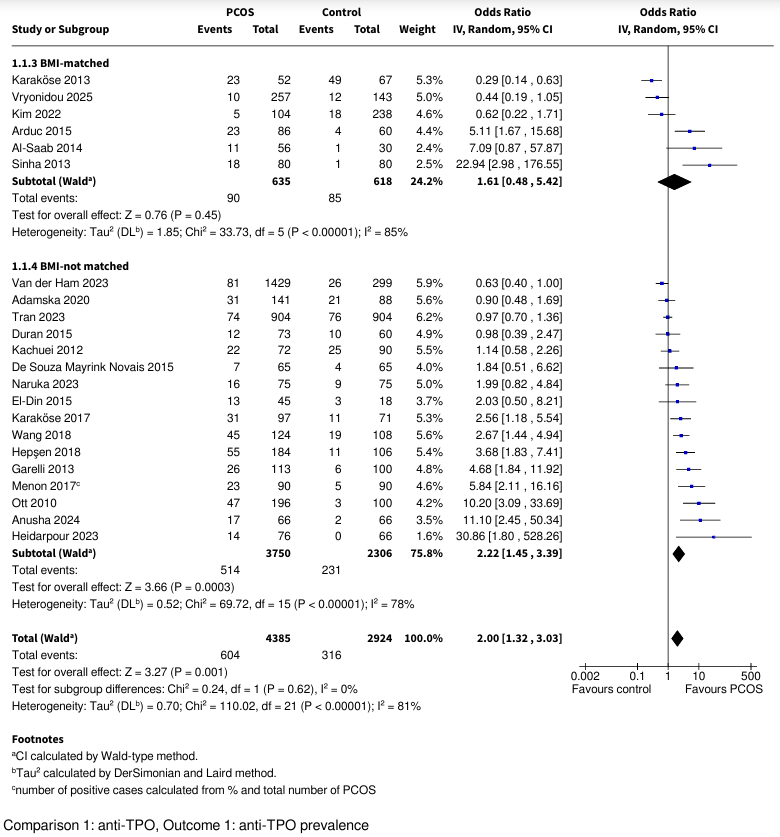

Supplement: Supplementary file 1 [file ijms-26-07525-s001.zip › Supplementary material S3 – sensitivity analyses anti-TPO prevalence/SUPPLEMENTARY MATERIAL S3AN.png]

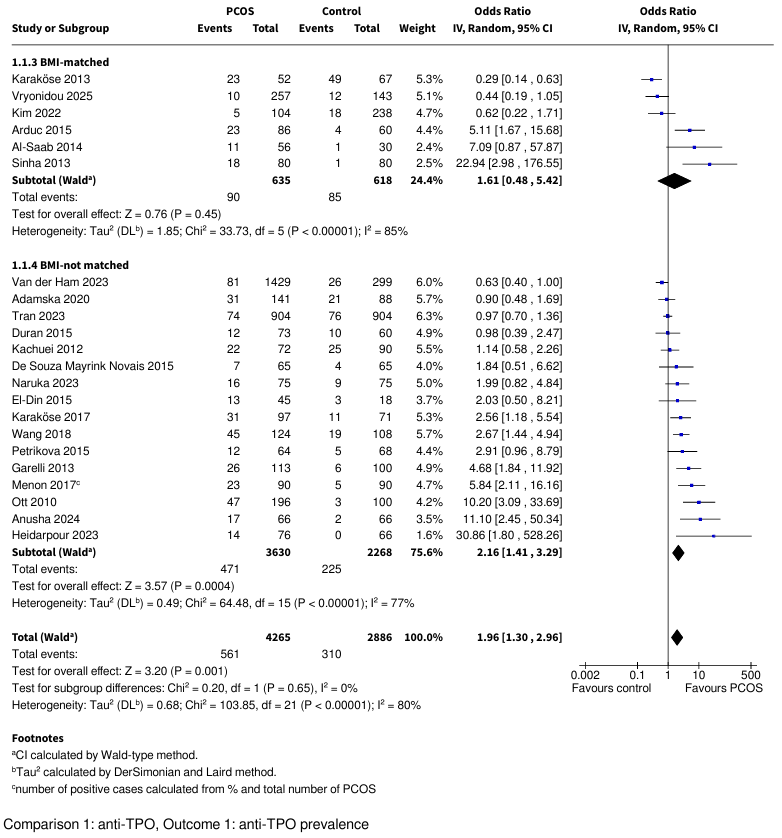

Supplement: Supplementary file 1 [file ijms-26-07525-s001.zip › Supplementary material S3 – sensitivity analyses anti-TPO prevalence/SUPPLEMENTARY MATERIAL S3AO.png]

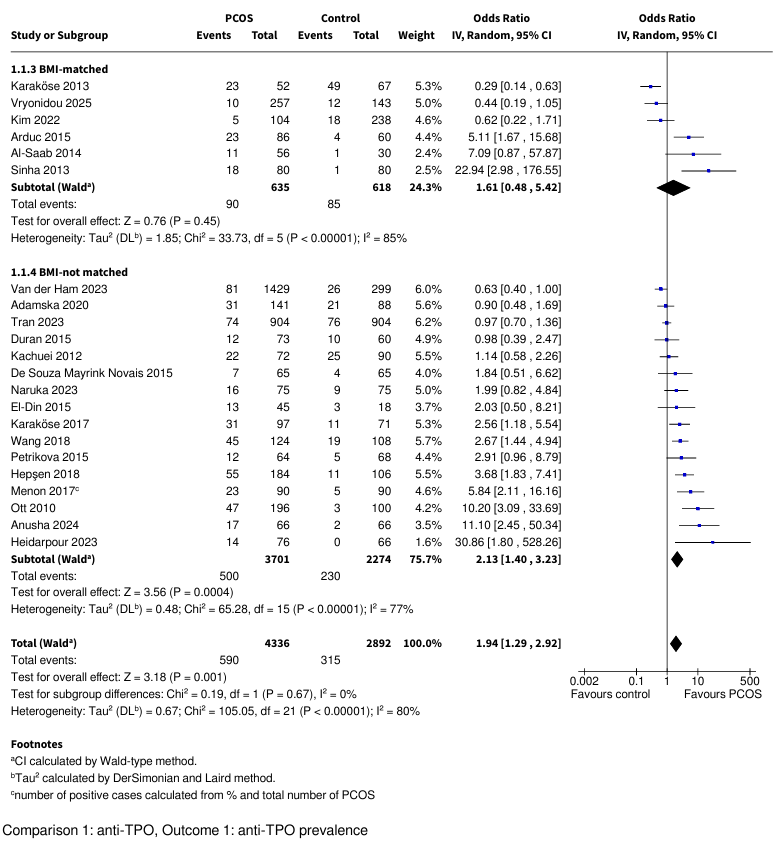

Supplement: Supplementary file 1 [file ijms-26-07525-s001.zip › Supplementary material S3 – sensitivity analyses anti-TPO prevalence/SUPPLEMENTARY MATERIAL S3AP.png]

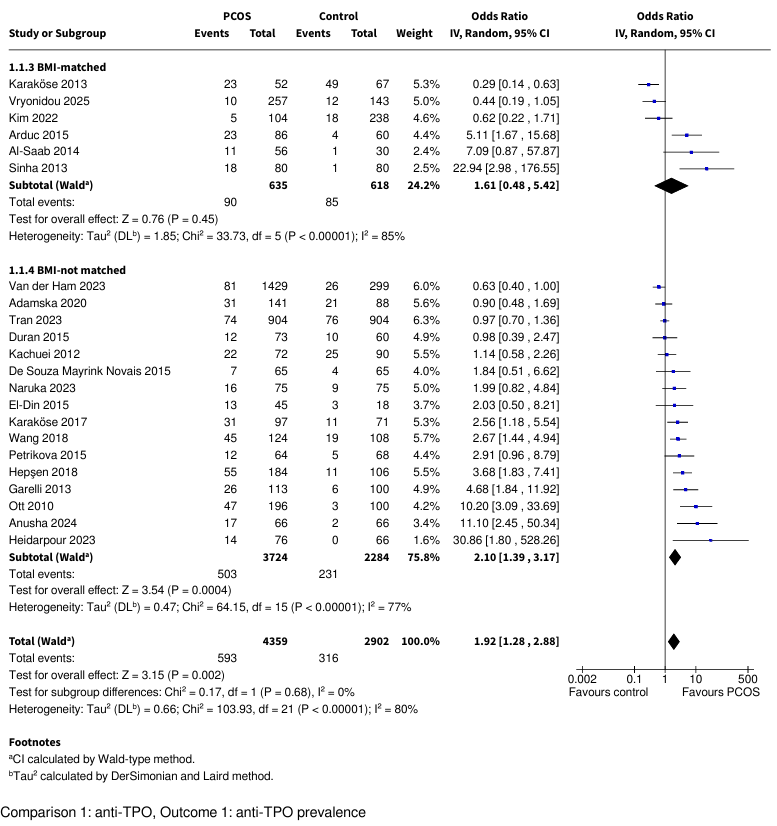

Supplement: Supplementary file 1 [file ijms-26-07525-s001.zip › Supplementary material S3 – sensitivity analyses anti-TPO prevalence/SUPPLEMENTARY MATERIAL S3AQ.png]

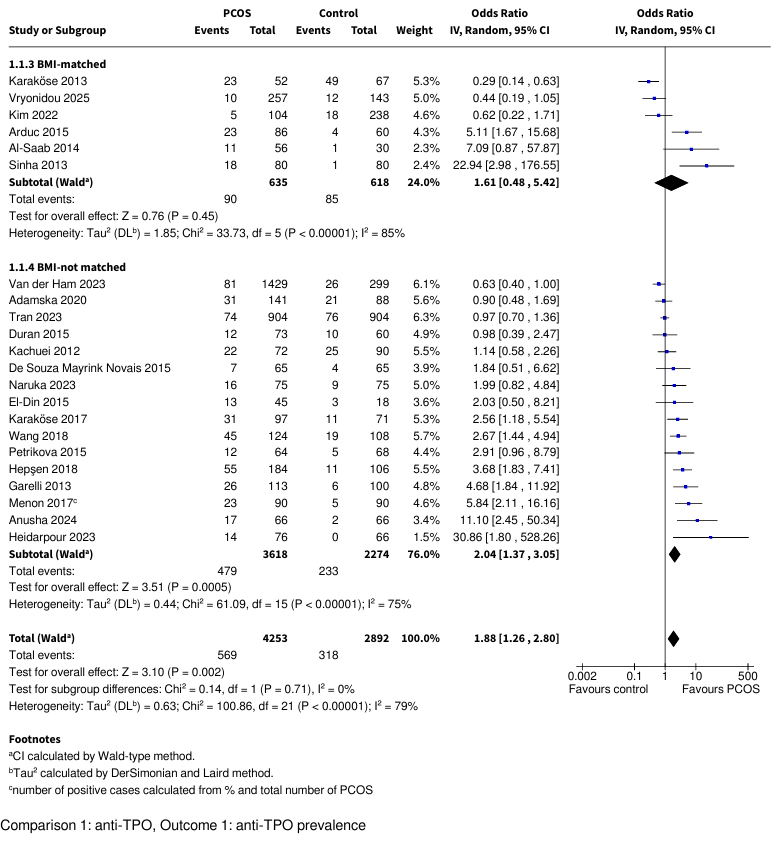

Supplement: Supplementary file 1 [file ijms-26-07525-s001.zip › Supplementary material S3 – sensitivity analyses anti-TPO prevalence/SUPPLEMENTARY MATERIAL S3AR.png]

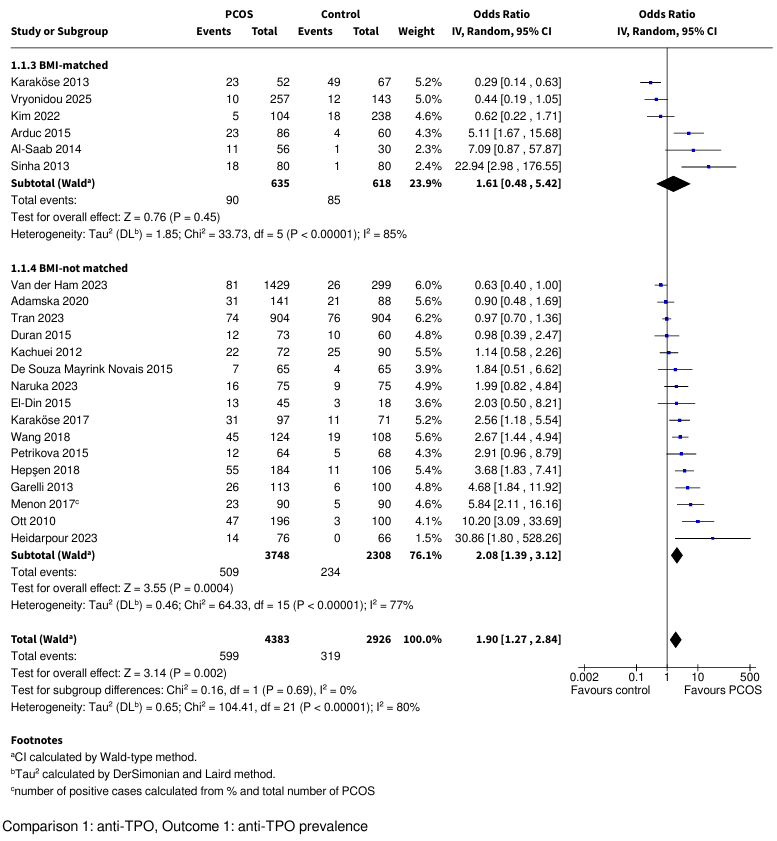

Supplement: Supplementary file 1 [file ijms-26-07525-s001.zip › Supplementary material S3 – sensitivity analyses anti-TPO prevalence/SUPPLEMENTARY MATERIAL S3AS.png]

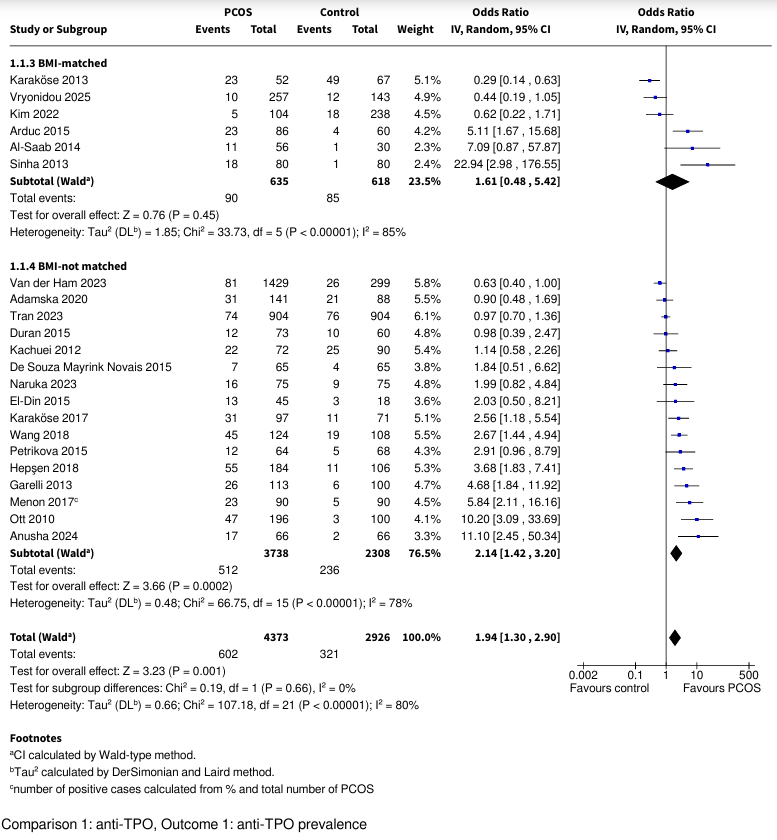

Supplement: Supplementary file 1 [file ijms-26-07525-s001.zip › Supplementary material S3 – sensitivity analyses anti-TPO prevalence/SUPPLEMENTARY MATERIAL S3AT.png]

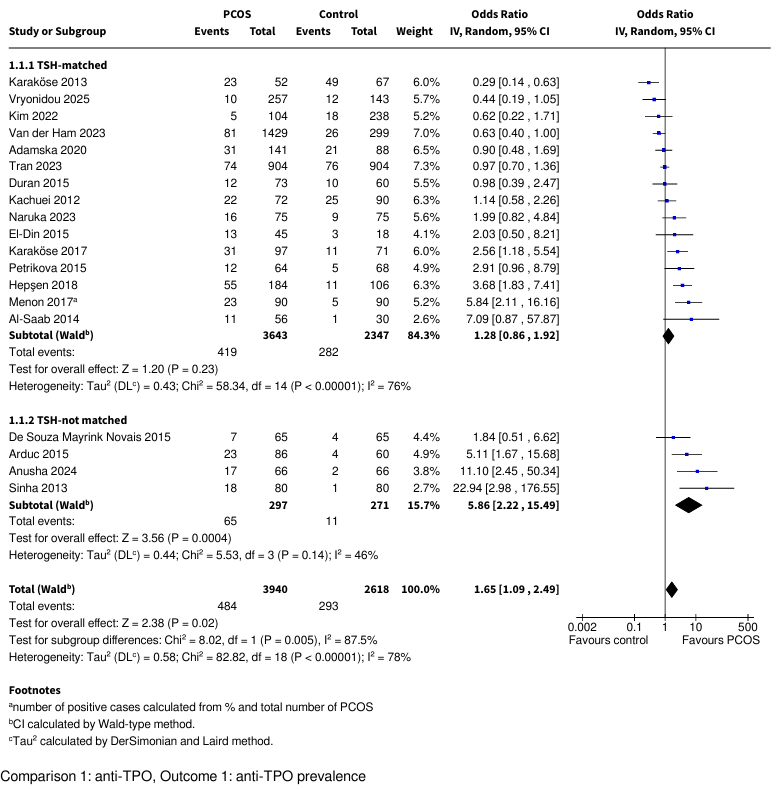

Supplement: Supplementary file 1 [file ijms-26-07525-s001.zip › Supplementary material S3 – sensitivity analyses anti-TPO prevalence/SUPPLEMENTARY MATERIAL S3AU.png]

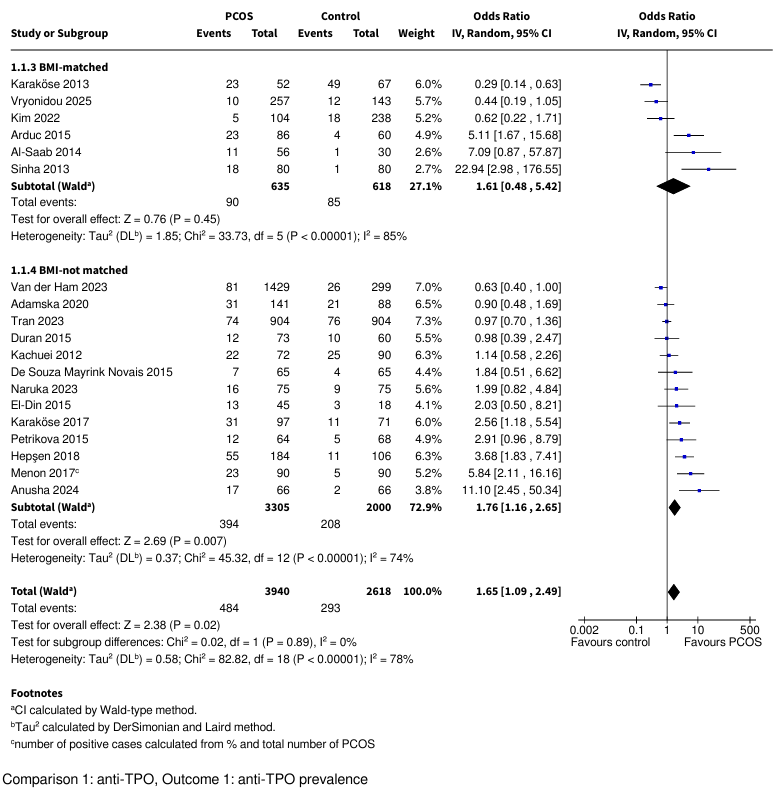

Supplement: Supplementary file 1 [file ijms-26-07525-s001.zip › Supplementary material S3 – sensitivity analyses anti-TPO prevalence/SUPPLEMENTARY MATERIAL S3AV.png]

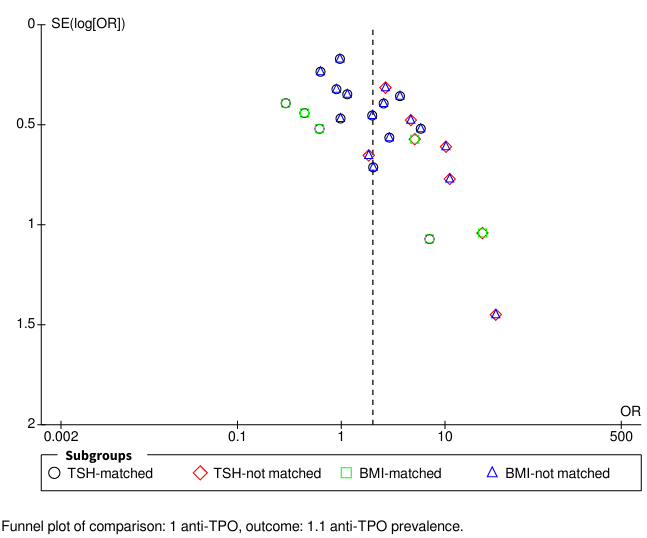

Supplement: Supplementary file 1 [file ijms-26-07525-s001.zip › Supplementary material S3 – sensitivity analyses anti-TPO prevalence/SUPPLEMENTARY MATERIAL S3AW.png]

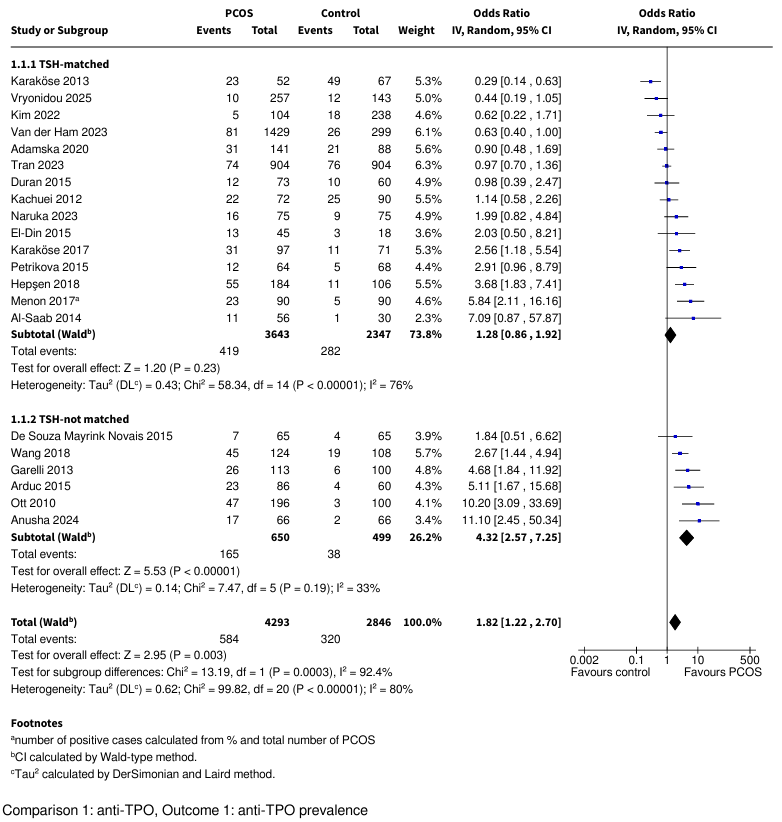

Supplement: Supplementary file 1 [file ijms-26-07525-s001.zip › Supplementary material S3 – sensitivity analyses anti-TPO prevalence/SUPPLEMENTARY MATERIAL S3AX.png]

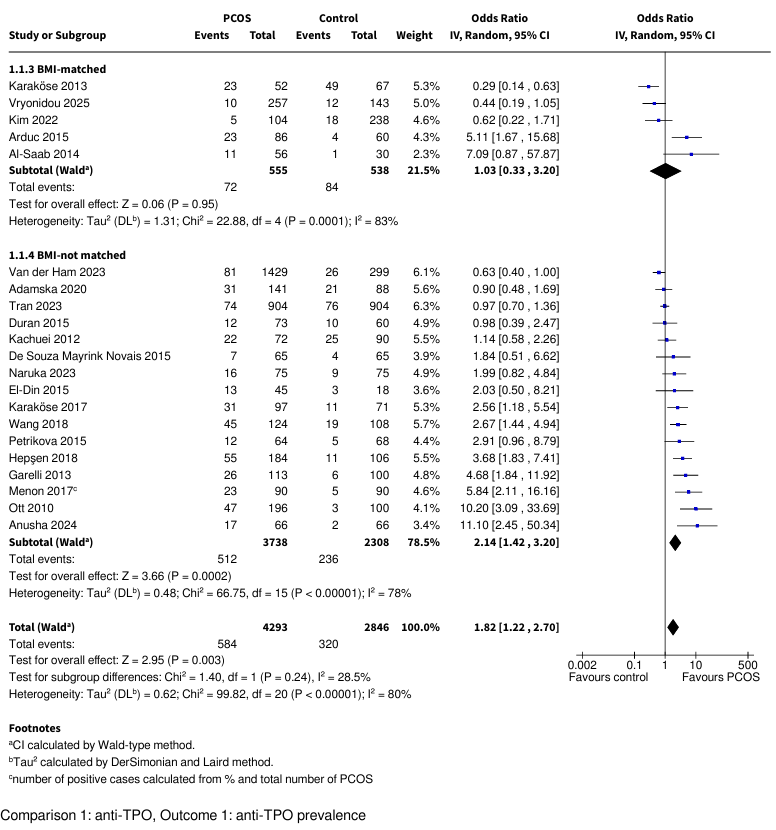

Supplement: Supplementary file 1 [file ijms-26-07525-s001.zip › Supplementary material S3 – sensitivity analyses anti-TPO prevalence/SUPPLEMENTARY MATERIAL S3AY.png]

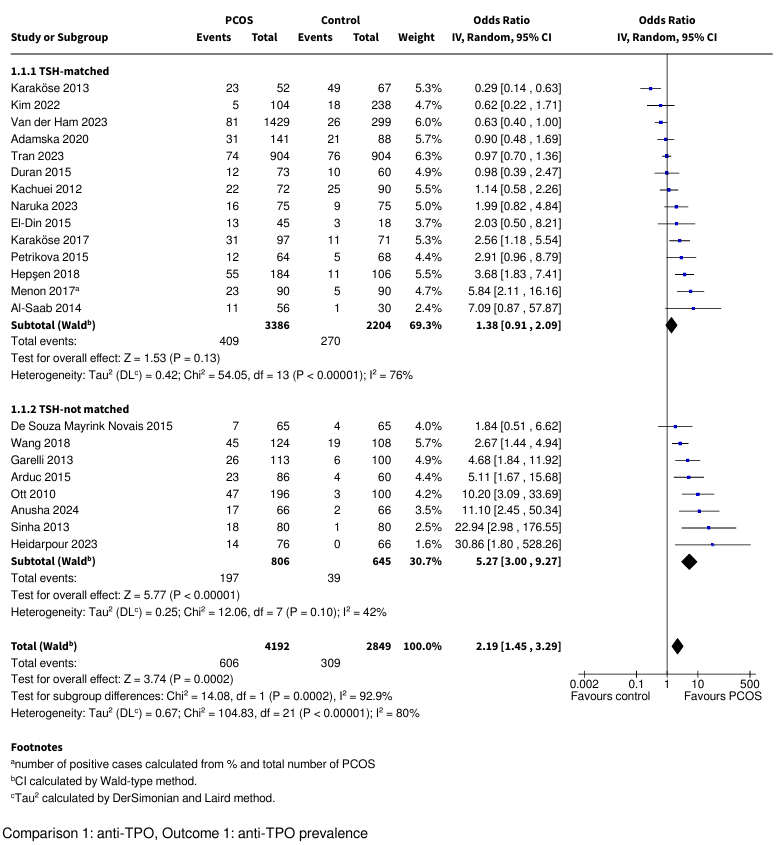

Supplement: Supplementary file 1 [file ijms-26-07525-s001.zip › Supplementary material S3 – sensitivity analyses anti-TPO prevalence/SUPPLEMENTARY MATERIAL S3B.png]

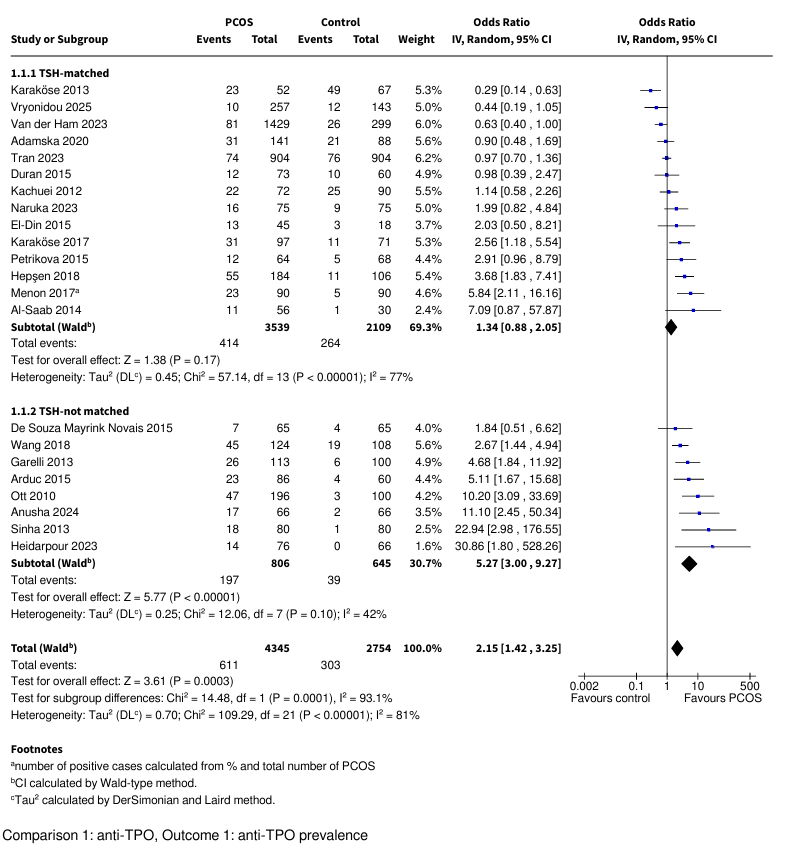

Supplement: Supplementary file 1 [file ijms-26-07525-s001.zip › Supplementary material S3 – sensitivity analyses anti-TPO prevalence/SUPPLEMENTARY MATERIAL S3C.png]

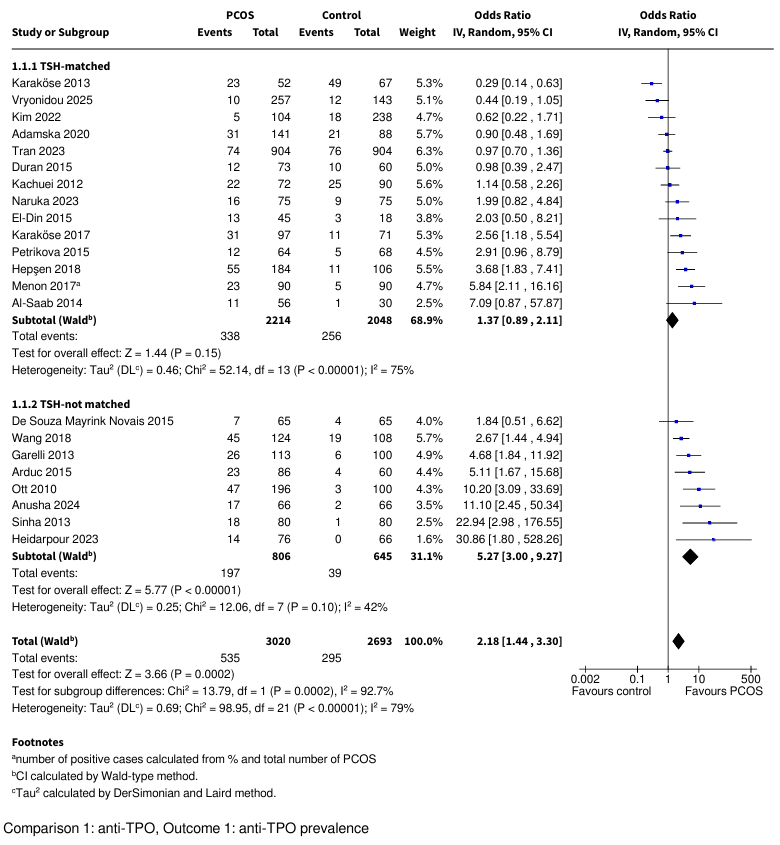

Supplement: Supplementary file 1 [file ijms-26-07525-s001.zip › Supplementary material S3 – sensitivity analyses anti-TPO prevalence/SUPPLEMENTARY MATERIAL S3D.png]

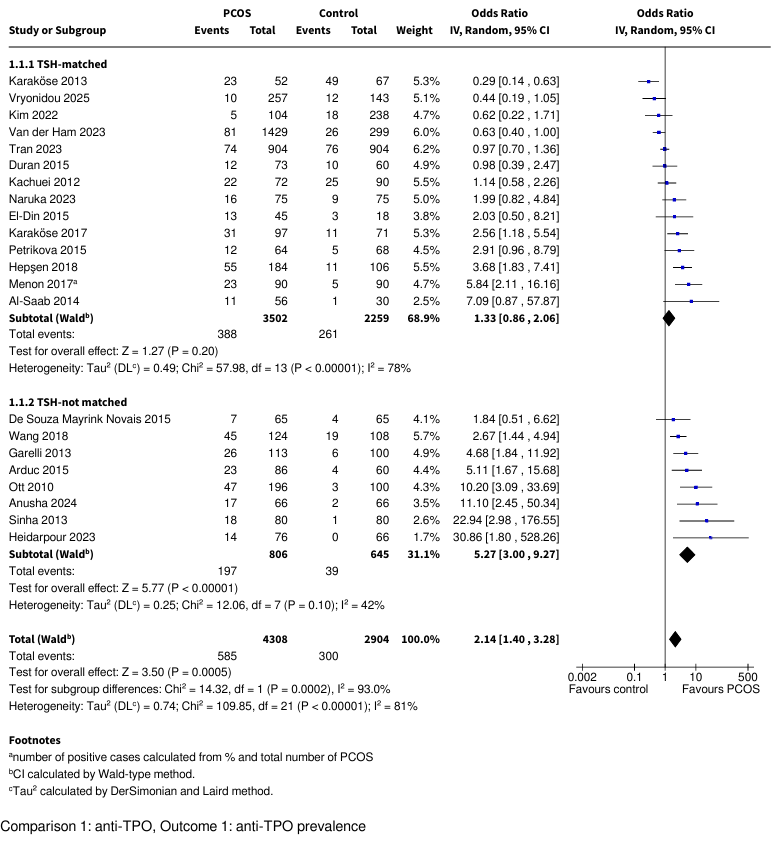

Supplement: Supplementary file 1 [file ijms-26-07525-s001.zip › Supplementary material S3 – sensitivity analyses anti-TPO prevalence/SUPPLEMENTARY MATERIAL S3E.png]

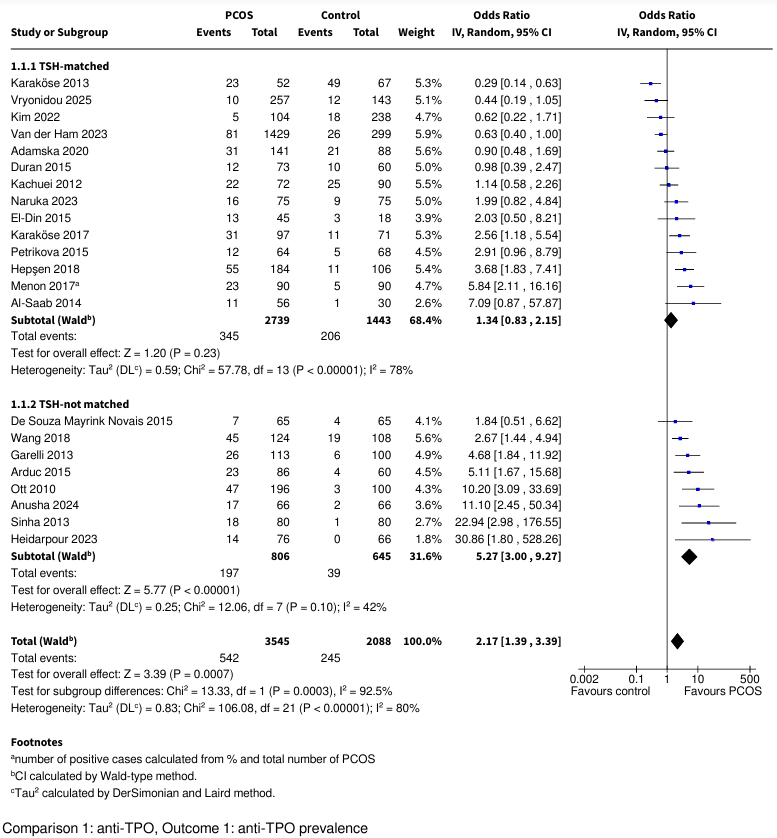

Supplement: Supplementary file 1 [file ijms-26-07525-s001.zip › Supplementary material S3 – sensitivity analyses anti-TPO prevalence/SUPPLEMENTARY MATERIAL S3F.png]

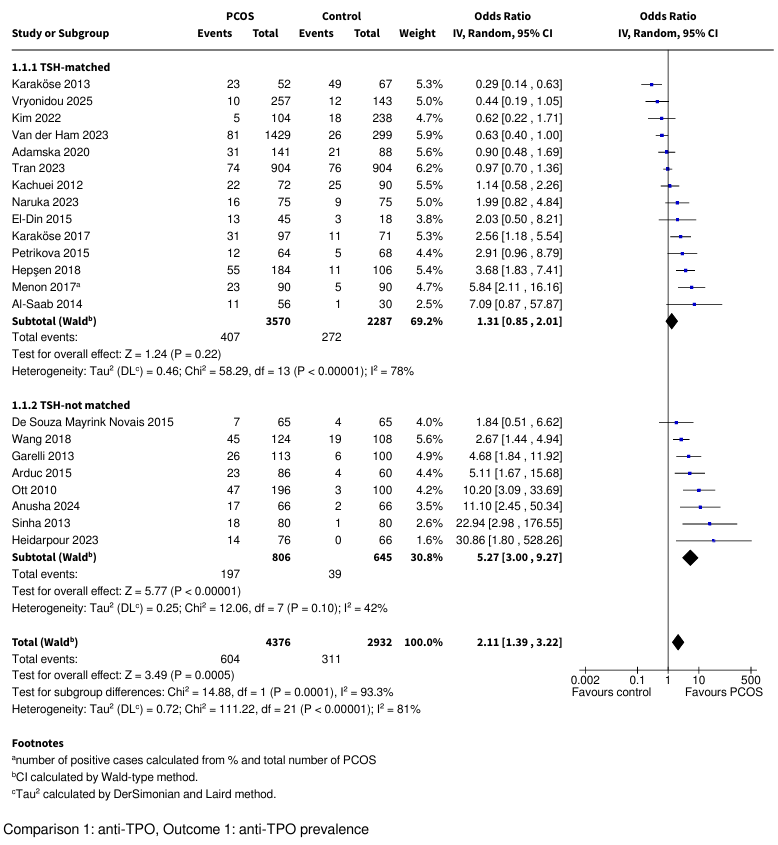

Supplement: Supplementary file 1 [file ijms-26-07525-s001.zip › Supplementary material S3 – sensitivity analyses anti-TPO prevalence/SUPPLEMENTARY MATERIAL S3G.png]

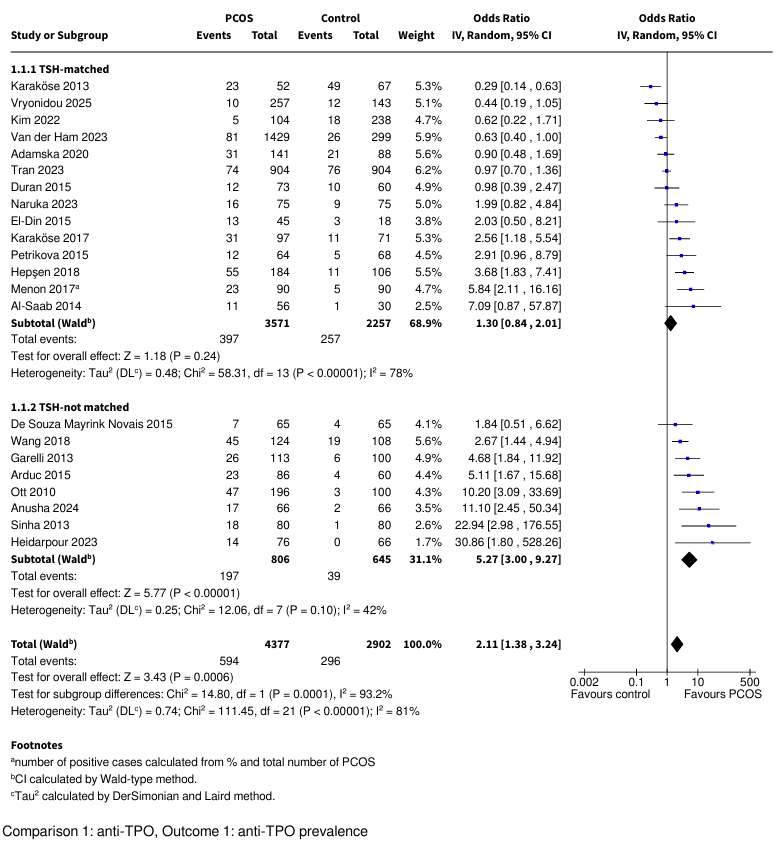

Supplement: Supplementary file 1 [file ijms-26-07525-s001.zip › Supplementary material S3 – sensitivity analyses anti-TPO prevalence/SUPPLEMENTARY MATERIAL S3H.png]

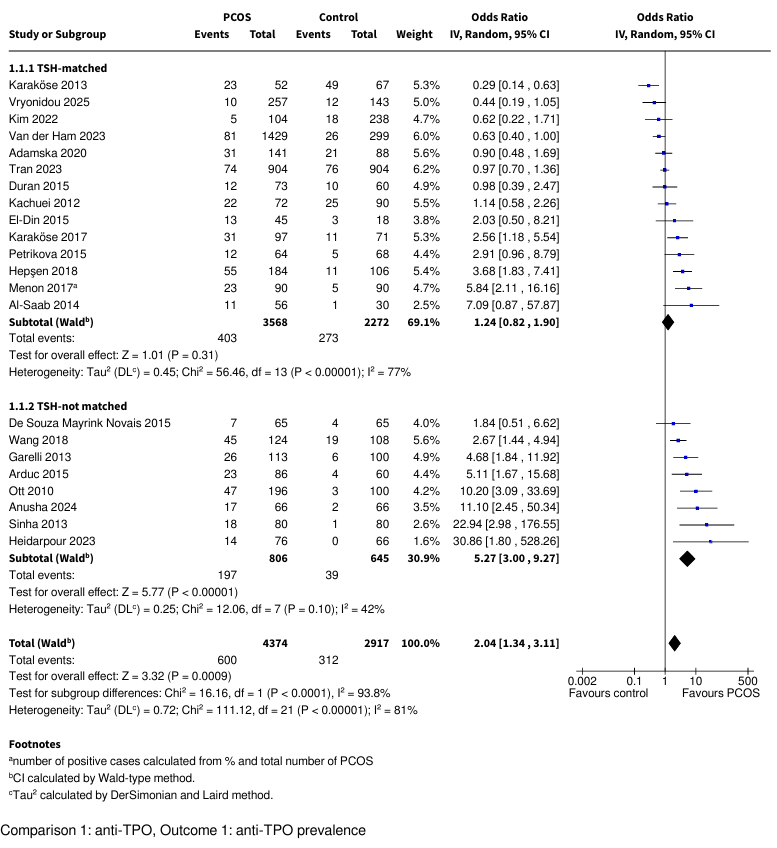

Supplement: Supplementary file 1 [file ijms-26-07525-s001.zip › Supplementary material S3 – sensitivity analyses anti-TPO prevalence/SUPPLEMENTARY MATERIAL S3I.png]

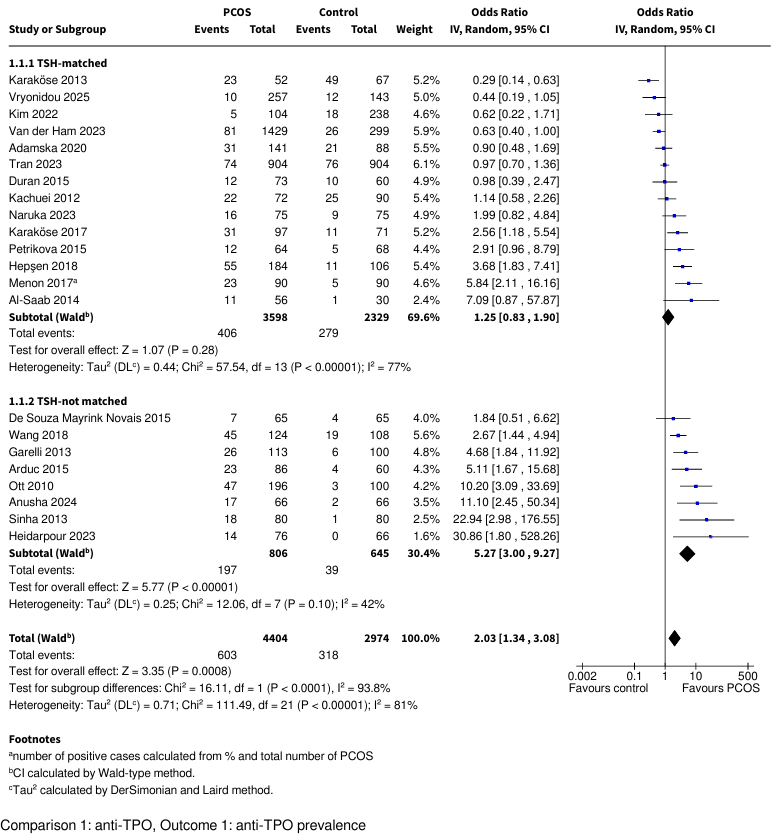

Supplement: Supplementary file 1 [file ijms-26-07525-s001.zip › Supplementary material S3 – sensitivity analyses anti-TPO prevalence/SUPPLEMENTARY MATERIAL S3J.png]

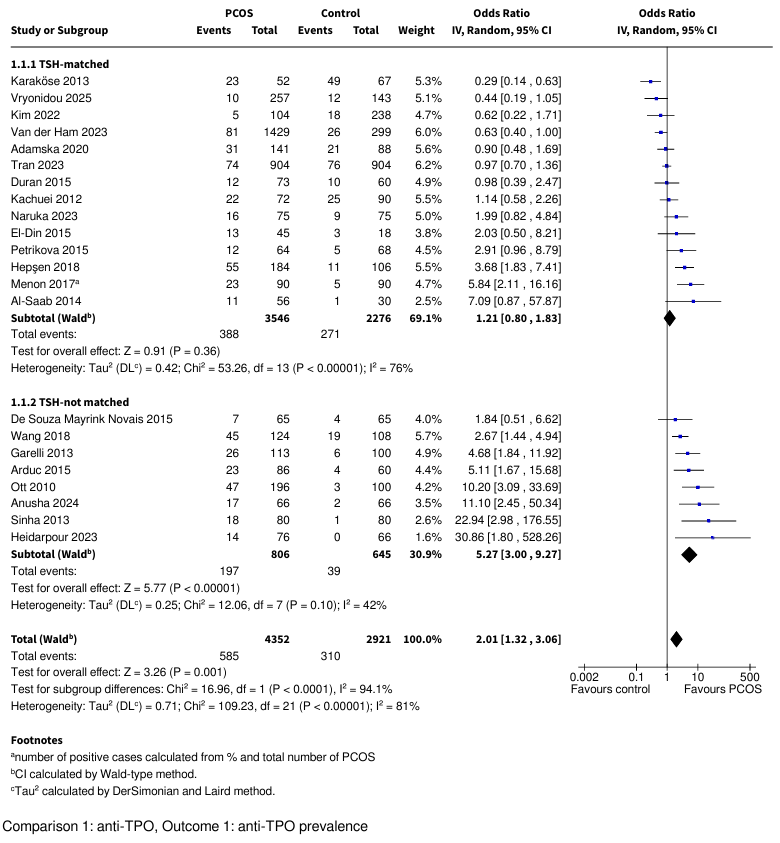

Supplement: Supplementary file 1 [file ijms-26-07525-s001.zip › Supplementary material S3 – sensitivity analyses anti-TPO prevalence/SUPPLEMENTARY MATERIAL S3K.png]

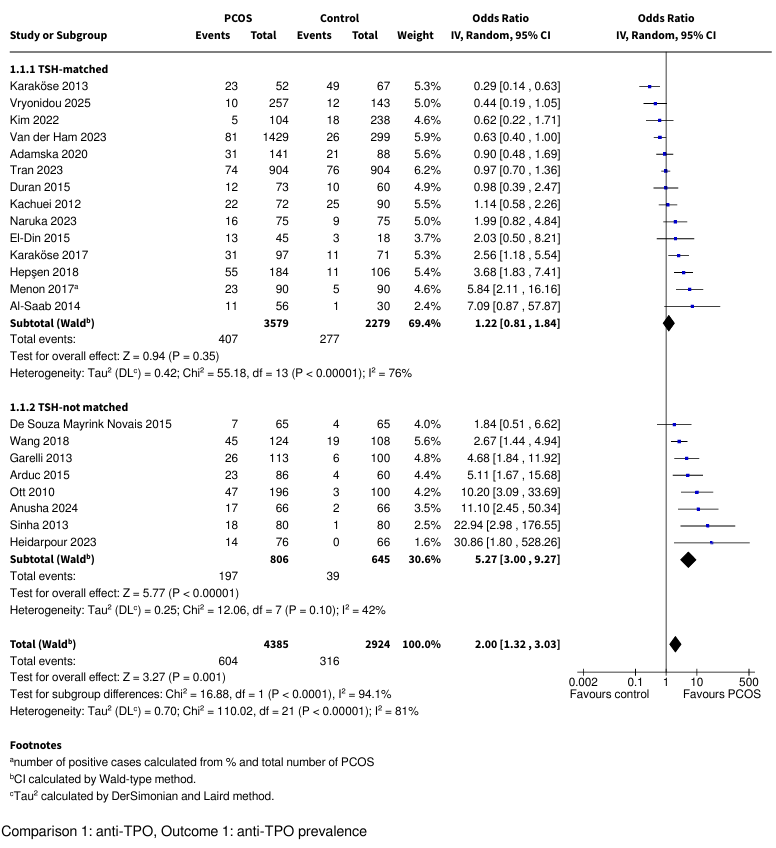

Supplement: Supplementary file 1 [file ijms-26-07525-s001.zip › Supplementary material S3 – sensitivity analyses anti-TPO prevalence/SUPPLEMENTARY MATERIAL S3L.png]

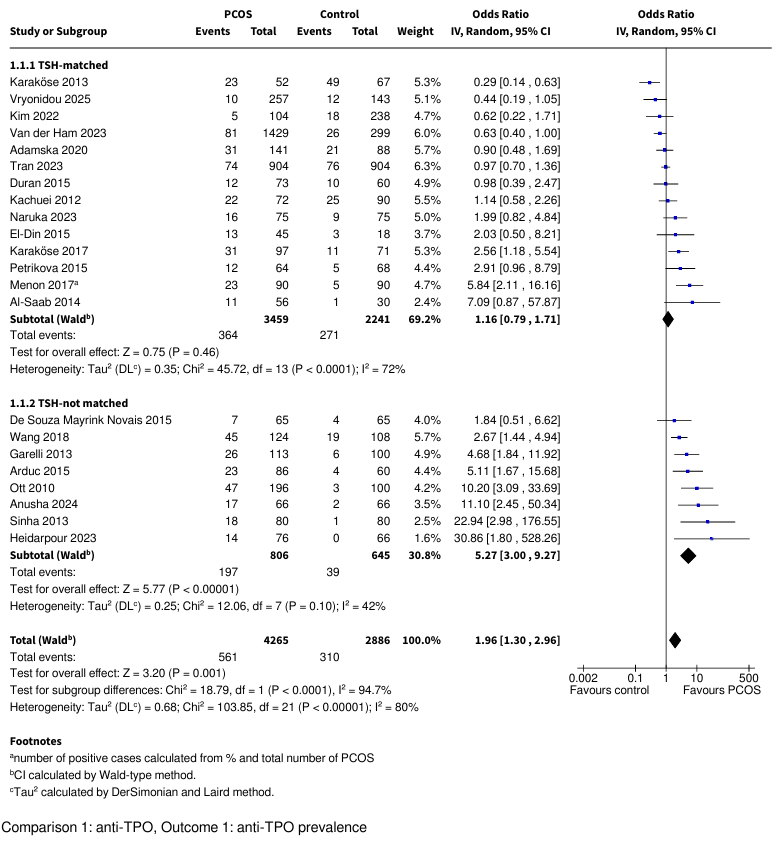

Supplement: Supplementary file 1 [file ijms-26-07525-s001.zip › Supplementary material S3 – sensitivity analyses anti-TPO prevalence/SUPPLEMENTARY MATERIAL S3M.png]

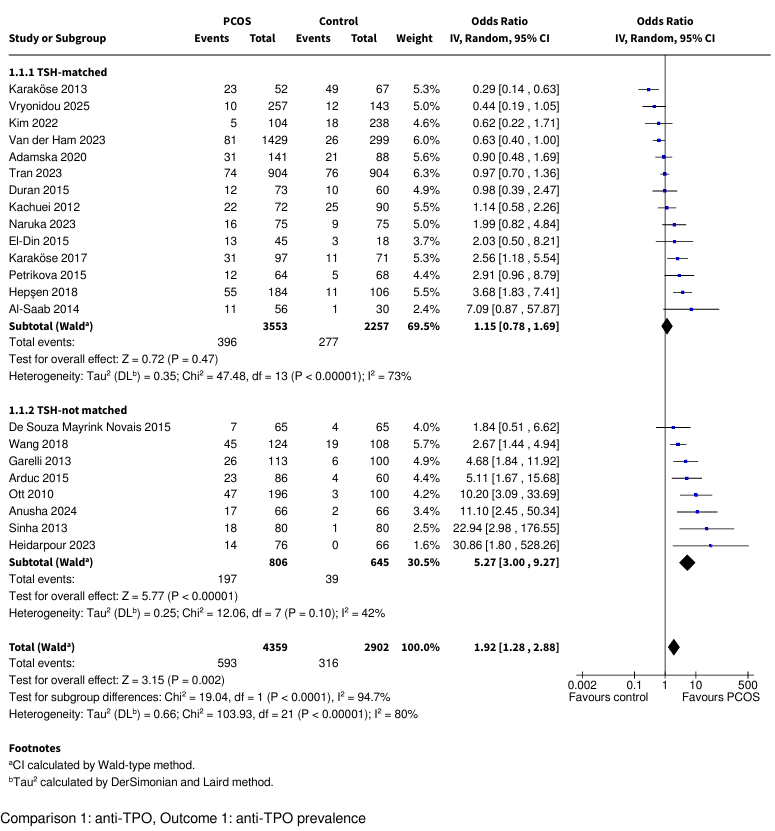

Supplement: Supplementary file 1 [file ijms-26-07525-s001.zip › Supplementary material S3 – sensitivity analyses anti-TPO prevalence/SUPPLEMENTARY MATERIAL S3N.png]

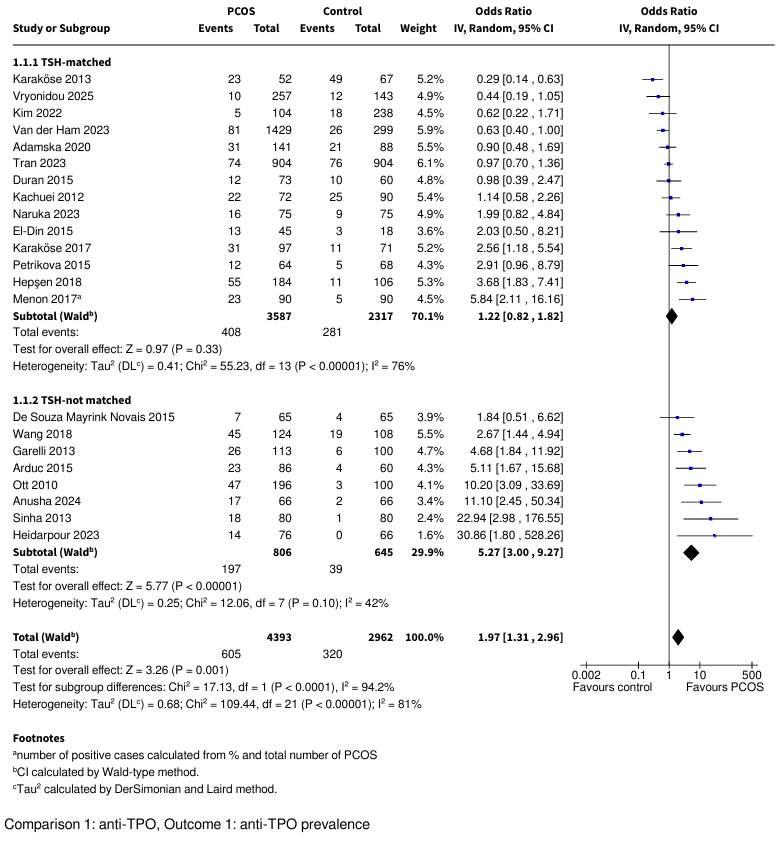

Supplement: Supplementary file 1 [file ijms-26-07525-s001.zip › Supplementary material S3 – sensitivity analyses anti-TPO prevalence/SUPPLEMENTARY MATERIAL S3O.png]

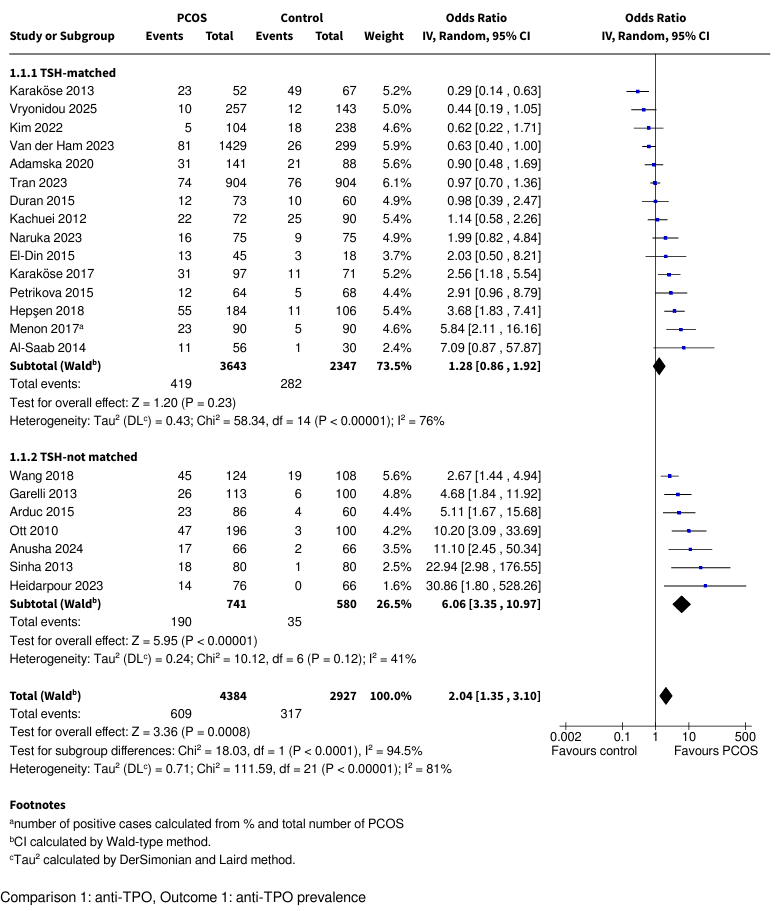

Supplement: Supplementary file 1 [file ijms-26-07525-s001.zip › Supplementary material S3 – sensitivity analyses anti-TPO prevalence/SUPPLEMENTARY MATERIAL S3P.png]

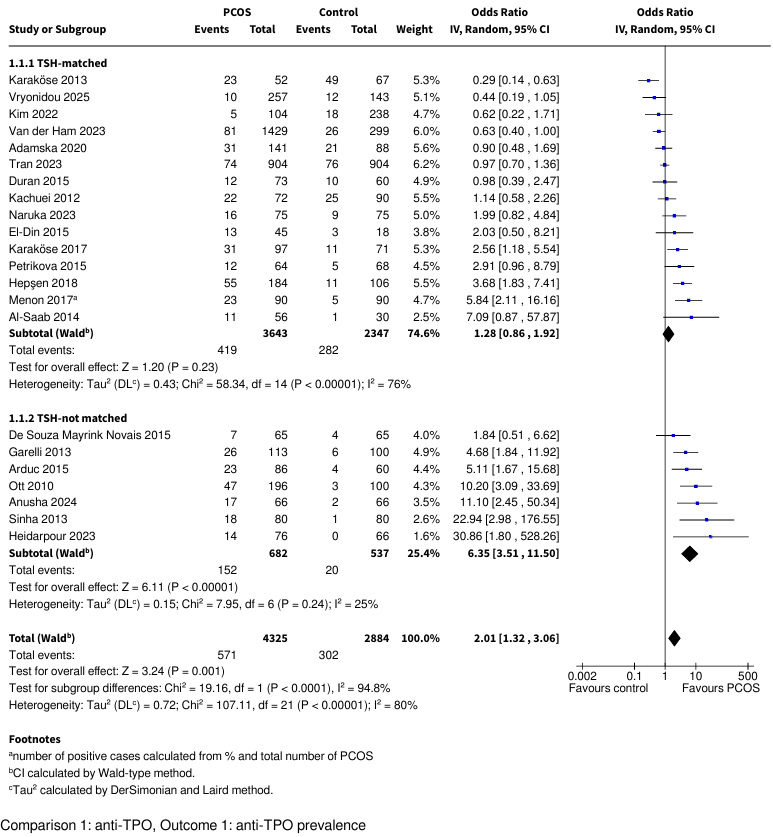

Supplement: Supplementary file 1 [file ijms-26-07525-s001.zip › Supplementary material S3 – sensitivity analyses anti-TPO prevalence/SUPPLEMENTARY MATERIAL S3Q.png]

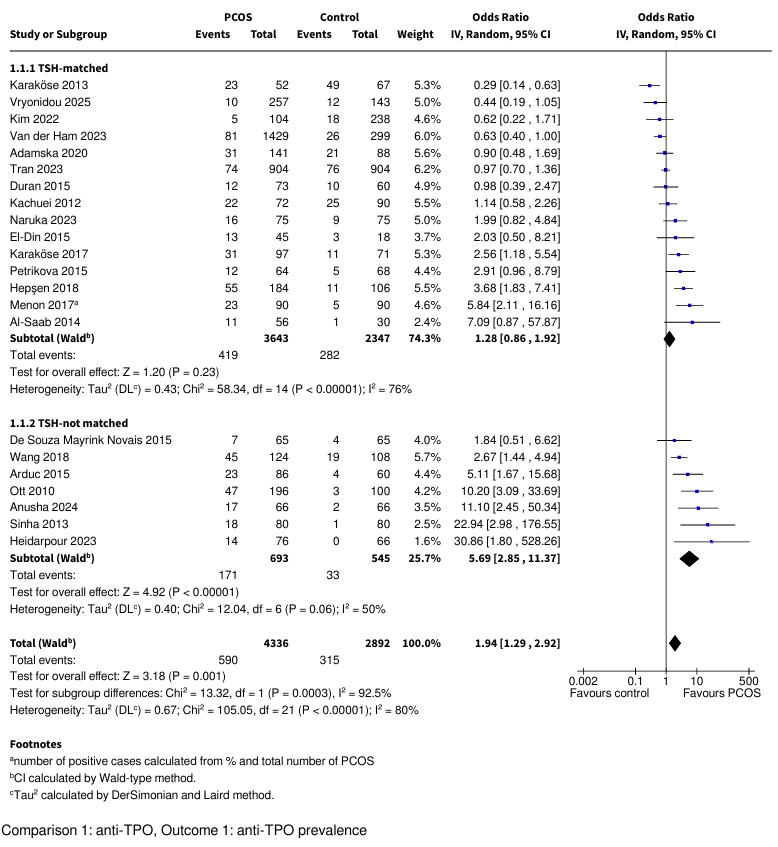

Supplement: Supplementary file 1 [file ijms-26-07525-s001.zip › Supplementary material S3 – sensitivity analyses anti-TPO prevalence/SUPPLEMENTARY MATERIAL S3R.png]

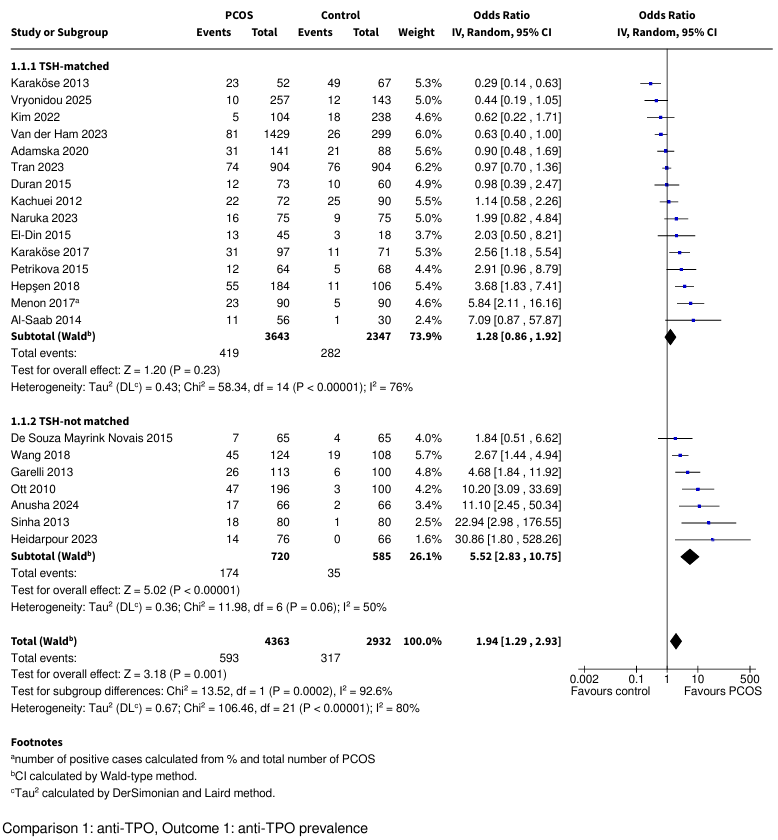

Supplement: Supplementary file 1 [file ijms-26-07525-s001.zip › Supplementary material S3 – sensitivity analyses anti-TPO prevalence/SUPPLEMENTARY MATERIAL S3S.png]

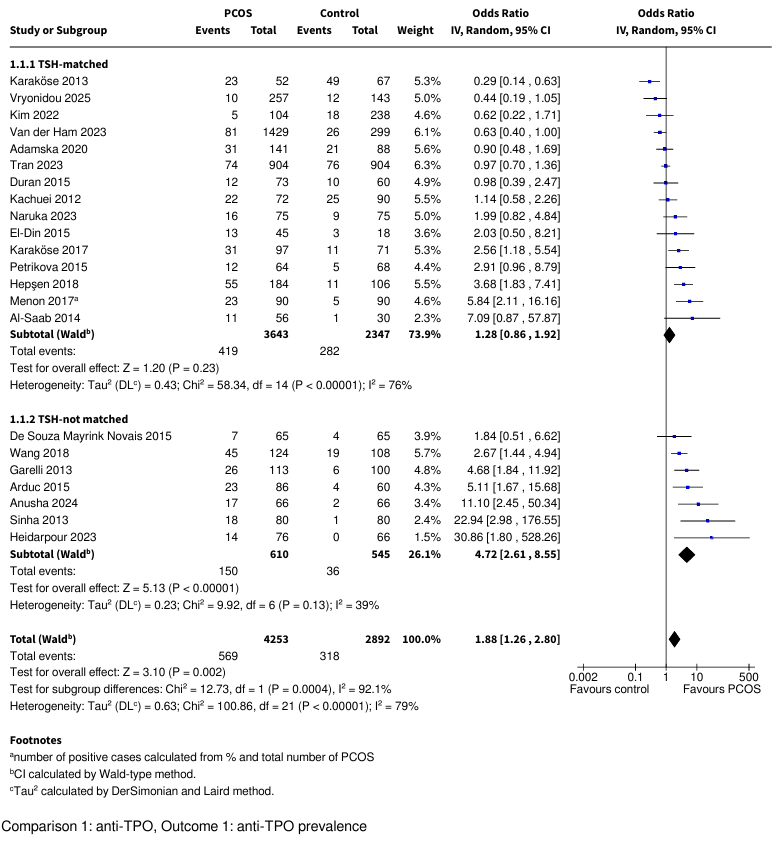

Supplement: Supplementary file 1 [file ijms-26-07525-s001.zip › Supplementary material S3 – sensitivity analyses anti-TPO prevalence/SUPPLEMENTARY MATERIAL S3T.png]

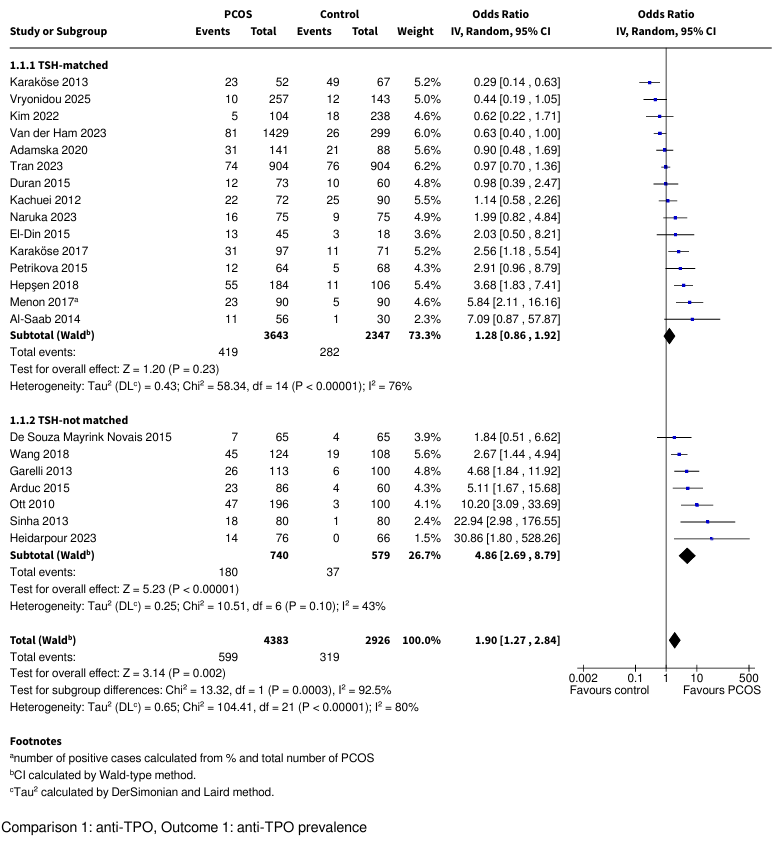

Supplement: Supplementary file 1 [file ijms-26-07525-s001.zip › Supplementary material S3 – sensitivity analyses anti-TPO prevalence/SUPPLEMENTARY MATERIAL S3U.png]

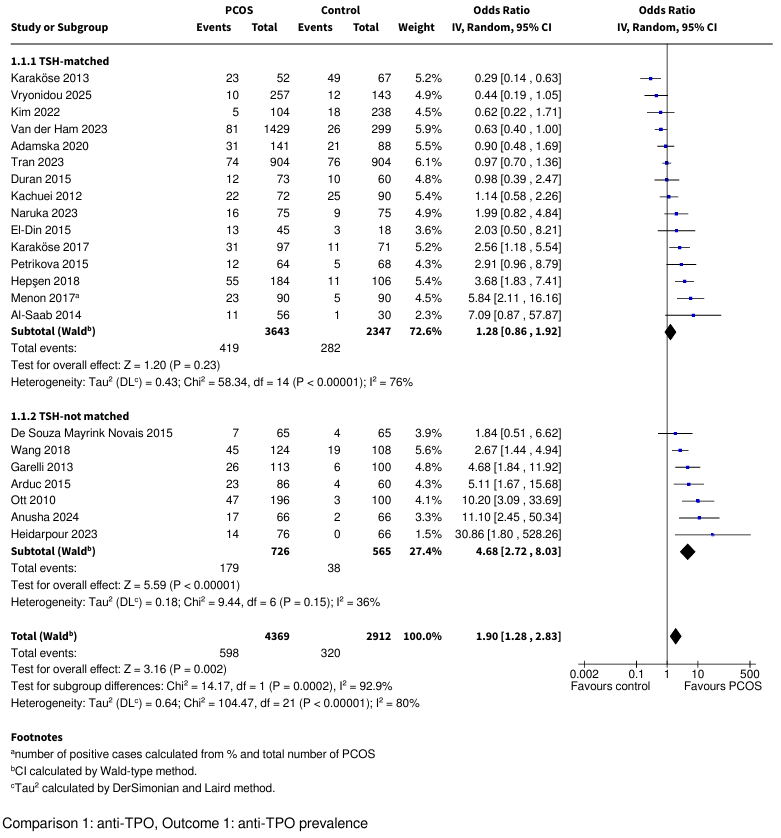

Supplement: Supplementary file 1 [file ijms-26-07525-s001.zip › Supplementary material S3 – sensitivity analyses anti-TPO prevalence/SUPPLEMENTARY MATERIAL S3V.png]

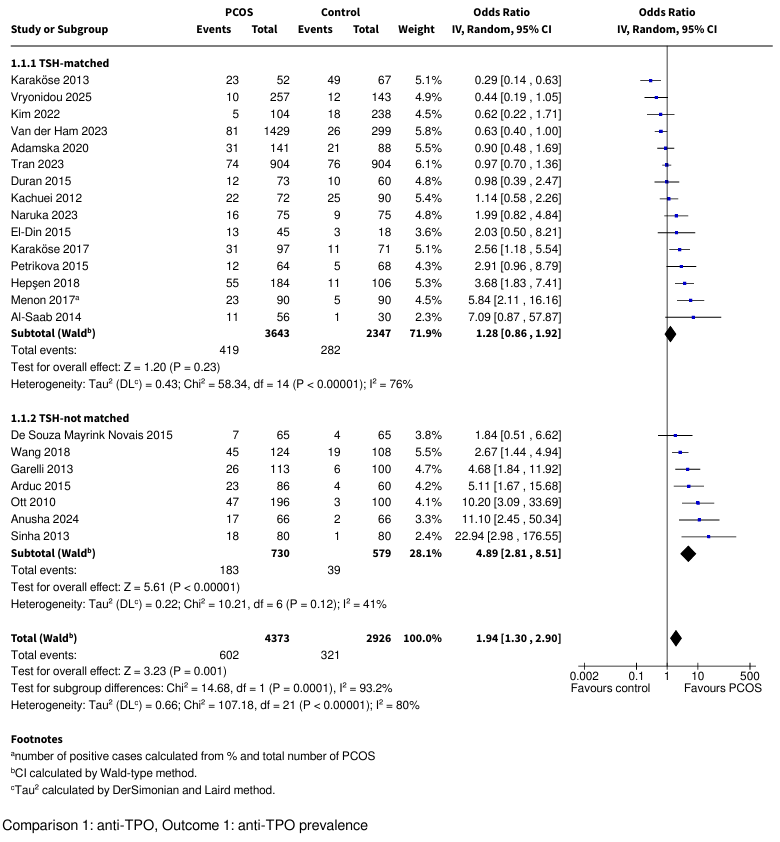

Supplement: Supplementary file 1 [file ijms-26-07525-s001.zip › Supplementary material S3 – sensitivity analyses anti-TPO prevalence/SUPPLEMENTARY MATERIAL S3W.png]

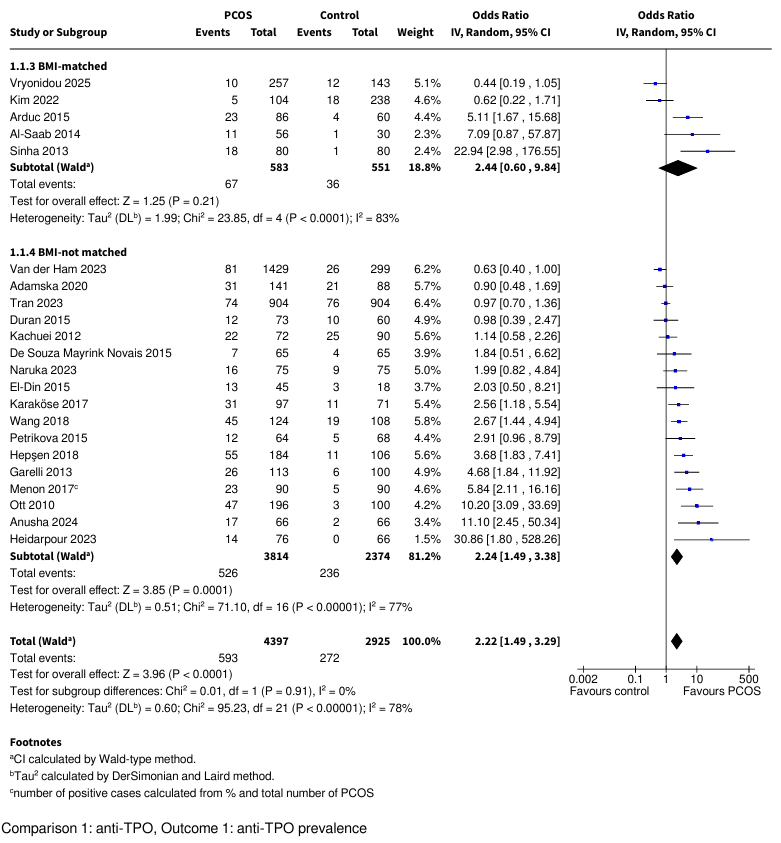

Supplement: Supplementary file 1 [file ijms-26-07525-s001.zip › Supplementary material S3 – sensitivity analyses anti-TPO prevalence/SUPPLEMENTARY MATERIAL S3X.png]

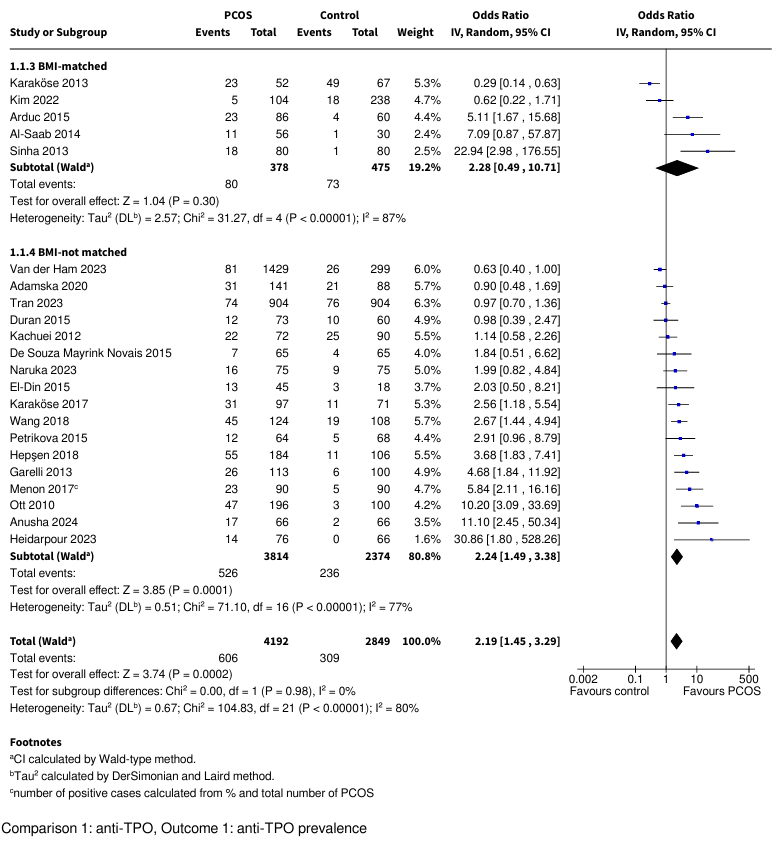

Supplement: Supplementary file 1 [file ijms-26-07525-s001.zip › Supplementary material S3 – sensitivity analyses anti-TPO prevalence/SUPPLEMENTARY MATERIAL S3Y.png]

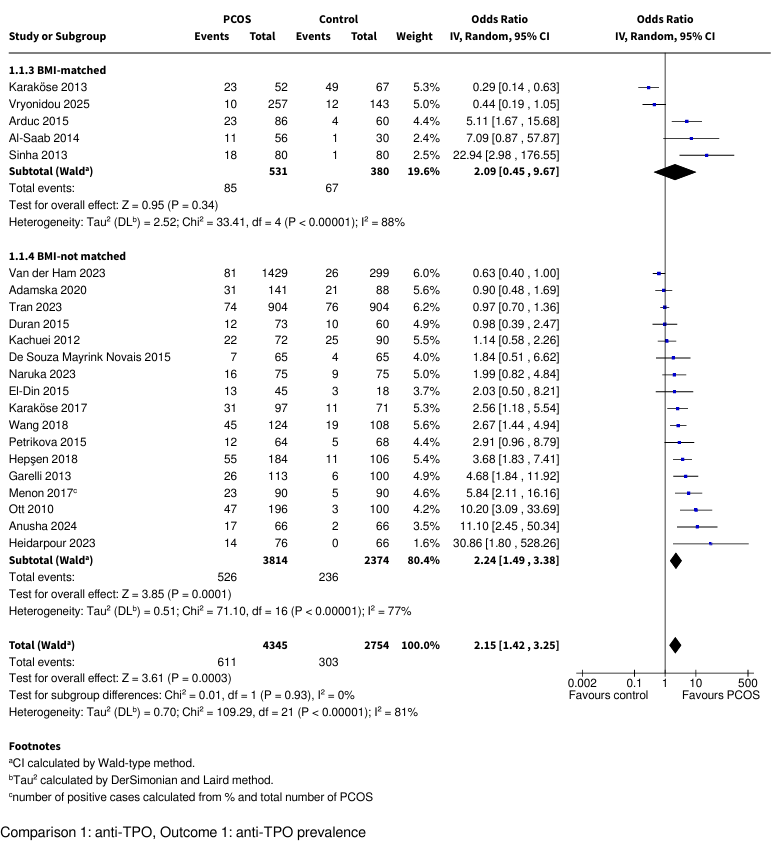

Supplement: Supplementary file 1 [file ijms-26-07525-s001.zip › Supplementary material S3 – sensitivity analyses anti-TPO prevalence/SUPPLEMENTARY MATERIAL S3Z.png]

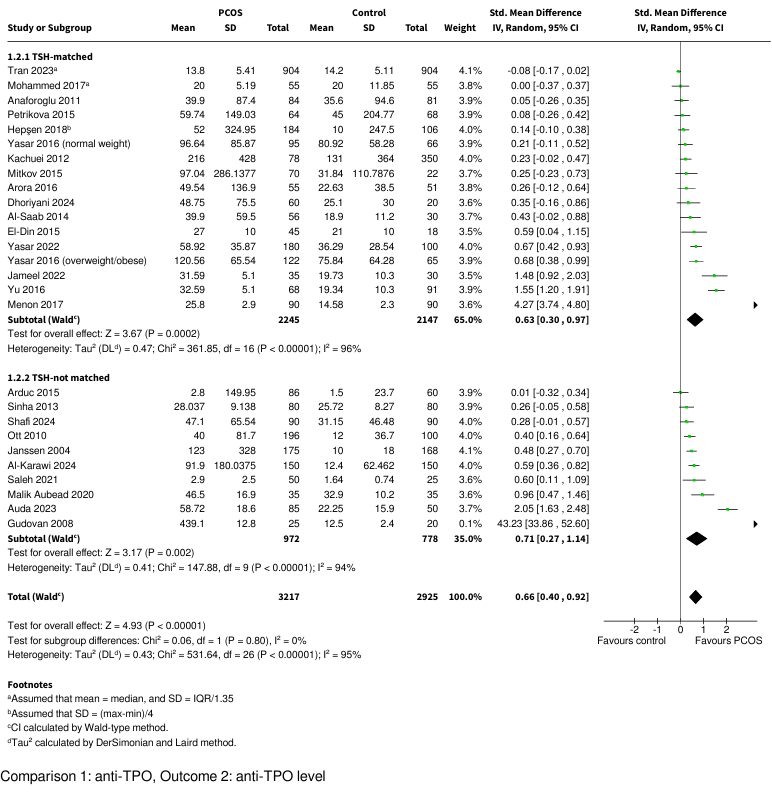

Supplement: Supplementary file 1 [file ijms-26-07525-s001.zip › Supplementary material S4 – sensitivity analyses anti-TPO level/SUPPLEMENTARY MATERIAL S4A.png]

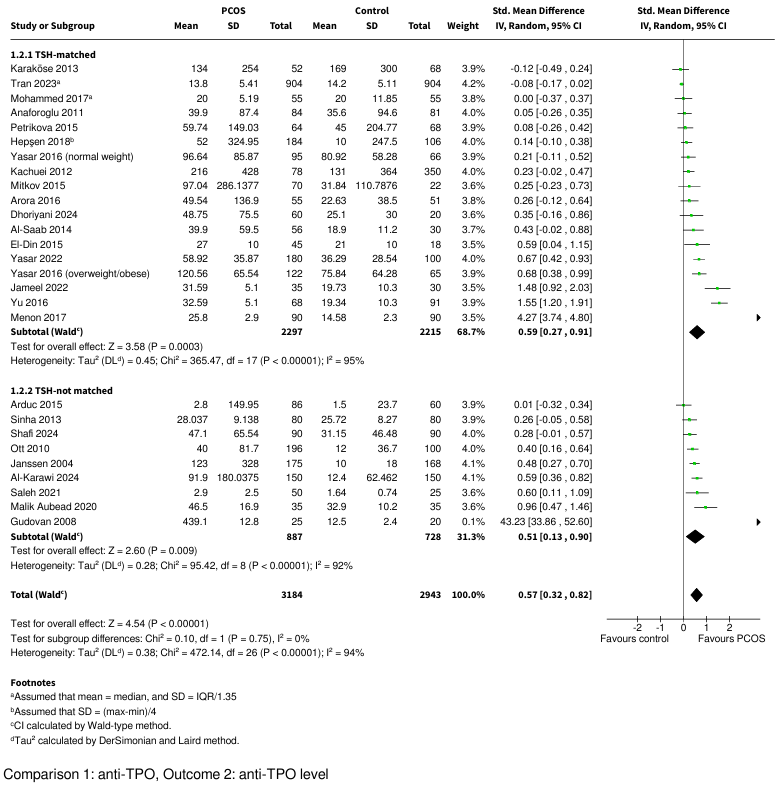

Supplement: Supplementary file 1 [file ijms-26-07525-s001.zip › Supplementary material S4 – sensitivity analyses anti-TPO level/SUPPLEMENTARY MATERIAL S4AA.png]

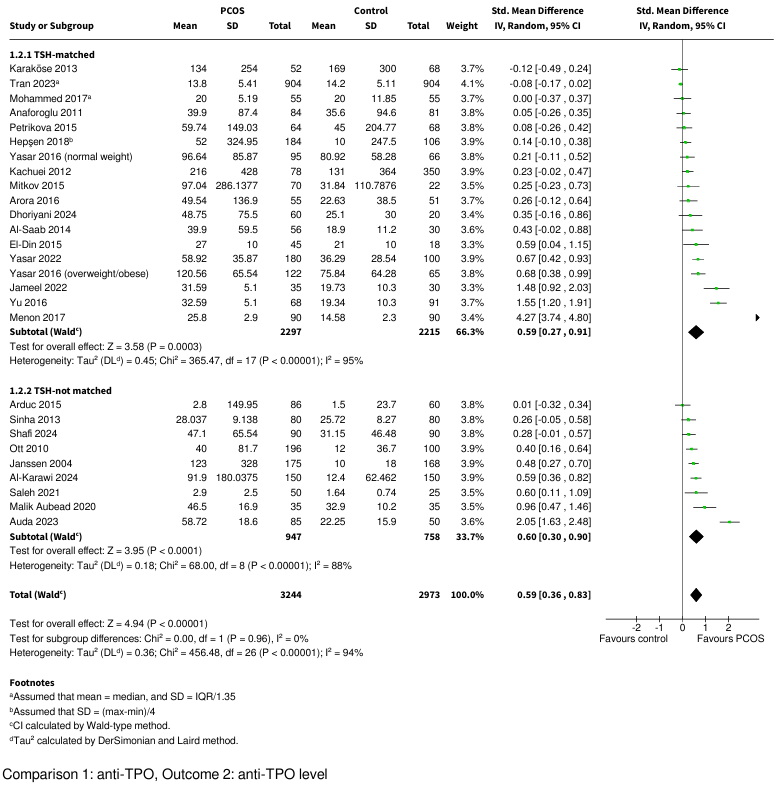

Supplement: Supplementary file 1 [file ijms-26-07525-s001.zip › Supplementary material S4 – sensitivity analyses anti-TPO level/SUPPLEMENTARY MATERIAL S4AB.png]

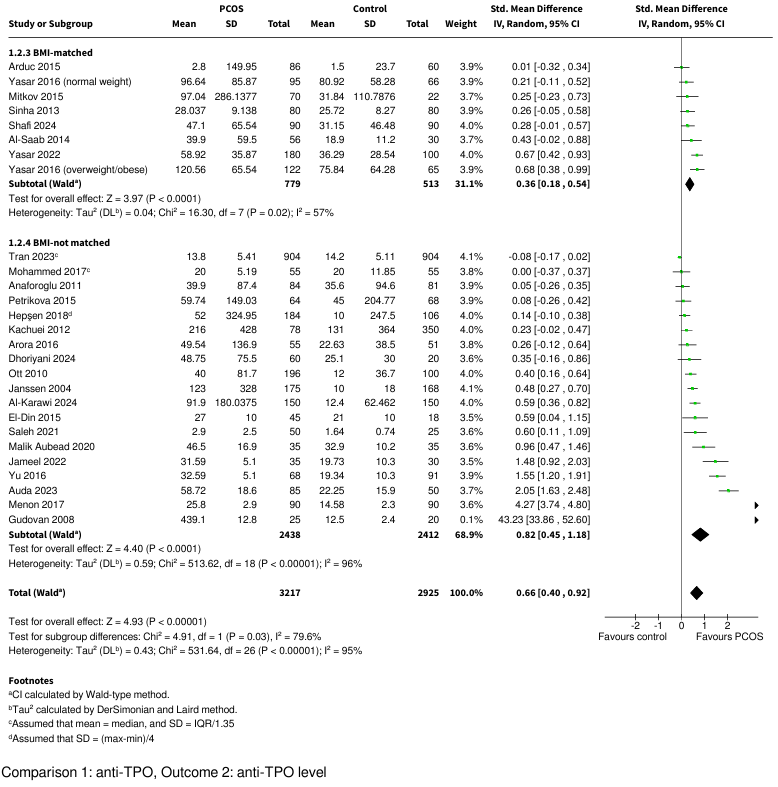

Supplement: Supplementary file 1 [file ijms-26-07525-s001.zip › Supplementary material S4 – sensitivity analyses anti-TPO level/SUPPLEMENTARY MATERIAL S4AC.png]

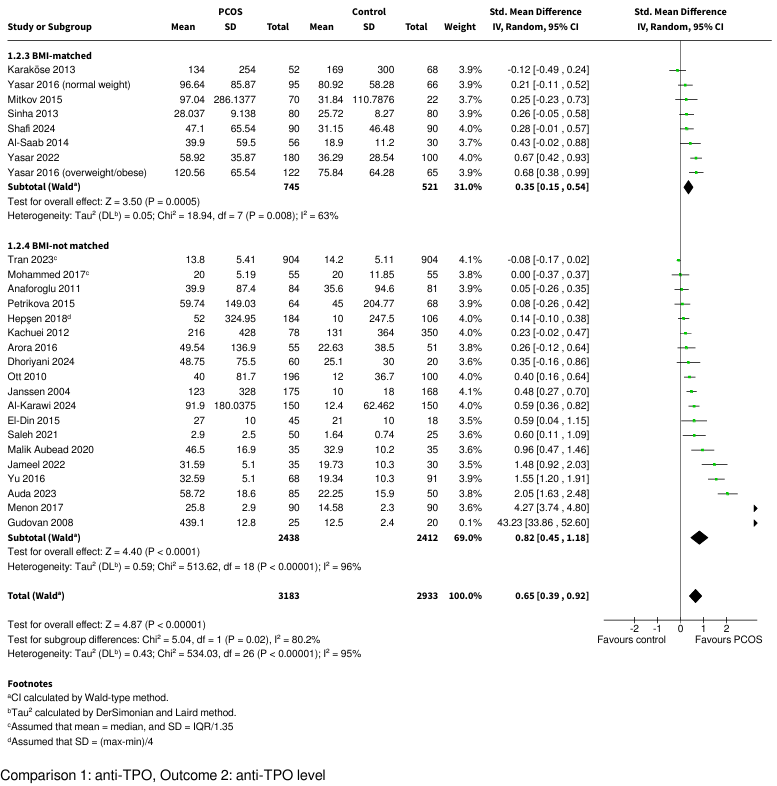

Supplement: Supplementary file 1 [file ijms-26-07525-s001.zip › Supplementary material S4 – sensitivity analyses anti-TPO level/SUPPLEMENTARY MATERIAL S4AD.png]

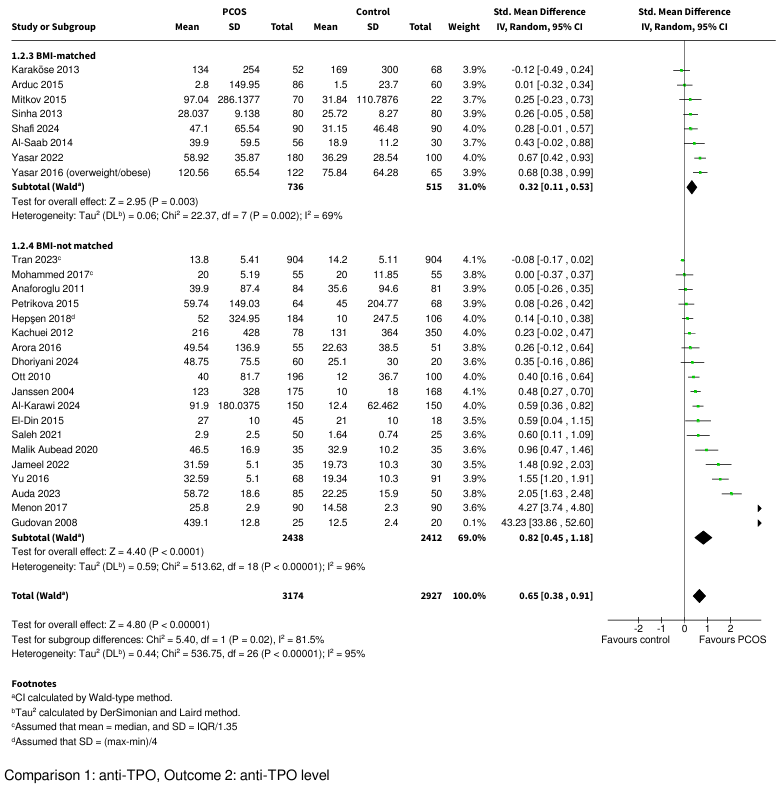

Supplement: Supplementary file 1 [file ijms-26-07525-s001.zip › Supplementary material S4 – sensitivity analyses anti-TPO level/SUPPLEMENTARY MATERIAL S4AE.png]

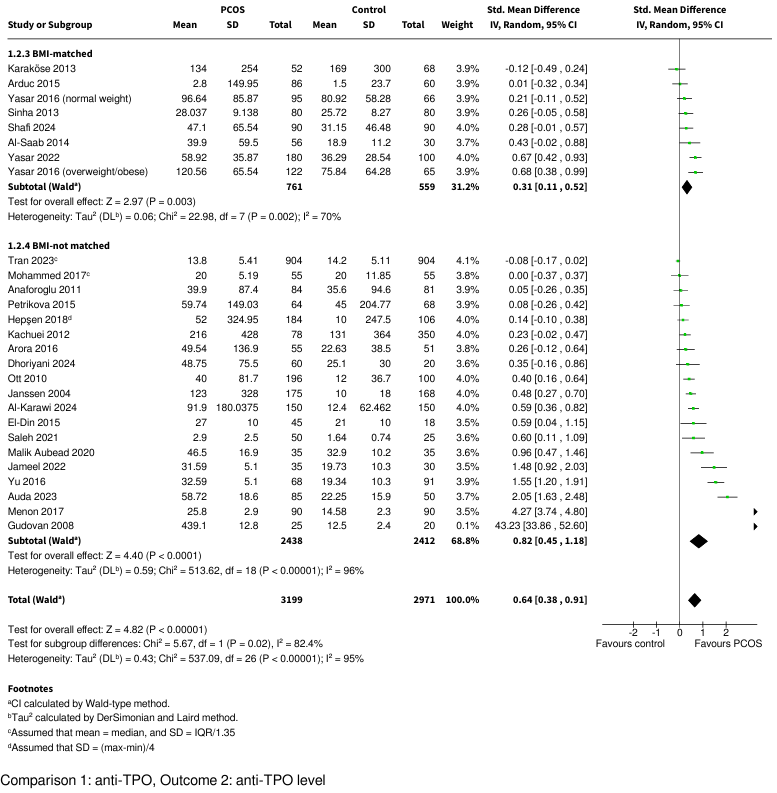

Supplement: Supplementary file 1 [file ijms-26-07525-s001.zip › Supplementary material S4 – sensitivity analyses anti-TPO level/SUPPLEMENTARY MATERIAL S4AF.png]

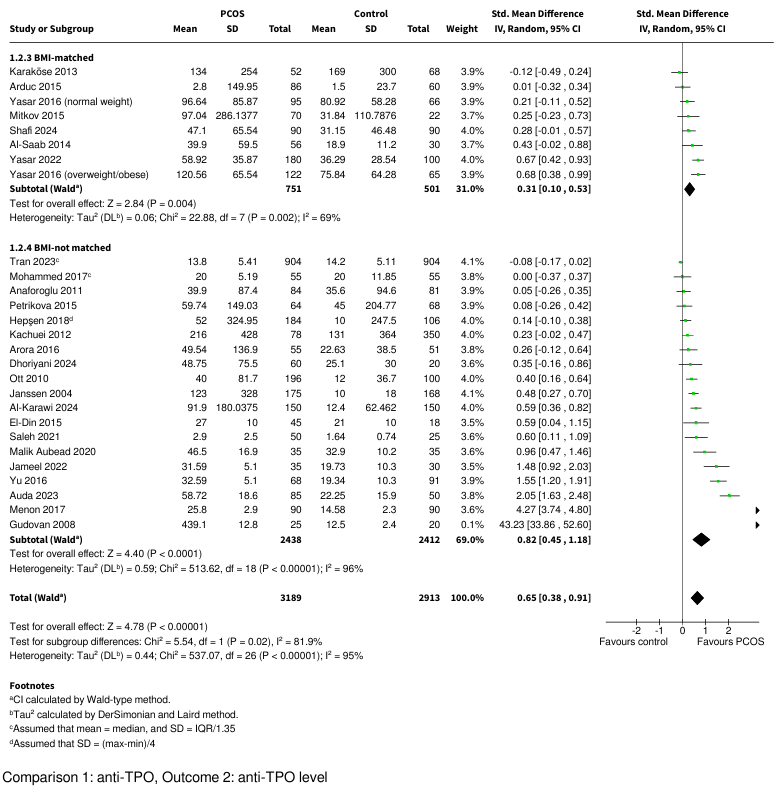

Supplement: Supplementary file 1 [file ijms-26-07525-s001.zip › Supplementary material S4 – sensitivity analyses anti-TPO level/SUPPLEMENTARY MATERIAL S4AG.png]

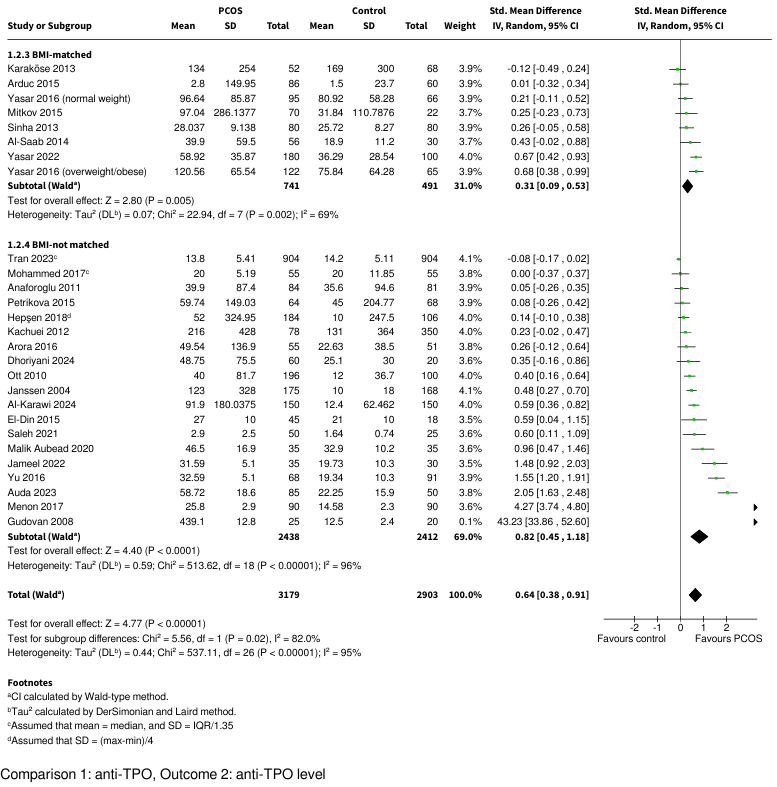

Supplement: Supplementary file 1 [file ijms-26-07525-s001.zip › Supplementary material S4 – sensitivity analyses anti-TPO level/SUPPLEMENTARY MATERIAL S4AH.png]

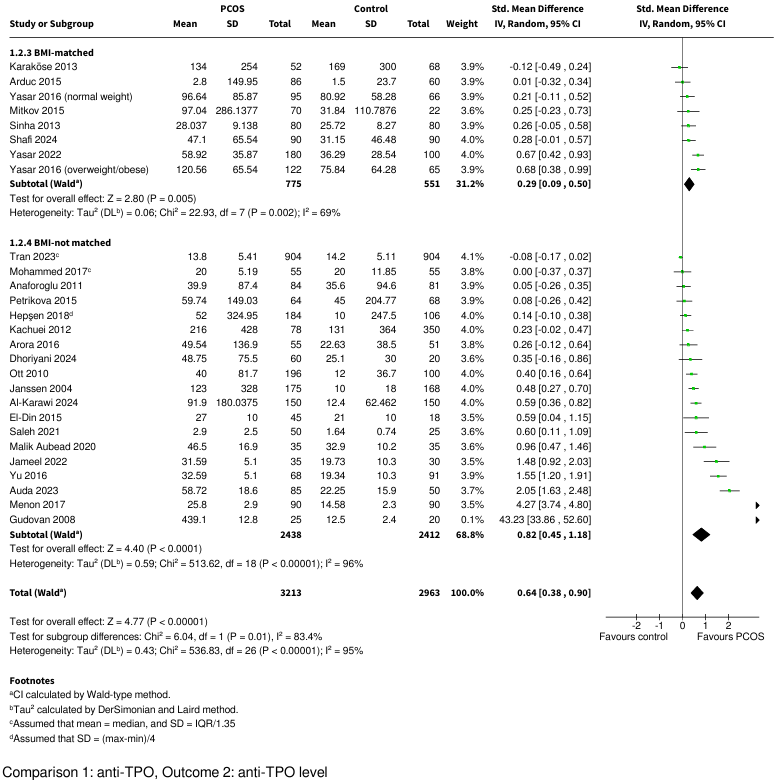

Supplement: Supplementary file 1 [file ijms-26-07525-s001.zip › Supplementary material S4 – sensitivity analyses anti-TPO level/SUPPLEMENTARY MATERIAL S4AI.png]

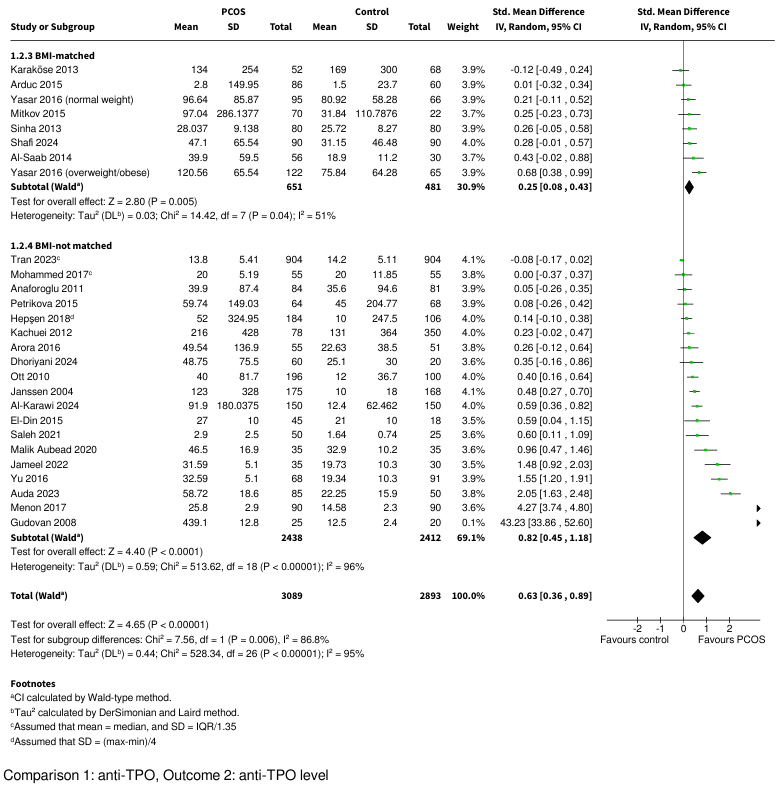

Supplement: Supplementary file 1 [file ijms-26-07525-s001.zip › Supplementary material S4 – sensitivity analyses anti-TPO level/SUPPLEMENTARY MATERIAL S4AJ.png]

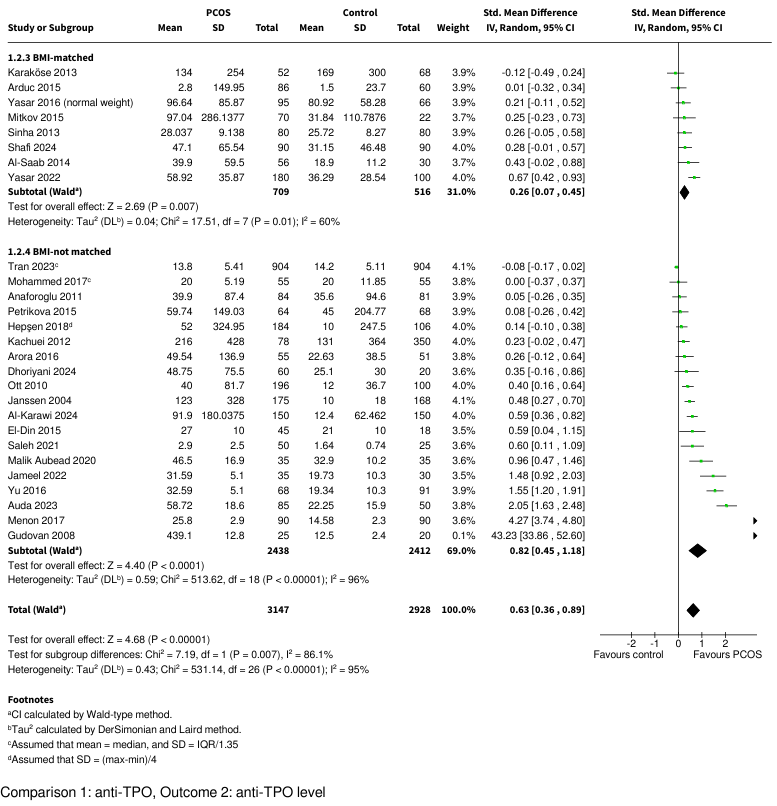

Supplement: Supplementary file 1 [file ijms-26-07525-s001.zip › Supplementary material S4 – sensitivity analyses anti-TPO level/SUPPLEMENTARY MATERIAL S4AK.png]

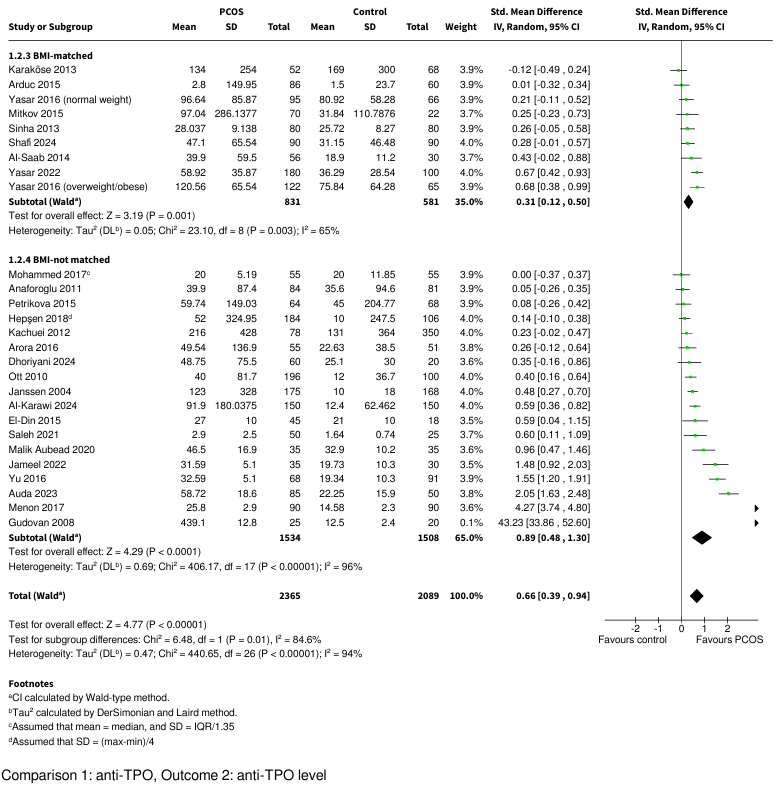

Supplement: Supplementary file 1 [file ijms-26-07525-s001.zip › Supplementary material S4 – sensitivity analyses anti-TPO level/SUPPLEMENTARY MATERIAL S4AL.png]

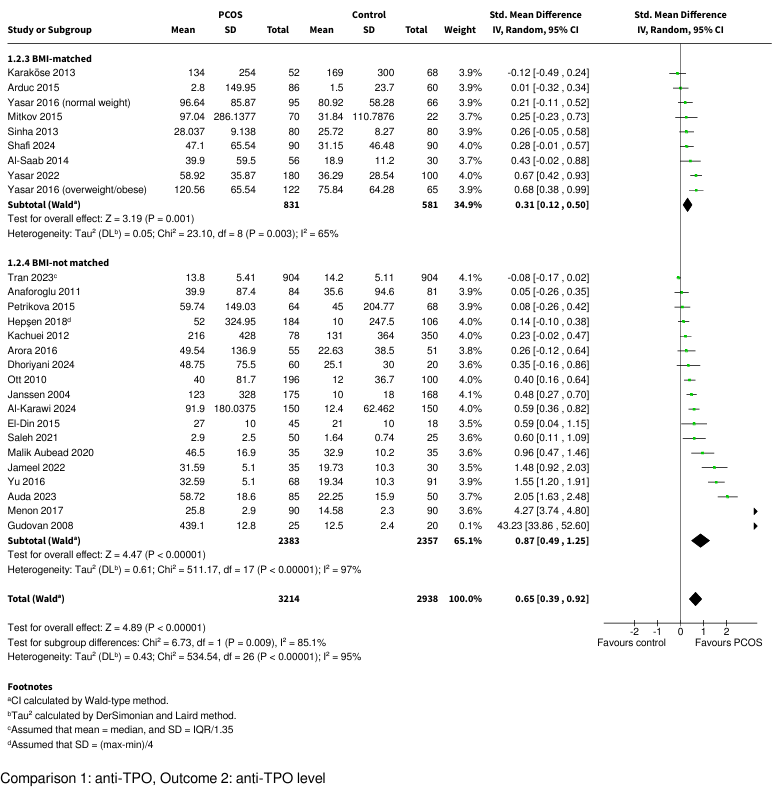

Supplement: Supplementary file 1 [file ijms-26-07525-s001.zip › Supplementary material S4 – sensitivity analyses anti-TPO level/SUPPLEMENTARY MATERIAL S4AM.png]

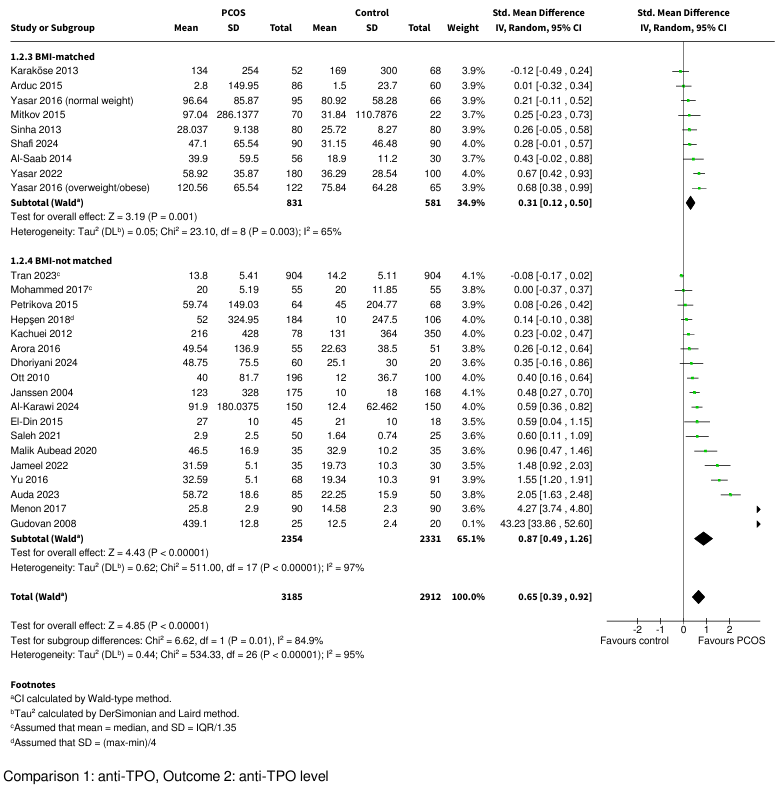

Supplement: Supplementary file 1 [file ijms-26-07525-s001.zip › Supplementary material S4 – sensitivity analyses anti-TPO level/SUPPLEMENTARY MATERIAL S4AN.png]

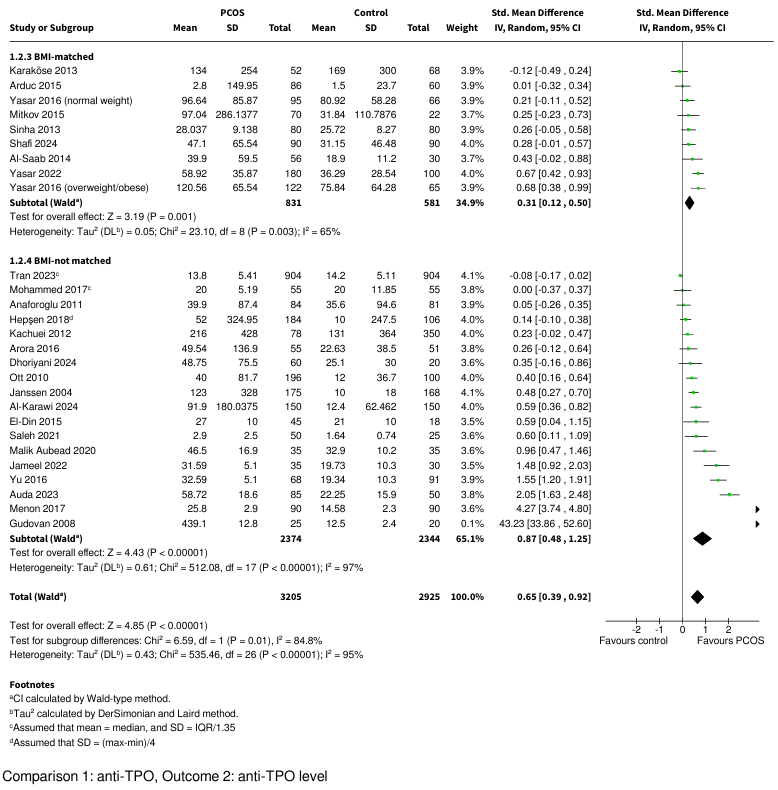

Supplement: Supplementary file 1 [file ijms-26-07525-s001.zip › Supplementary material S4 – sensitivity analyses anti-TPO level/SUPPLEMENTARY MATERIAL S4AO.png]

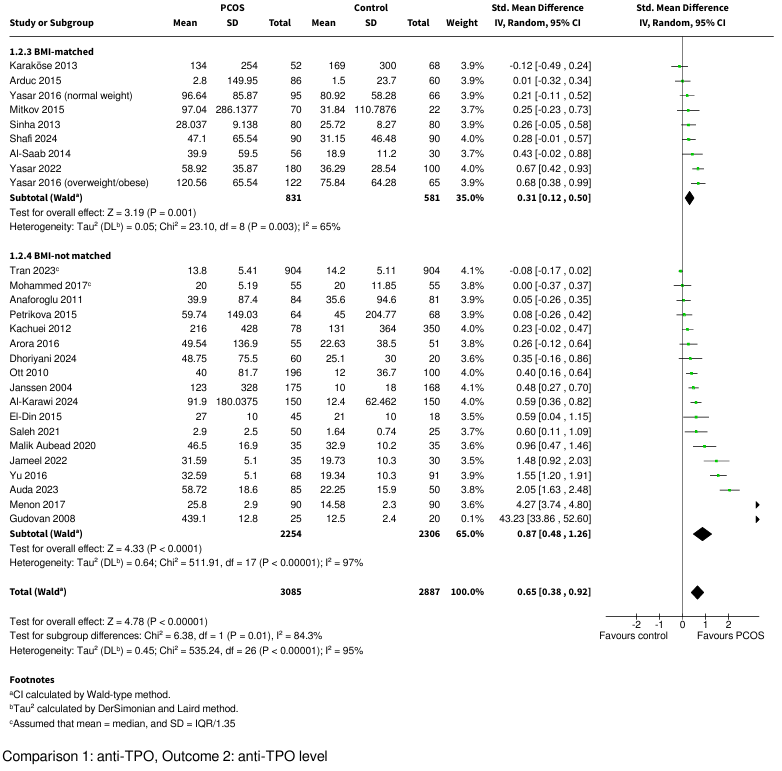

Supplement: Supplementary file 1 [file ijms-26-07525-s001.zip › Supplementary material S4 – sensitivity analyses anti-TPO level/SUPPLEMENTARY MATERIAL S4AP.png]

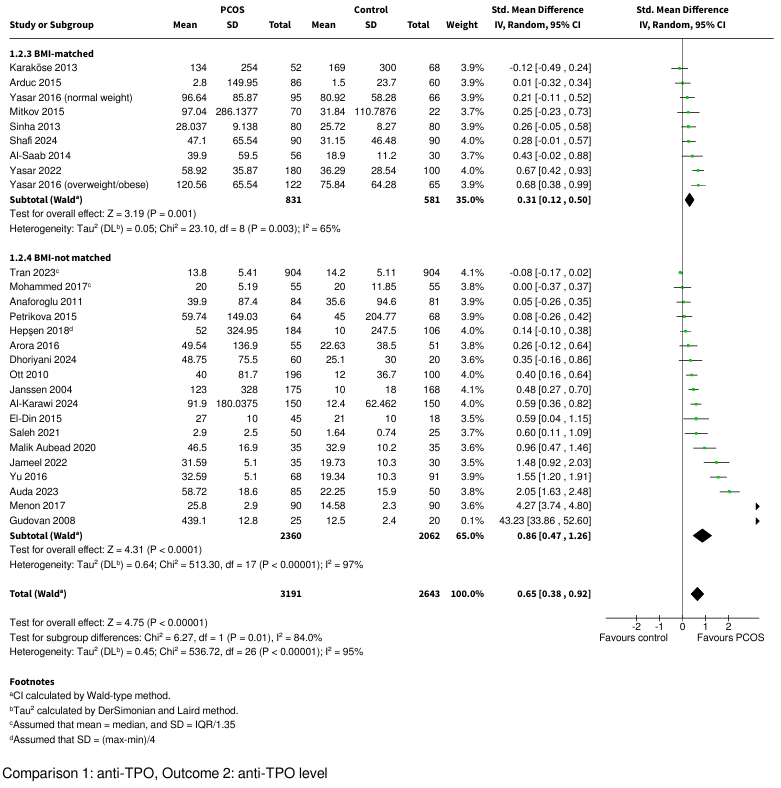

Supplement: Supplementary file 1 [file ijms-26-07525-s001.zip › Supplementary material S4 – sensitivity analyses anti-TPO level/SUPPLEMENTARY MATERIAL S4AQ.png]

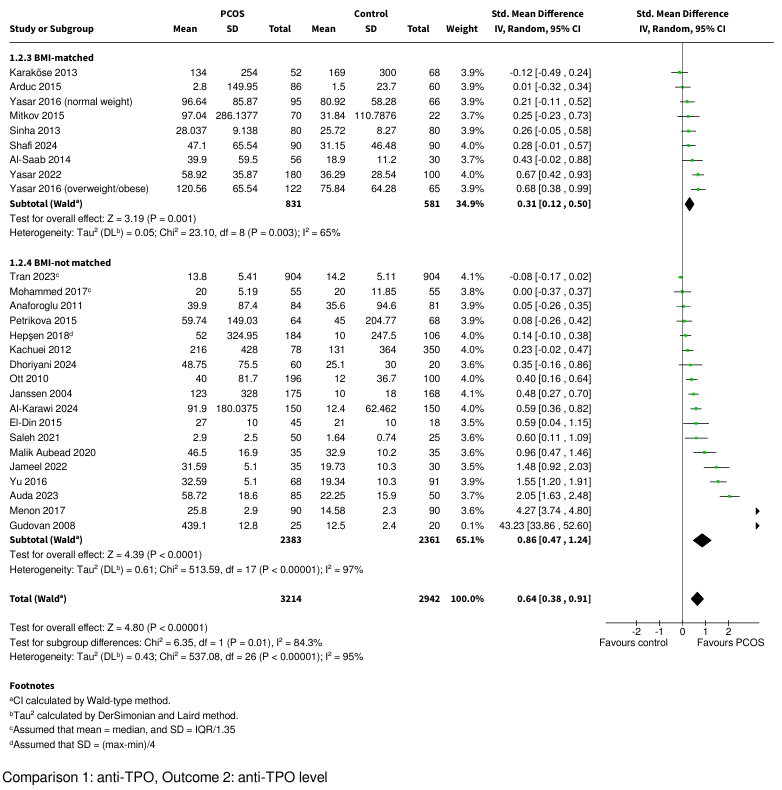

Supplement: Supplementary file 1 [file ijms-26-07525-s001.zip › Supplementary material S4 – sensitivity analyses anti-TPO level/SUPPLEMENTARY MATERIAL S4AR.png]

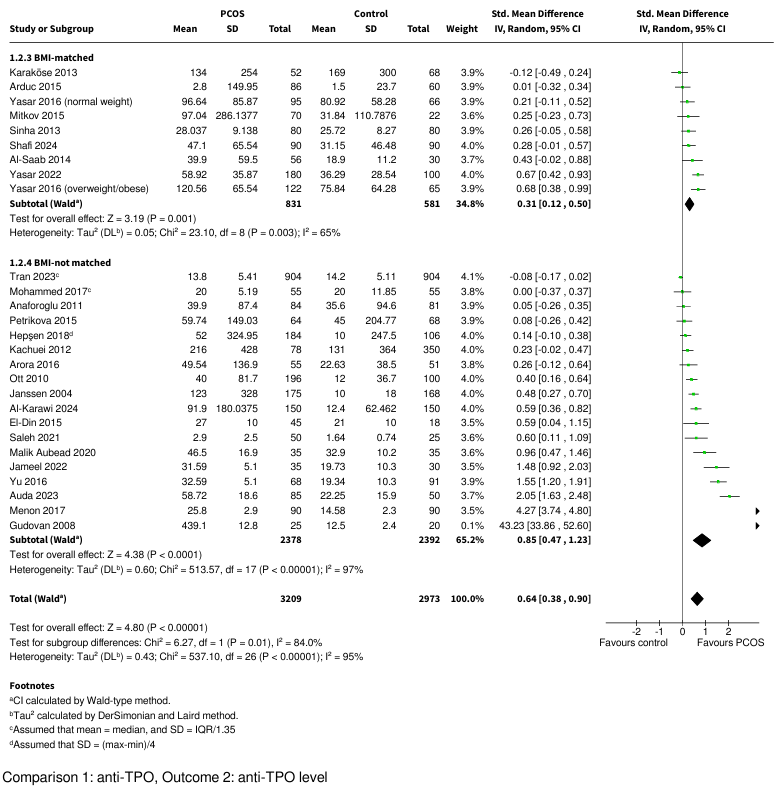

Supplement: Supplementary file 1 [file ijms-26-07525-s001.zip › Supplementary material S4 – sensitivity analyses anti-TPO level/SUPPLEMENTARY MATERIAL S4AS.png]

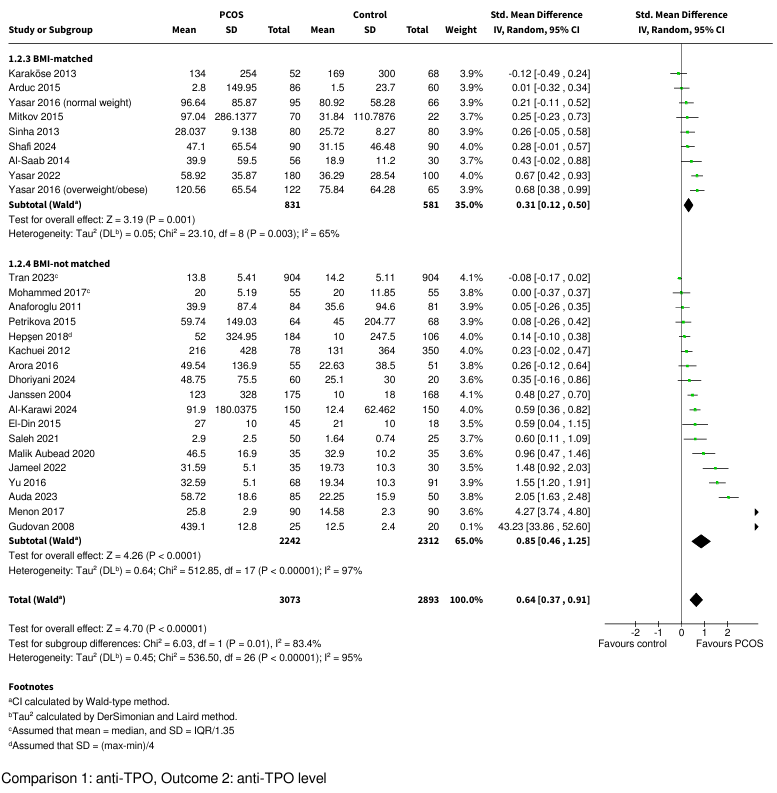

Supplement: Supplementary file 1 [file ijms-26-07525-s001.zip › Supplementary material S4 – sensitivity analyses anti-TPO level/SUPPLEMENTARY MATERIAL S4AT.png]

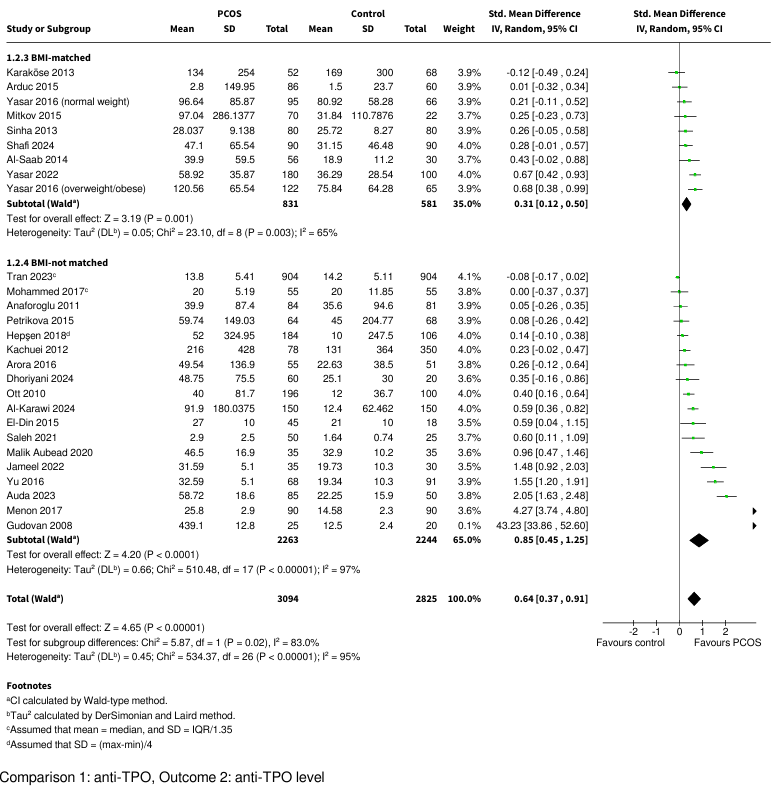

Supplement: Supplementary file 1 [file ijms-26-07525-s001.zip › Supplementary material S4 – sensitivity analyses anti-TPO level/SUPPLEMENTARY MATERIAL S4AU.png]

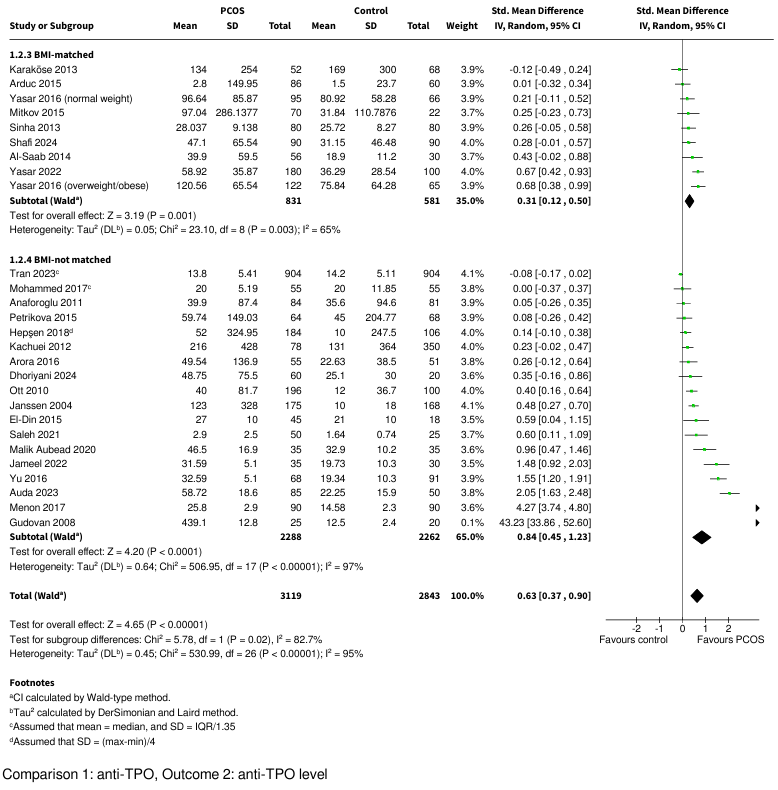

Supplement: Supplementary file 1 [file ijms-26-07525-s001.zip › Supplementary material S4 – sensitivity analyses anti-TPO level/SUPPLEMENTARY MATERIAL S4AV.png]

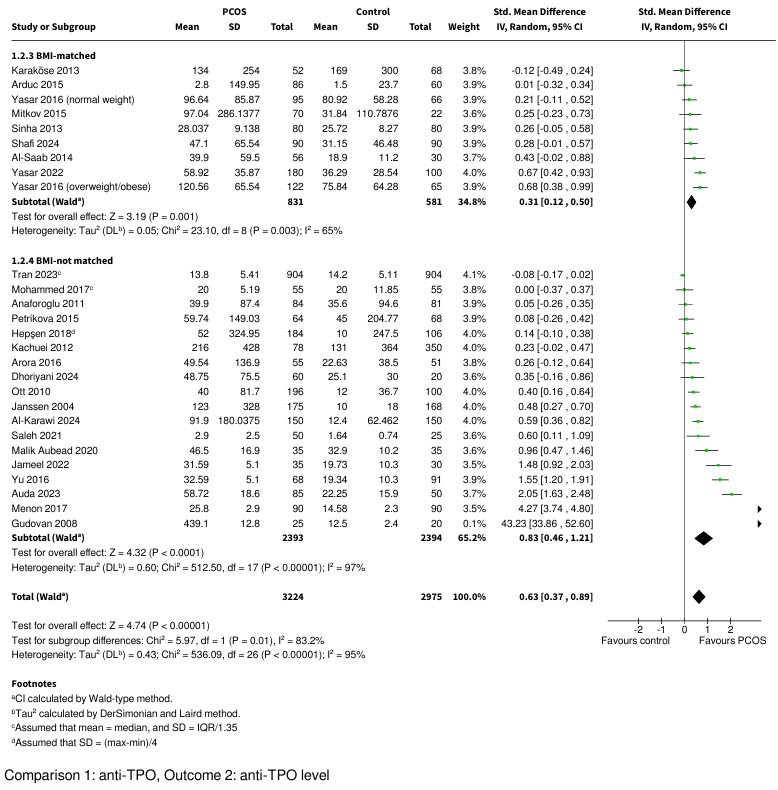

Supplement: Supplementary file 1 [file ijms-26-07525-s001.zip › Supplementary material S4 – sensitivity analyses anti-TPO level/SUPPLEMENTARY MATERIAL S4AW.png]

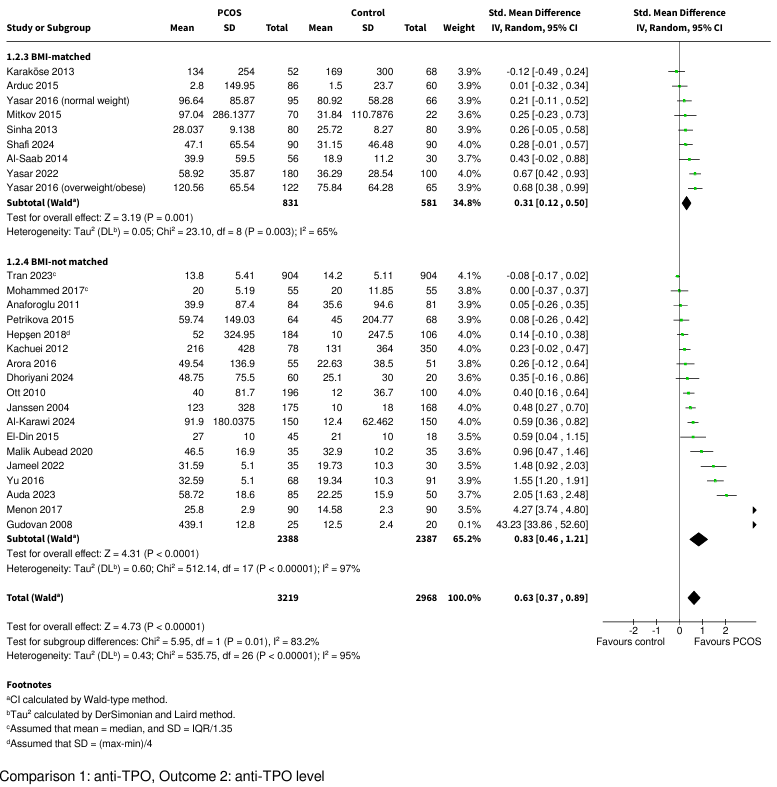

Supplement: Supplementary file 1 [file ijms-26-07525-s001.zip › Supplementary material S4 – sensitivity analyses anti-TPO level/SUPPLEMENTARY MATERIAL S4AX.png]

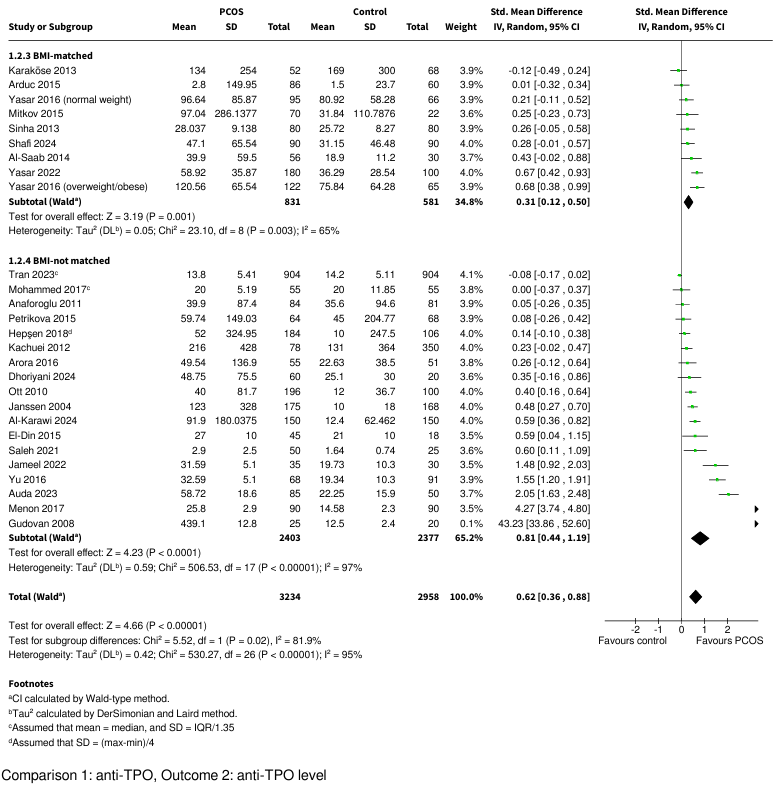

Supplement: Supplementary file 1 [file ijms-26-07525-s001.zip › Supplementary material S4 – sensitivity analyses anti-TPO level/SUPPLEMENTARY MATERIAL S4AY.png]

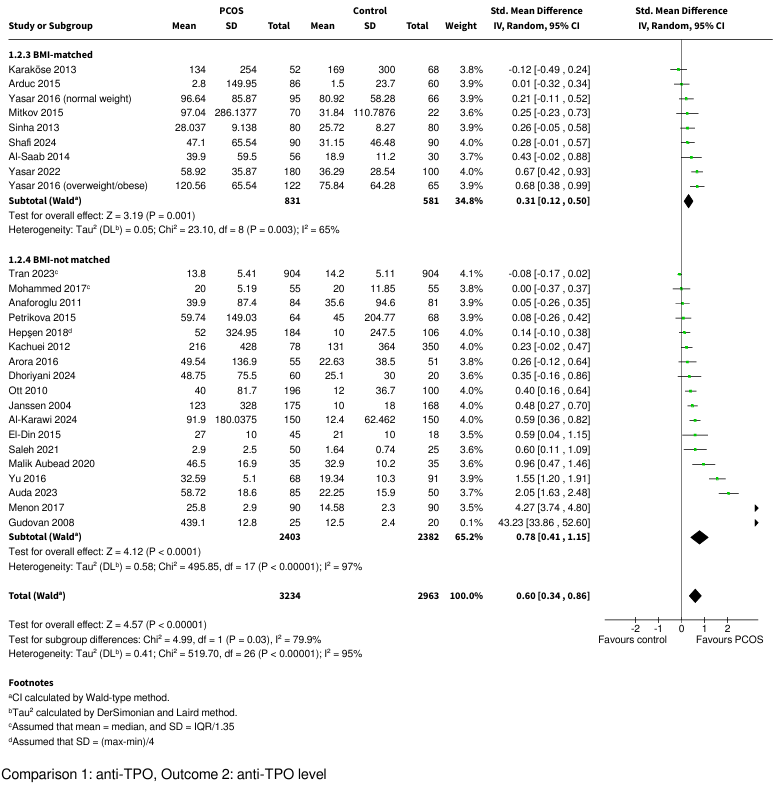

Supplement: Supplementary file 1 [file ijms-26-07525-s001.zip › Supplementary material S4 – sensitivity analyses anti-TPO level/SUPPLEMENTARY MATERIAL S4AZ.png]

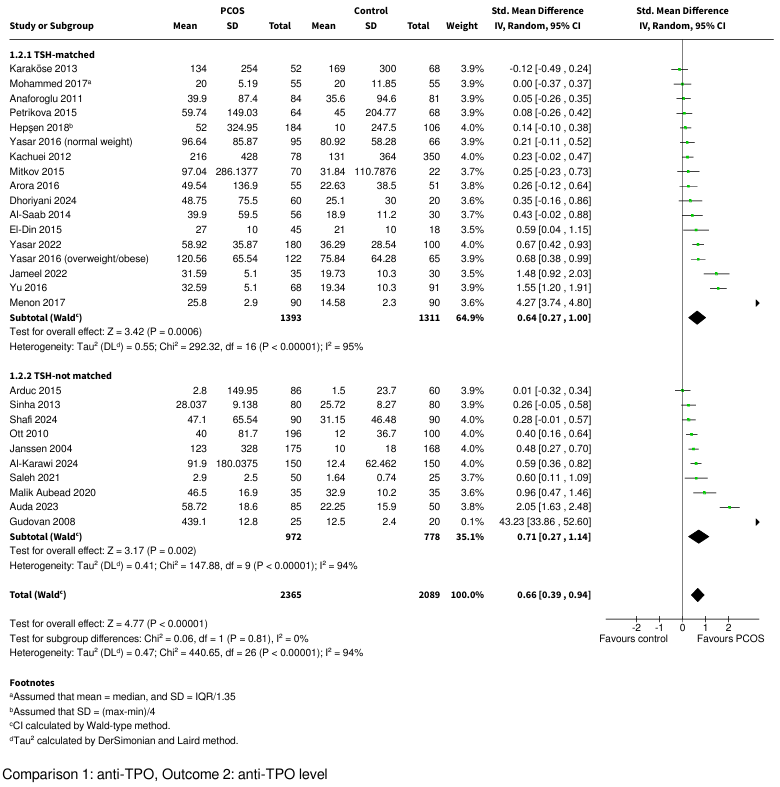

Supplement: Supplementary file 1 [file ijms-26-07525-s001.zip › Supplementary material S4 – sensitivity analyses anti-TPO level/SUPPLEMENTARY MATERIAL S4B.png]

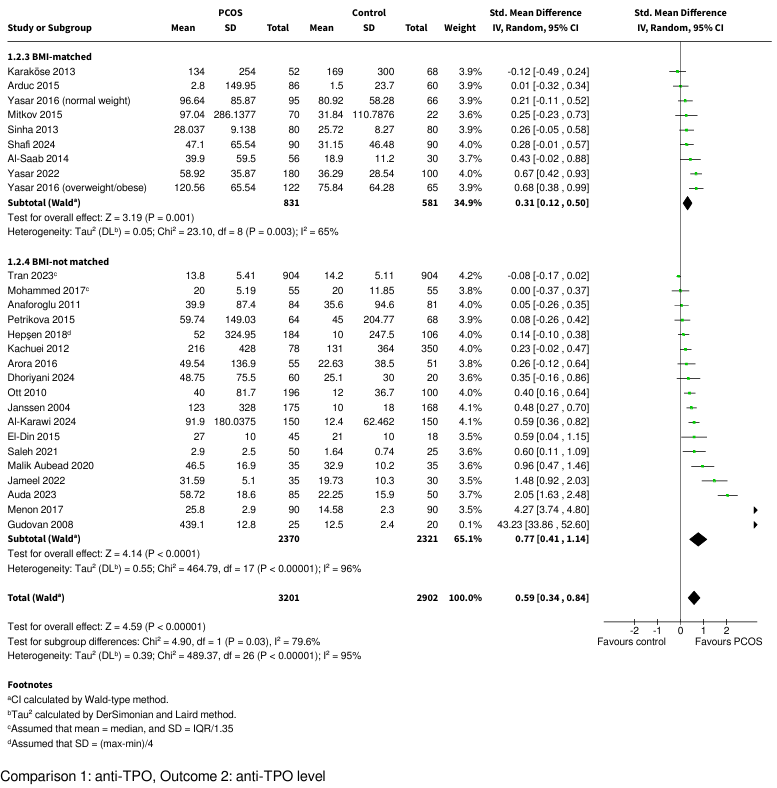

Supplement: Supplementary file 1 [file ijms-26-07525-s001.zip › Supplementary material S4 – sensitivity analyses anti-TPO level/SUPPLEMENTARY MATERIAL S4BA.png]

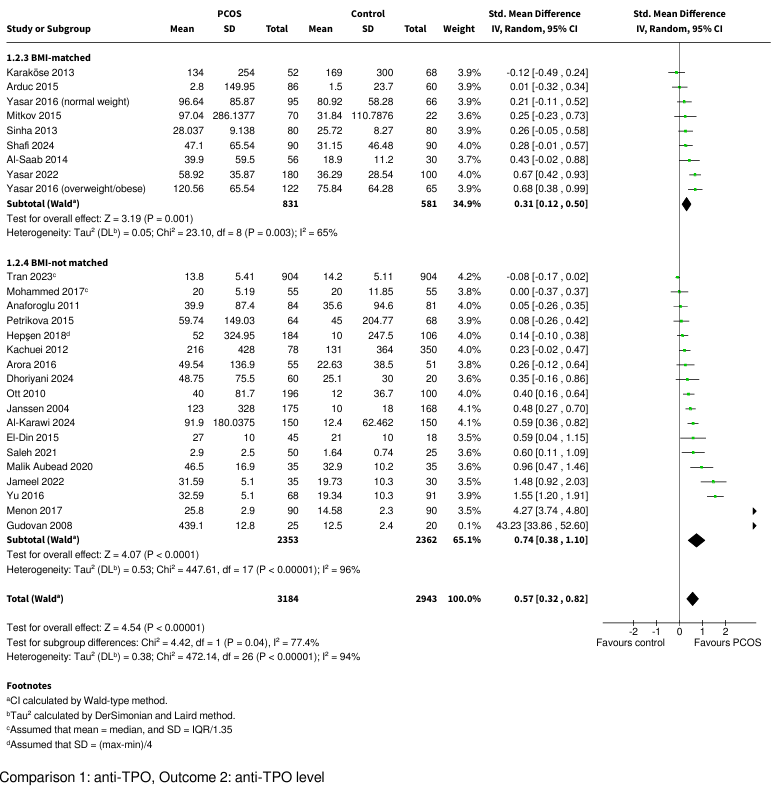

Supplement: Supplementary file 1 [file ijms-26-07525-s001.zip › Supplementary material S4 – sensitivity analyses anti-TPO level/SUPPLEMENTARY MATERIAL S4BB.png]

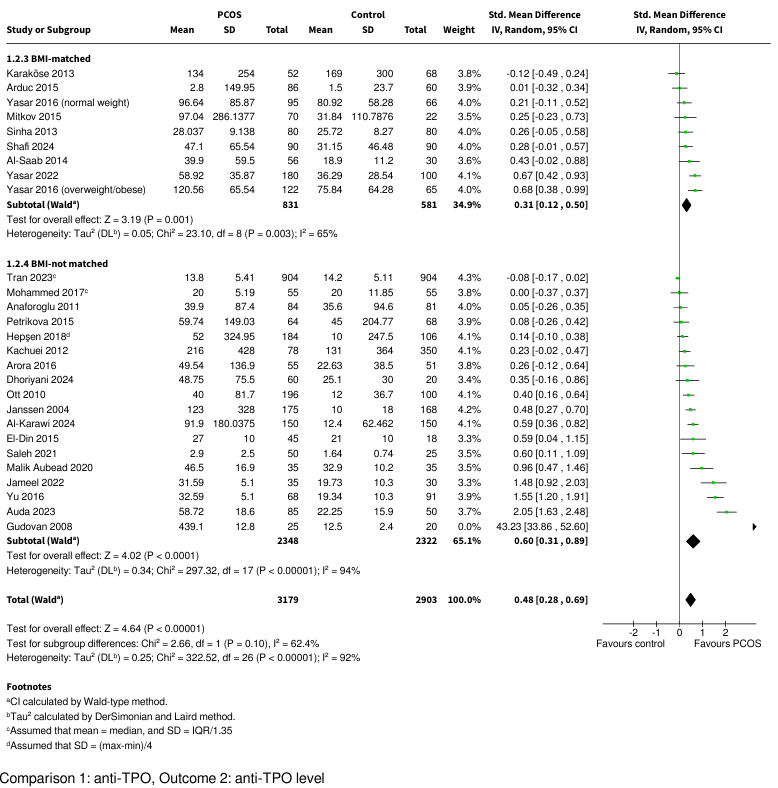

Supplement: Supplementary file 1 [file ijms-26-07525-s001.zip › Supplementary material S4 – sensitivity analyses anti-TPO level/SUPPLEMENTARY MATERIAL S4BC.png]

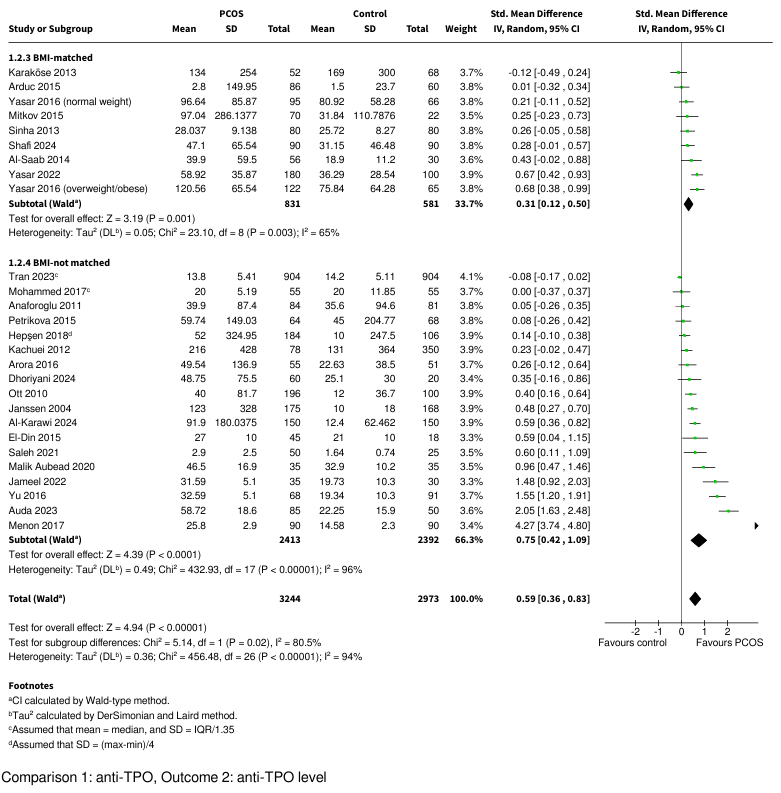

Supplement: Supplementary file 1 [file ijms-26-07525-s001.zip › Supplementary material S4 – sensitivity analyses anti-TPO level/SUPPLEMENTARY MATERIAL S4BD.png]

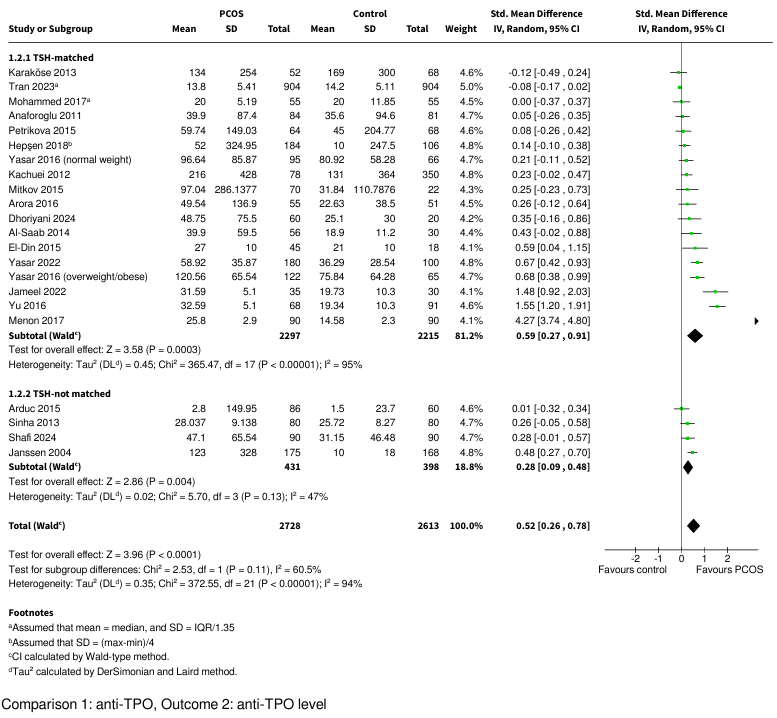

Supplement: Supplementary file 1 [file ijms-26-07525-s001.zip › Supplementary material S4 – sensitivity analyses anti-TPO level/SUPPLEMENTARY MATERIAL S4BE.png]

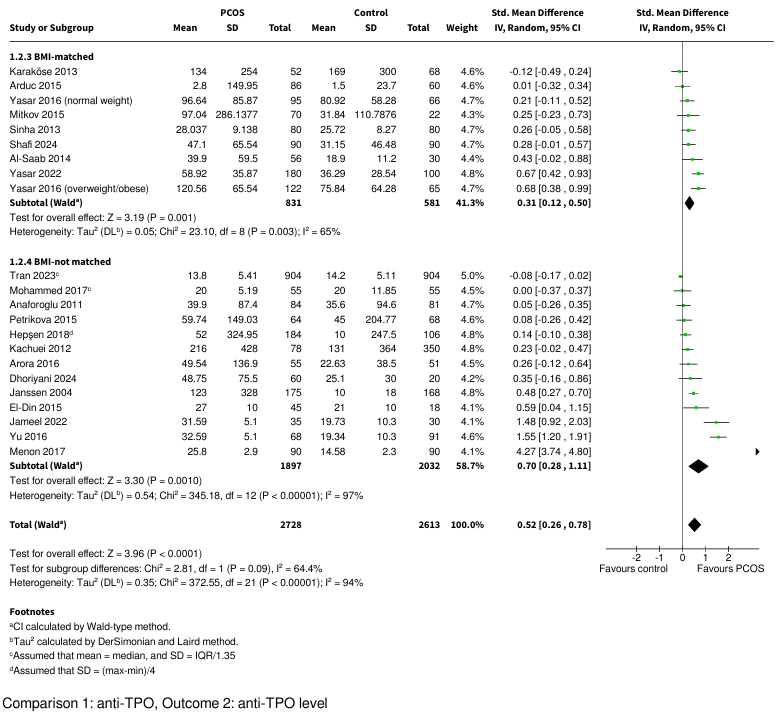

Supplement: Supplementary file 1 [file ijms-26-07525-s001.zip › Supplementary material S4 – sensitivity analyses anti-TPO level/SUPPLEMENTARY MATERIAL S4BF.png]

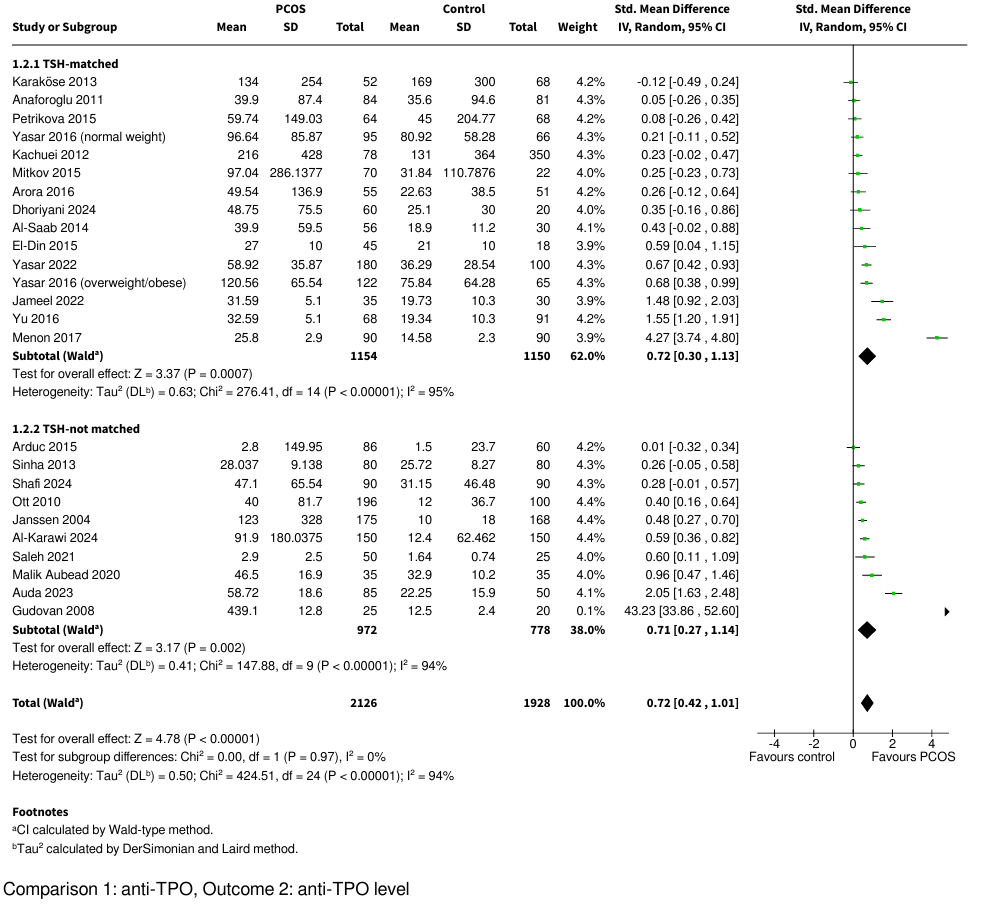

Supplement: Supplementary file 1 [file ijms-26-07525-s001.zip › Supplementary material S4 – sensitivity analyses anti-TPO level/SUPPLEMENTARY MATERIAL S4BG.png]

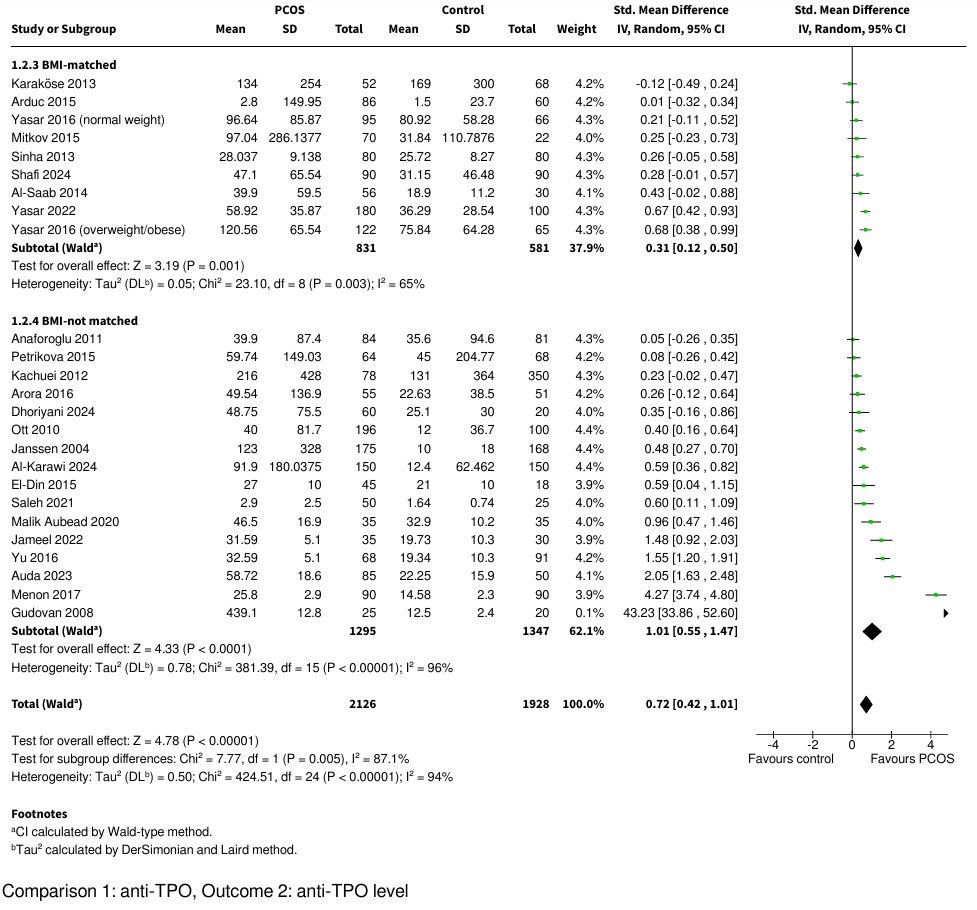

Supplement: Supplementary file 1 [file ijms-26-07525-s001.zip › Supplementary material S4 – sensitivity analyses anti-TPO level/SUPPLEMENTARY MATERIAL S4BH.png]

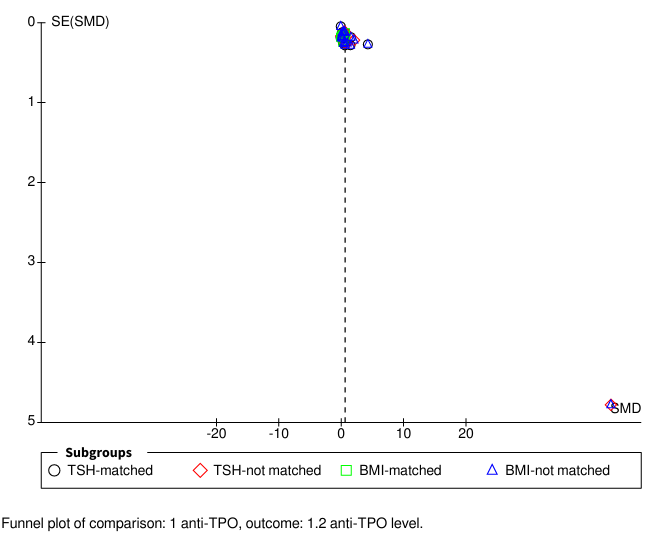

Supplement: Supplementary file 1 [file ijms-26-07525-s001.zip › Supplementary material S4 – sensitivity analyses anti-TPO level/SUPPLEMENTARY MATERIAL S4BI.png]

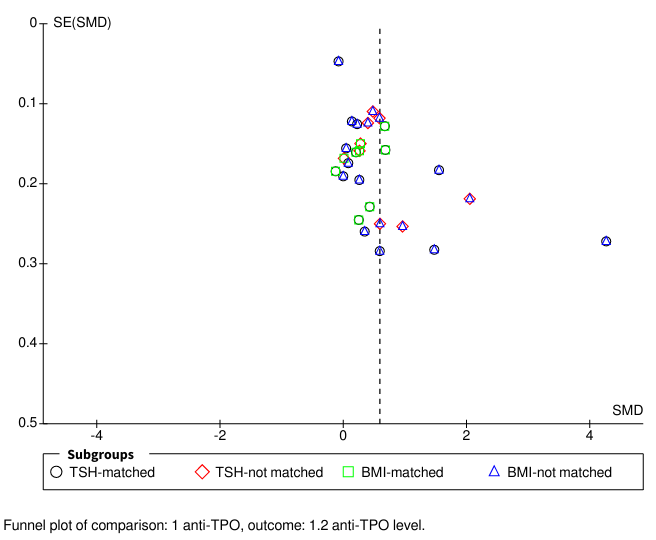

Supplement: Supplementary file 1 [file ijms-26-07525-s001.zip › Supplementary material S4 – sensitivity analyses anti-TPO level/SUPPLEMENTARY MATERIAL S4BJ.png]

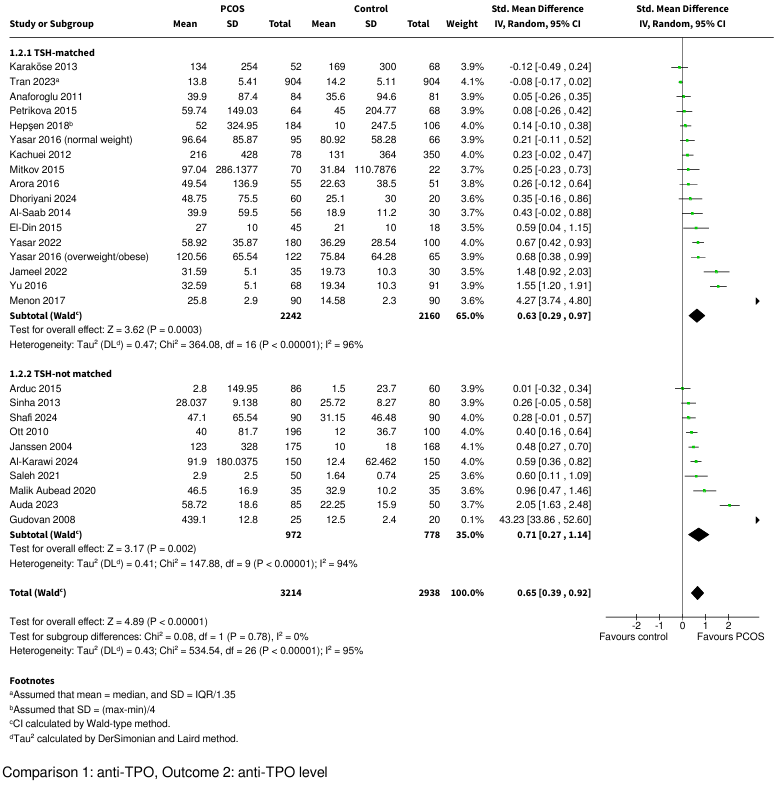

Supplement: Supplementary file 1 [file ijms-26-07525-s001.zip › Supplementary material S4 – sensitivity analyses anti-TPO level/SUPPLEMENTARY MATERIAL S4C.png]

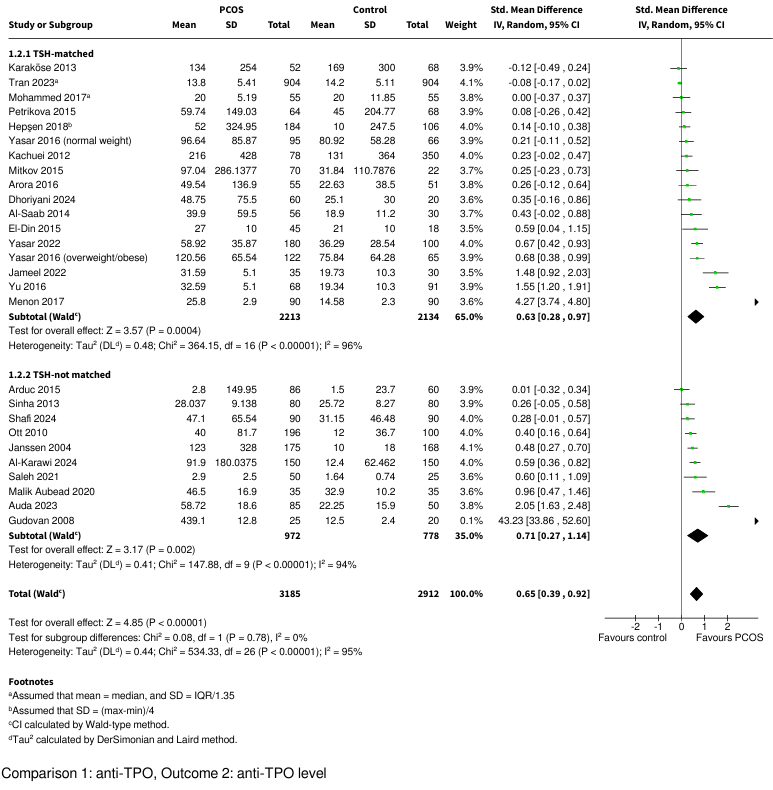

Supplement: Supplementary file 1 [file ijms-26-07525-s001.zip › Supplementary material S4 – sensitivity analyses anti-TPO level/SUPPLEMENTARY MATERIAL S4D.png]

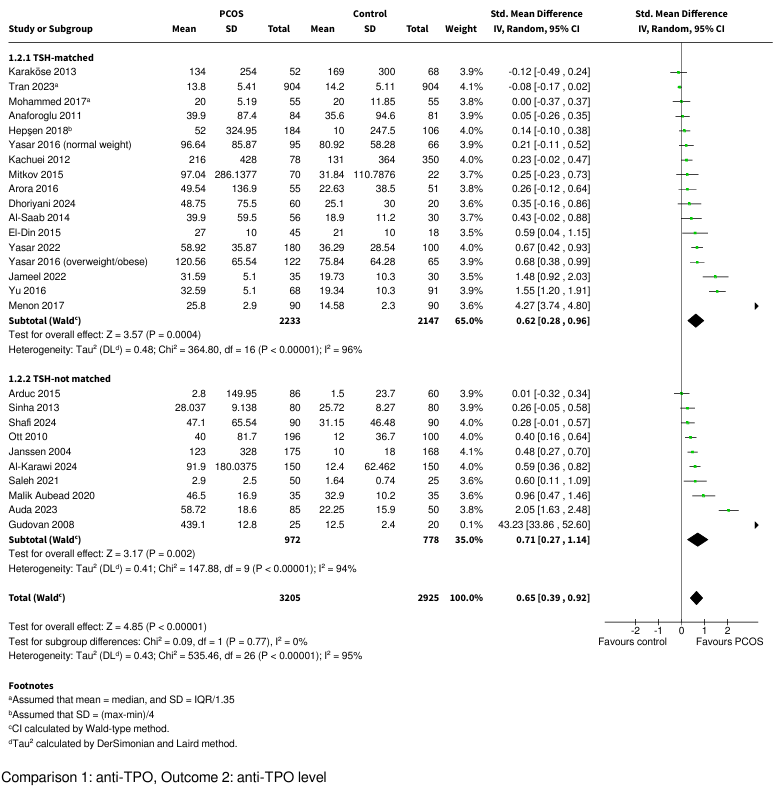

Supplement: Supplementary file 1 [file ijms-26-07525-s001.zip › Supplementary material S4 – sensitivity analyses anti-TPO level/SUPPLEMENTARY MATERIAL S4E.png]

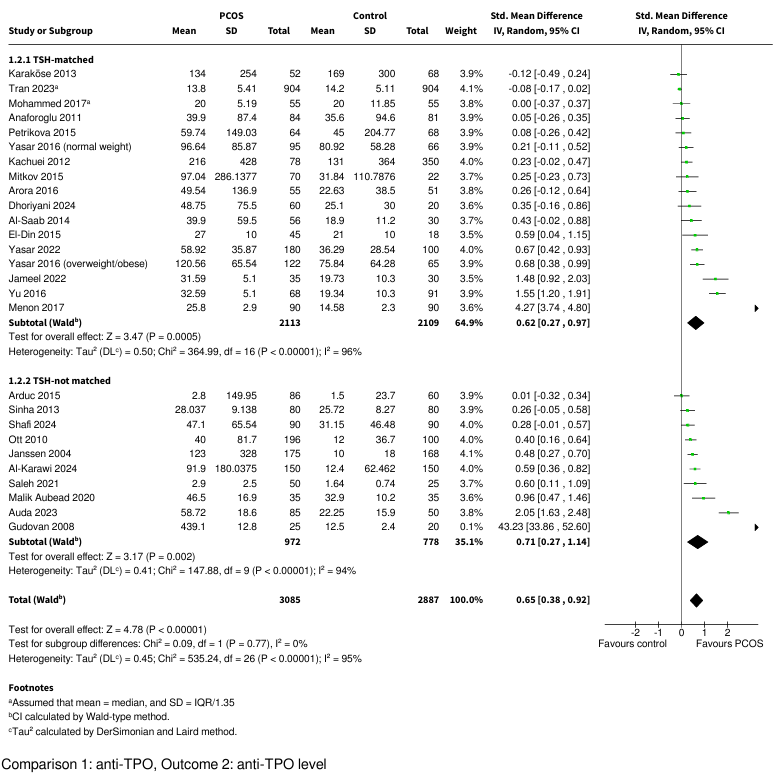

Supplement: Supplementary file 1 [file ijms-26-07525-s001.zip › Supplementary material S4 – sensitivity analyses anti-TPO level/SUPPLEMENTARY MATERIAL S4F.png]

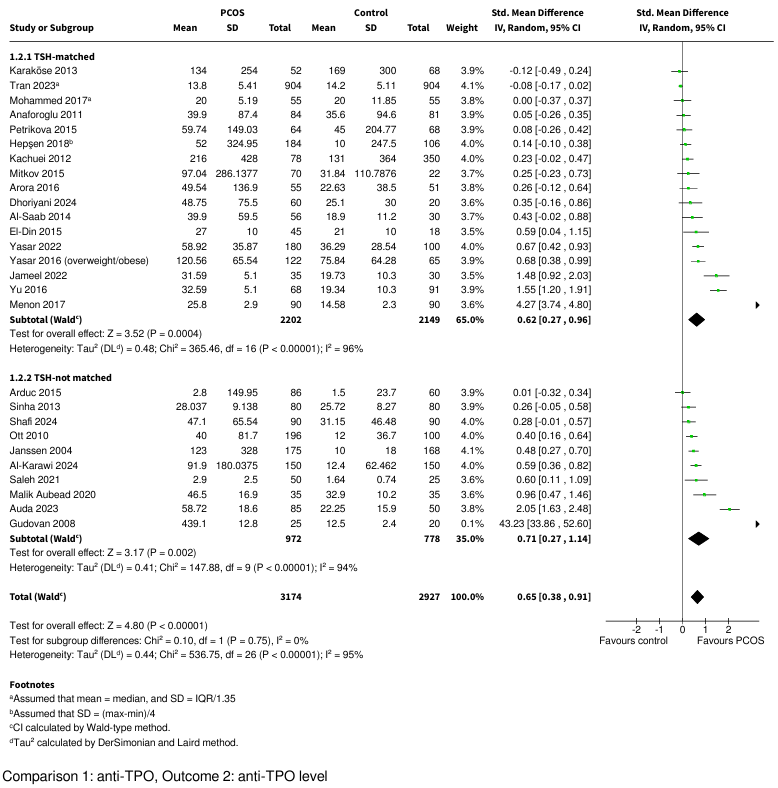

Supplement: Supplementary file 1 [file ijms-26-07525-s001.zip › Supplementary material S4 – sensitivity analyses anti-TPO level/SUPPLEMENTARY MATERIAL S4G.png]

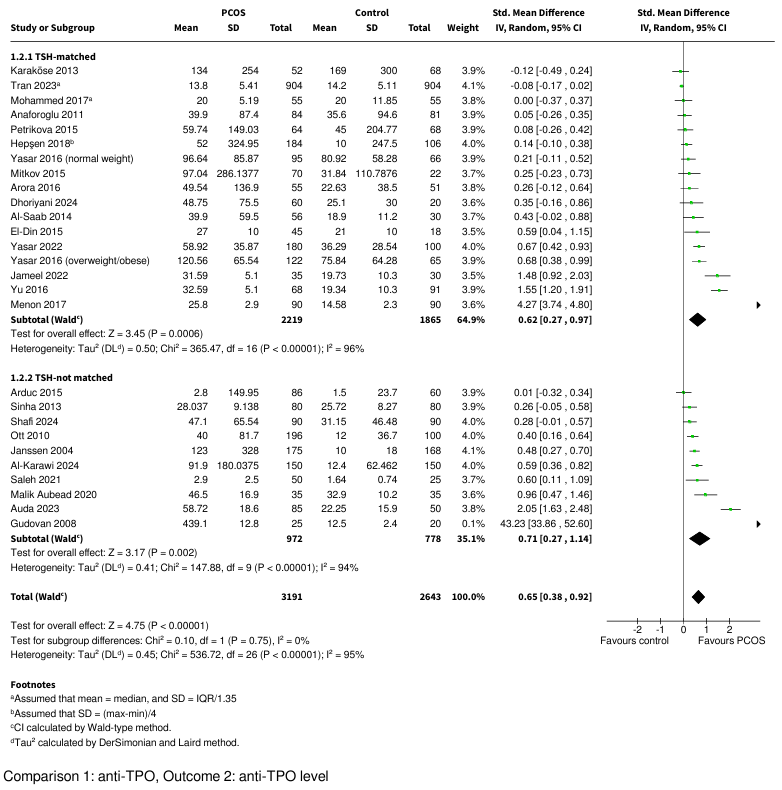

Supplement: Supplementary file 1 [file ijms-26-07525-s001.zip › Supplementary material S4 – sensitivity analyses anti-TPO level/SUPPLEMENTARY MATERIAL S4H.png]

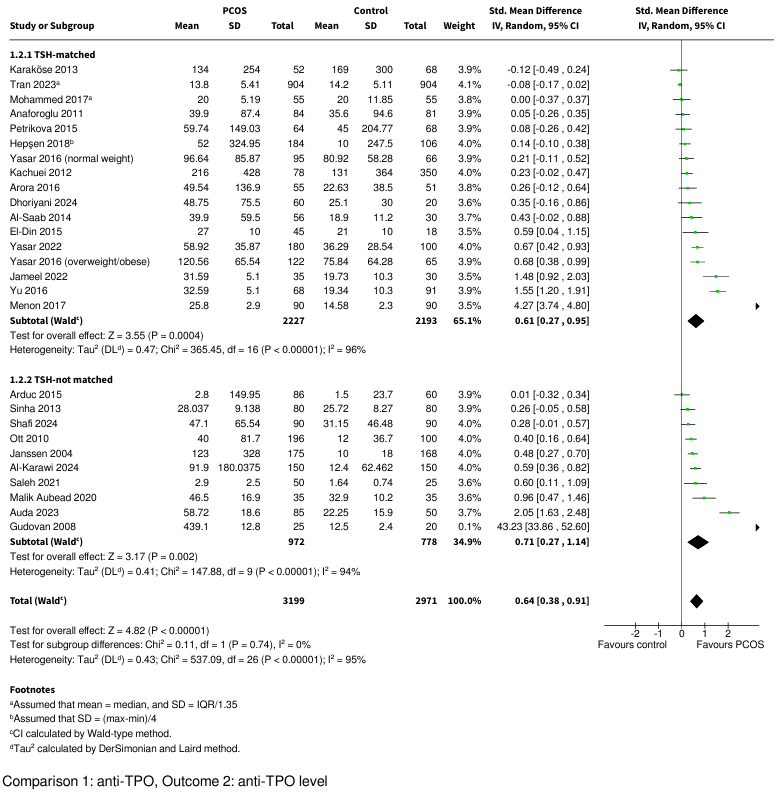

Supplement: Supplementary file 1 [file ijms-26-07525-s001.zip › Supplementary material S4 – sensitivity analyses anti-TPO level/SUPPLEMENTARY MATERIAL S4I.png]

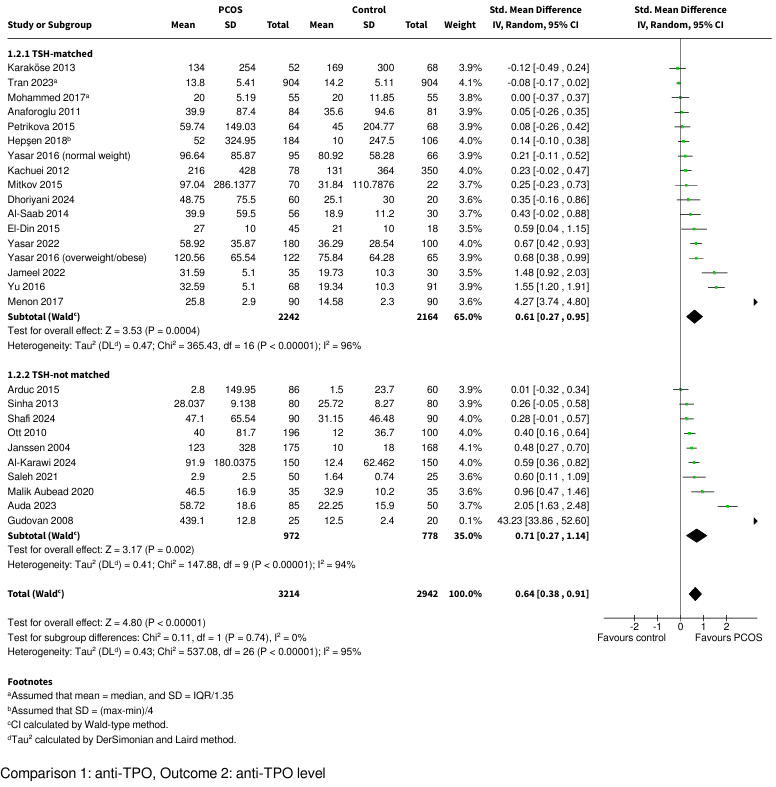

Supplement: Supplementary file 1 [file ijms-26-07525-s001.zip › Supplementary material S4 – sensitivity analyses anti-TPO level/SUPPLEMENTARY MATERIAL S4J.png]

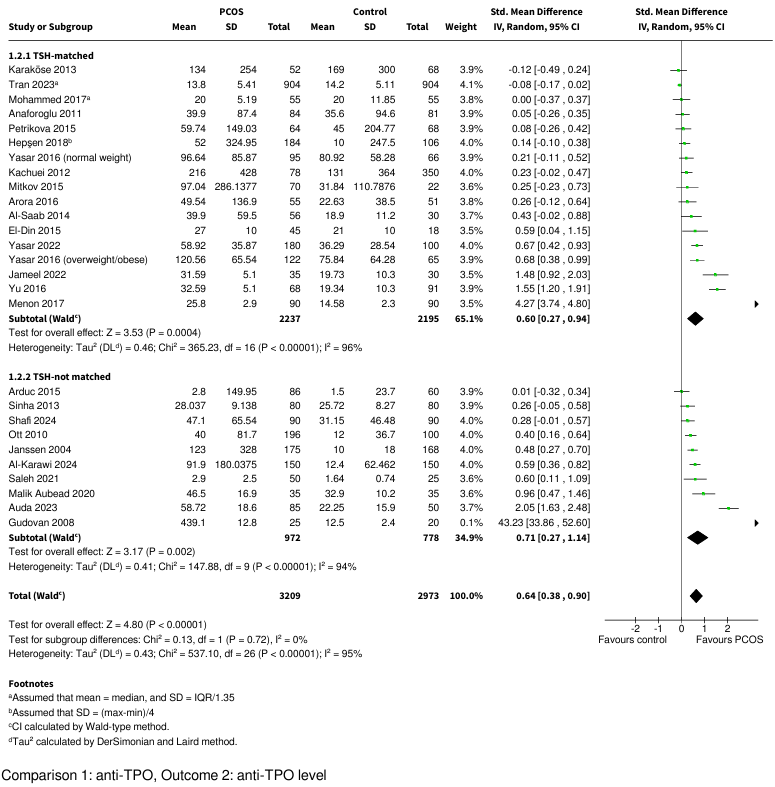

Supplement: Supplementary file 1 [file ijms-26-07525-s001.zip › Supplementary material S4 – sensitivity analyses anti-TPO level/SUPPLEMENTARY MATERIAL S4K.png]

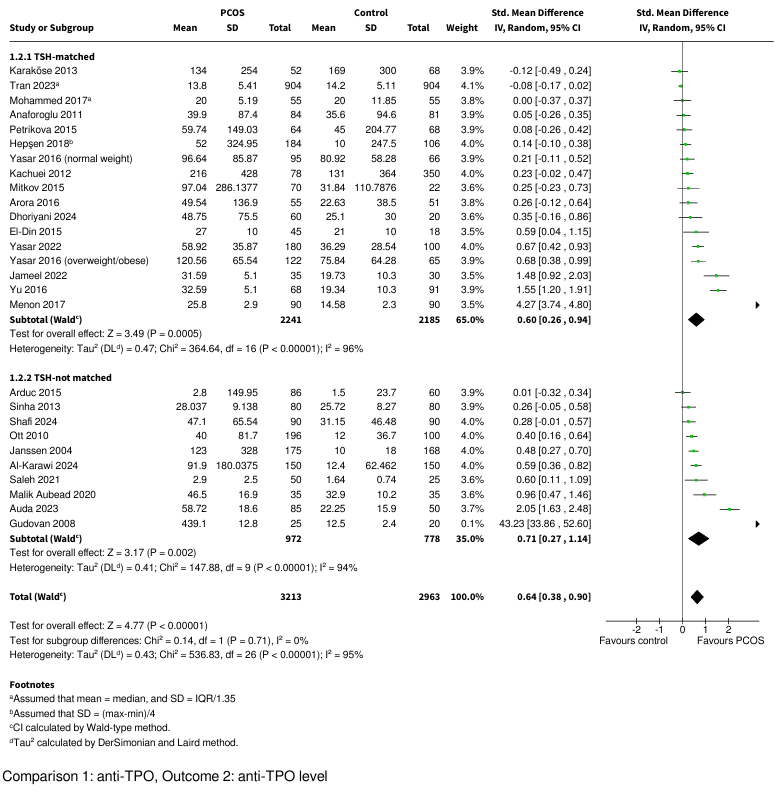

Supplement: Supplementary file 1 [file ijms-26-07525-s001.zip › Supplementary material S4 – sensitivity analyses anti-TPO level/SUPPLEMENTARY MATERIAL S4L.png]

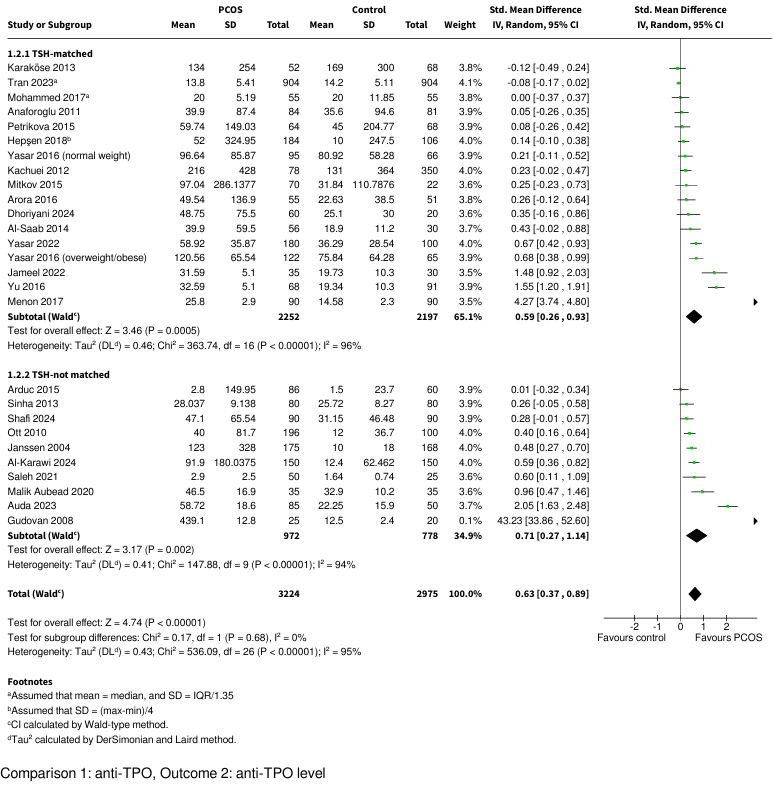

Supplement: Supplementary file 1 [file ijms-26-07525-s001.zip › Supplementary material S4 – sensitivity analyses anti-TPO level/SUPPLEMENTARY MATERIAL S4M.png]
